# EV71 5’UTR interacts with 3D protein affecting replication through the AKT-mTOR pathway

**DOI:** 10.1186/s12985-024-02385-z

**Published:** 2024-05-22

**Authors:** Xiaoying Xu, Shao Ma, Ziwei Liu, Haowen Yuan, Yao Wang, Mengting Chen, Mengyu Du, Haopeng Kan, Zequn Wang, Xiaowen Chong, Hongling Wen

**Affiliations:** 1 School of Public Health, Cheeloo College of Medicine, Shandong University, No. 44 Wenhua West Road, Lixia District, Jinan, 250012 China; 2 Department of Breast Surgery, QiLu Hospital of Shandong University, Jinan, Shandong 250012 People’s Republic of China; 3 Jinan Center For Disease Control And Prevention, Jinan, Shandong 250014 China

**Keywords:** EV71, 5′UTR, 3D protein, Co-mutation, Autophagy, AKT-mTOR pathway

## Abstract

**Background:**

EV71 is one of the important pathogens of Hand-foot-and-mouth disease (HFMD), which causes serious neurological symptoms. Several studies have speculated that there will be interaction between 5′UTR and 3D protein. However, whether 5′UTR interacts with the 3D protein in regulating virus replication has not been clarified.

**Methods:**

Four 5'UTR mutation sites (nt88C/T, nt90-102-3C, nt157G/A and nt574T/A) and two 3D protein mutation sites (S37N and R142K) were mutated or co-mutated using virulent strains as templates. The replication of these mutant viruses and their effect on autophagy were determined.

**Results:**

5'UTR single-point mutant strains, except for EGFP-EV71(nt90-102-3C), triggered replication attenuation. The replication ability of them was weaker than that of the parent strain the virulent strain SDLY107 which is the fatal strain that can cause severe neurological complications. While the replication level of the co-mutant strains showed different characteristics. 5 co-mutant strains with interaction were screened: EGFP-EV71(S37N-nt88C/T), EGFP-EV71(S37N-nt574T/A), EGFP-EV71(R142K-nt574T/A), EGFP-EV71(R142K-nt88C/T), and EGFP-EV71(R142K-nt157G/A). The results showed that the high replicative strains significantly promoted the accumulation of autophagosomes in host cells and hindered the degradation of autolysosomes. The low replicative strains had a low ability to regulate the autophagy of host cells. In addition, the high replicative strains also significantly inhibited the phosphorylation of AKT and mTOR.

**Conclusions:**

EV71 5'UTR interacted with the 3D protein during virus replication. The co-mutation of S37N and nt88C/T, S37N and nt574T/ A, R142K and nt574T/A induced incomplete autophagy of host cells and promoted virus replication by inhibiting the autophagy pathway AKT-mTOR. The co-mutation of R142K and nt88C/T, and R142K and nt157G/A significantly reduced the inhibitory effect of EV71 on the AKT-mTOR pathway and reduced the replication ability of the virus.

**Supplementary Information:**

The online version contains supplementary material available at 10.1186/s12985-024-02385-z.

## Introduction

Enterovirus 71 (EV71), a member of the Enterovirus family Picronaviridae, is the main pathogen of HFMD, which infects infants under the five years and can lead to serious neurological symptoms. It is one of the major public health problems in Asia and other regions [1]. At present, EV71 is widely concerned because of its high mutation rate and lack of specific drugs [2].

Enterovirus 71 (EV71), which belongs to the Picornaviridae family, contains only one open reading frame (ORF) flanked by 5′ and 3′ untranslated regions (UTRs). ORF encodes four structural proteins (VP1-VP4) and seven nonstructural proteins (2 A ~ C, 3 A ~ D), constituting the viral capsid and participating in virus replication. The 5′UTR of EV71 includes a clover leaf structure and an internal ribosome entry site (IRES), which folds into multiple spatial structures and changes the characteristics of the virus with the gene mutations. IRES can regulate the translation process of viruses by interacting with host proteins, such as hnRNP A1 or IRES trans-acting factors, which affects the transformation process of viral protein and virus replication [3–5]. In addition, research has found that some of the virulence typing bases of Poliovirus are located on IRES, such as 480, 481, and 472 [6–8]. Studies have shown that viral nucleic acids undergo extensive replication under the regulatory effect of 5’UTR which interacts with Poly C protein, thereby promoting the transition of the virus from protein translation to viral RNA synthesis [9]. Thus, we selected four sites (nt88 C/T, nt90-102–3 C, nt157 G/A, nt574 T/A) from different domains of 5’UTR for the construction of mutant viruses. EV71 3D protein, whose crystal is a “right hand” conformation, including “finger”, “palm” and “thumb” domains, is mainly responsible for the extension of RNA strands during virus replication as RNA-dependent RNA polymerase(RdRp). The 3D polymerase can interact with host factor Annexin A2(ANXA2) and promote the replication of EV71 [10]. More importantly, studies have proved that 5′UTR and 3D protein jointly regulate virus replication in a variety of ways. EV71 activates the production of silent mating type information regulation 2 home 1(SIRT1), which can react with 5′UTR and 3D at the same time, to reduce virus replication and translation [11]. In addition, it is reported that the 3D finger domain will be combined with 5′UTR from the crystal structure, which may have been utilized by interacting with RNA bases to optimize 3D protein function [12]. And the importance of the fingers-thumb domain interaction for the function and structural stability of 3D protein cannot be ignored [13]. In this study we selected two key amino acid sites Site-37 ( S37N) and Site-142 ( R142K), which located respectively in the thumb and finger domain of the 3D protein, to explore the influence and interaction of 5′UTR and 3D protein in virus replication.

Autophagy, which is a fundamental catabolic process, degrades long-lived cellular proteins and recycles damaged organelles for the maintenance of cellular and tissue homeostasis, as well as in viral infection and pathogenesis. However, during a long evolutionary process, autophagy is hijacked by many viruses to form an immune escape mechanism, which provides convenient conditions for virus replication and transmission, such as classical swine fever virus [14], Newcastle disease virus [15], dengue virus [16], hepatitis B virus [17] and so on. Enterovirus 71 can also promote virus replication by inducing an increase in host autophagy [18]. Viruses can hijack host autophagy in a variety of ways, including directly inhibiting autophagy activation by blocking the function of host ATG protein, inhibiting downstream degradation pathways of autophagy, or destroying host autophagy [19]. In 2009, researchers demonstrated that EV71 can induce cell autophagy and promote replication through a combination of in vitro and in vivo experiments [20]. Since then, there has been an increasing amount of research on EV71 regulating autophagy and promoting replication. For example, EV71 regulates has-miR-30a which promotes the expression of Beclin-1 (a gene that promotes autophagy) in the early stages of autophagosome formation to promote viral replication [21].

Mammalian target of rapamycin (mTOR) is a relay station of cellular metabolism, which can regulate many activities such as cell growth, autophagy, and apoptosis. mTOR is an important signal molecule, affected by a variety of upstream proteins, and plays a role in a variety of signal pathways regulating cell metabolism. Activation of the AMPK/mTOR pathway can regulate autophagy and thus alleviate viral myocarditis [22]. Porcine epidemic diarrhea virus activates autophagy induced by PI3K / AKT / mTOR pathway through nonstructural protein 6, thereby promoting virus replication [23]. As a key regulator of autophagy, the mammalian target of phosphokinase (PI3K) / protein kinase B (AKT) / rapamycin (mTOR) pathway has diverse interactions with autophagy. Moreover, our previous study showed that the EV71 VP1 protein regulates autophagy in nerve cells through the mTOR molecule [24]. However, the upstream molecules of mTOR are complex, and there is no study to prove the role of AKT on mTOR during EV71 infection.

**Fig. 1:**
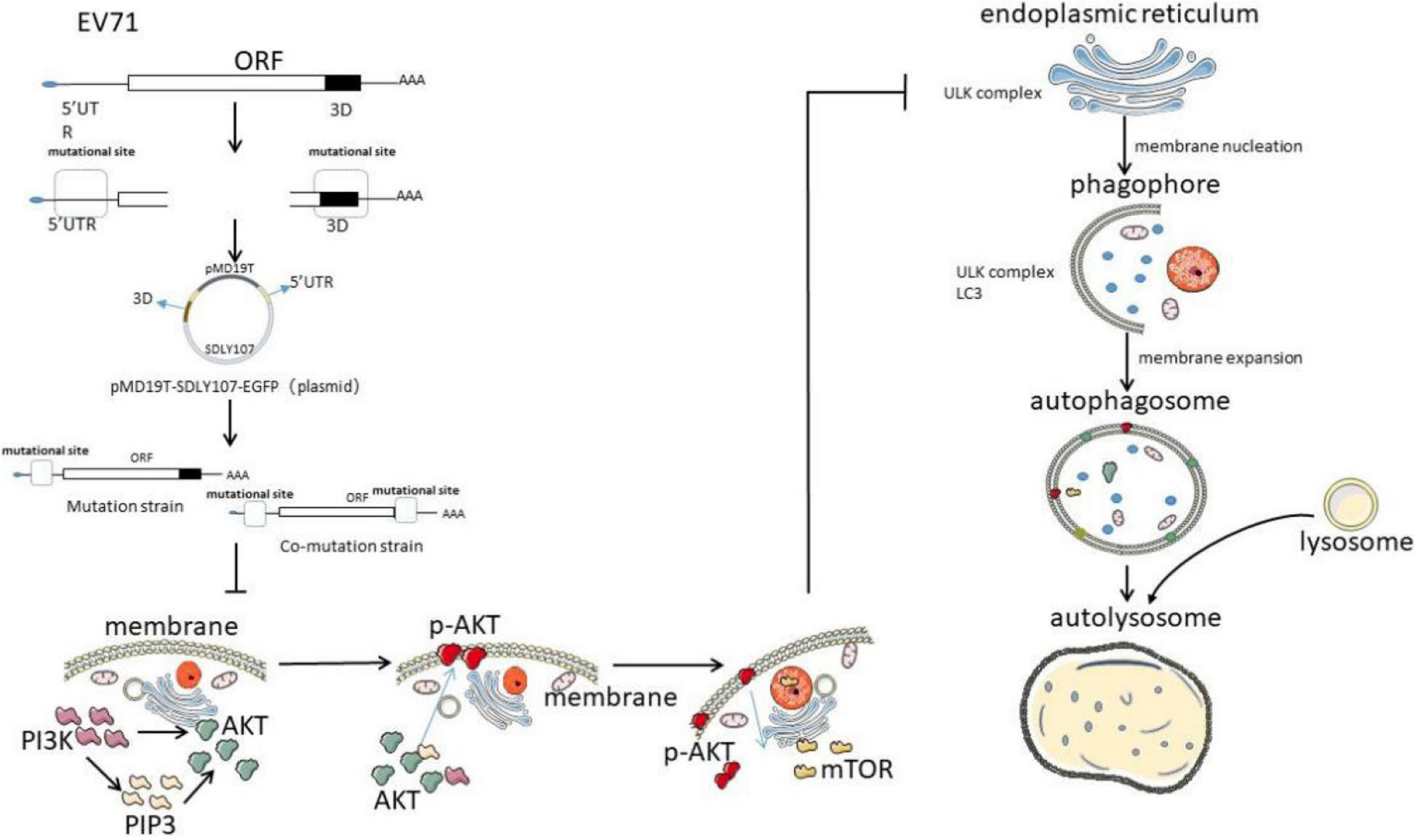
Mutated viruses regulate autophagy through AKT-mTOR

In this study, four 5′UTR mutation sites (nt88C/T, nt90-102–3 C, nt157G/A and nt574T/A) and two 3D protein mutation sites (S37N and R142K) were mutated or co-mutated using virulent strains as templates. The replication of these mutant viruses and their effect on autophagy were determined, which provides a new idea for the research of EV71 pathogenesis and specific drug development. The research process is shown in Fig. 1.

## Materials and methods

### Cells and viruses

Human neuroblastoma (SH-SY5Y) cells kept by our laboratory, were cultured in minimum essential medium (MEM, USA, Gibco) containing 15% heat-inactivated fetal bovine serum (FBS, Gibco) at 37℃. Rhabdomyosarcoma (RD) cells kept by our laboratory, were cultured in MEM medium supplemented with 10%FBS at 37℃.

The virulent strain SDLY107 was isolated from a fatal patient, causing severe neurological complications, and the attenuated strain SDLY1 was isolated from a patient without neuro-virulence. In vivo and in vitro experiments, we have previously confirmed that SDLY107 can cause severe neurological symptoms, while SDLY1 cannot. EGFP-EV71 constructed with fluorescent gene clones of the virulent strain, observed no significant differences in biological characteristics with wild-type virulent strain SDLY107 [25]. Four 5′UTR mutation sites (nt88C/T, nt90-102–3 C, nt157G/A and nt574T/A) and two 3D protein mutation sites (S37N and R142K) were mutated or co-mutated using virulent strains as templates. Four 5′UTR mutant strains(EGFP-EV71(nt88C/T), EGFP-EV71(nt90-102–3 C), EGFP-EV71(nt157G/A), EGFP-EV71(nt574T/A)) and eight co-mutant strains(EGFP-EV71(S37N-nt88C/T), EGFP-EV71(S37N-nt90-102–3 C), EGFP-EV71(S37N-nt157G/A), EGFP-EV71(S37N-nt574T/A), EGFP-EV71(R142K-nt88C/T), EGFP-EV71(R142K-nt90-102–3 C), EGFP-EV71(R142K-nt157G/A). EGFP-EV71(R142K-nt574T/A)), were successfully rescued by the infectious cDNA of virulent strain as a template (Table 1).

**Table 1:** Primers used in this study

Name	Sequence (5′→3′)	sites	Length(bp)
PF	TGATTACGCCAAGCTTTTAAAACAGCCTGTGGGTTGC	1–21^a^	
PR	GGCATTGCACTGCACGTGGATGCAGAACCCTGATCGG	1255–1291	
PF1	CTGTTTTA*C*(T)ACCCCCCCCCCAGTGA	80–104	100
PR1	GGGGGGT*G*(A)TAAAACAGGCGCACAA	72–95	1900
PF2	CCTGTTTTATA*CCC*CCCCCCCCCCAGTGAAAC	79–107	150
PR2	GCTTCTAAGTTTCACTGGGGGGGGGG*GGG*TATA	86–115	1850
PF3	GCGCTCCAGTT*G*(A)TGTCTTGATCAAGCAC	143–170	170
PR3	CTTGATCAAGACA*C*(T)AACTGGAGCGCTATGC	138–167	1830
PF4	GTCCGTGTTTCCTTTTA*T*(A)CTTTATACTGGCTGCT	554–587	600
PR4	GCAGCCAGTATAAAG*A*(T)TAAAAGGAAACACGGAC	554–586	1400

^a^Partial sites are located on the pMD19T

### Antibodies and chemical reagents

The antibodies anti-LC3B(Cat#2775), anti-AKT(Cat#4060), anti-phospho-AKT(Cat#31,957), anti-mTOR (Cat#2983), and anti-phospho-mTOR (Cat#5536) used in the study were purchased from Cell Signaling Technology. Anti-p62(ab109012) was purchased from Abcam. And the antibodies used were FICT-conjugated Affinipure Goat Anti-Mouse IgG (ZSGB-BIO), HPR-conjugated Affinipure Goat Anti-Rabbit IgG (Proteintech, SA-00001-2), and HPR-conjugated Affinipure Goat Anti-Mouse IgG (Proteintech, SA-00001-1). The chemical reagent used in the study was Afuresertib (TargetMol, T1911).

### Virus quantification

Quantification of viruses was determined by the amount of infectious viral particle (CCID50). Medium used throughout the test was MEM supplemented with 1% FBS for RD cell. The virus solution after multiple dilution was added to 96 well, in which cell density was 80%. The CCID50 assay lasted for Karber method.

### Virus infection

RD cells and SH-SY5Y cells were infected with viruses at the same multiplicity of infection (MOI = 1). After washed repeatedly and gently with PBS, they were replenished with fresh MEM containing 1% FBS. In the experiment of adding the AKT inhibitor, the cells were pretreated with the inhibitor (dissolved in DMSO) for 2 h before viral infection. And then performed the above operation.

### Replication kinetics

The cells were cultured in the cell culture plate with 90%~95% confluency, and infected by viruses at MOI = 1. The culture supernatants were sampled every 12 h post infection. Subsequently, the total viral RNAs was extracted with the RNA extraction kit (E.Z.N.A.® Viral RNA Kit, Omega, Guangzhou, China). The replication kinetics of each strain was determined by real-time quantitative PCR (qRT-PCR) and repeated three times to take the average value.

### Detection of protein by western blotting

In the experiment of detecting protein, cells were lysed with RIPA lysis buffer containing 0.1% phenylmethylsulfonyl fluoride (PMSF) at 4℃, and whole cell extracts were prepared by centrifugation and boiled. Then the protein concentration was measured with the BCA Protein Assay Kit (Beyotime Protein Assay Kit, China). The protein samples of the same quality were isolated by SDS polyacrylamide gel electrophoresis, and were transferred to 0.22 μm PVDF membrane. Membranes were blocked with 5% skim milk for 1 h. Finally, the protein bands were detected by ultra-sensitive multifunctional imager after the membranes incubated with primary antibodies and corresponding horseradish peroxidase conjugated secondary antibodies.

## Results

### Construction and rescue of mutant viruses

**Fig. 2:**
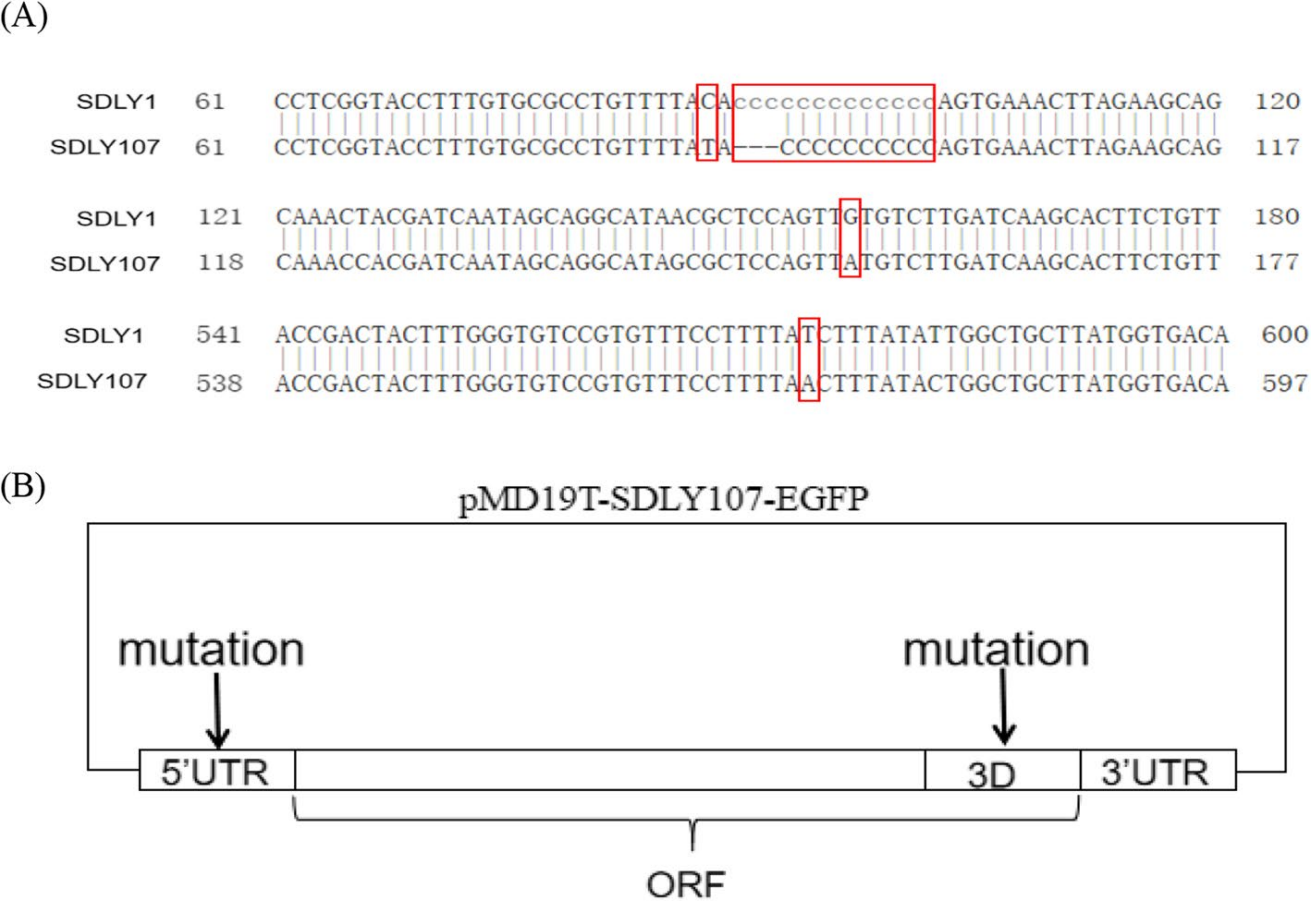
Construction of mutated viruses. **A** The 5'UTR sequences of the attenuated strain and virulent strain were compared, and four key sites were screened according to the 5'UTR sequence and structure of different virus strains. **B** The position of 5'UTR and 3D protein in the pMD19T-SDLY107-EGFP. When constructing a 5'UTR single mutant virus, only one site of 5'UTR is mutated. While, when constructing a co-mutant virus, a 5'UTR and a 3D protein site were co-mutated

Four mutation sites were selected according to the 5′UTR sequence and structure of different virus strains (Fig. 2A). Four 5′UTR mutant viruses, EGFP-EV71 (nt88C/T), EGFP-EV71 (nt90-102–3 C), EGFP-EV71 (nt157G/A), EGFP-EV71 (nt574T/A), which selected from the clover leaf structure、the interval between clover leaf、 SLII and SLVI successively, were constructed and saved to explore the impact of the mutation on virus replication. Then eight co-mutation strains, EGFP-EV71(S37N-nt88C/T)、EGFP-EV71(S37N-nt90-102–3 C)、EGFP-EV71(S37N-nt157G/A) 、EGFP-EV71(S37N-nt574T/A)、EGFP-EV71(R142K-nt88C/T)、EGFP-EV71(R142K-nt90-102–3 C)、EGFP-EV71(R142K-nt157G/A)、EGFP-EV71(R142K-nt574T/A), were rescued to study the interaction between 5′UTR and 3D protein based on the two 3D protein mutation sites (S37N and R142K) Table 2.

**Table 2:** The numbering of the mutated viruses

3D protein	5′UTR			
	nt88C/T(M1)	nt90-102–3 C(M2)	nt157G/A(M3)	nt574T/A(M4)
S37N(D1)	D1M1	D1M2	D1M3	D1M4
R142K(D2)	D2M1	D2M2	D2M3	D2M4

**Fig. 3:**
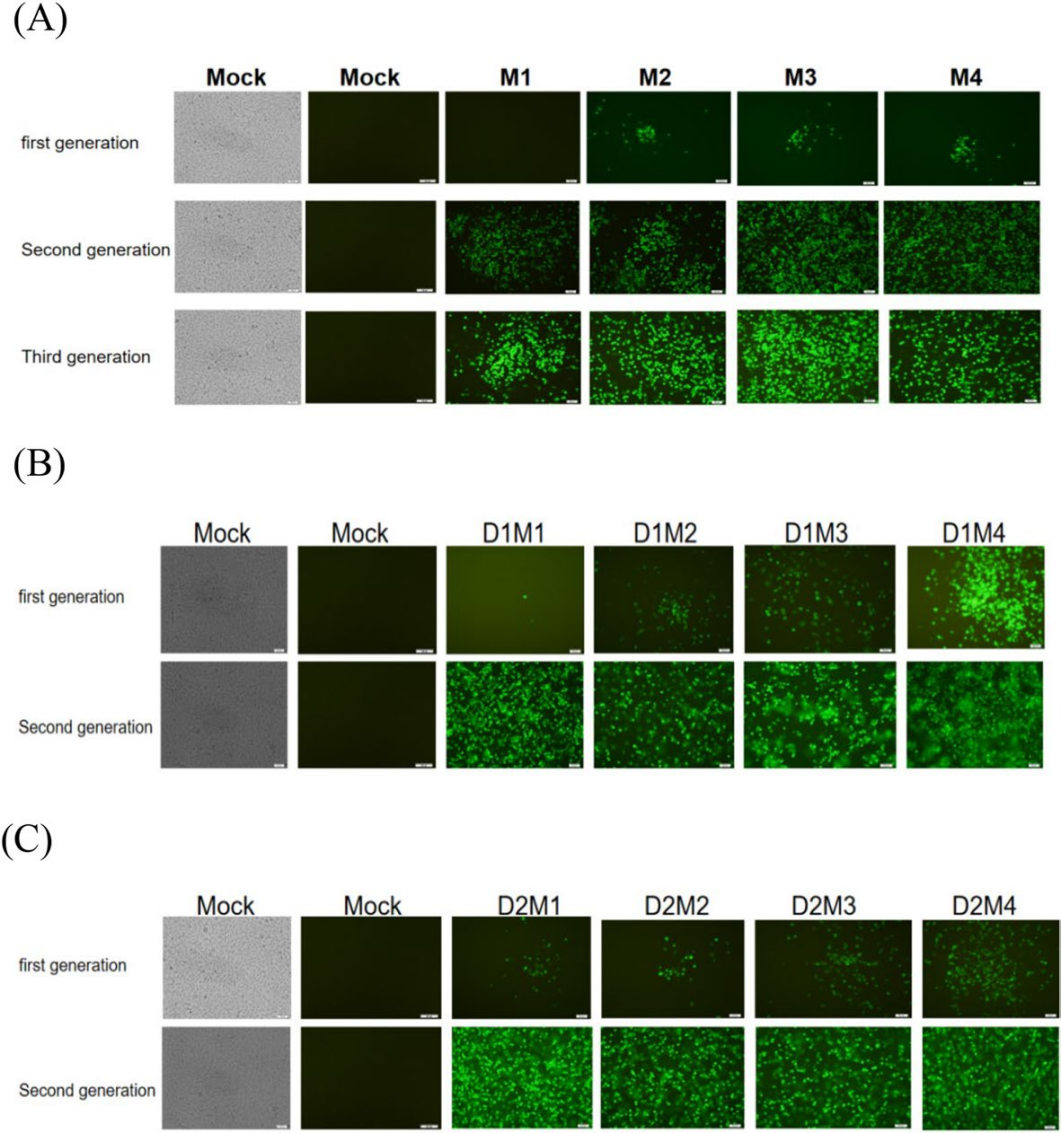
IFA identification of the mutant strains. **A** Four 5'UTR single-point mutant strains were transfected into RD cells, the first-generation fluorescence single was detected at 60h after transfection, and the second and third-generation fluorescence pictures were taken at 24h in the RD cells infected with viruses, while control cells were negative. **B** and **C** are fluorescence phenomena of 3D and 5'UTR co-mutant viruses. The fluorescence of the first generation appeared 72 hours later, while the fluorescence of the second generation appeared 24 hours later after the virus transfected cells

The schematic diagram of the construction of the mutation and co-mutation was shown in Fig. 2(B). The obvious cytopathic effect (CPE) and fluorescent phenomena were observed after the mutant RNA was transfected into RD cells. Subsequently, the mutated and co-mutated viruses were propagated by several passages in RD cells to improve the stability and titer of the virus (Fig. 3A-C). After measurement, the virus titers (CCID_50_) of strains were measured, as shown in Table 3. Then the mutant viruses were identified by sequencing to further confirm that the mutation site has successfully mutated as required.

**Table 3:** The titer of viruses^a^

Numbering	Virus	Titer(CCID_50_)
	SDLY1	10^−7.3^/0.1 ml
	SDLY107	10^−7.7^/0.1 ml
	EGFP-EV71	10^−7.2^/0.1 ml
D1	EGFP-EV71(S37N)	10^−6.8^/0.1 ml
D2	EGFP-EV71(R142K)	10^−6.3^/0.1 ml
M1	EGFP-EV71(nt88C/T)	10^−7.5^/0.1 ml
M2	EGFP-EV71(nt90-102–3 C)	10^−7.1^/0.1 ml
M3	EGFP-EV71(nt157G/A)	10^−7.8^/0.1 ml
M4	EGFP-EV71(nt574T/A)	10^−7.5^/0.1 ml
D1M1	EGFP-EV71(S37N-nt88C/T)	10^−7.4^/0.1 ml
D1M2	EGFP-EV71(S37N-nt90-102–3 C)	10^−8.5^/0.1 ml
D1M3	EGFP-EV71(S37N-nt157G/A)	10^−8.5^/0.1 ml
D1M4	EGFP-EV71(S37N-nt574T/A)	10^−8.5^/0.1 ml
D2M1	EGFP-EV71(R142K-nt88C/T)	10^−9.5^/0.1 ml
D2M2	EGFP-EV71(R142K-nt90-102-3)	10^−8.4^/0.1 ml
D2M3	EGFP-EV71(R142K-nt157G/A)	10^−9.0^/0.1 ml
D2M4	EGFP-EV71(R142K-nt574T/A)	10^−8.5^/0.1 ml

^a^The above data is only obtained during the construction of mutant strains, not the subsequent working concentration. To ensure the same MOI, the aforementioned viruses have been diluted to the same working concentration in subsequent experiments

### Mutant strains biological characteristics

#### The 5′UTR single-point mutant strains reduced virus replication

**Fig. 4:**
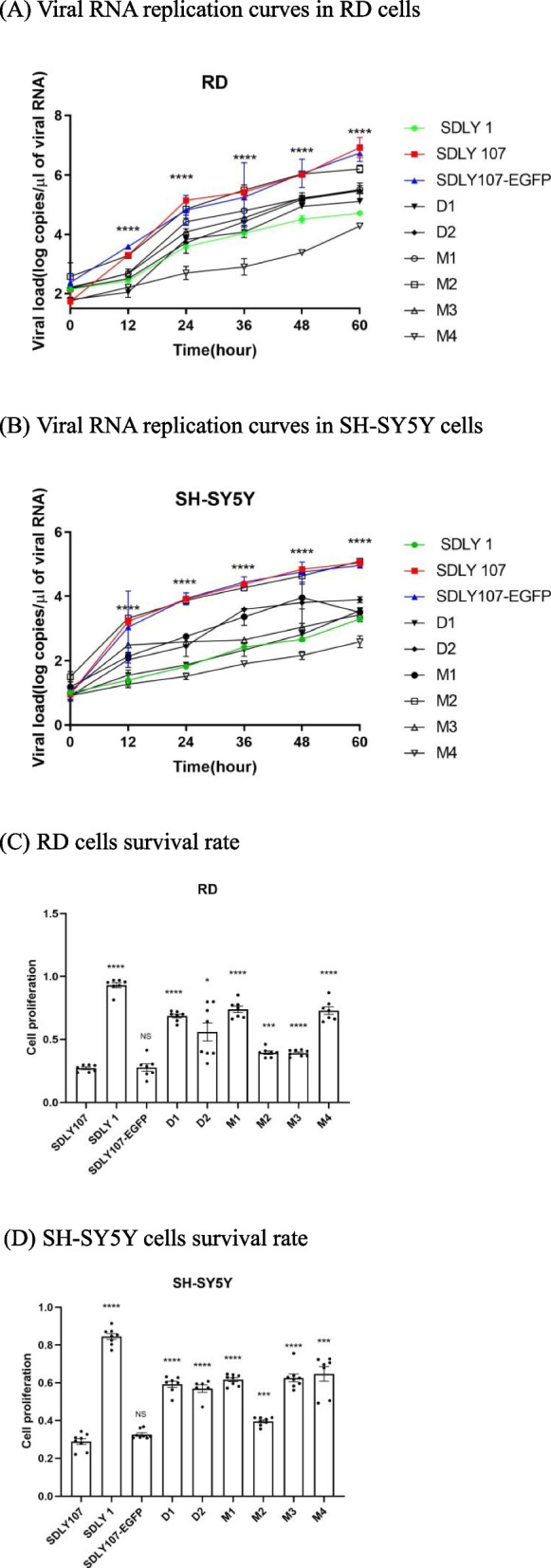
Viral RNA replication curves and cell survival rate of the mutant viruses. RD, and SH-SY5Y cells were infected with SDLY1, SDLY107, SDLY107-EGFP, D1, D2, M1, M2, M3 and M4 respectively at MOI=1PFU/cell. Viral loads in the supermatants of infected cell cultures were detected by RT-qPCR. The survival rate was reflected by cck-8. Results are expressed as the mean±SD. *p<0.05, **p<0.01, ***p<0.001, ****p<0.0001 compared with the virulent strain SDLY107. The straight line represents the comparison of two corresponding strains

Four 5′UTR mutant strains replication curves in RD cells and SH-SY5Y cells were analyzed by detecting the total viral RNA (Fig. 4). Except M2, the replication of two 3D mutant strains and the other three 5′UTR mutant strains was lower than that of the virulent strain. The replication of the EGFP-EV71 strain with the fluorescence gene was similar to that of the virulent strain. The survival level of cells infected with high replicative strains was significantly lower than that of the parent virulent strain by CCK-8. The result showed that the mutation affected the replication.

#### The co-mutant viruses had different effects on virus replication

The 5′UTR and 3D co-mutant strains replication curves were analyzed by detecting the total viral RNA (Fig. 4). Three different situations were detected:

##### The replication of the co-mutant viruses was between two single-point mutant strains or similar to one of them.

**Fig. 5:**
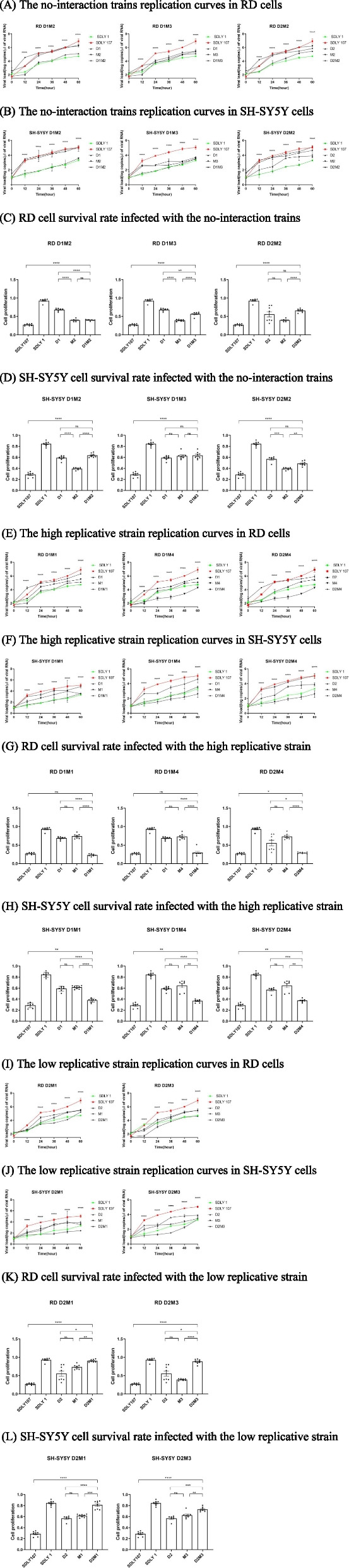
Viral RNA replication curves and cell survival rate of the co-mutant viruses. RD, and SH-SY5Y cells were infected with SDLY1, SDLY107, the co-mutant strains and related single mutant strains respectively at MOI=1PFU/cell. Viral loads in the supermatants of infected cell cultures were detected at 12h, 24h, 36h, 48h and 60h by RT-qPCR. The survival rate was reflected at 24h by cck-8. Results are expressed as the mean±SD. *p<0.05, **p<0.01, ***p<0.001, ****p<0.0001 compared with the virulent strain SDLY107. The straight line represents the comparison of two corresponding strains

It was found that the replication of D1M2 strain was similar to that of the M2; The replication of D1M3 was similar to that of D1 and M3; The replication of the D2M2 strain is between D2 and M2 (Fig. 5A, B). At the same time, the cell survival rate infected with D1M2, D1M3, and D2M2 strains was consistent with the level of replication (Fig. 5C, D). It was proved that D1M2, D1M3, and D2M2 mutation sites would weaken the virus replication ability. However, this result could not provide evidence for the interaction between 3D and 5′UTR.

##### The replication of the co-mutant viruses was higher than that of the two single-point mutant virus

The replication of D1M1, D1M4, and D2M4 co-mutant strains was higher than that of the two single-point mutant viruses (Fig. 5E, F). The survival rate of cells infected with the co-mutant strains D1M1, D1M4, and D2M4 was lower than that of the single-point mutant viruses (Fig. 5G, H). It showed that the three co-mutations greatly improved the virus replication, which indicated that EV71 5′UTR interacted with 3D at these sites.

##### The replication of the co-mutant viruses was lower than that of the two single-point mutations

The replication of D2M1 and D2M3 were lower than that of the two single-point mutation viruses (Fig. 5I, J). The cell survival rate of cells infected with the co-mutant strains D2M1 and D2M3 was higher than that of single-point mutant strains (Fig. 5K, L). These results proved that 3D can interact with 5′UTR, which could inhibit virus replication and weak the damage of virus to cells.

### Autophagy regulated by mutant and co-mutant viruses

**Fig. 6:**
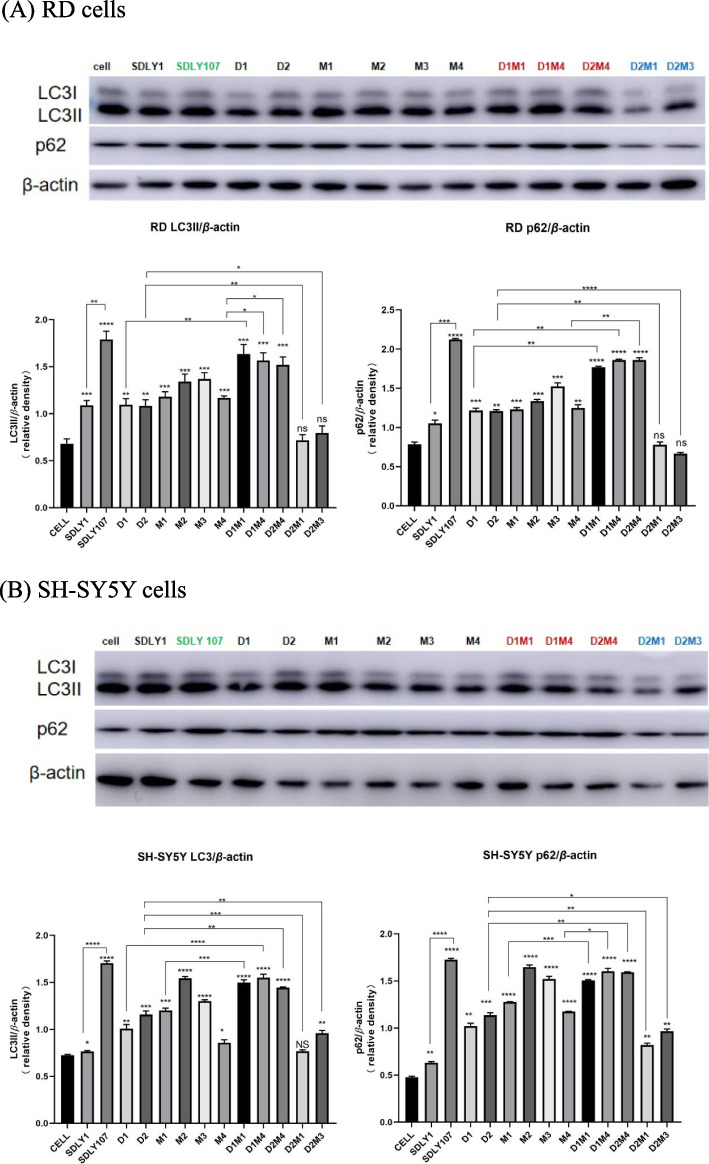
Western blot assay for the autophagy protein. D1M1, D1M4 and D2M4 (red) were the high replicative strains; D2M1 and D2M3 (blue mark) were the low replicative strains. RD, and SH-SY5Y cells were infected with SDLY1, SDLY107, 3D mutant strains, 5'UTR mutant strains and the co-mutant strains with interreaction respectively at MOI=1PFU/cell. Cell lysates were collected at 12h. Results are expressed as the mean±SD. *p<0.05, **p<0.01, ***p<0.001, ****p<0.0001 compared with the cell without virus. The straight line represents the comparison of two corresponding strains

Autophagy was quantitatively analyzed to explore the the mechanism by which the mutation and co-mutation affects the EV71 replication. The attenuated strain, the virulent strain, two 3D mutant viruses, four 5′UTR mutant viruses, and five co-mutant viruses (D1M1, D1M4h, D2M4, D2M1, and D2M3) which preliminarily proved that 5′UTR interacted with 3D, were inoculated into RD cells and SH-SY5Y cells respectively. Cell lysates were collected 12 h later. Uninfected cells served as the sham control. p62 and LC3 were detected by Western blotting. As shown in Fig. 6, the content of LC3 and p62 in cells infected with the high replicative strains (red) was significantly higher than that of the low replicative strains (blue). It indicated that the high replicative strains inhibited the autophagic flux in cells and promoted the accumulation of autophagy.

### AKT-mTOR is the key signaling pathway affecting EV71 replication

**Fig. 7:**
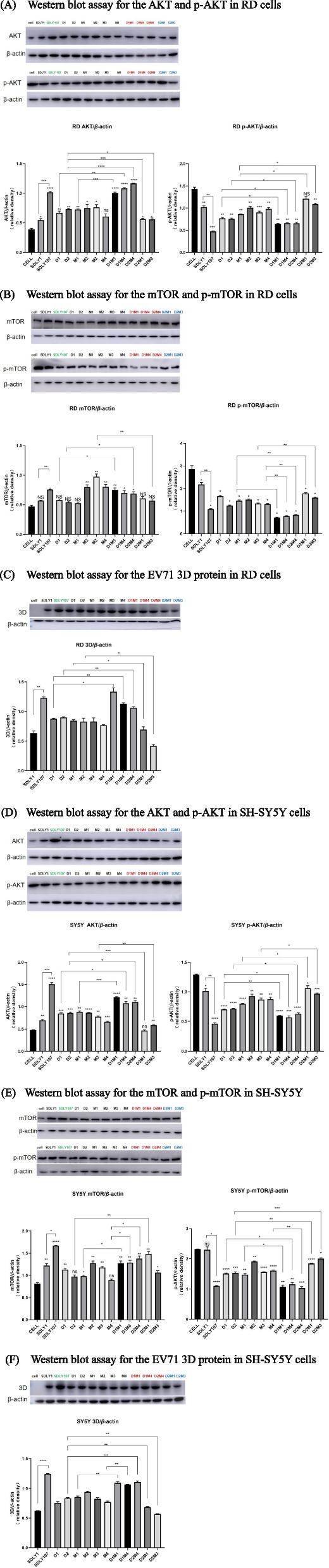
Western blot assay for the AKT-mTOR signaling pathway and EV71 3D protein. D1M1, D1M4 and D2M4 (red) were high replicative strains; D2M1 and D2M3 (blue) were low replicative strains. RD, and SH-SY5Y cells were infected with SDLY1, SDLY107, 3D mutant strains, 5'UTR mutant strains and the co-mutant strains with interreaction respectively at MOI=1PFU/cell. Cell lysates were collected at 12h. Results are expressed as the mean±SD. *p<0.05, **p<0.01, ***p<0.001, ****p<0.0001 compared with the cell without virus. The straight line represents the comparison of two corresponding strains

Research has shown that mTOR is an important molecule in the regulation of autophagy by EV71 [24]. The attenuated strain, the virulent strain, two 3D mutant strains, four 5′UTR mutant strains, and five co-mutant strains (D1M1, D1M4, D2M4, D2M1 and D2M3) were inoculated into RD cells and SH-SY5Y cells respectively. The cell lysate was collected 12 h later to detect AKT, p-AKT, mTOR, p-mTOR and EV71 3D proteins. As shown in Fig. 7, the p-AKT and p-mTOR contents of high replicative strains were significantly lower than those of the low replicative strains. The 3D protein content of high replicative strains was higher than that of low replicative strains. It indicated that the interaction between 5′UTR and 3D can regulate the effect of EV71 on autophagy.

**Fig. 8:**
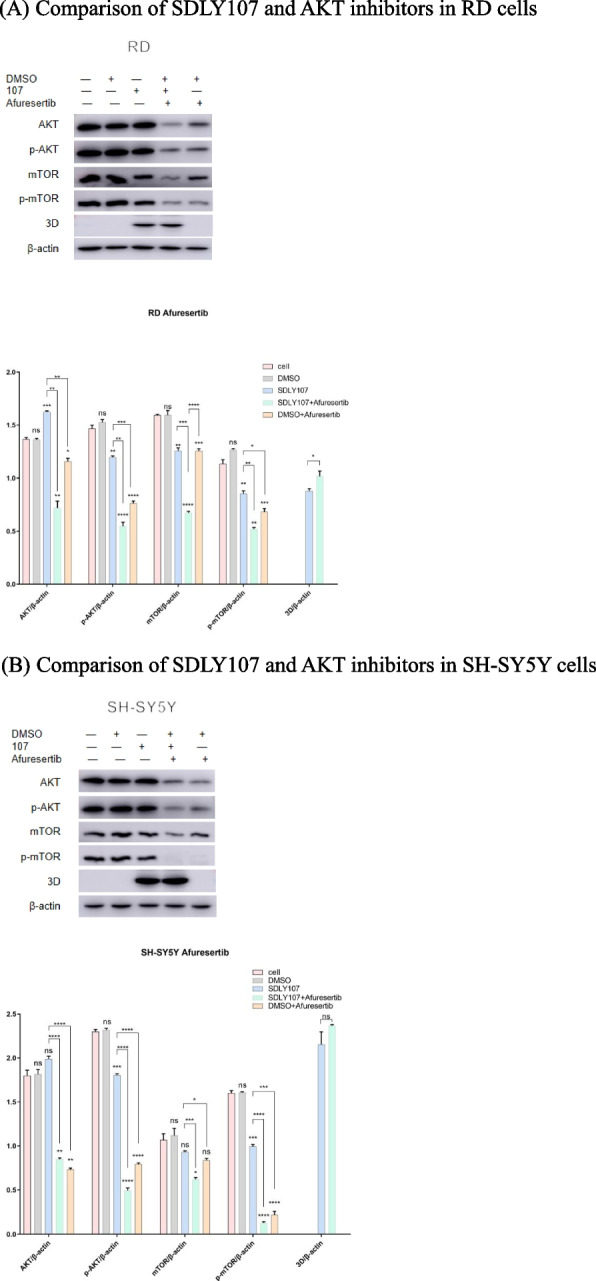
Western blot assay for the AKT-mTOR signaling pathway and EV71 3D protein in cells with Afuresertib. *p < 0.05, **p < 0.01, ***p < 0.001, ****p < 0.0001. MOI = 1PFU/cell. Cell lysates were collected at 12 h. Results are expressed as the mean ± SD. *p < 0.05, **p < 0.01, ***p < 0.001, ****p < 0.0001 compared with the cell without virus. The straight line represents the comparison of two corresponding strains

**Fig. 9:**
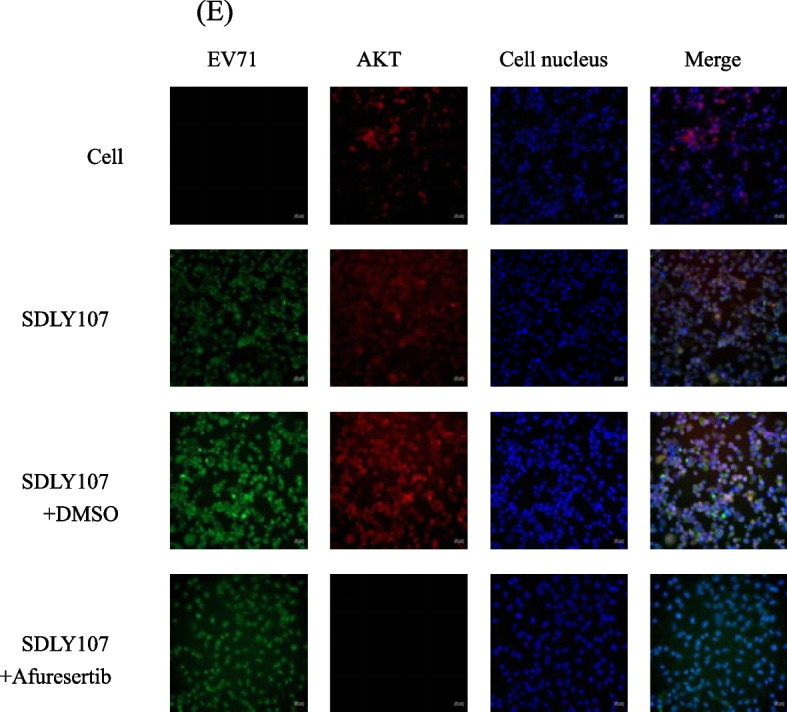
EV71 and AKT were detected by co-localization immunofluorescence at 12h after infection. FICT stained EV71 green, TRITC stained AKT red, and DAPI stained nucleus blue

 Afuresertib is an AKT inhibitor, which can inhibit the phosphorylation of AKT protein [26]. In this study, cell lysates infected with strains were collected at 12 h (Fig. 8A, B), after RD cells and SH-SY5Y cells were treated with Afuresertib 1mM. The effect of Afuresertib on reducing p-AKT mTOR and p-mTOR protein is more significant than the virulent strain, which showed that EV71 and Afuresertib had similar effects. It showed that the virus and AKT protein could co-locate (Fig. 9). The results proved that EV71 regulates autophagy through AKT-mTOR signal pathway.

## Discussion

EV71 is one of the pathogens of hand, foot and mouth disease, which can cause nervous system complications. In recent years, HFMD has broken out frequently in many countries and regions, posing a huge threat to public health security [27–30]. Most studies only focus on the structure and function of a single functional region of EV71, resulting in little attention to the interaction between multiple domains in the viral genome. Based on the effect of 5′UTR on virus replication, the interaction mechanism between 5′UTR and 3D was deeply discussed, which provided ideas for the development of anti-EV71 drugs.

The EV71 5′UTR has a complex spatial structure, including a cloverleaf structure and an internal ribosome entry site (IRES), which co-associated with viral RNA replication. Research showed that when the activity of 5′UTR was inhibited by the host restriction factor APOBEC3G (A3G), the role of the host protein poly (C)-binding protein 1 (PCBP1), which is necessary for the synthesis of EV71 virus protein and RNA, was inhibited, thus reducing the virulence of EV71 [31]. Sabin3-like mutation (U473 to C) introduced in CVB3 IRES genome led to a defective mutant with a serious reduction in translation efficiency, which effected the binding affinity of IRES to some standard translation initiation factors: eIF4G, eIF3b, and eIF4B [32]. In addition, in mice, the mutant strain in the domain V of the IRES RNA of CVB3 display different levels of decreased replication and translation initiation efficiency [33].

Research showed that Poly C binding protein played a role in promoting the transition of virus from protein translation to viral RNA synthesis by regulating of 5′UTR clover leaf structure [34]. In this study, the mutant nt88C/T in the clover leaf structure was selected to study the effect on clover leaf structure. When cytosine mutates into uracil in SLII, the replication and virulence of the virus had changed significantly. There was also a site mutation (nt157 G/A) in this region of the virus used for comparison. There is an interval between the clover leaf and SL II in 5′UTR. Study has shown that interval has an important impact on the neurotoxicity and temperature sensitivity of poliovirus [35]. In this study, it found that the virulent strain lacks 3 cytosines at this interval than the attenuated strain. In addition, EV71 virus isolated from patients with different clinical manifestations has base mutation at the SL VI structure of 5′UTR [36]. The virus strain used in this study also has a nt574T/A mutation at this region. To sum up, four mutation sites, namely nt88 C/T, nt90-102–3 C, nt157 G/A, nt574 T/A, were selected from the clover leaf structure、the interval between clover leaf、 SLII and SLVI successively. The mutation of nt88C/T, nt157G/A and nt574T/A reduced the replication ability and cell damage ability of EV71 in vitro experiments, indicating that the different structures of 5′UTR had an important impact on the replication of EV71.

3D protein, which is an RNA dependent RNA polymerase (RdRp), is responsible for regulating the extension of nucleic acid chains during virus replication. 3D protein crystal is a “right hand” conformation, including “finger”, “palm” and “thumb” domains, that different domains have different functions [37]. Studies have shown that there was potential interaction between 3D and 5′UTR. For example, EV71 can activate SIRT1, which can inhibit RNA replication by combining with 5′UTR clover leaf structure and can also weaken the translation ability of viral proteins by combining with IRES. In addition, SIRT1 can also combine with EV71 3D to inhibit 3D acetylation and polymerase activity [38]. In poliovirus, the interaction between 5′UTR and Poly (rC) binding protein (PCBP) was regulated by 3CD of the EV71, which could affect virus replication [9]. In this study, based on the construction of two 3D protein mutant viruses [39], eight 5′UTR and 3D co-mutant viruses were constructed to precisely locate the interaction sites. The results showed that three co-mutant strains EGFP-EV71 (S37N-nt88C/T), EGFP-EV71 (S37N-nt574T/A) and EGFP-EV71 (R142K-nt574T/A) significantly enhanced the replication ability and cell damage ability of the virus. The two co-mutant strains EGFP-EV71 (R142K-nt88C/T) and EGFP-EV71 (R142K-nt157G/A) significantly reduced the replication ability and cell damage ability of the virus. These results indicated that there was different interaction between different sites of 5′UTR and 3D. The crystal structure of EV71 showed that there is a cross-region between 3D protein and 5′UTR sequence [12]. It speculated that mutation may change their structures affecting their structural binding ability, which affected the viral replication ability and cell damage ability.

Autophagy is a metabolic pathway of eukaryotic cells, which plays an important role in maintaining cell homeostasis. In recent years, studies have found that some viruses promoted virus replication by manipulating autophagy, such as HIV, CVB3 and EV71 [40–42]. The replication of EV71 promoted the formation of autophagy and increased disease severity [18]. At the same time, study showed that during EV71 infection, the virus regulated incomplete autophagy by affecting mTOR signaling molecules, thereby promoting the virus replication. However, the signaling pathway is still unclear. Studies showed that EV71 infection induced the activation of PI3K/AKT pathway [24, 38]. AKT is a pathway molecule which activates many downstream molecules to affect autophagy. In this study, it found that EV71 and AKT could co-locate by immunofluorescence test. Cells infected EV71 inhibited the phosphorylation of AKT, which is slightly weaker but same effect than that treated with Afuresertib. Both of them inhibited the activity of mTOR, which promoted the synthesis of virus 3D protein. The results proved that EV71 regulated cell autophagy through AKT-mTOR pathway to promote virus replication.

## Conclusions

In summary, the characteristics of the interaction of 5′UTR and 3D were related to the different site. The co-mutation of S37N and nt88C/T, S37N and nt574T/A, R142K and nt574T/A induced incomplete autophagy by inhibiting the AKT-mTOR signaling pathway, which promoted virus replication. The co-mutation of R142K and nt88C/T, R142K and nt157G/A significantly reduced the inhibition of EV71 on AKT-mTOR pathway. This study promoted the understanding of the pathogenesis of EV71, which provided ideas for the development of clinical specific drugs.

### Supplementary Information

Supplementary Material 1.

## Data Availability

No datasets were generated or analysed during the current study.
