# Supplementary figures and images for: EV71 5’UTR interacts with 3D protein affecting replication through the AKT-mTOR pathway (part 2 of 2)
Source: Virol J. 2024 May 22;21:114. doi: 10.1186/s12985-024-02385-z (PMC11110317; doi:10.1186/s12985-024-02385-z)

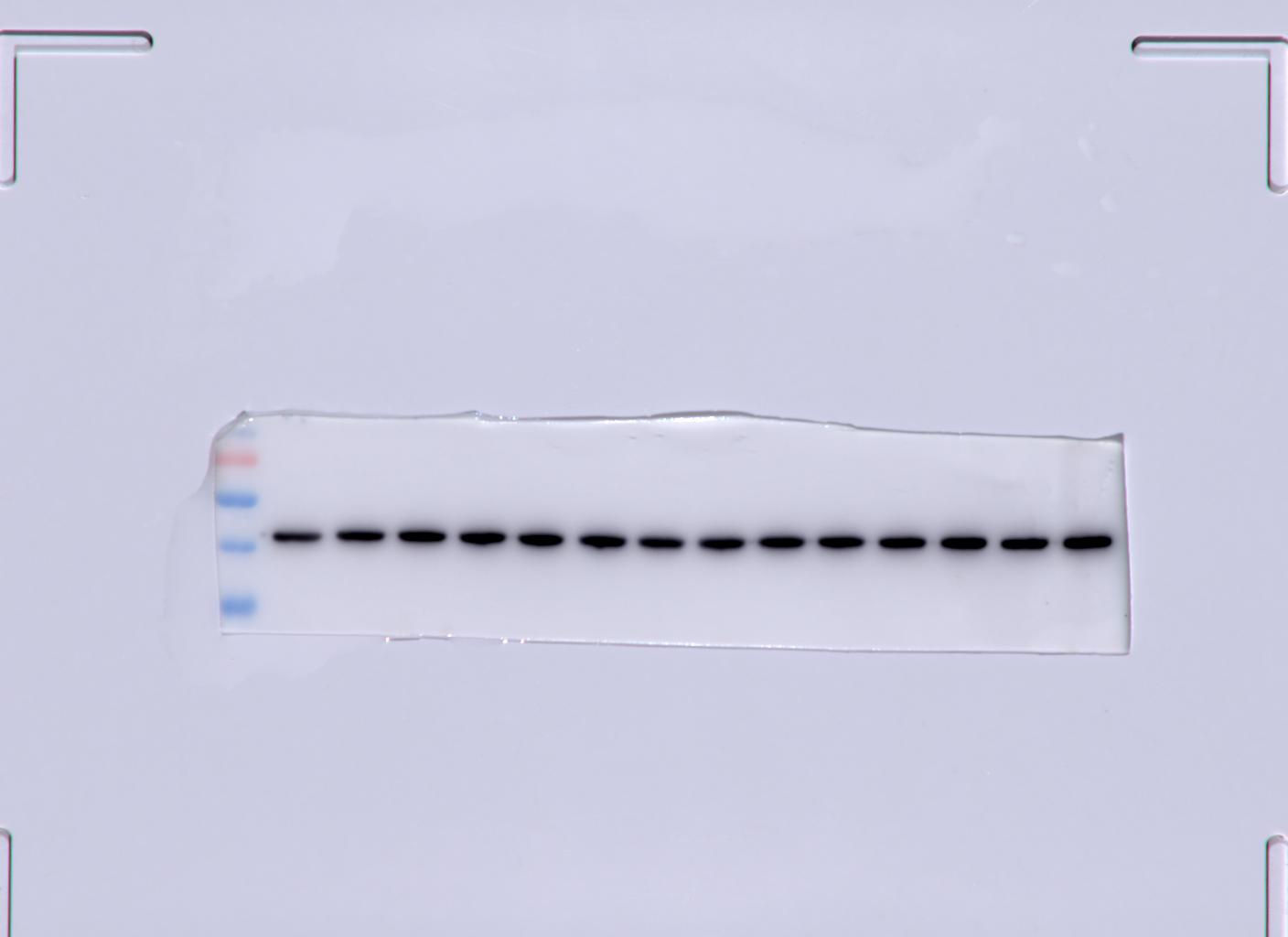

Supplement: Supplementary file 1 — Supplementary Material 1. [file 12985_2024_2385_MOESM1_ESM.zip › xuxiaoying WB/SY5YmTOR nc1 2021.09.16_18.04.11_Ch/nc1 2021.09.16_18.04.11_Ch+Marker.jpg]

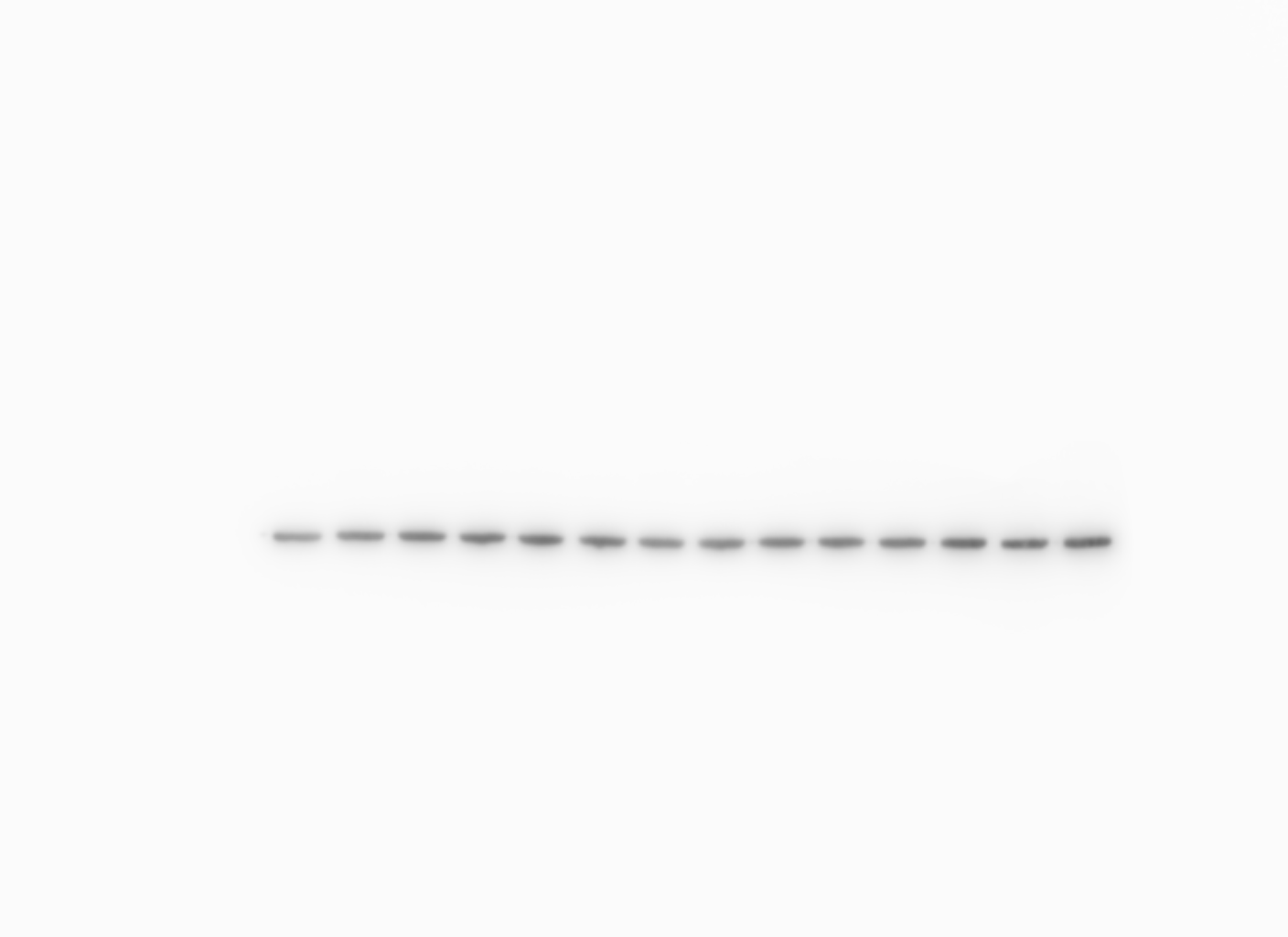

Supplement: Supplementary file 1 — Supplementary Material 1. [file 12985_2024_2385_MOESM1_ESM.zip › xuxiaoying WB/SY5YmTOR nc1 2021.09.16_18.04.11_Ch/nc1 2021.09.16_18.04.11_Ch.tif]

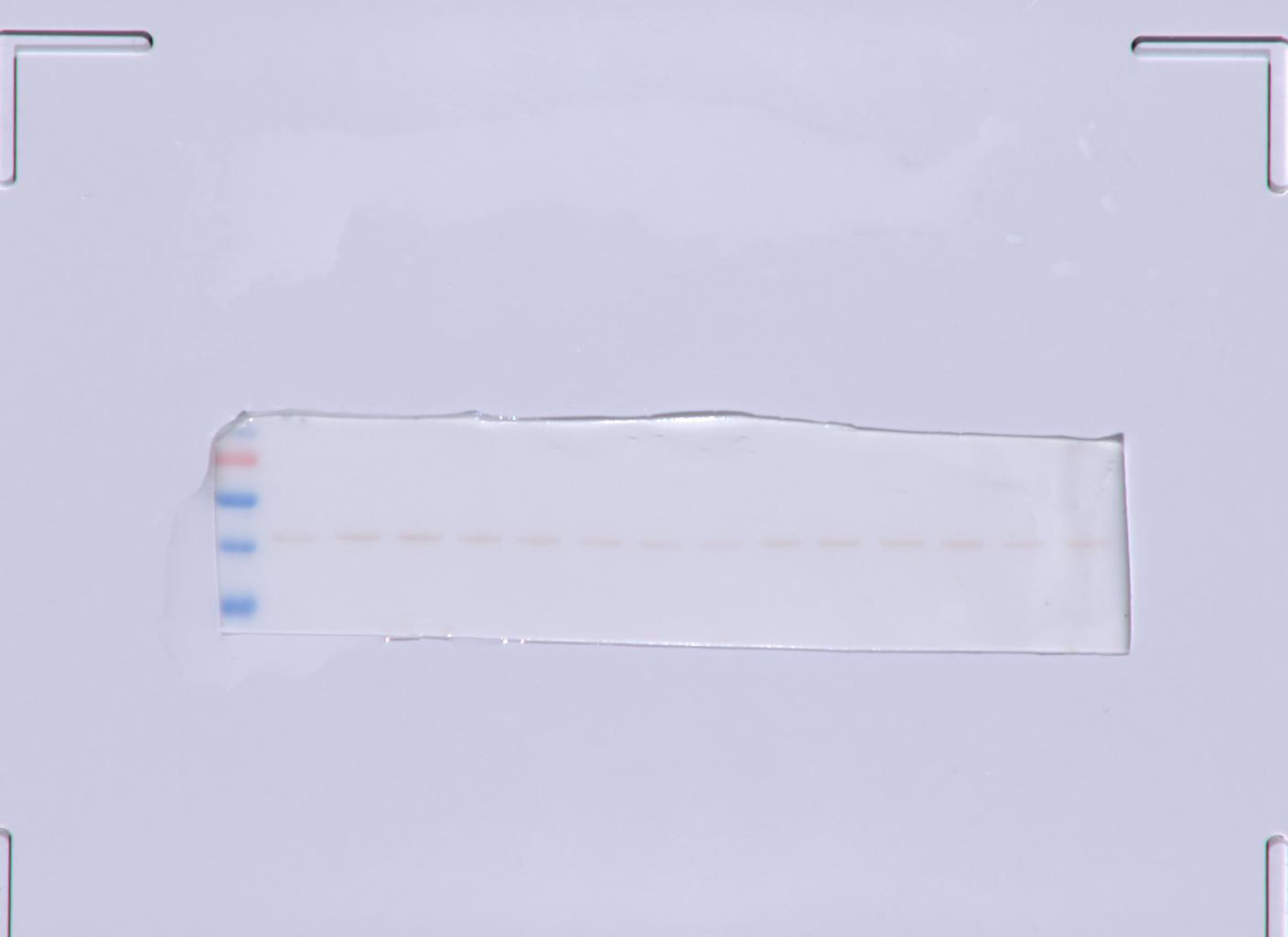

Supplement: Supplementary file 1 — Supplementary Material 1. [file 12985_2024_2385_MOESM1_ESM.zip › xuxiaoying WB/SY5YmTOR nc1 2021.09.16_18.04.11_Ch/nc1 2021.09.16_18.04.11_Ch-Marker.jpg]

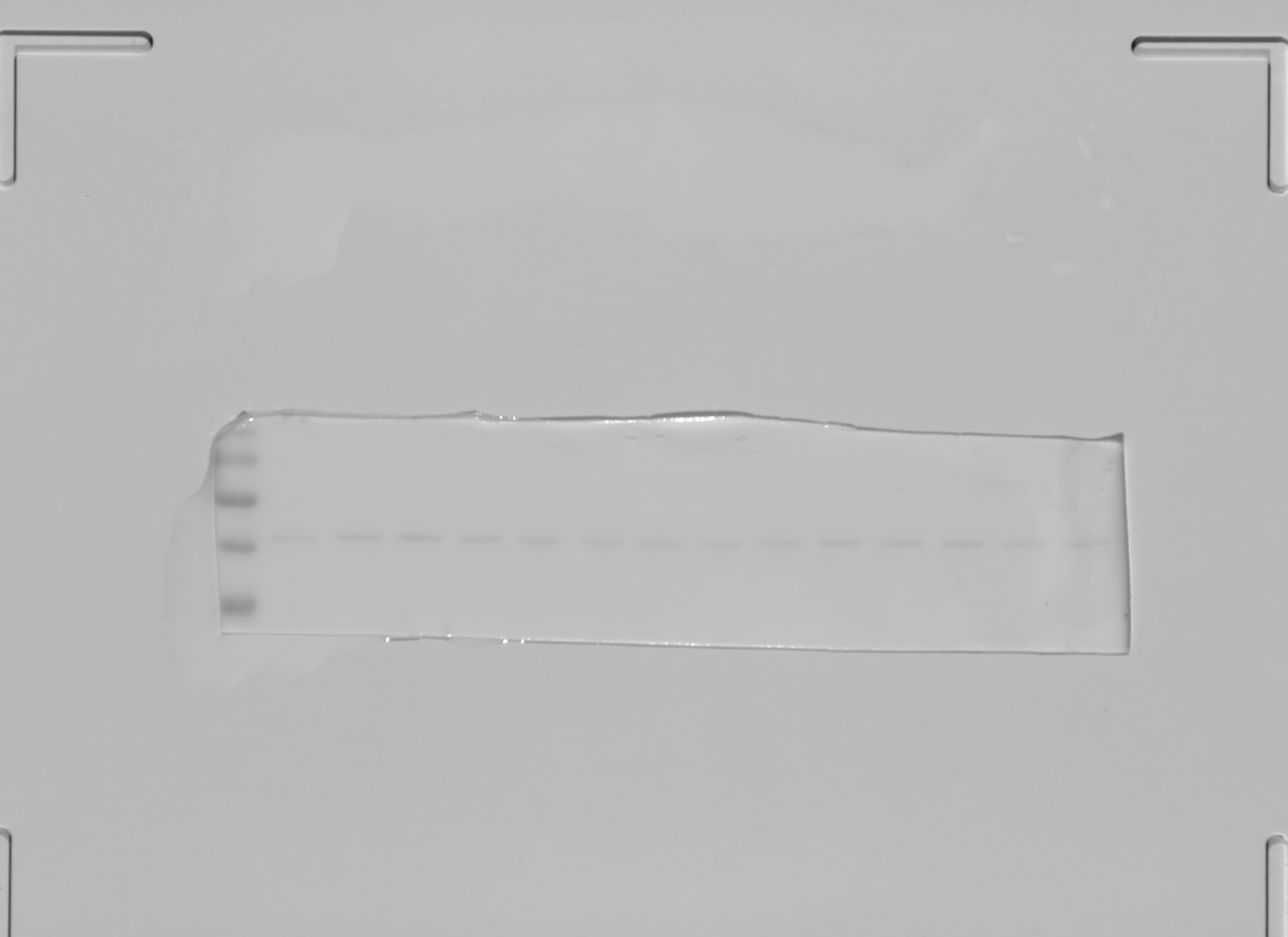

Supplement: Supplementary file 1 — Supplementary Material 1. [file 12985_2024_2385_MOESM1_ESM.zip › xuxiaoying WB/SY5YmTOR nc1 2021.09.16_18.04.11_Ch/nc1 2021.09.16_18.04.11_Ch-Marker.tif]
