# Supplementary figures and images for: EV71 5’UTR interacts with 3D protein affecting replication through the AKT-mTOR pathway (part 1 of 2)
Source: Virol J. 2024 May 22;21:114. doi: 10.1186/s12985-024-02385-z (PMC11110317; doi:10.1186/s12985-024-02385-z)

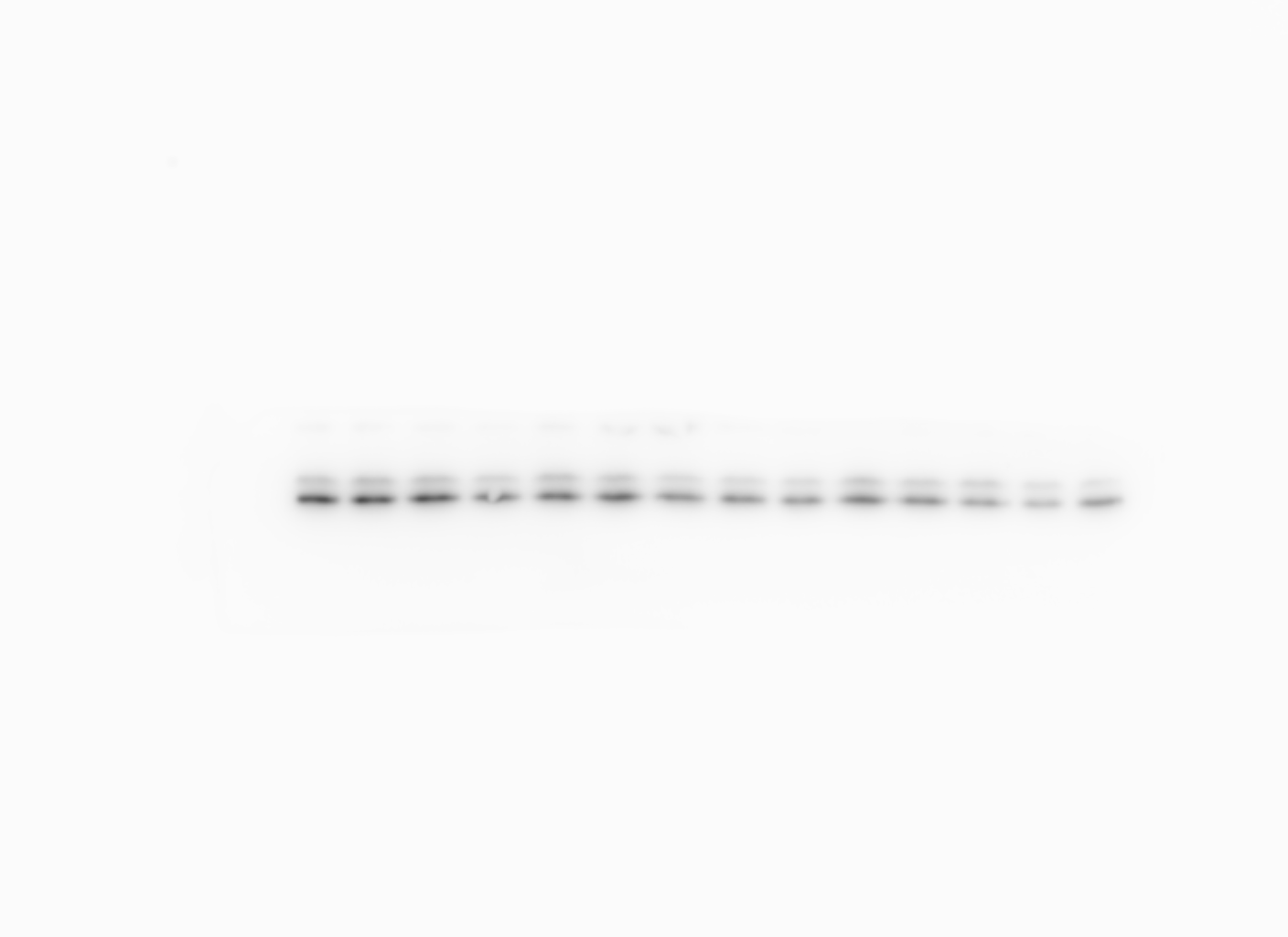

Supplement: Supplementary file 1 — Supplementary Material 1. [file 12985_2024_2385_MOESM1_ESM.zip › xuxiaoying WB/lc3 2021.09.10_14.53.15_Ch/lc3 2021.09.10_14.53.15_Ch.tif]

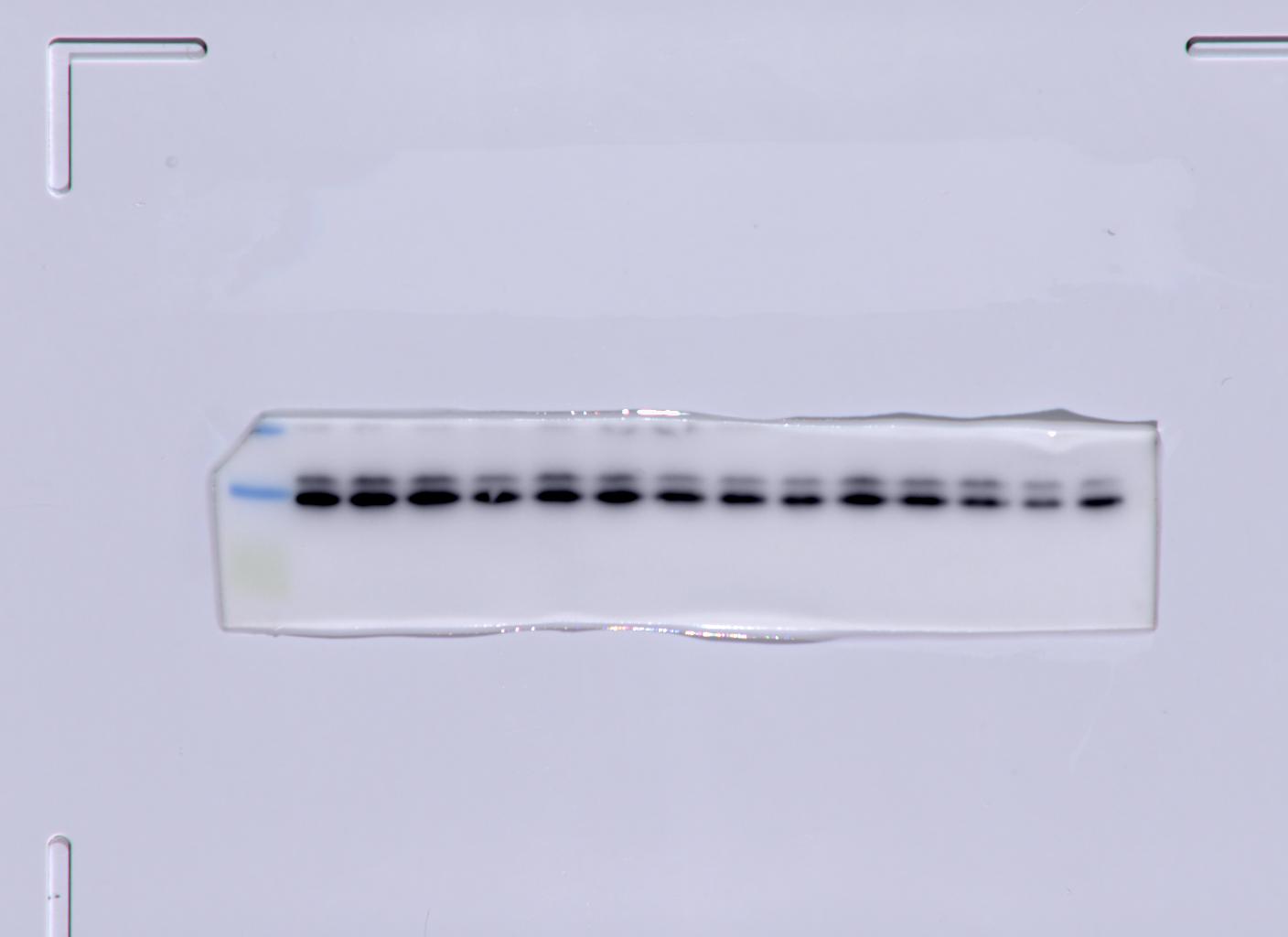

Supplement: Supplementary file 1 — Supplementary Material 1. [file 12985_2024_2385_MOESM1_ESM.zip › xuxiaoying WB/lc3 2021.09.10_14.53.15_Ch/lc3 2021.09.10_14.53.15_Ch+Marker.jpg]

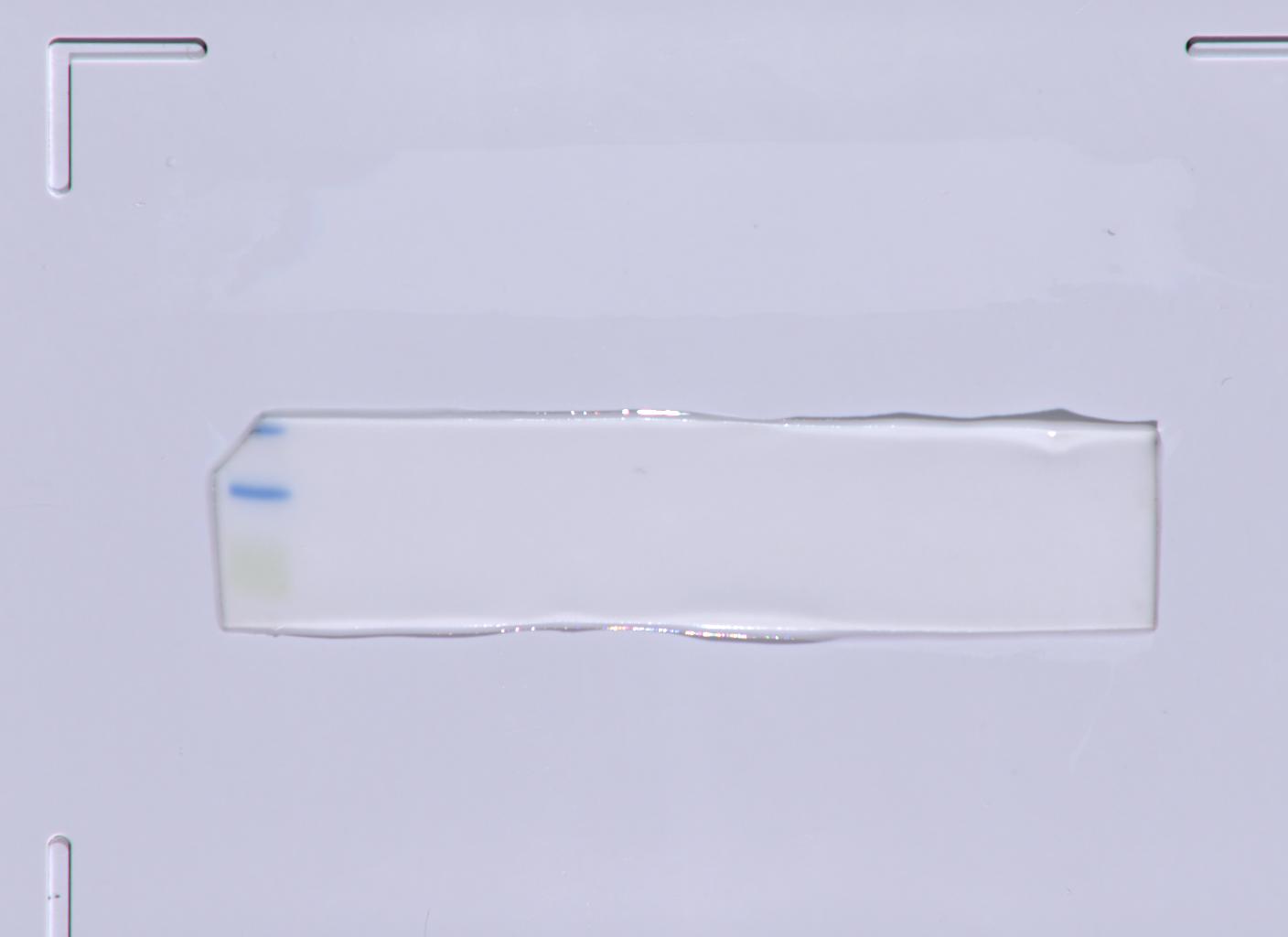

Supplement: Supplementary file 1 — Supplementary Material 1. [file 12985_2024_2385_MOESM1_ESM.zip › xuxiaoying WB/lc3 2021.09.10_14.53.15_Ch/lc3 2021.09.10_14.53.15_Ch-Marker.jpg]

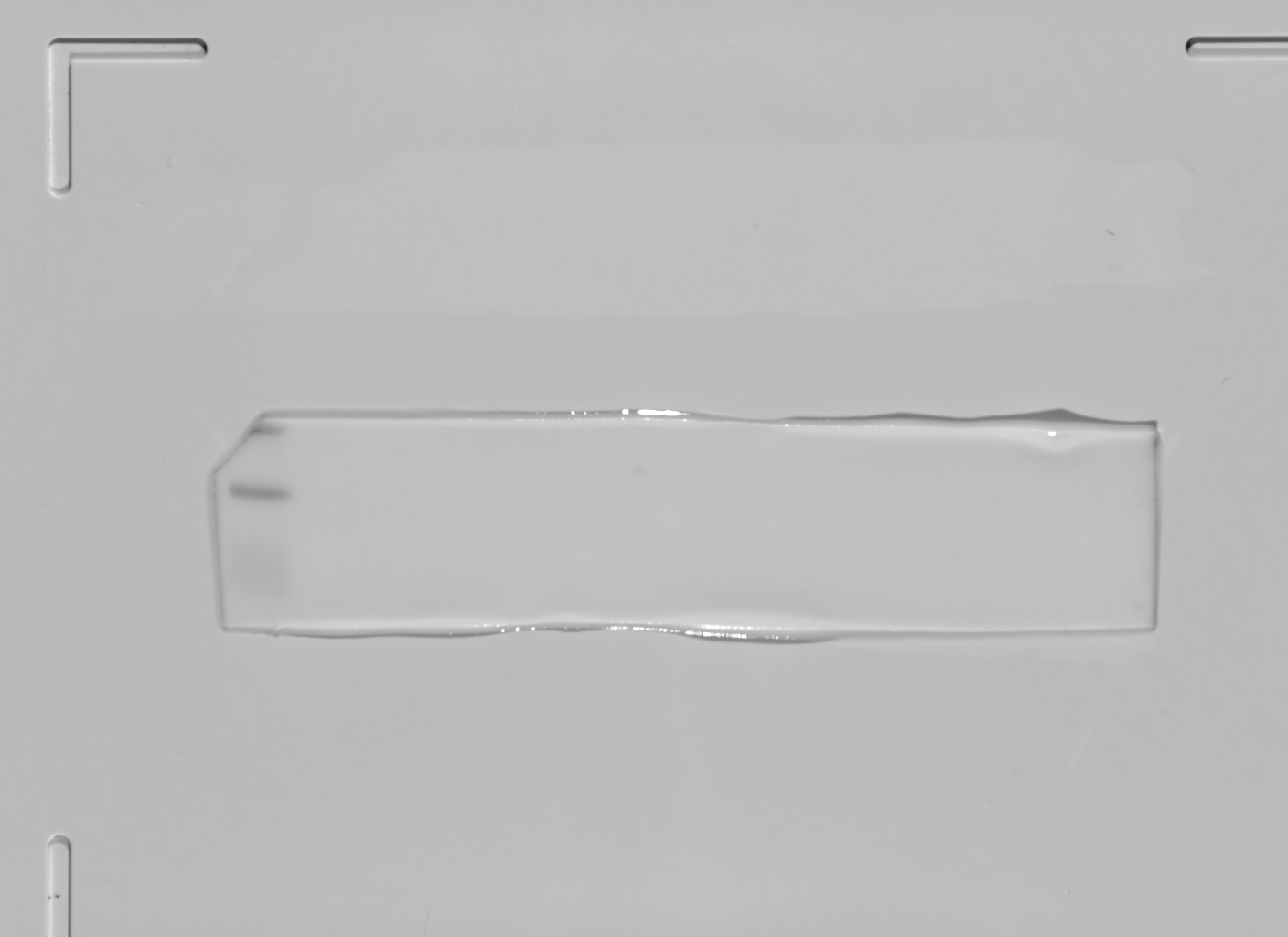

Supplement: Supplementary file 1 — Supplementary Material 1. [file 12985_2024_2385_MOESM1_ESM.zip › xuxiaoying WB/lc3 2021.09.10_14.53.15_Ch/lc3 2021.09.10_14.53.15_Ch-Marker.tif]

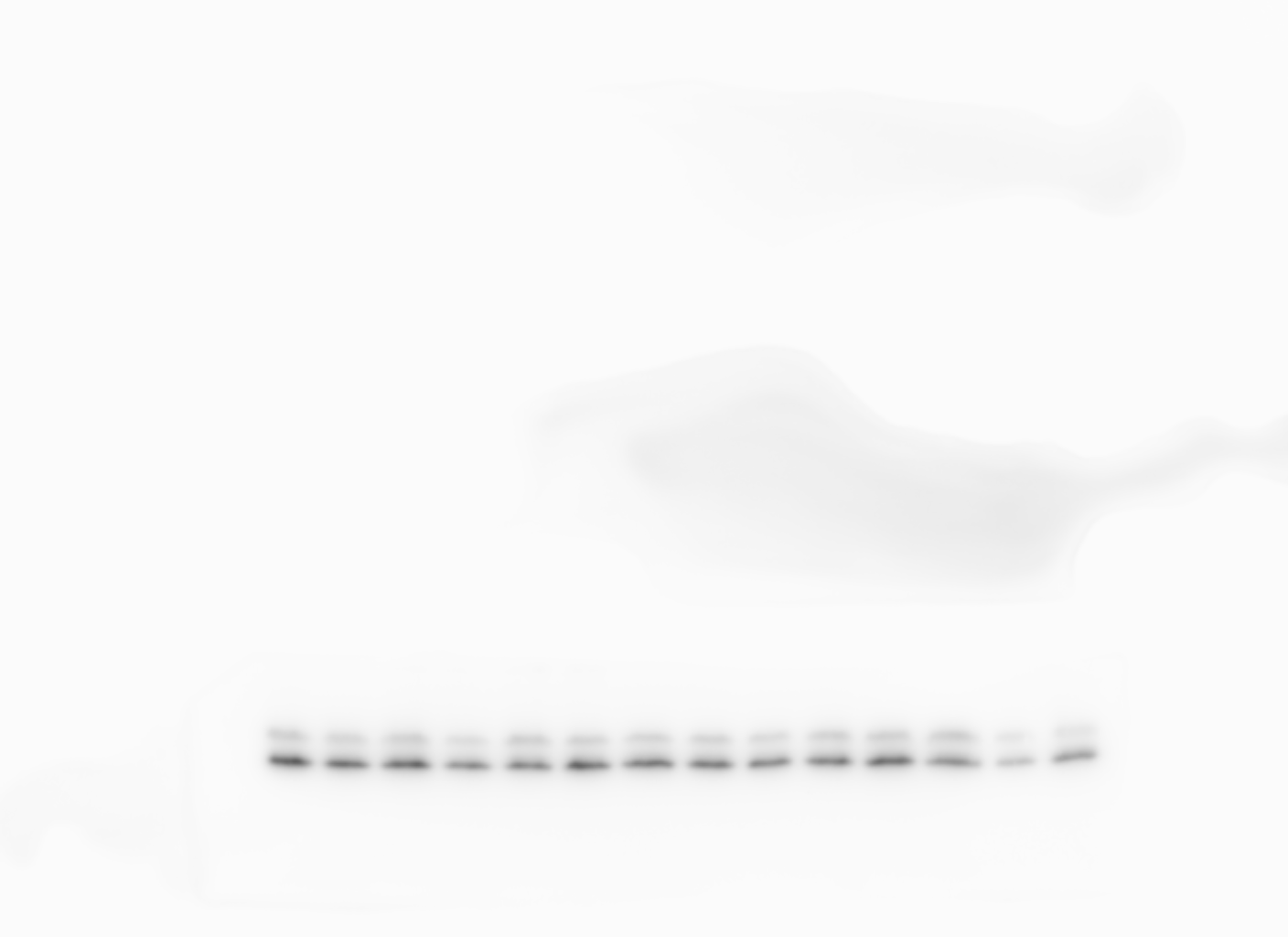

Supplement: Supplementary file 1 — Supplementary Material 1. [file 12985_2024_2385_MOESM1_ESM.zip › xuxiaoying WB/RD lc3I-II 2021.09.10_14.47.50_Ch/lc3 2021.09.10_14.47.50_Ch.tif]

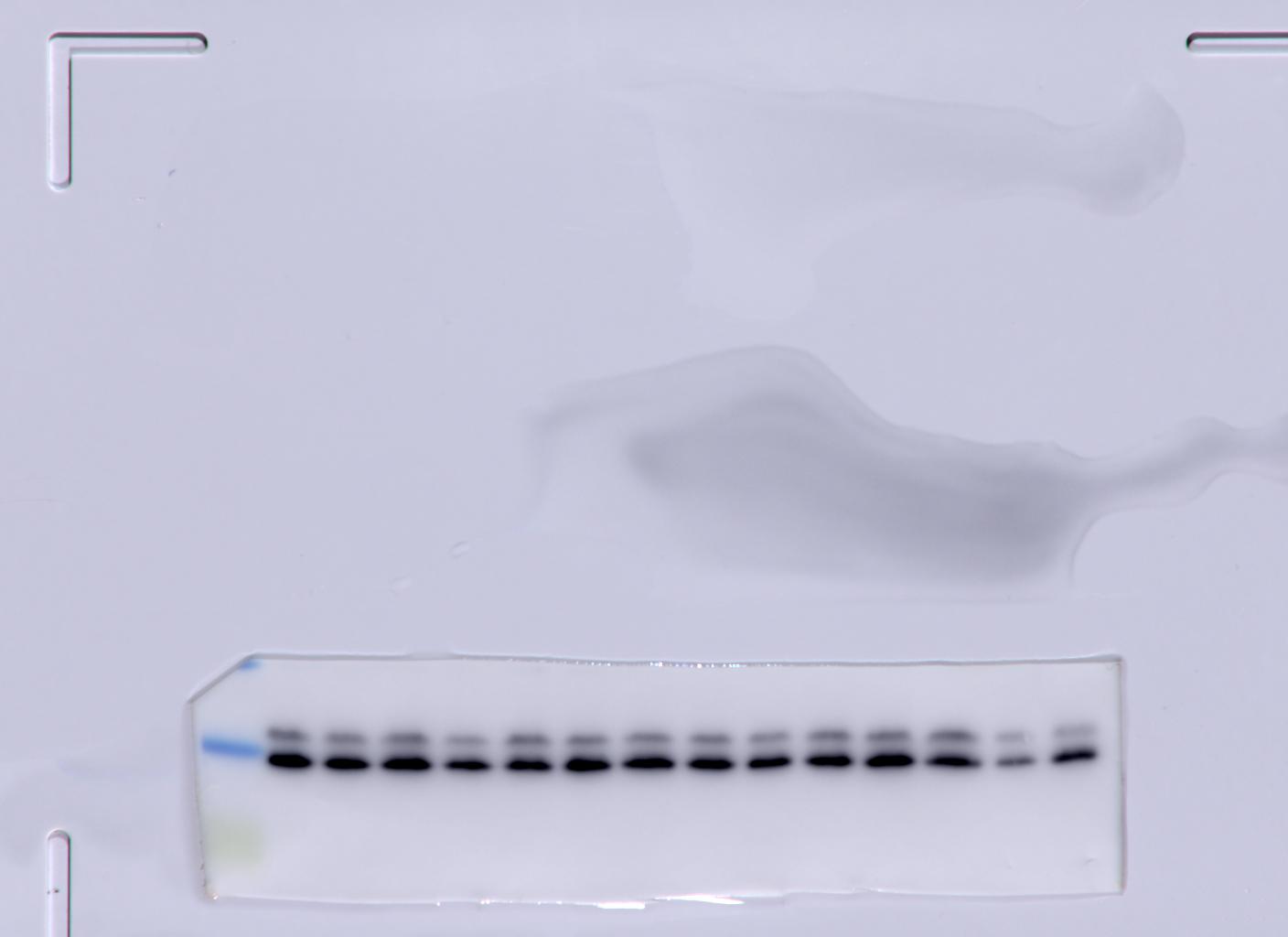

Supplement: Supplementary file 1 — Supplementary Material 1. [file 12985_2024_2385_MOESM1_ESM.zip › xuxiaoying WB/RD lc3I-II 2021.09.10_14.47.50_Ch/lc3 2021.09.10_14.47.50_Ch+Marker.jpg]

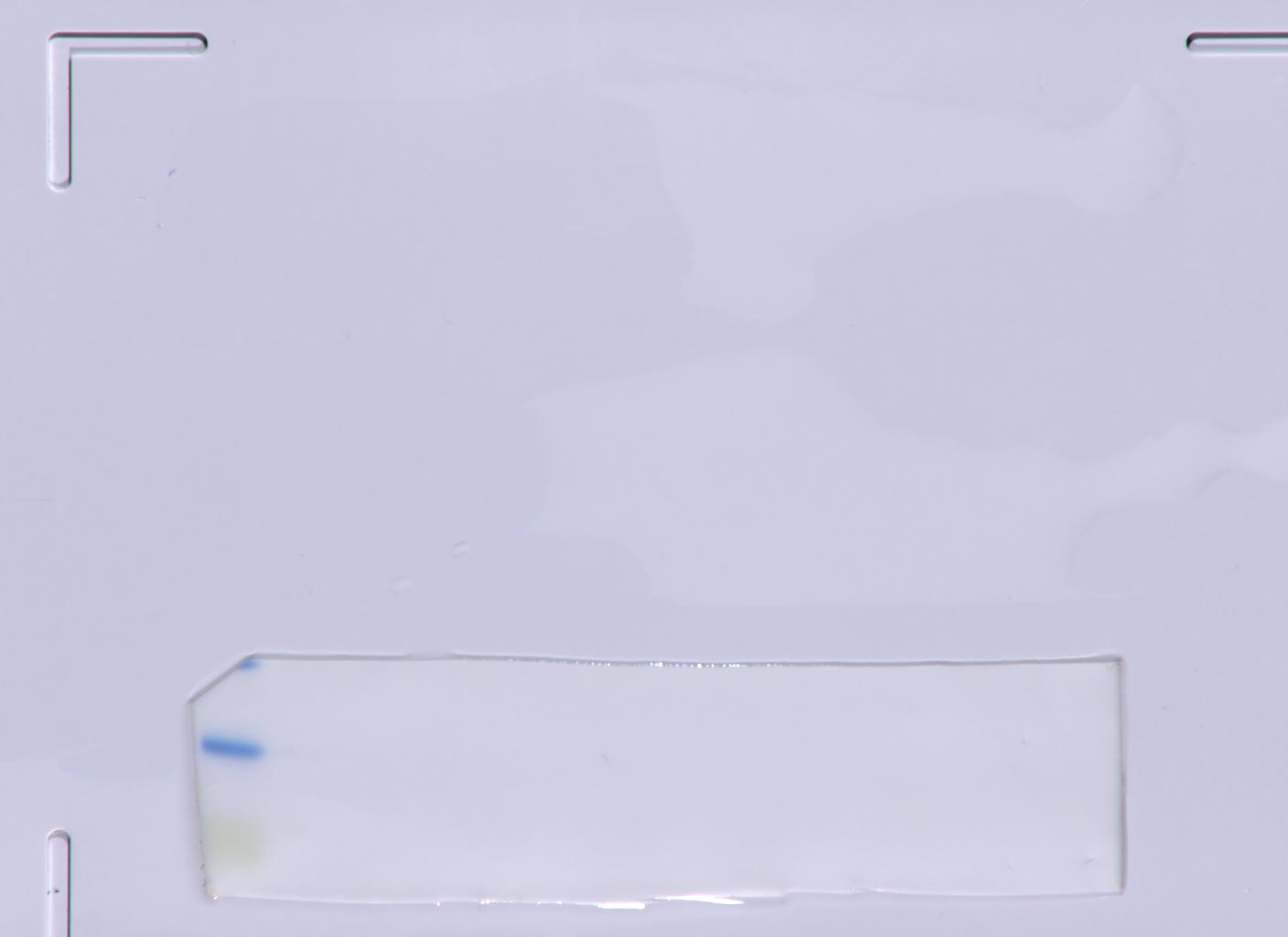

Supplement: Supplementary file 1 — Supplementary Material 1. [file 12985_2024_2385_MOESM1_ESM.zip › xuxiaoying WB/RD lc3I-II 2021.09.10_14.47.50_Ch/lc3 2021.09.10_14.47.50_Ch-Marker.jpg]

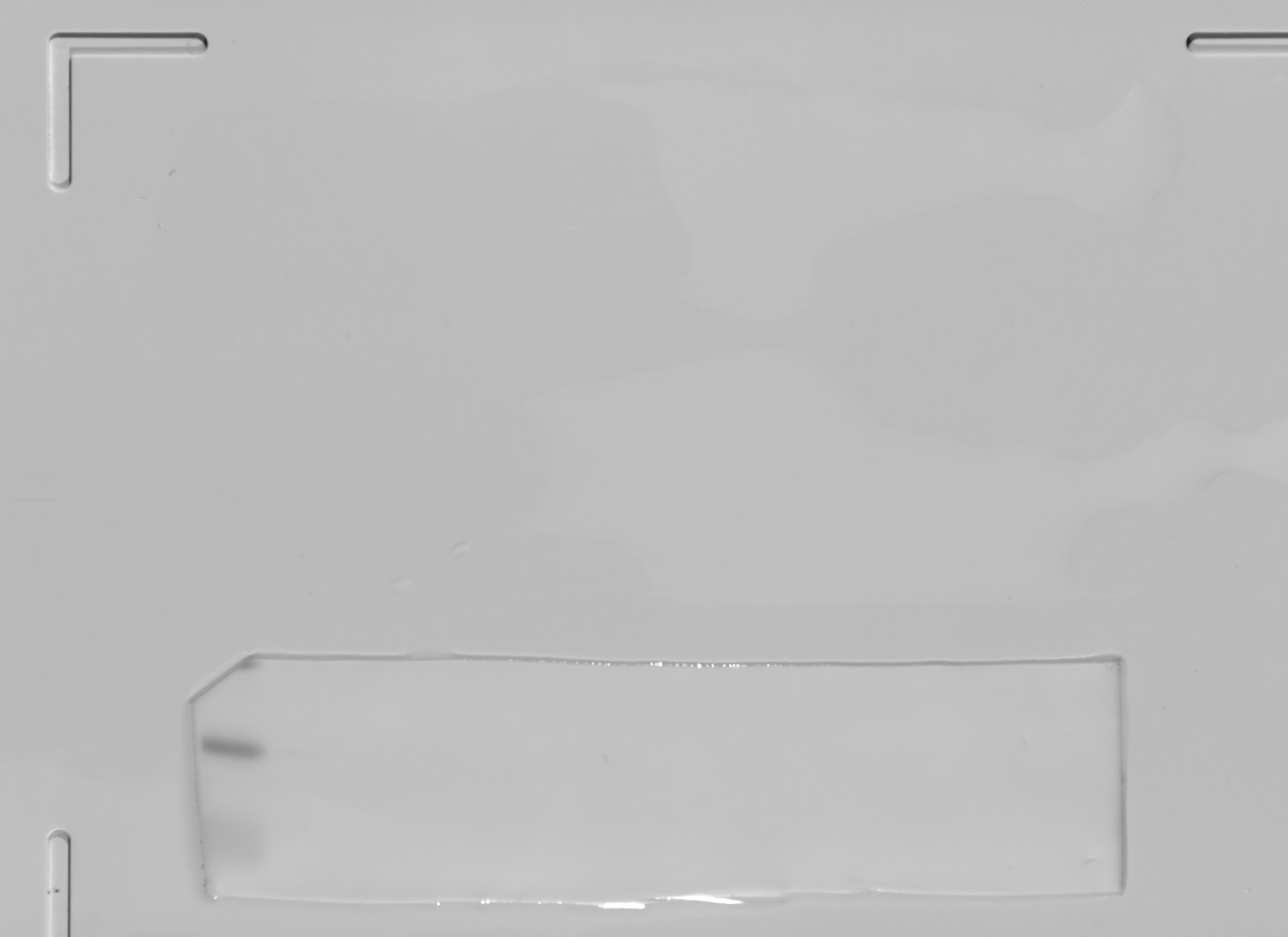

Supplement: Supplementary file 1 — Supplementary Material 1. [file 12985_2024_2385_MOESM1_ESM.zip › xuxiaoying WB/RD lc3I-II 2021.09.10_14.47.50_Ch/lc3 2021.09.10_14.47.50_Ch-Marker.tif]

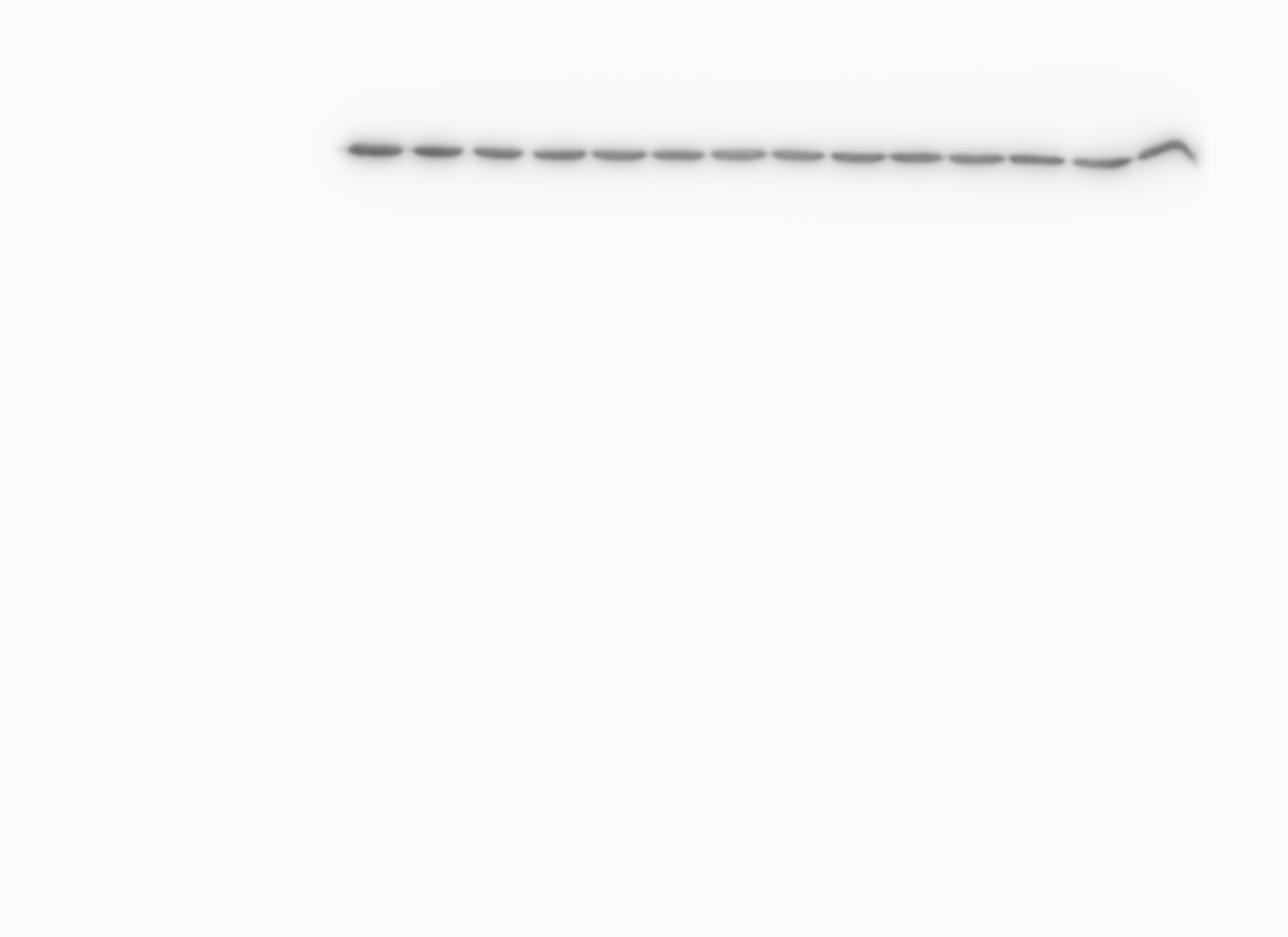

Supplement: Supplementary file 1 — Supplementary Material 1. [file 12985_2024_2385_MOESM1_ESM.zip › xuxiaoying WB/RD 3D b-actin 1 2021.09.14_16.38.13_Ch/b-actin 1 2021.09.14_16.38.13_Ch.tif]

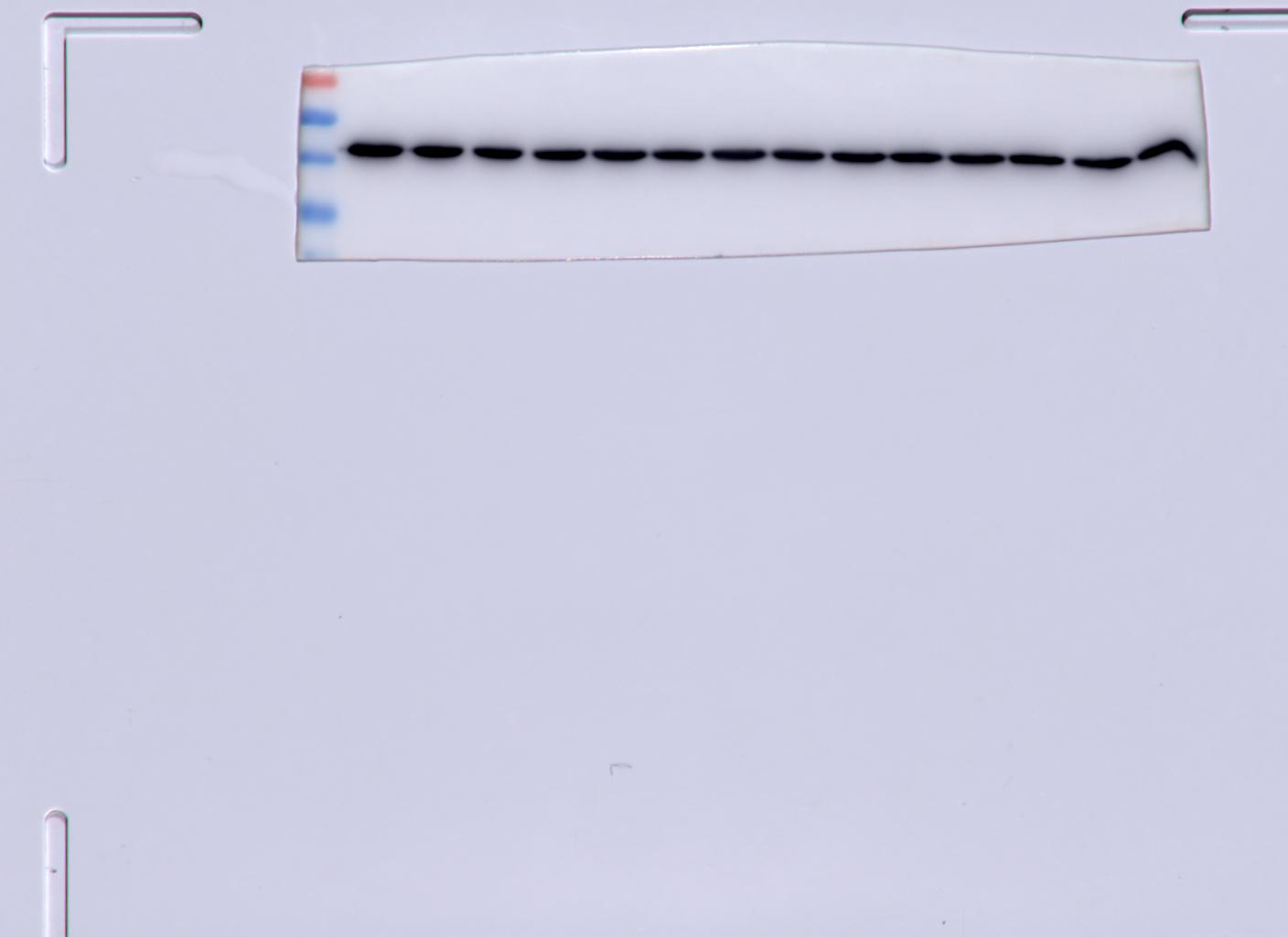

Supplement: Supplementary file 1 — Supplementary Material 1. [file 12985_2024_2385_MOESM1_ESM.zip › xuxiaoying WB/RD 3D b-actin 1 2021.09.14_16.38.13_Ch/b-actin 1 2021.09.14_16.38.13_Ch+Marker.jpg]

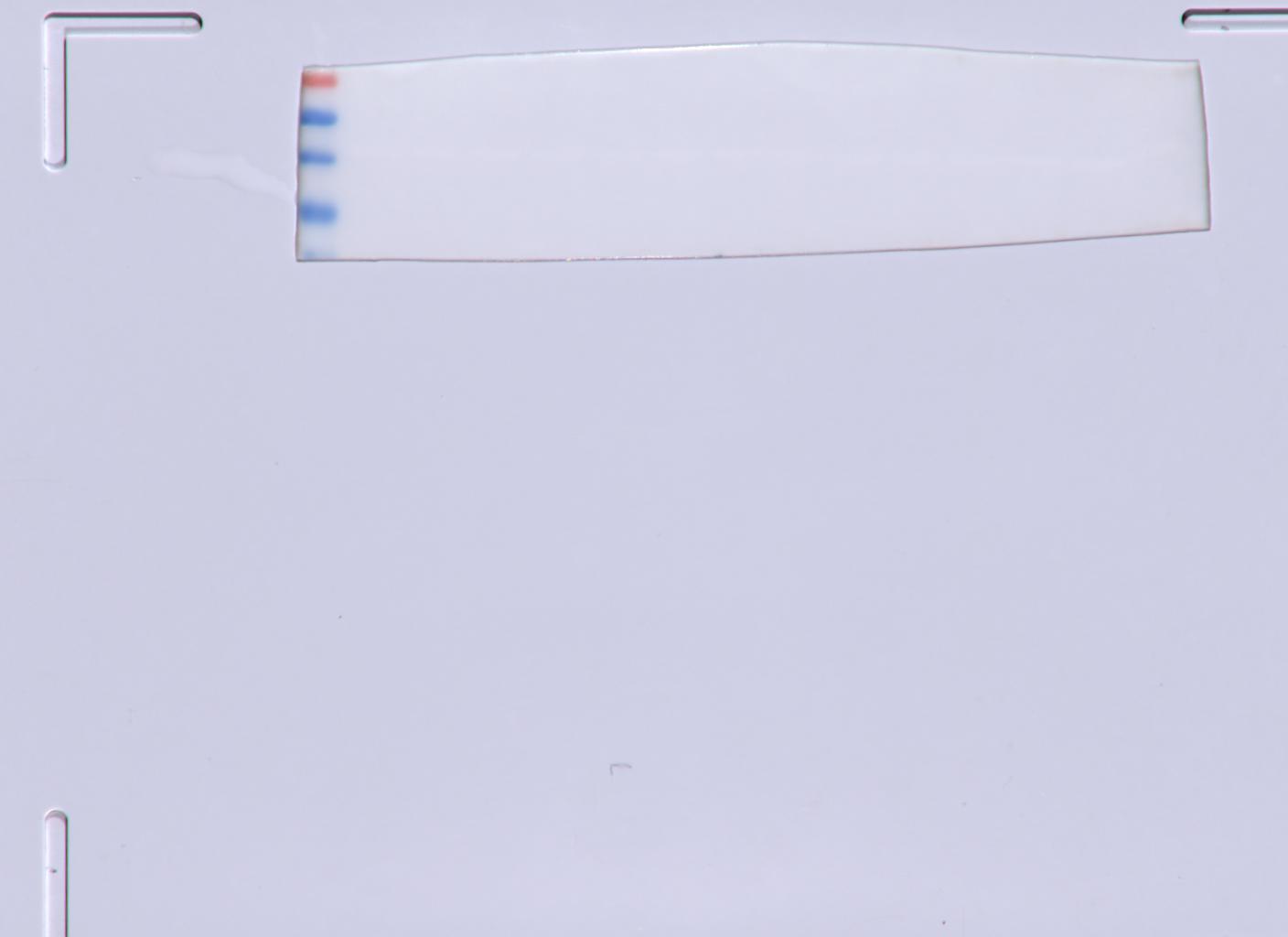

Supplement: Supplementary file 1 — Supplementary Material 1. [file 12985_2024_2385_MOESM1_ESM.zip › xuxiaoying WB/RD 3D b-actin 1 2021.09.14_16.38.13_Ch/b-actin 1 2021.09.14_16.38.13_Ch-Marker.jpg]

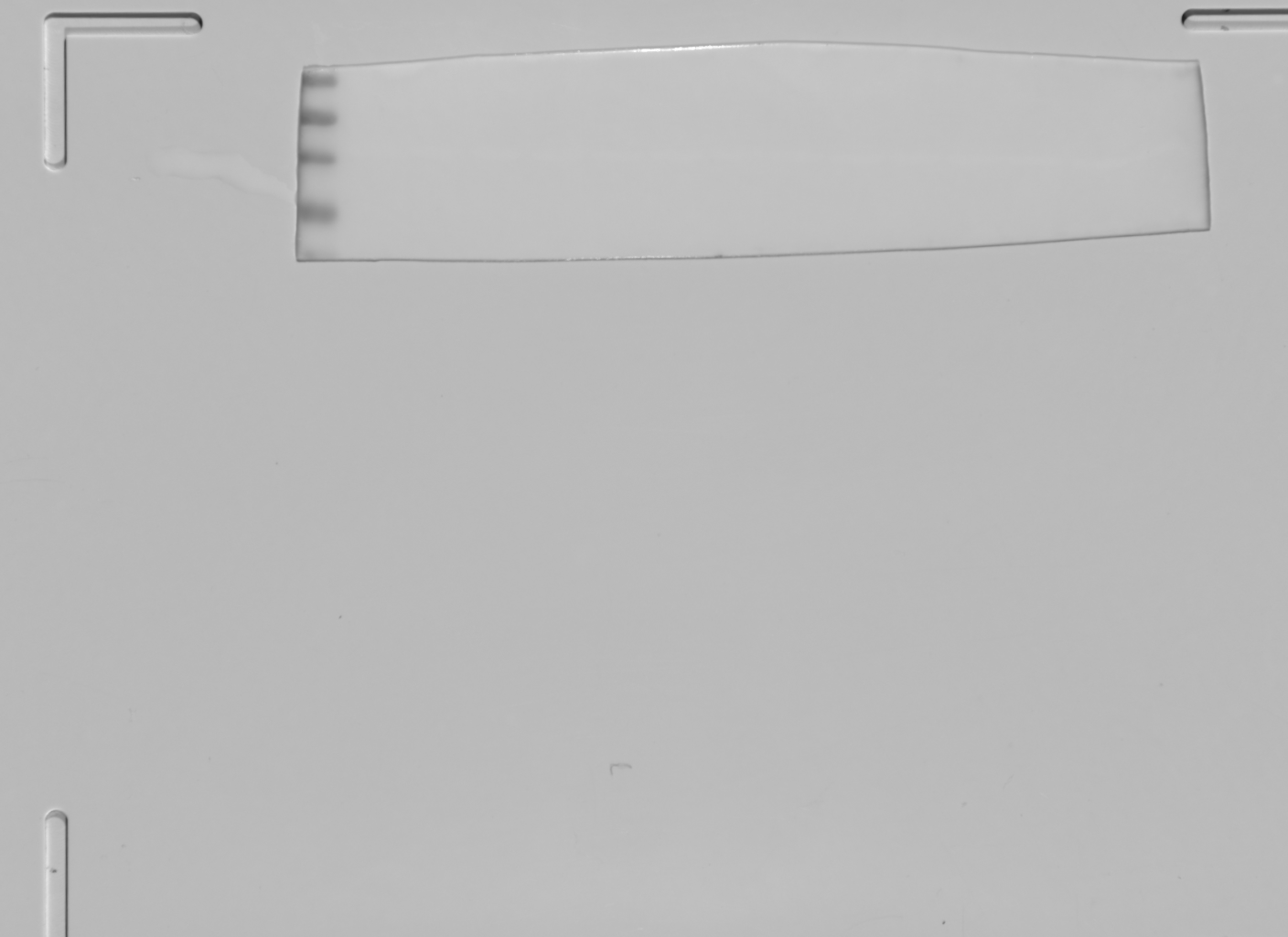

Supplement: Supplementary file 1 — Supplementary Material 1. [file 12985_2024_2385_MOESM1_ESM.zip › xuxiaoying WB/RD 3D b-actin 1 2021.09.14_16.38.13_Ch/b-actin 1 2021.09.14_16.38.13_Ch-Marker.tif]

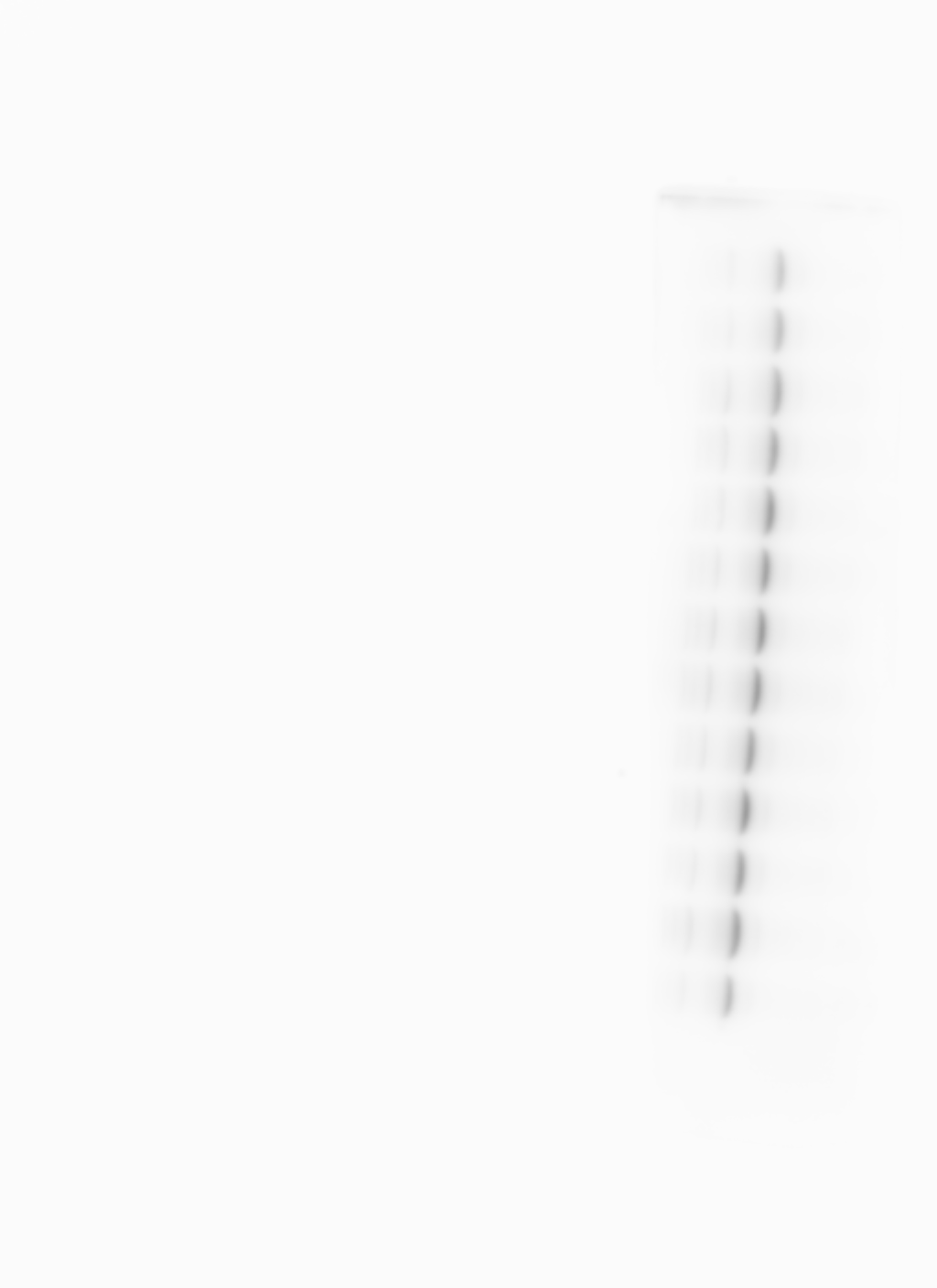

Supplement: Supplementary file 1 — Supplementary Material 1. [file 12985_2024_2385_MOESM1_ESM.zip › xuxiaoying WB/RD 3d1 2021.10.09_16.28.33_Ch/3d1 2021.10.09_16.28.33_Ch.tif]

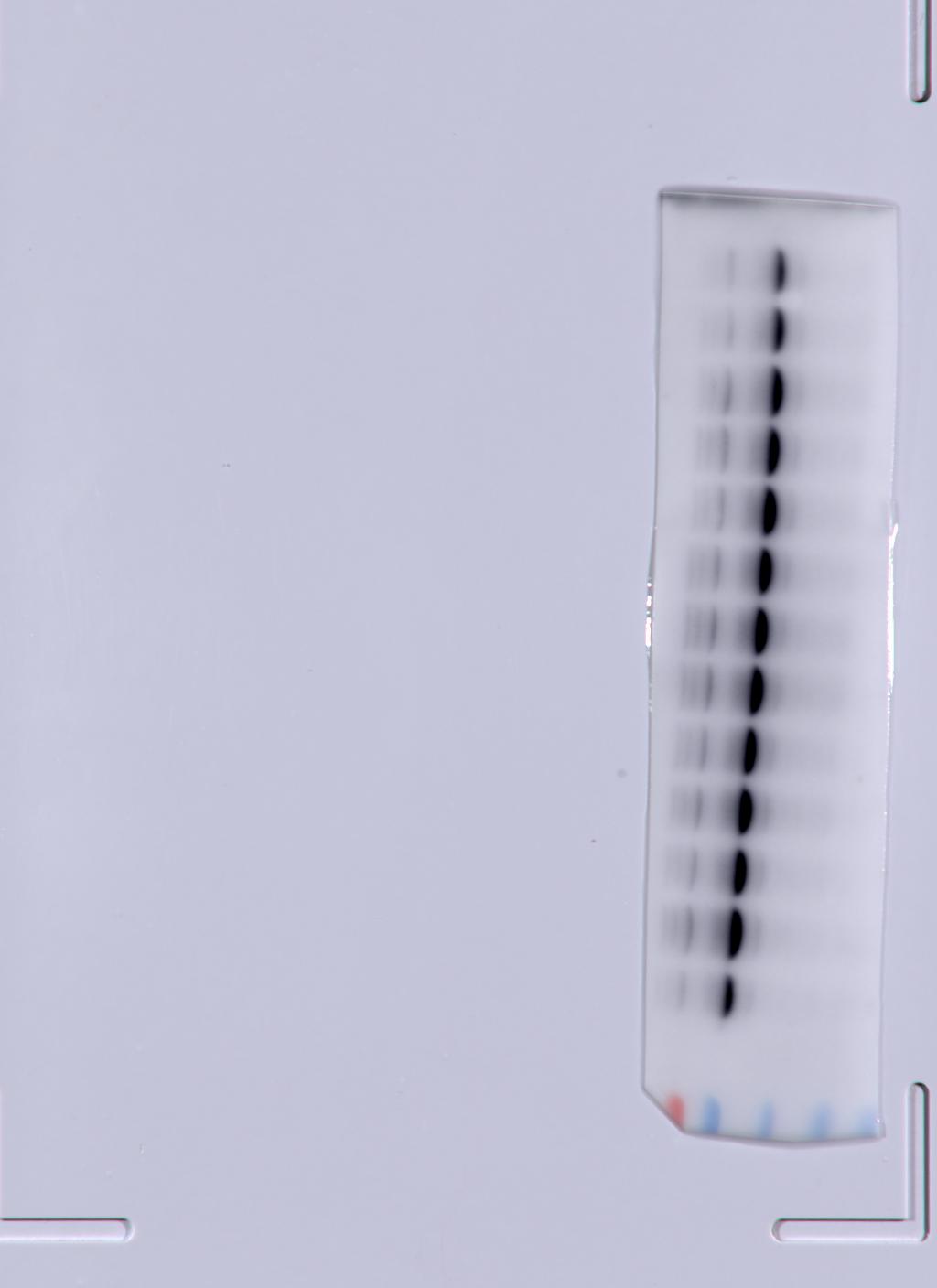

Supplement: Supplementary file 1 — Supplementary Material 1. [file 12985_2024_2385_MOESM1_ESM.zip › xuxiaoying WB/RD 3d1 2021.10.09_16.28.33_Ch/3d1 2021.10.09_16.28.33_Ch+Marker.jpg]

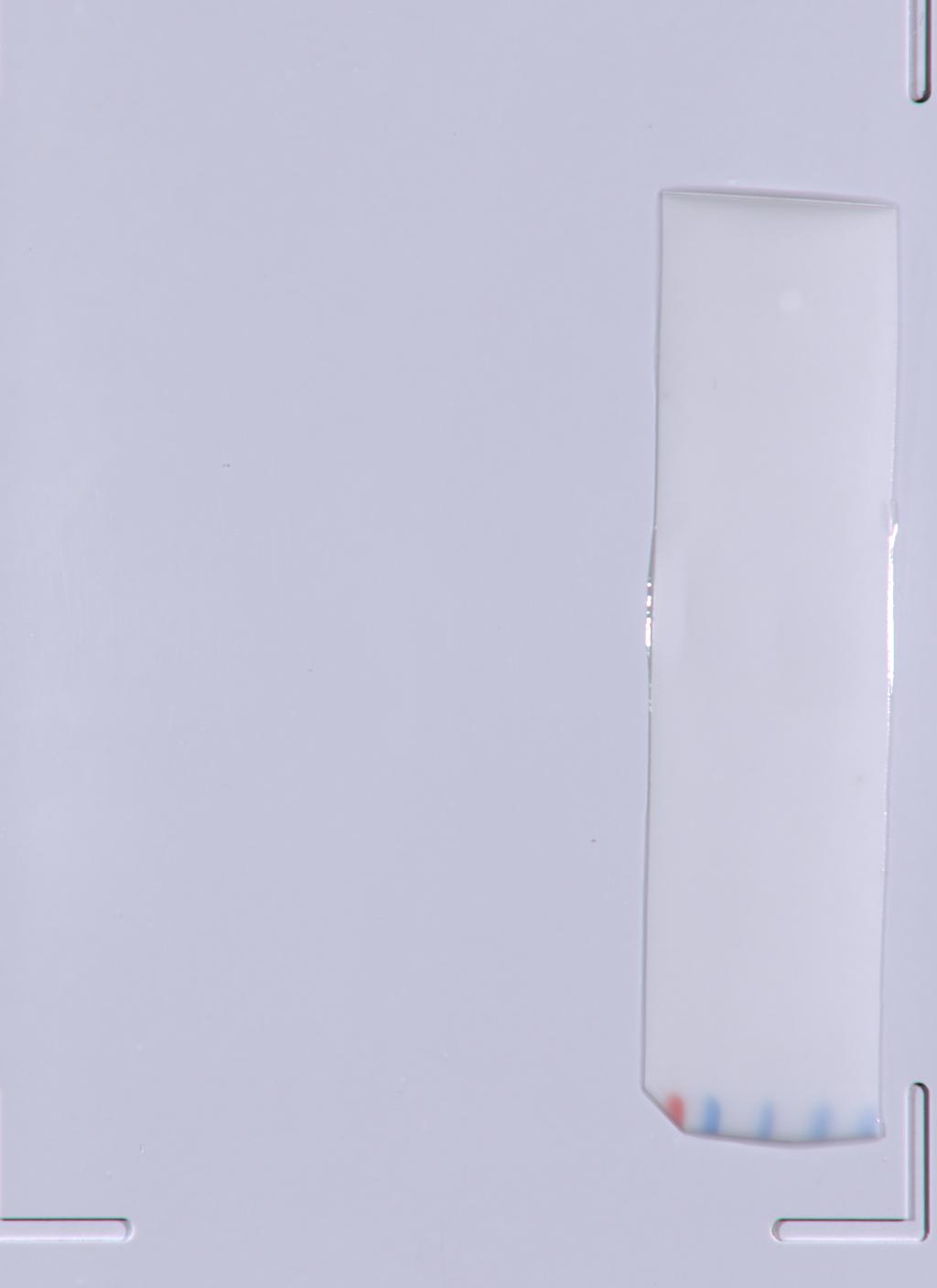

Supplement: Supplementary file 1 — Supplementary Material 1. [file 12985_2024_2385_MOESM1_ESM.zip › xuxiaoying WB/RD 3d1 2021.10.09_16.28.33_Ch/3d1 2021.10.09_16.28.33_Ch-Marker.jpg]

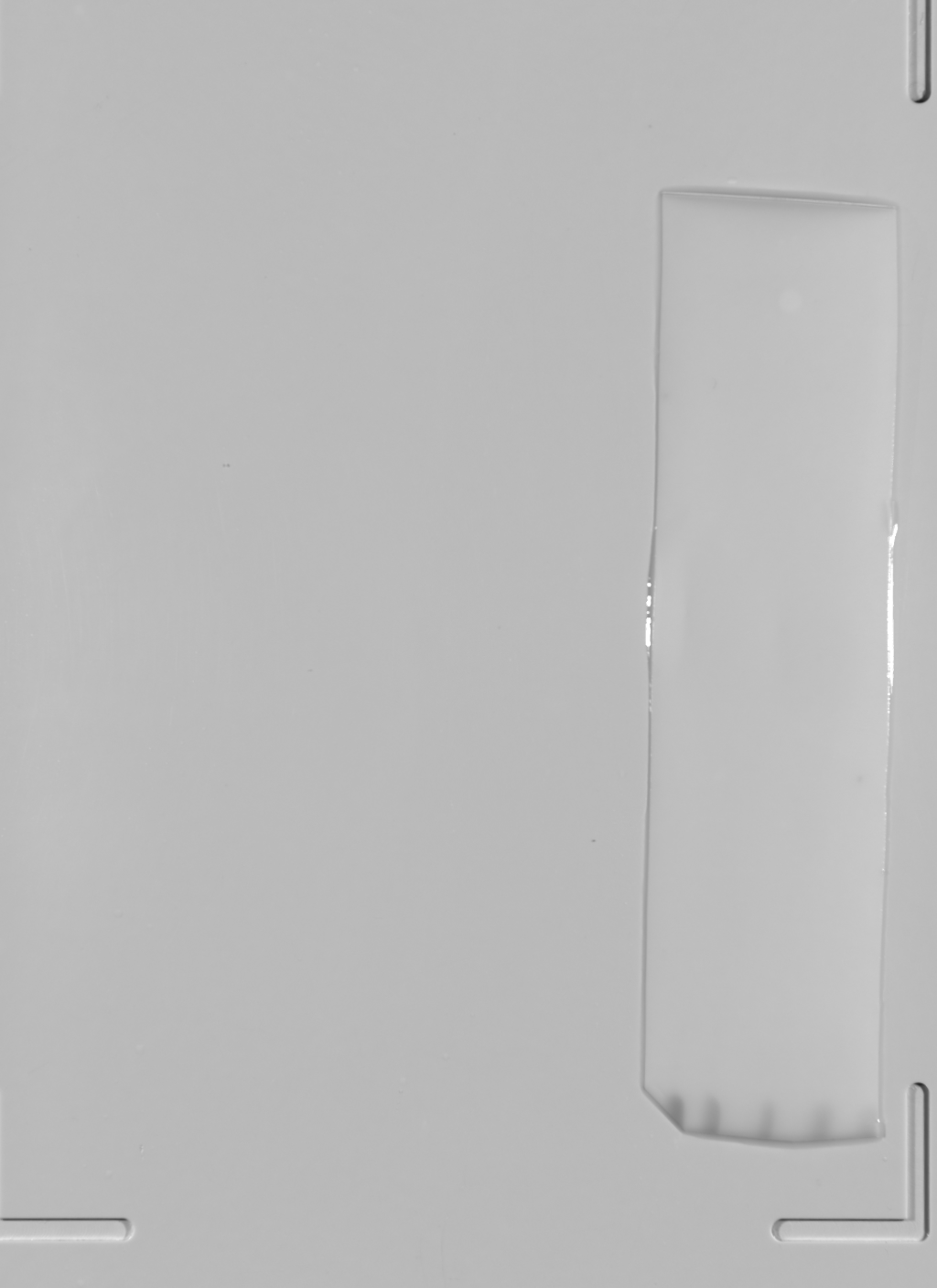

Supplement: Supplementary file 1 — Supplementary Material 1. [file 12985_2024_2385_MOESM1_ESM.zip › xuxiaoying WB/RD 3d1 2021.10.09_16.28.33_Ch/3d1 2021.10.09_16.28.33_Ch-Marker.tif]

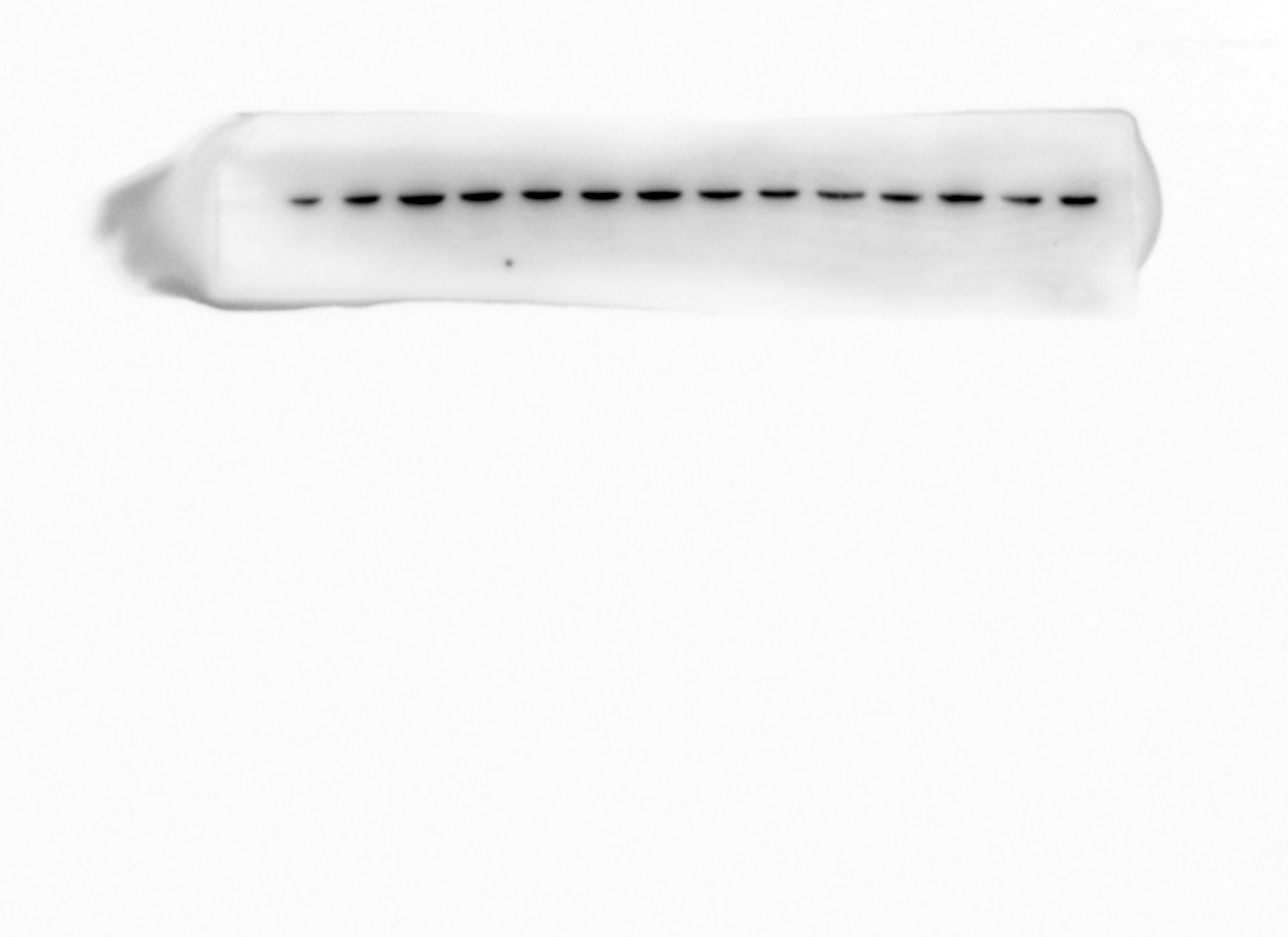

Supplement: Supplementary file 1 — Supplementary Material 1. [file 12985_2024_2385_MOESM1_ESM.zip › xuxiaoying WB/RD akt 2021.09.16_18.54.57_Ch/akt 2021.09.16_18.54.57_Ch.tif]

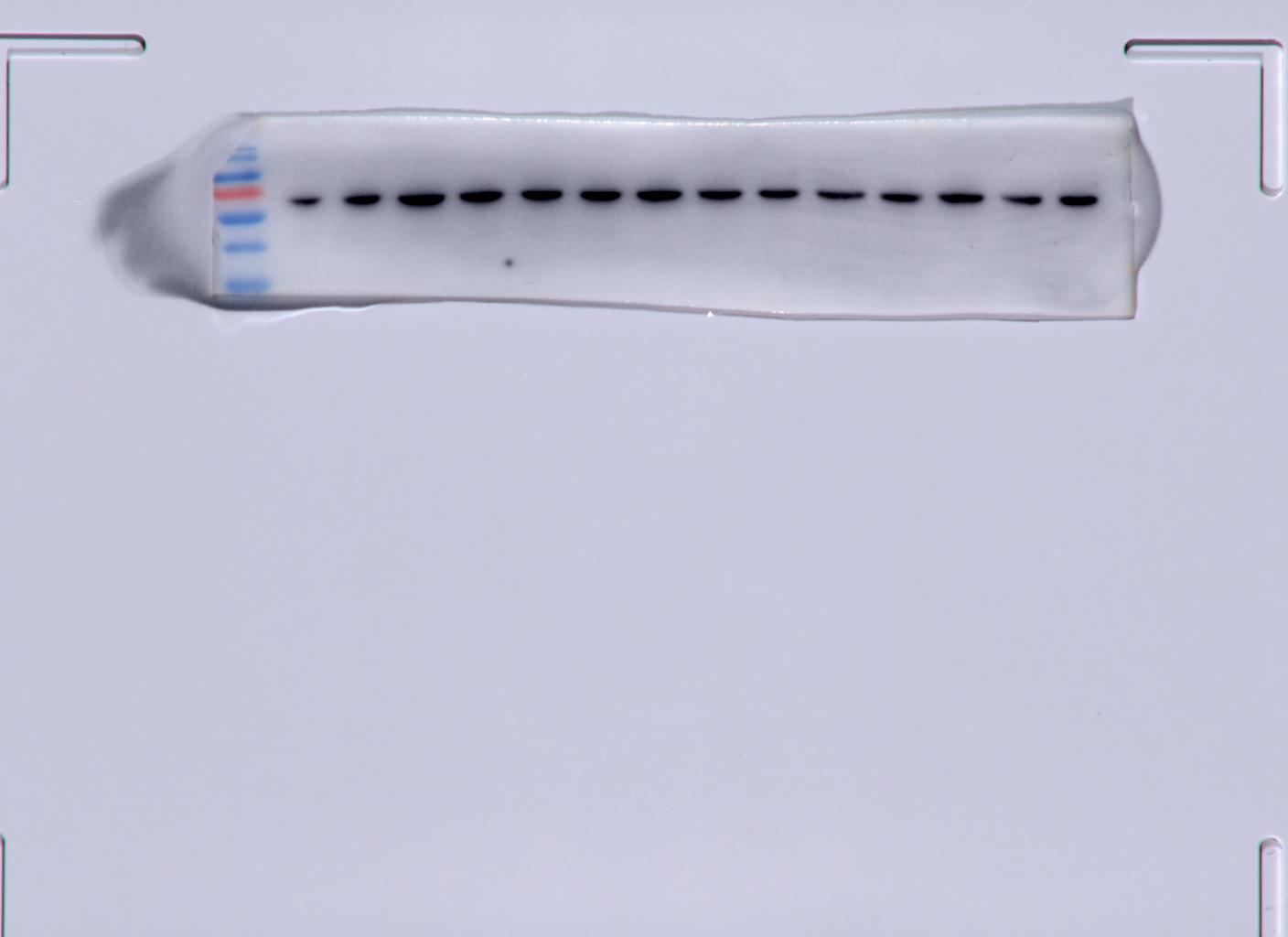

Supplement: Supplementary file 1 — Supplementary Material 1. [file 12985_2024_2385_MOESM1_ESM.zip › xuxiaoying WB/RD akt 2021.09.16_18.54.57_Ch/akt 2021.09.16_18.54.57_Ch+Marker.jpg]

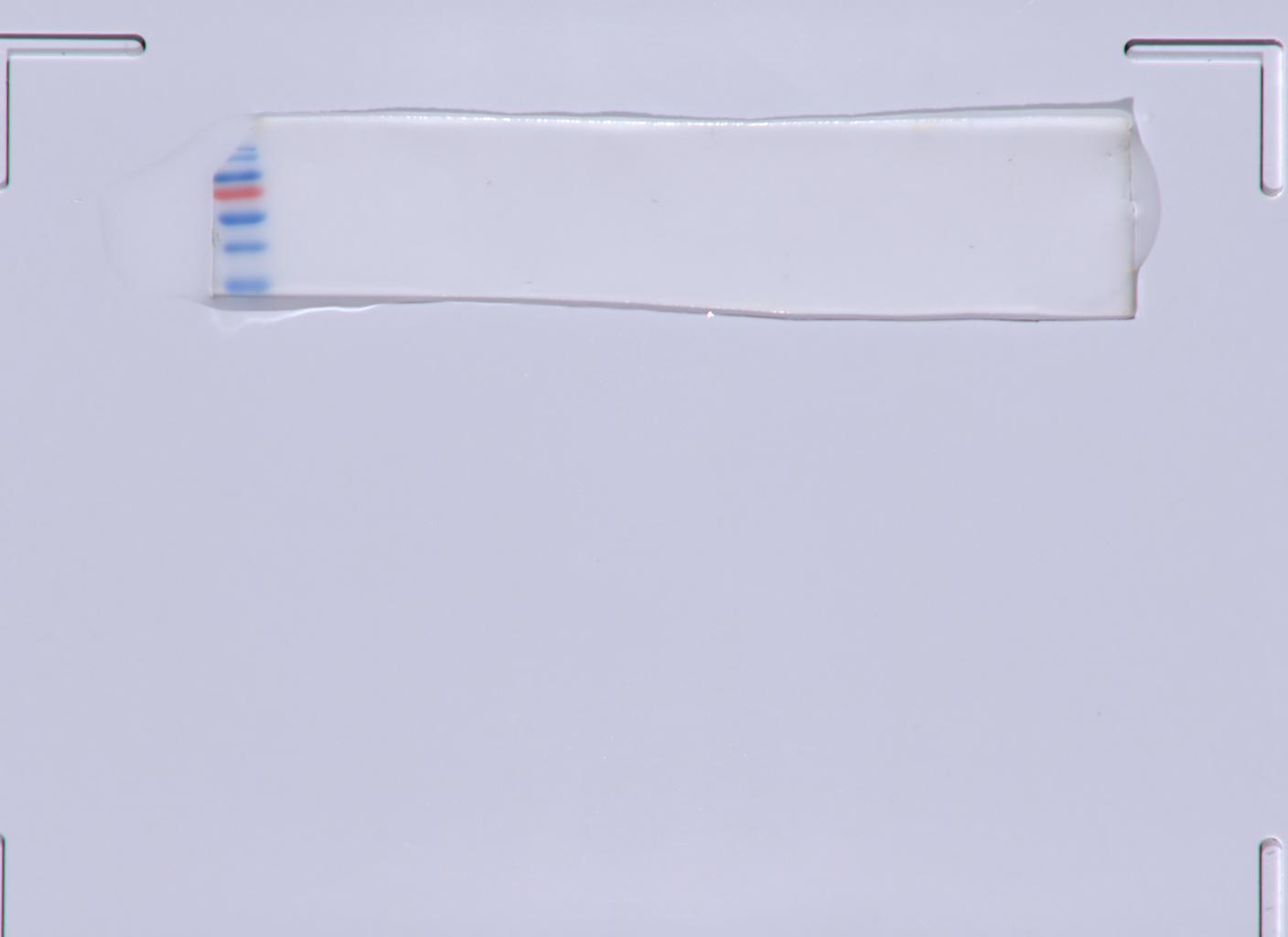

Supplement: Supplementary file 1 — Supplementary Material 1. [file 12985_2024_2385_MOESM1_ESM.zip › xuxiaoying WB/RD akt 2021.09.16_18.54.57_Ch/akt 2021.09.16_18.54.57_Ch-Marker.jpg]

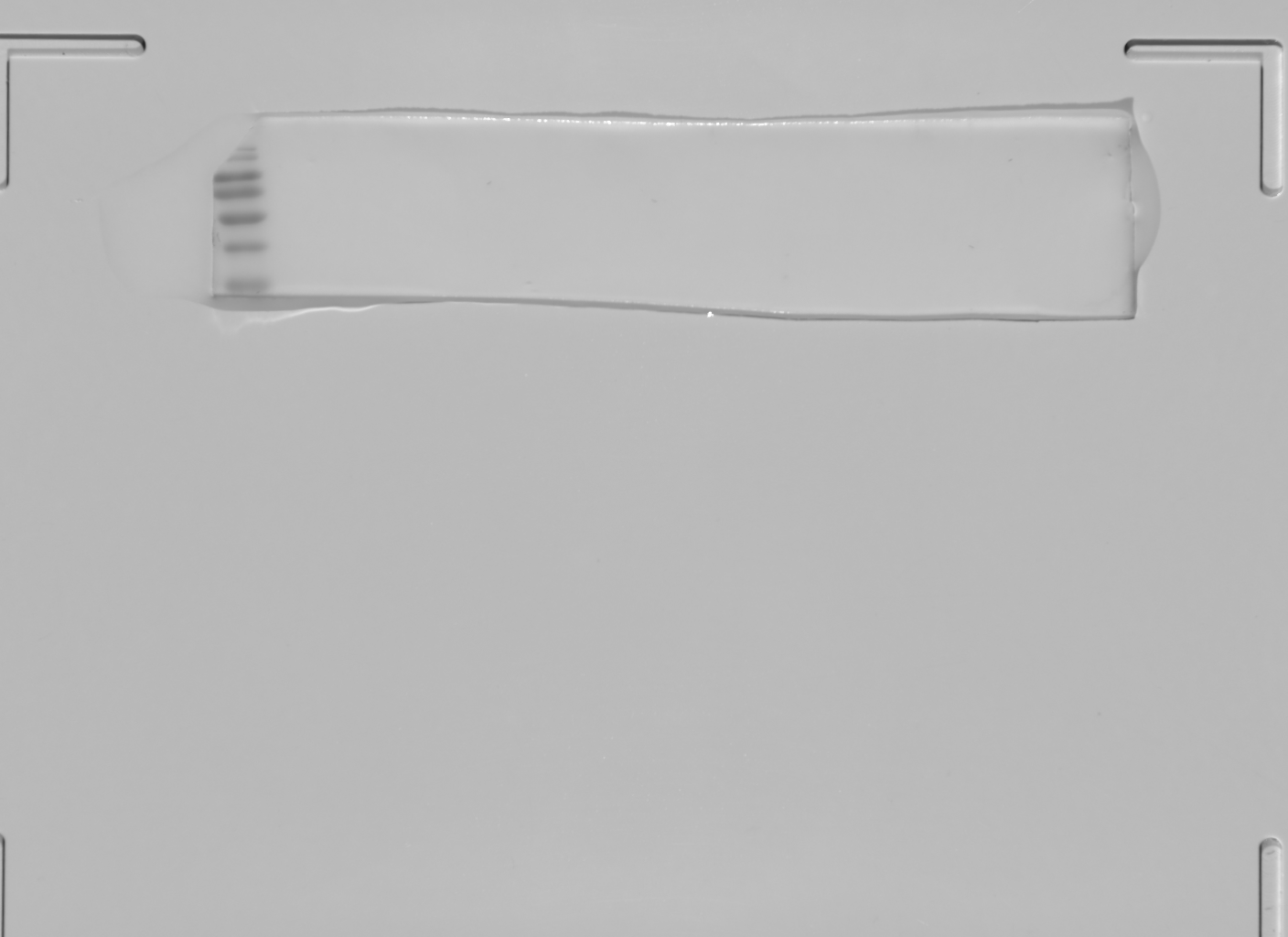

Supplement: Supplementary file 1 — Supplementary Material 1. [file 12985_2024_2385_MOESM1_ESM.zip › xuxiaoying WB/RD akt 2021.09.16_18.54.57_Ch/akt 2021.09.16_18.54.57_Ch-Marker.tif]

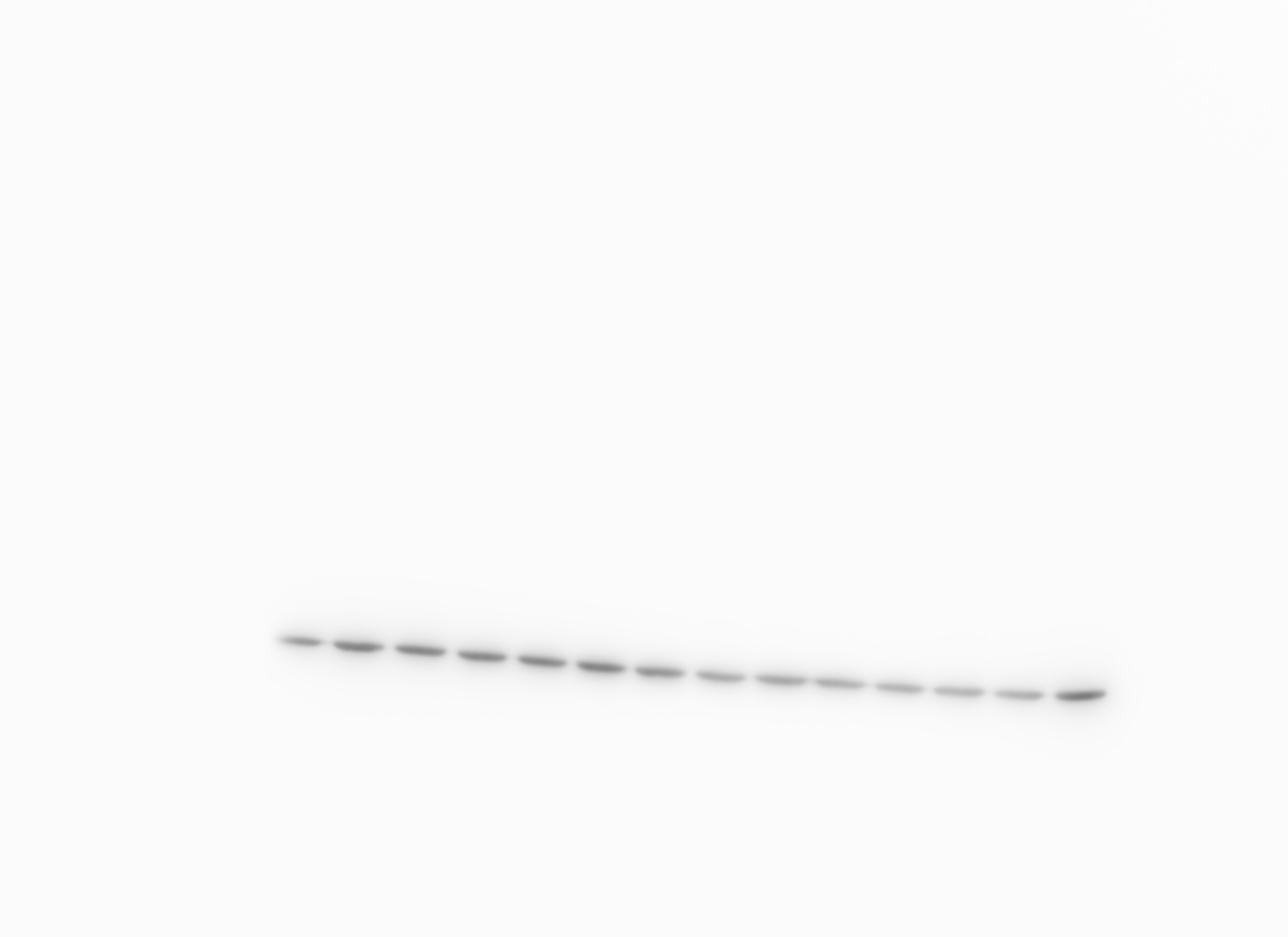

Supplement: Supplementary file 1 — Supplementary Material 1. [file 12985_2024_2385_MOESM1_ESM.zip › xuxiaoying WB/RD AKT b-actin 3 2021.09.14_16.54.12_Ch/b-actin 3 2021.09.14_16.54.12_Ch.tif]

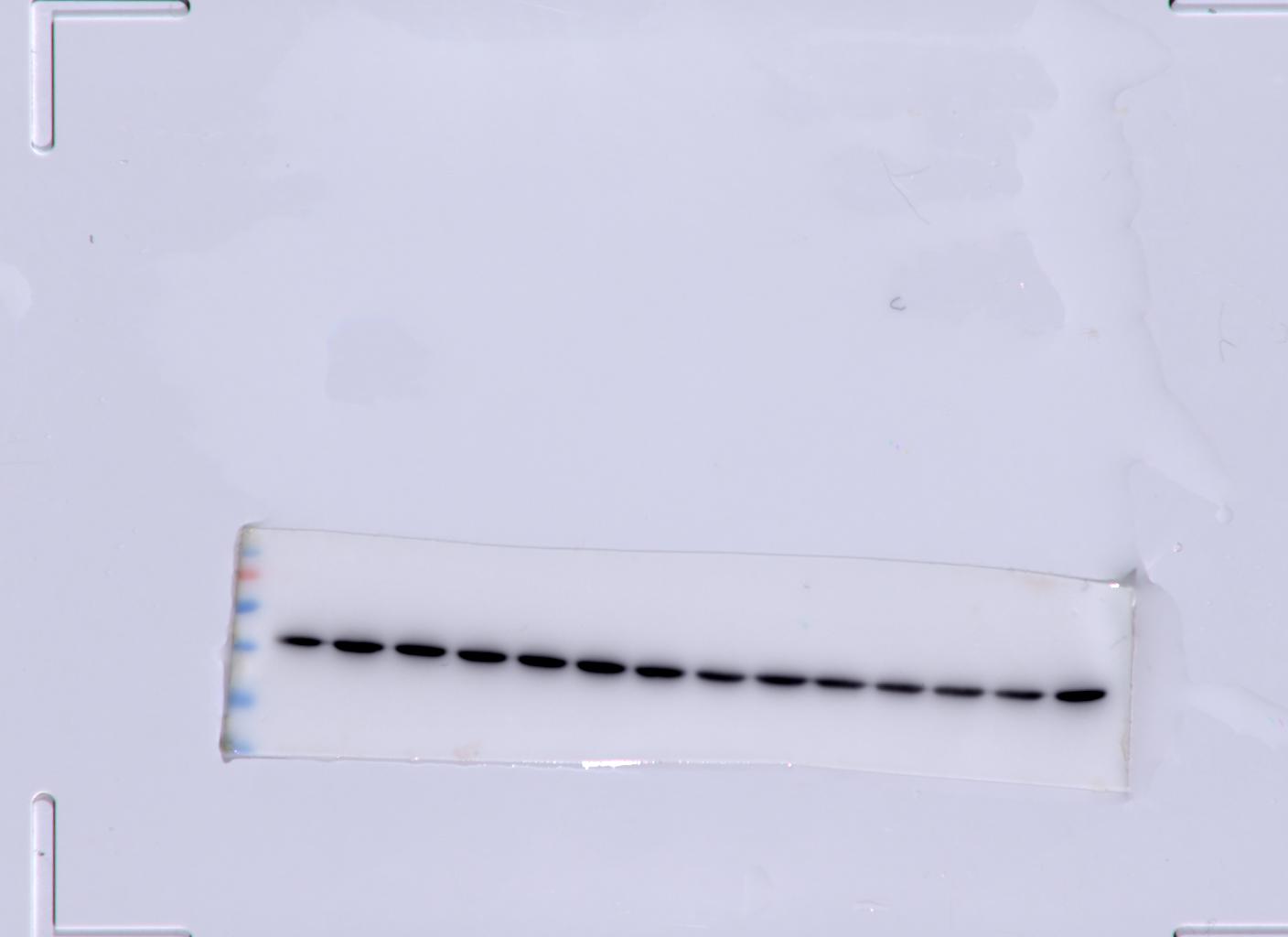

Supplement: Supplementary file 1 — Supplementary Material 1. [file 12985_2024_2385_MOESM1_ESM.zip › xuxiaoying WB/RD AKT b-actin 3 2021.09.14_16.54.12_Ch/b-actin 3 2021.09.14_16.54.12_Ch+Marker.jpg]

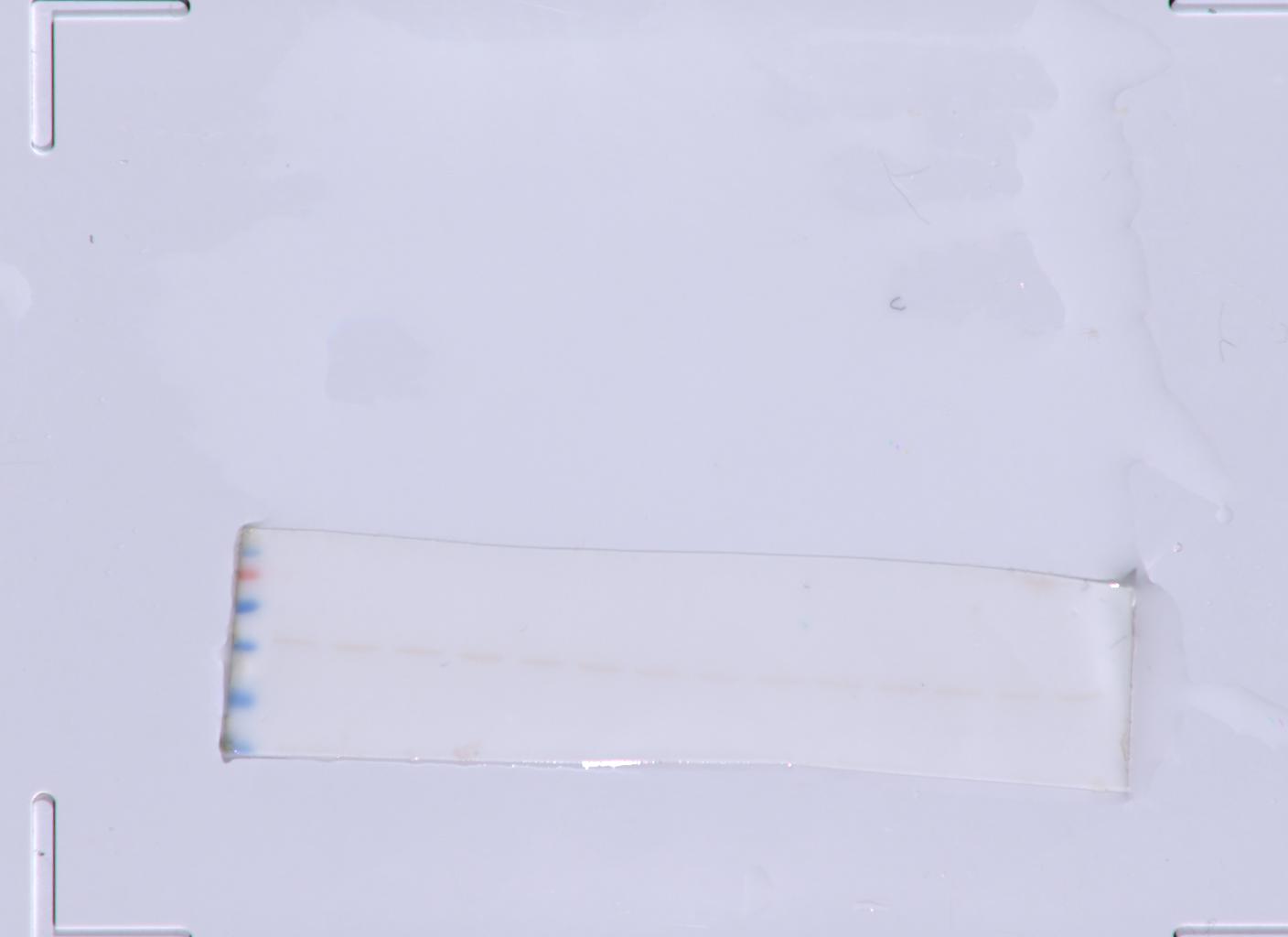

Supplement: Supplementary file 1 — Supplementary Material 1. [file 12985_2024_2385_MOESM1_ESM.zip › xuxiaoying WB/RD AKT b-actin 3 2021.09.14_16.54.12_Ch/b-actin 3 2021.09.14_16.54.12_Ch-Marker.jpg]

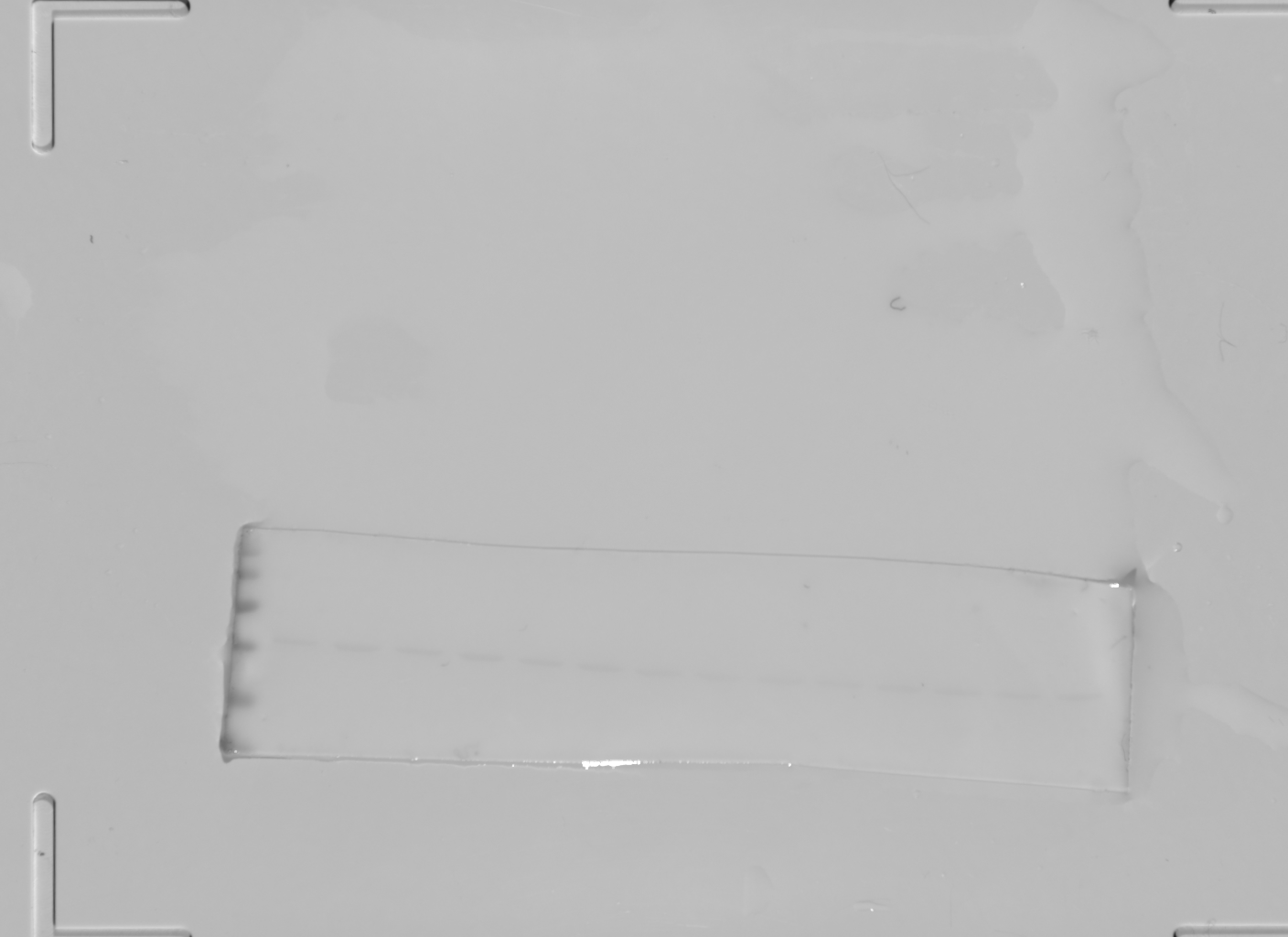

Supplement: Supplementary file 1 — Supplementary Material 1. [file 12985_2024_2385_MOESM1_ESM.zip › xuxiaoying WB/RD AKT b-actin 3 2021.09.14_16.54.12_Ch/b-actin 3 2021.09.14_16.54.12_Ch-Marker.tif]

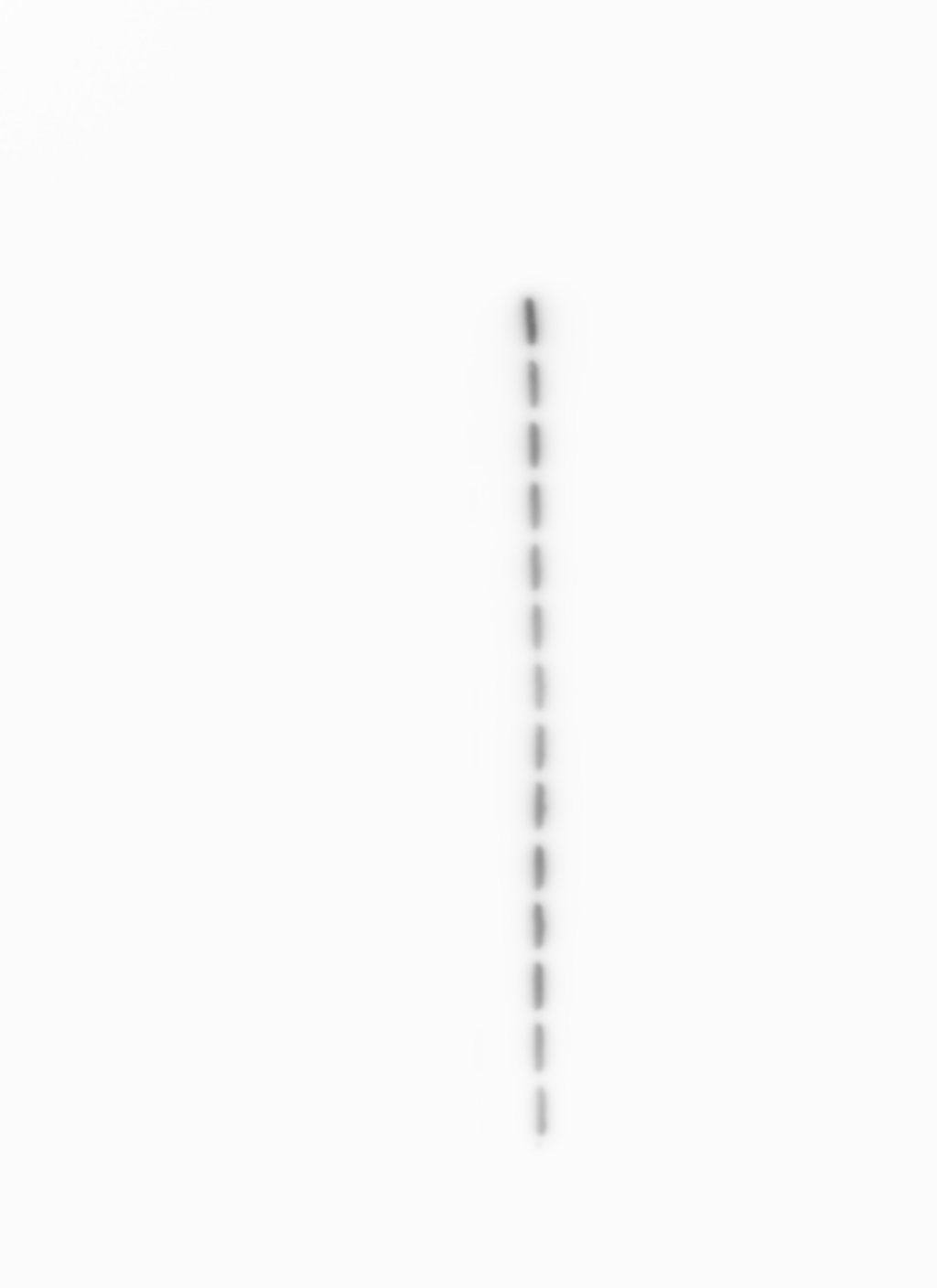

Supplement: Supplementary file 1 — Supplementary Material 1. [file 12985_2024_2385_MOESM1_ESM.zip › xuxiaoying WB/RD lc3 b-actin 5 2021.09.14_17.05.57_Ch/b-actin 5 2021.09.14_17.05.57_Ch.tif]

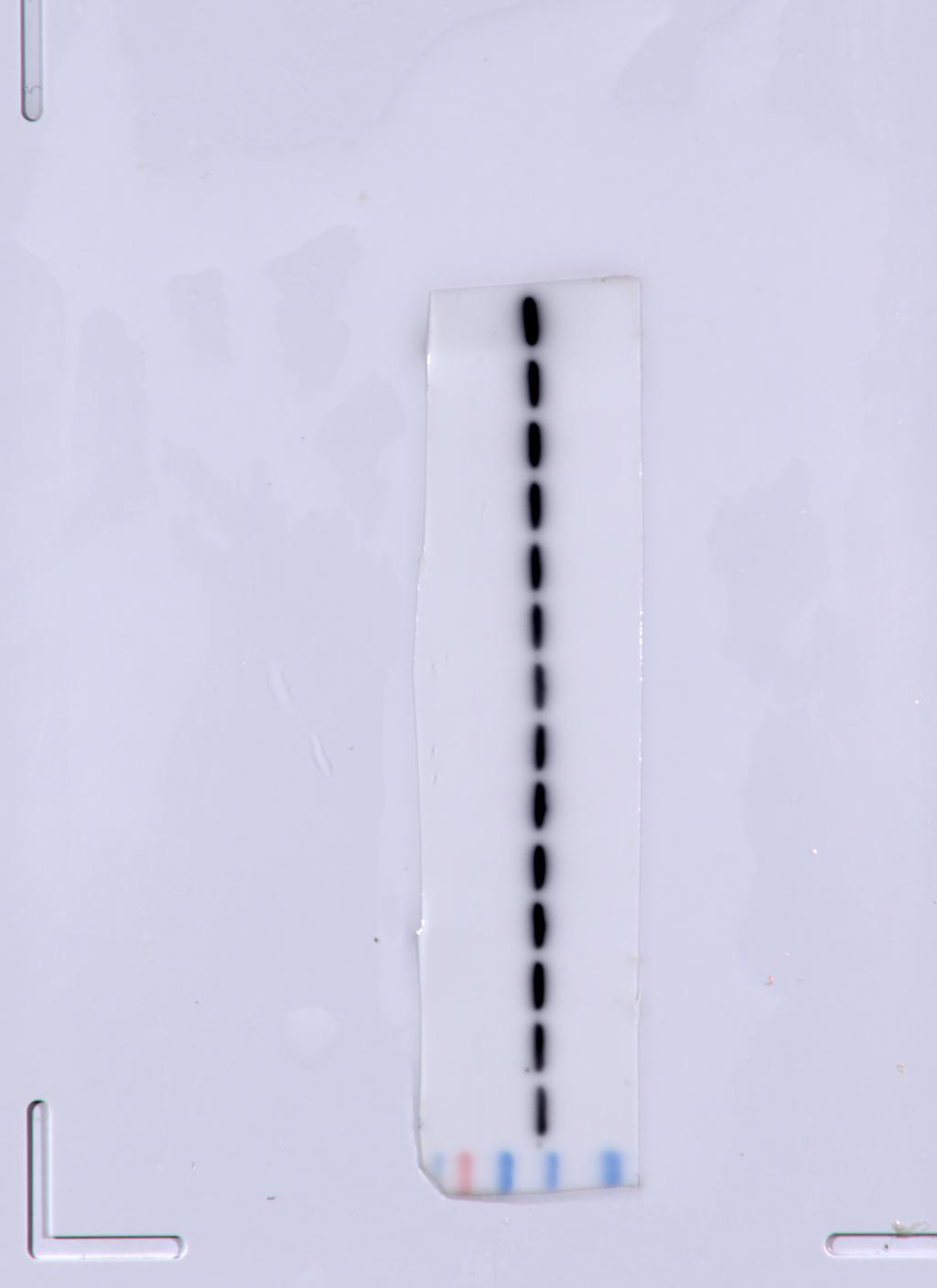

Supplement: Supplementary file 1 — Supplementary Material 1. [file 12985_2024_2385_MOESM1_ESM.zip › xuxiaoying WB/RD lc3 b-actin 5 2021.09.14_17.05.57_Ch/b-actin 5 2021.09.14_17.05.57_Ch+Marker.jpg]

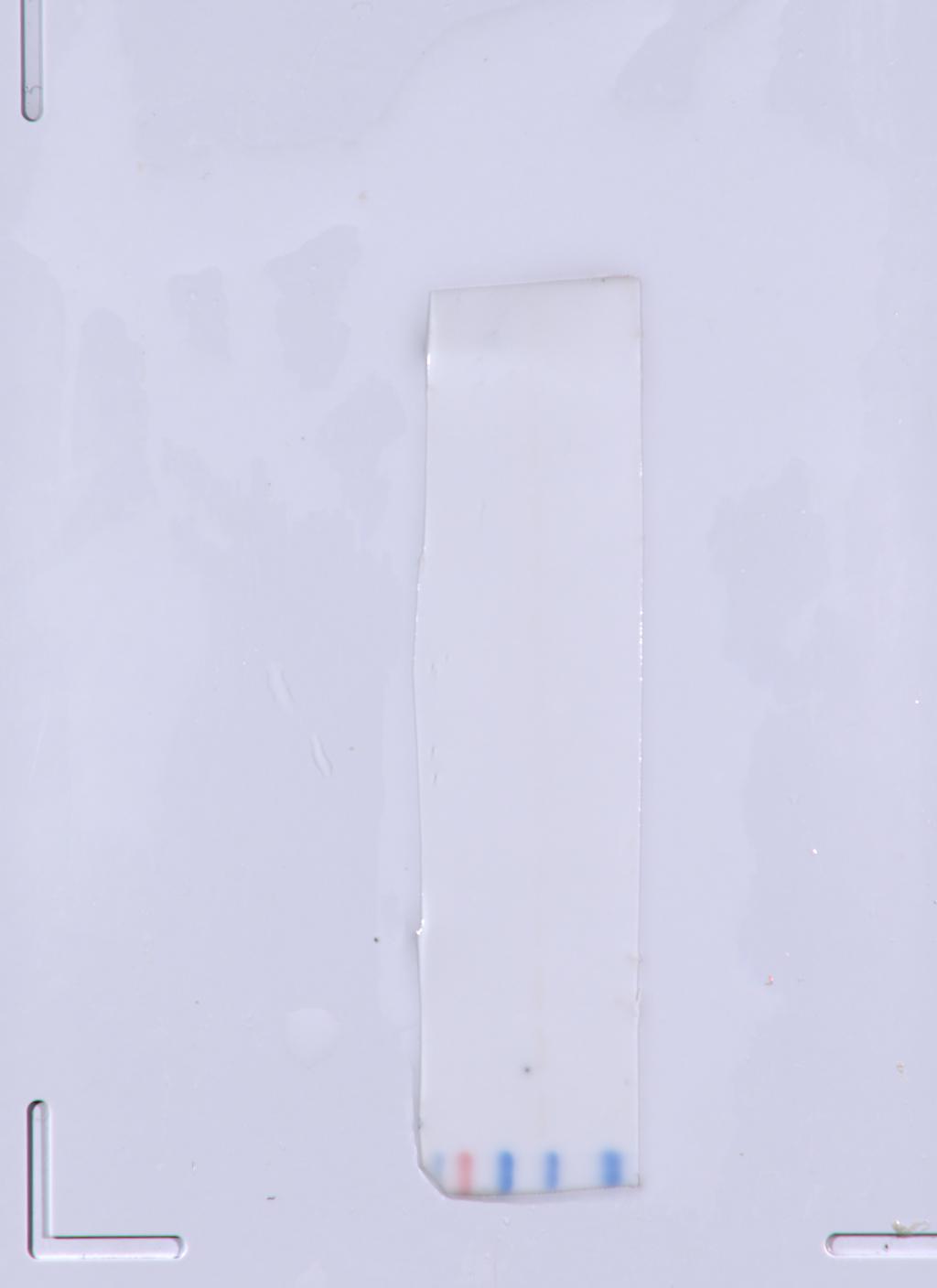

Supplement: Supplementary file 1 — Supplementary Material 1. [file 12985_2024_2385_MOESM1_ESM.zip › xuxiaoying WB/RD lc3 b-actin 5 2021.09.14_17.05.57_Ch/b-actin 5 2021.09.14_17.05.57_Ch-Marker.jpg]

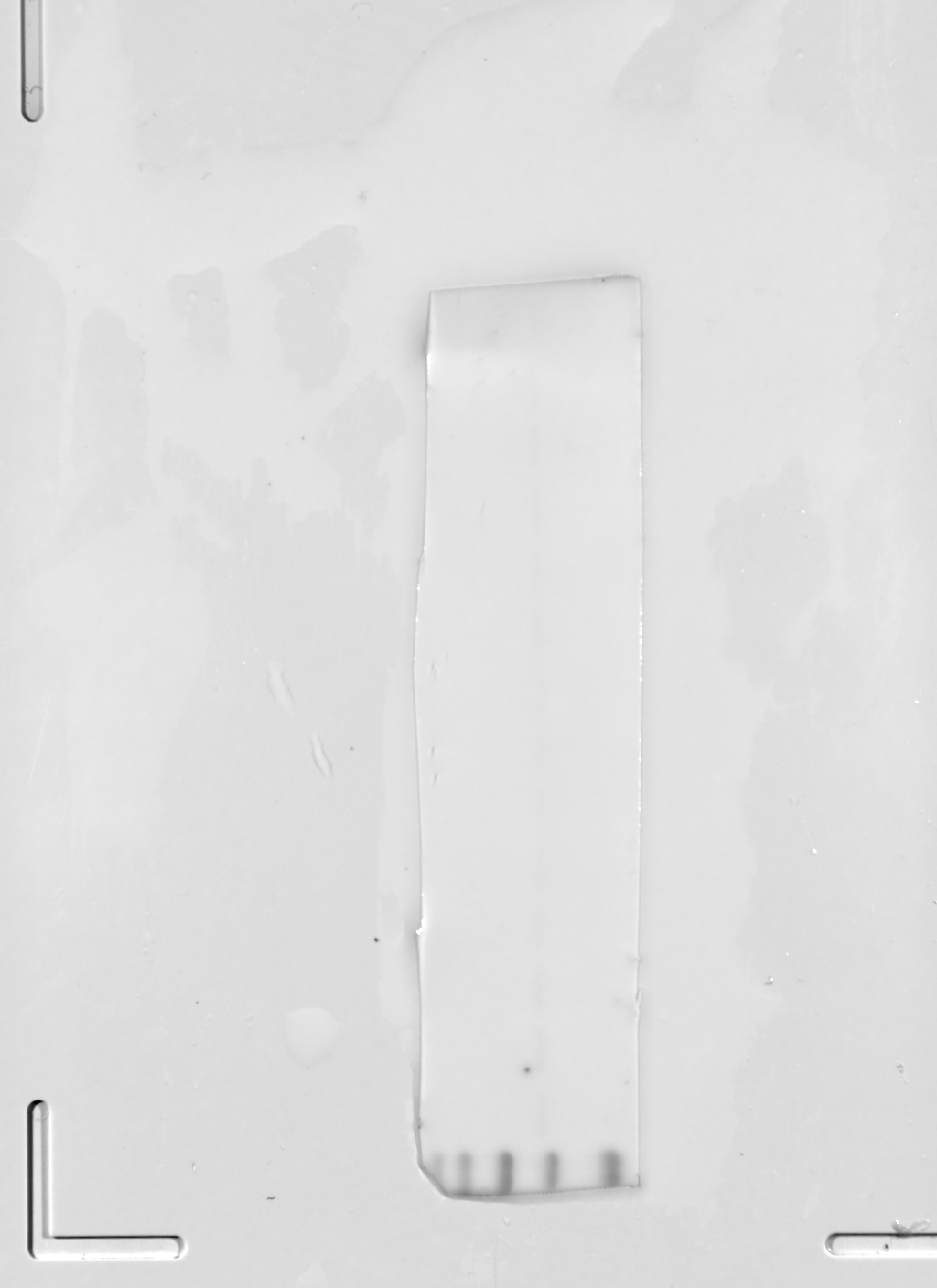

Supplement: Supplementary file 1 — Supplementary Material 1. [file 12985_2024_2385_MOESM1_ESM.zip › xuxiaoying WB/RD lc3 b-actin 5 2021.09.14_17.05.57_Ch/b-actin 5 2021.09.14_17.05.57_Ch-Marker.tif]

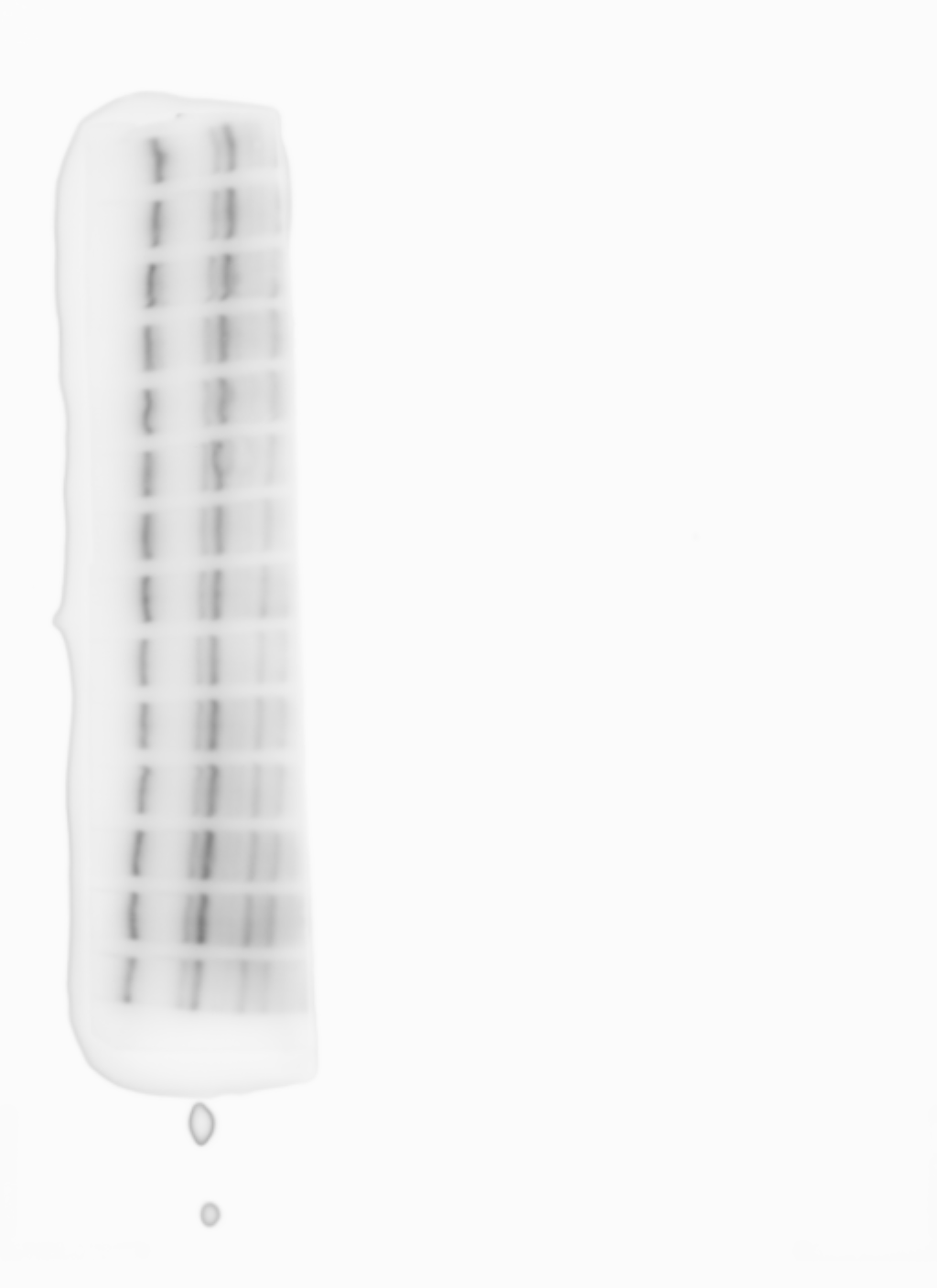

Supplement: Supplementary file 1 — Supplementary Material 1. [file 12985_2024_2385_MOESM1_ESM.zip › xuxiaoying WB/RD mTOR1 2021.10.31_22.44.23_Ch/mTOR1 2021.10.31_22.44.23_Ch.tif]

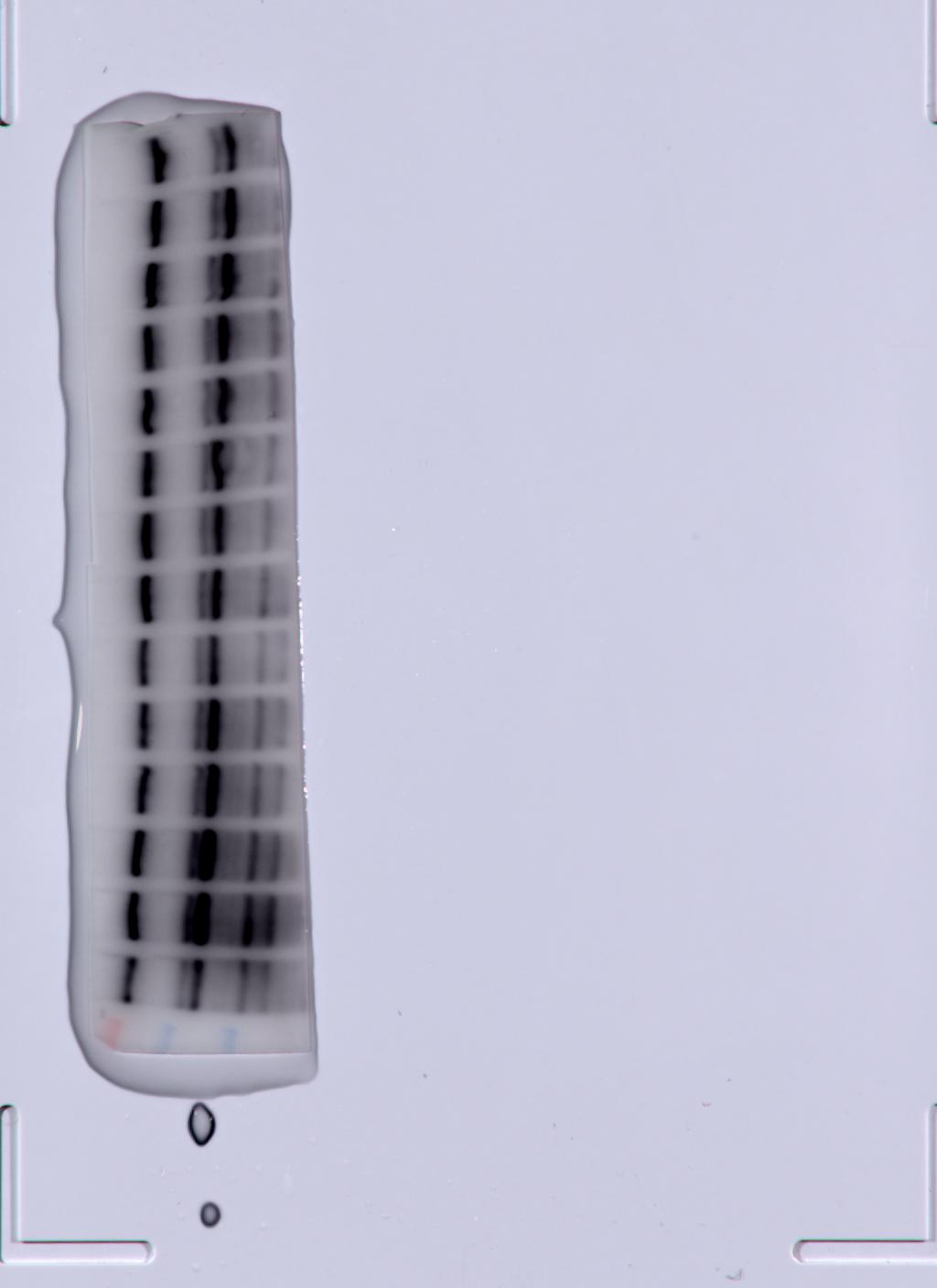

Supplement: Supplementary file 1 — Supplementary Material 1. [file 12985_2024_2385_MOESM1_ESM.zip › xuxiaoying WB/RD mTOR1 2021.10.31_22.44.23_Ch/mTOR1 2021.10.31_22.44.23_Ch+Marker.jpg]

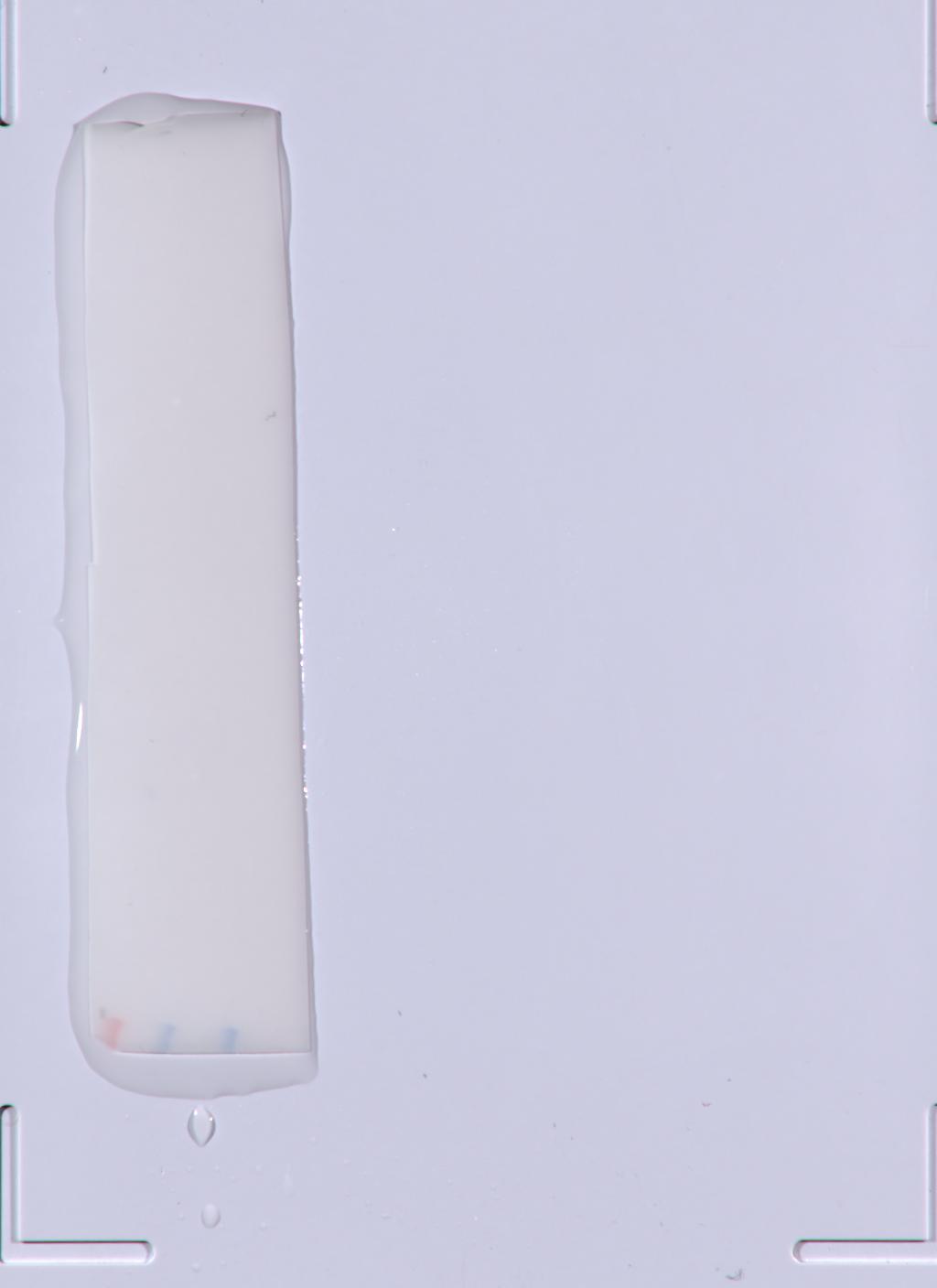

Supplement: Supplementary file 1 — Supplementary Material 1. [file 12985_2024_2385_MOESM1_ESM.zip › xuxiaoying WB/RD mTOR1 2021.10.31_22.44.23_Ch/mTOR1 2021.10.31_22.44.23_Ch-Marker.jpg]

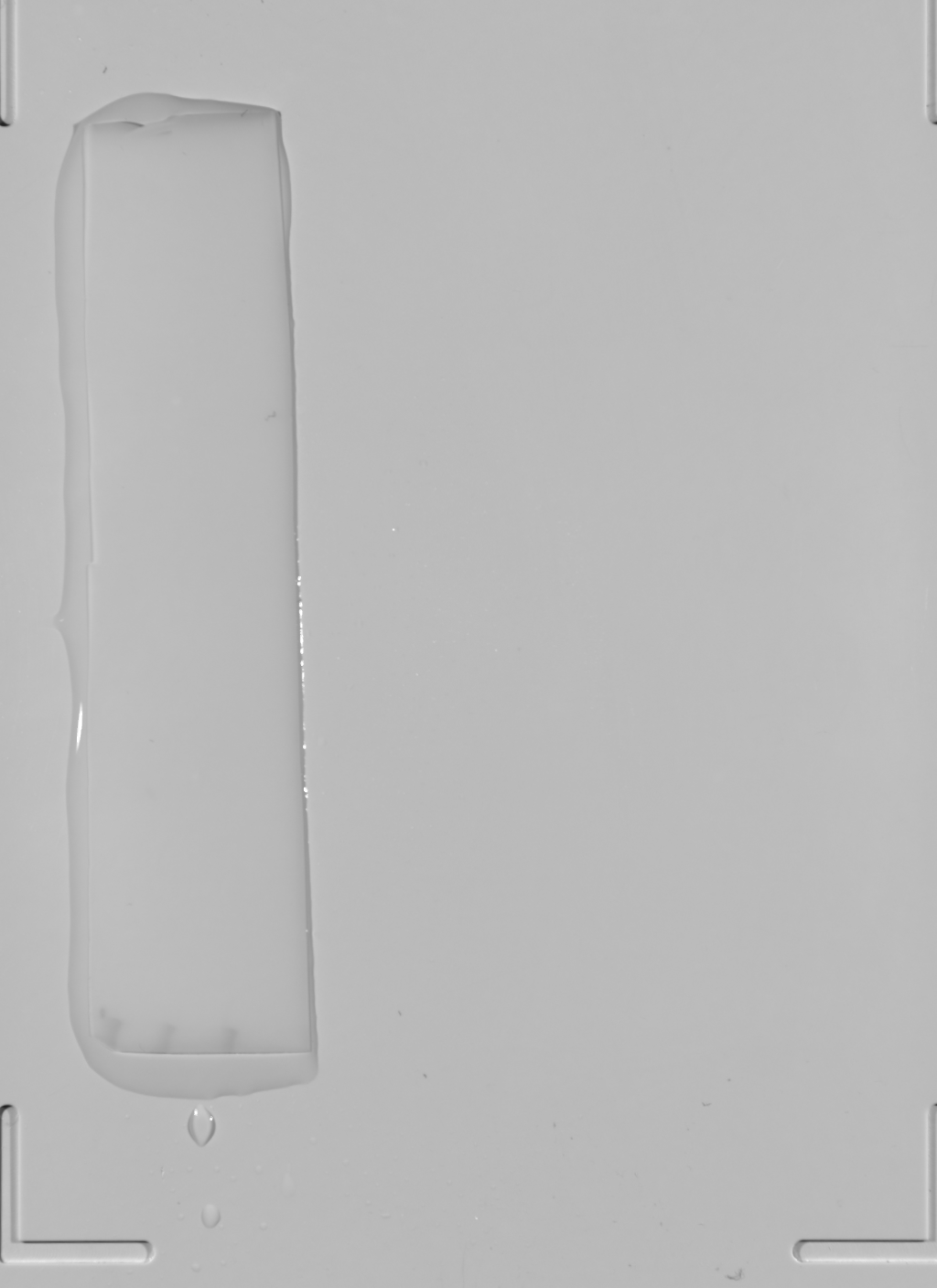

Supplement: Supplementary file 1 — Supplementary Material 1. [file 12985_2024_2385_MOESM1_ESM.zip › xuxiaoying WB/RD mTOR1 2021.10.31_22.44.23_Ch/mTOR1 2021.10.31_22.44.23_Ch-Marker.tif]

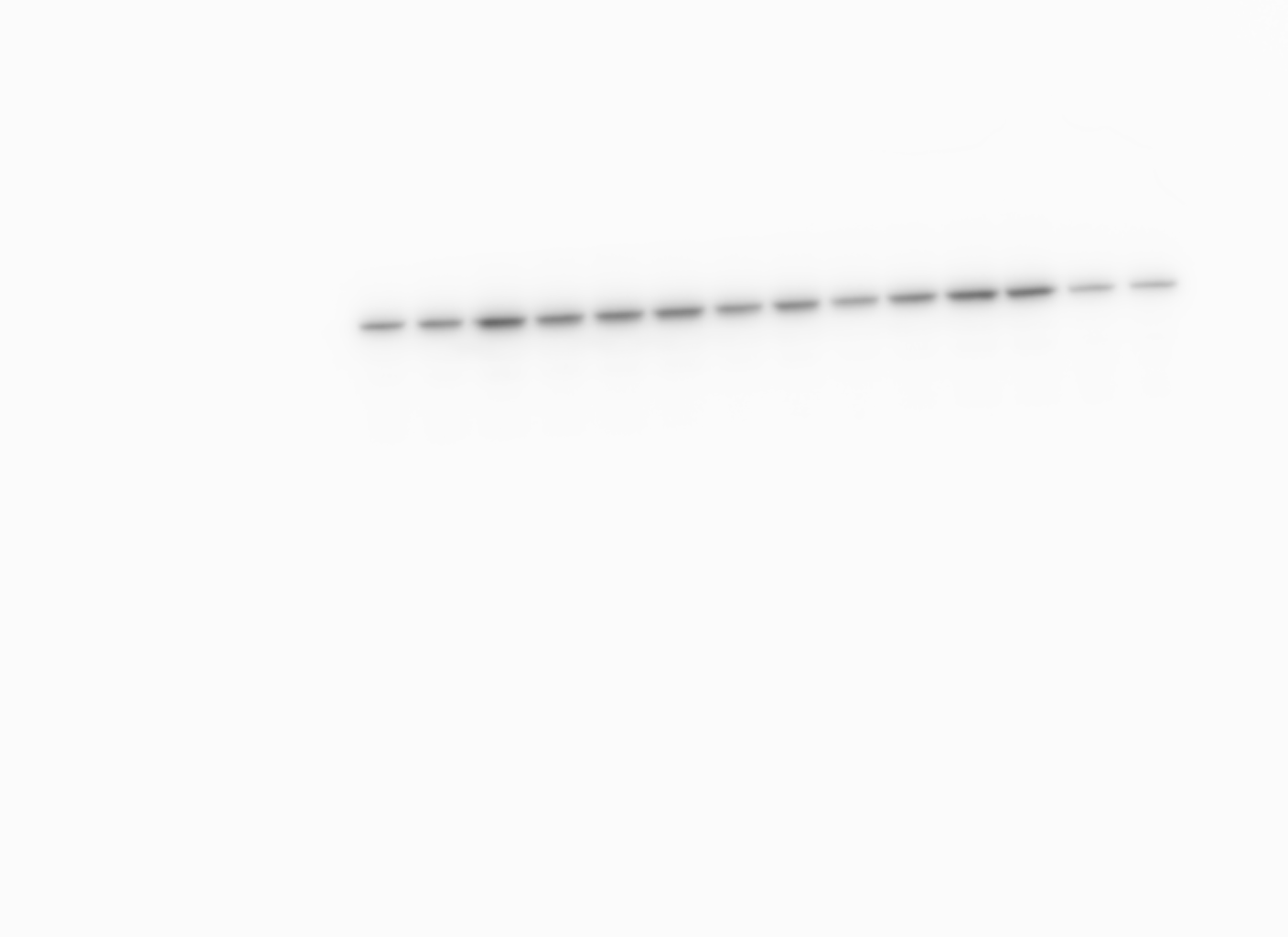

Supplement: Supplementary file 1 — Supplementary Material 1. [file 12985_2024_2385_MOESM1_ESM.zip › xuxiaoying WB/RD p62 2021.09.14_17.51.29_Ch/lp62 2021.09.14_17.51.29_Ch.tif]

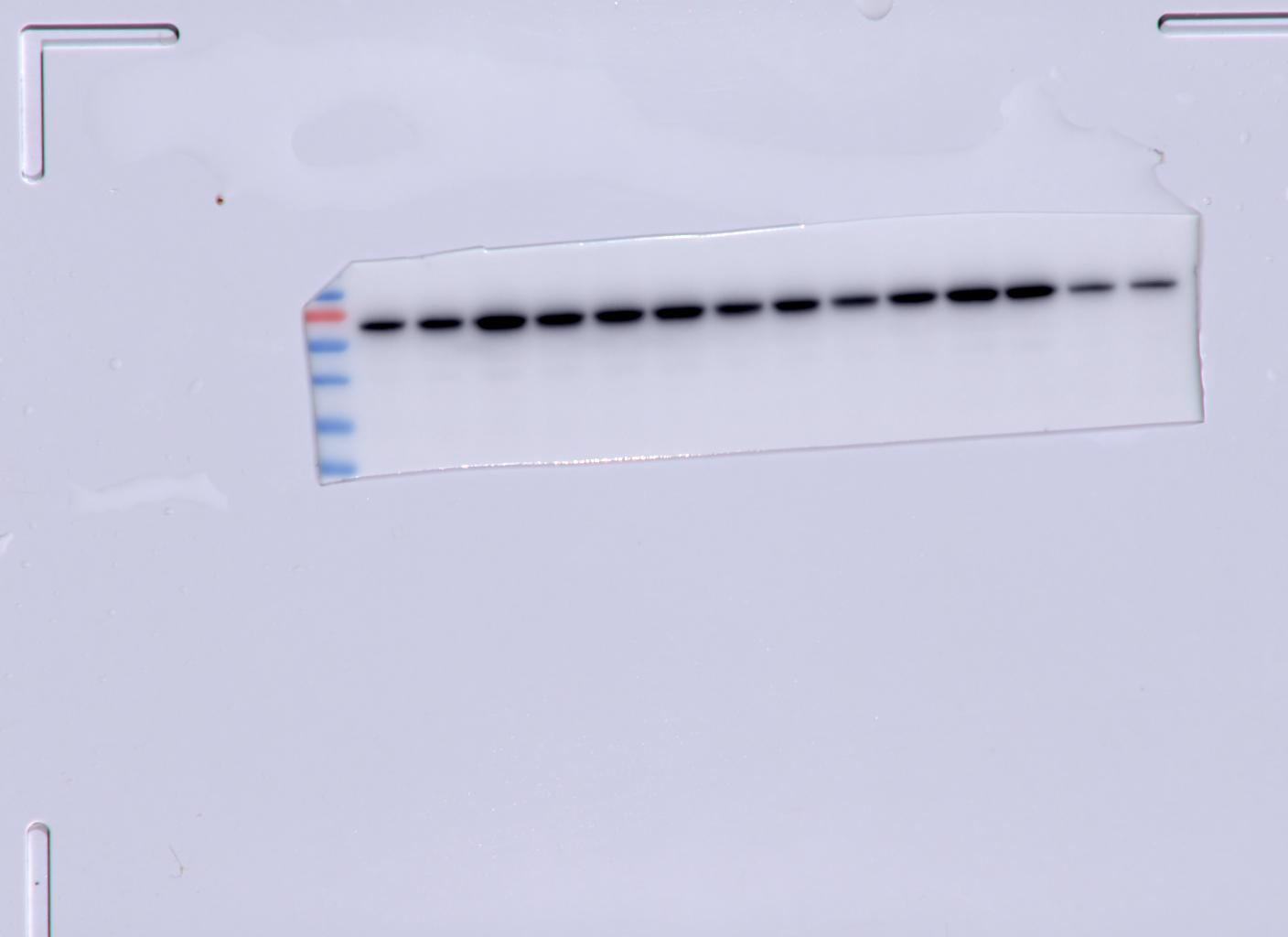

Supplement: Supplementary file 1 — Supplementary Material 1. [file 12985_2024_2385_MOESM1_ESM.zip › xuxiaoying WB/RD p62 2021.09.14_17.51.29_Ch/lp62 2021.09.14_17.51.29_Ch+Marker.jpg]

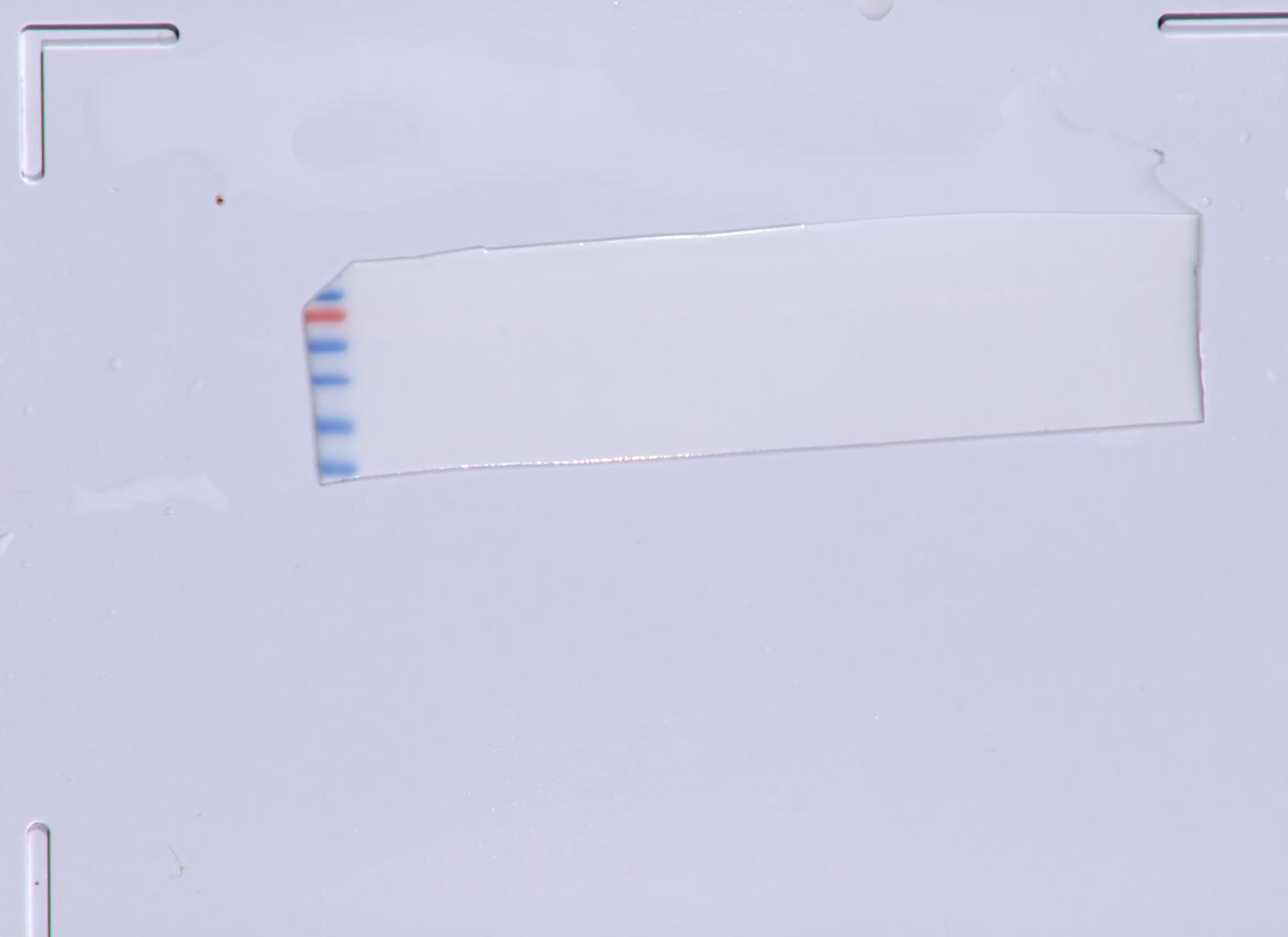

Supplement: Supplementary file 1 — Supplementary Material 1. [file 12985_2024_2385_MOESM1_ESM.zip › xuxiaoying WB/RD p62 2021.09.14_17.51.29_Ch/lp62 2021.09.14_17.51.29_Ch-Marker.jpg]

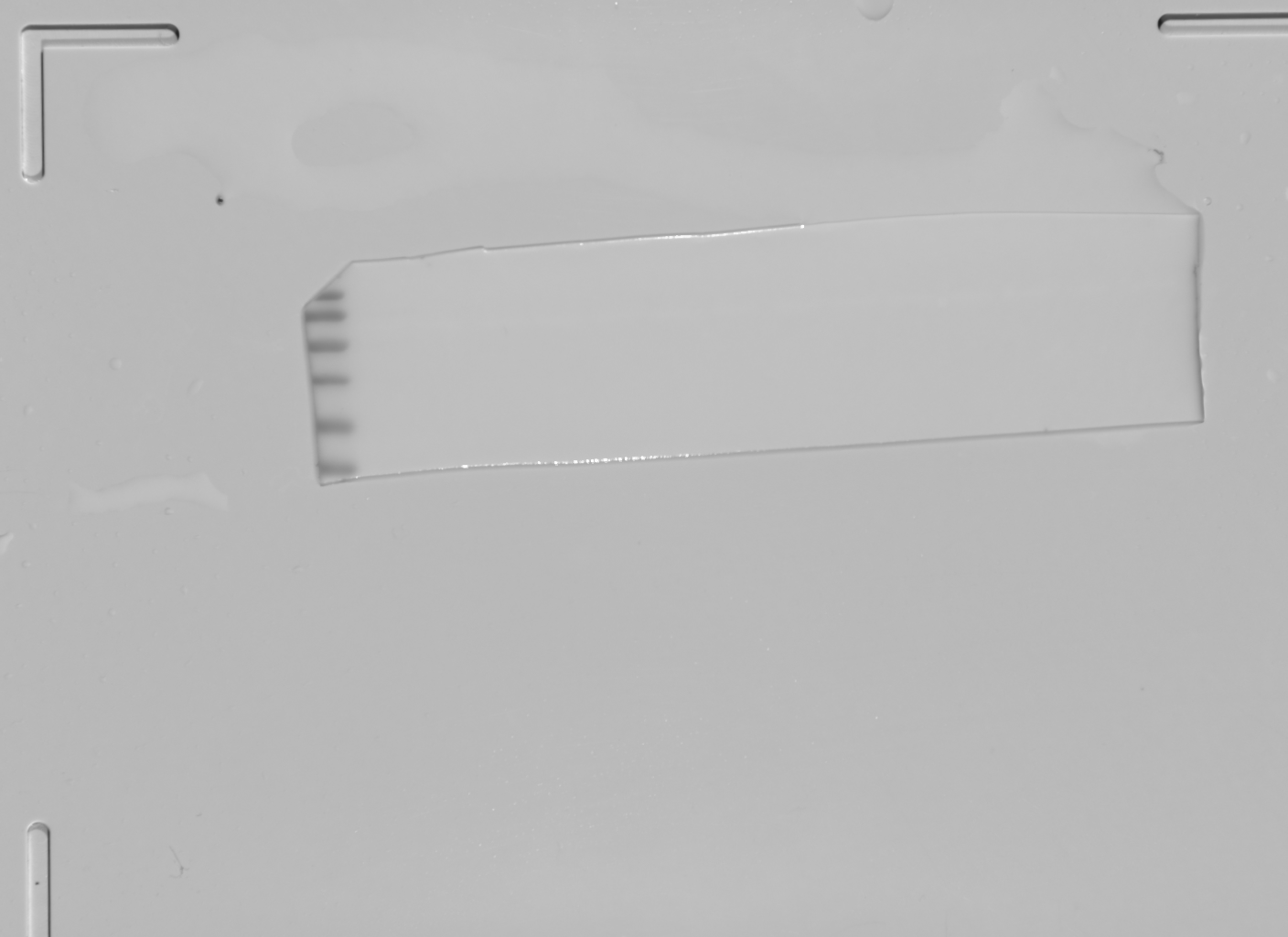

Supplement: Supplementary file 1 — Supplementary Material 1. [file 12985_2024_2385_MOESM1_ESM.zip › xuxiaoying WB/RD p62 2021.09.14_17.51.29_Ch/lp62 2021.09.14_17.51.29_Ch-Marker.tif]

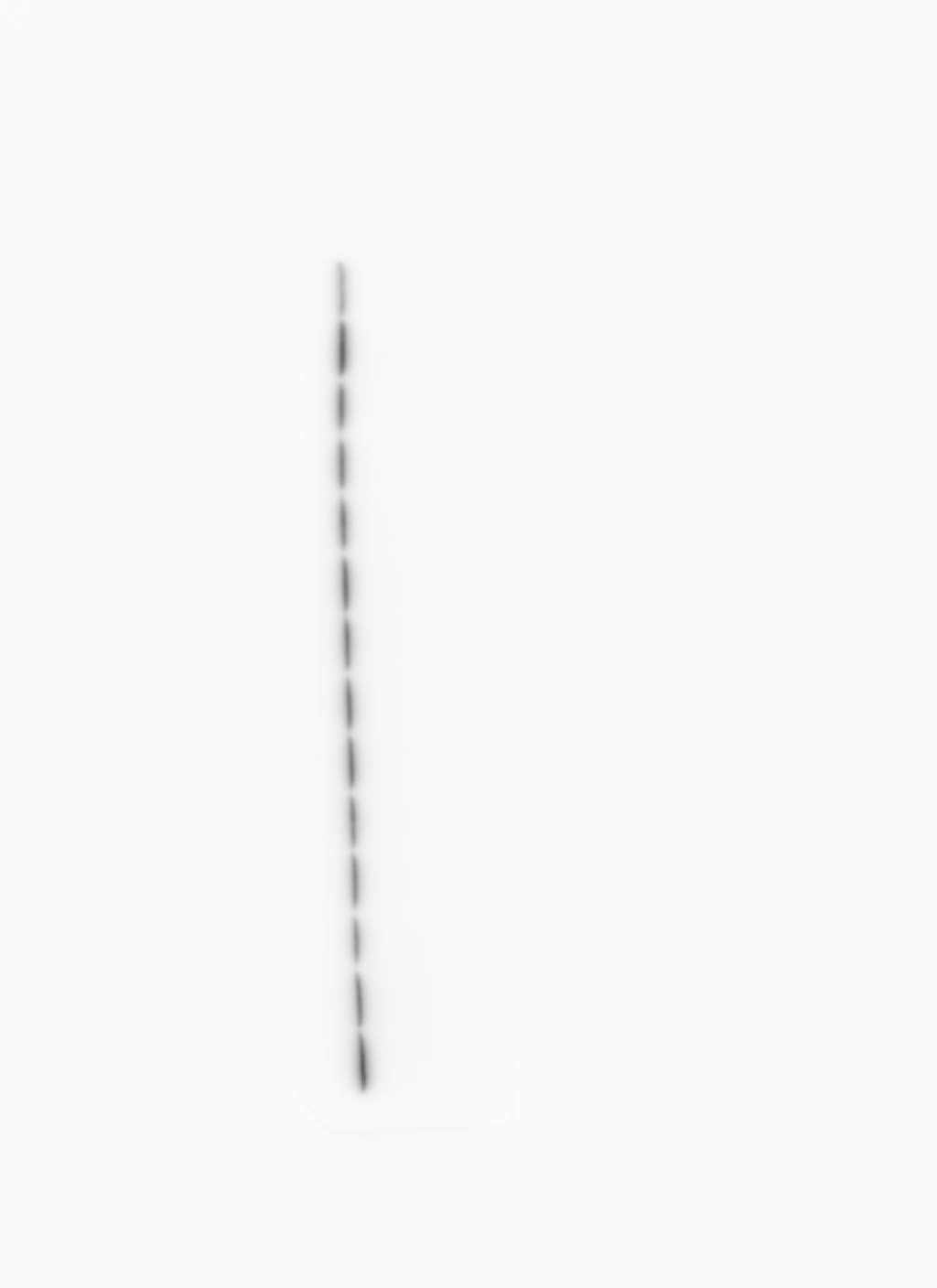

Supplement: Supplementary file 1 — Supplementary Material 1. [file 12985_2024_2385_MOESM1_ESM.zip › xuxiaoying WB/RD p-akt 2021.11.18_23.05.54_Ch/p-akt 2021.11.18_23.05.54_Ch.tif]

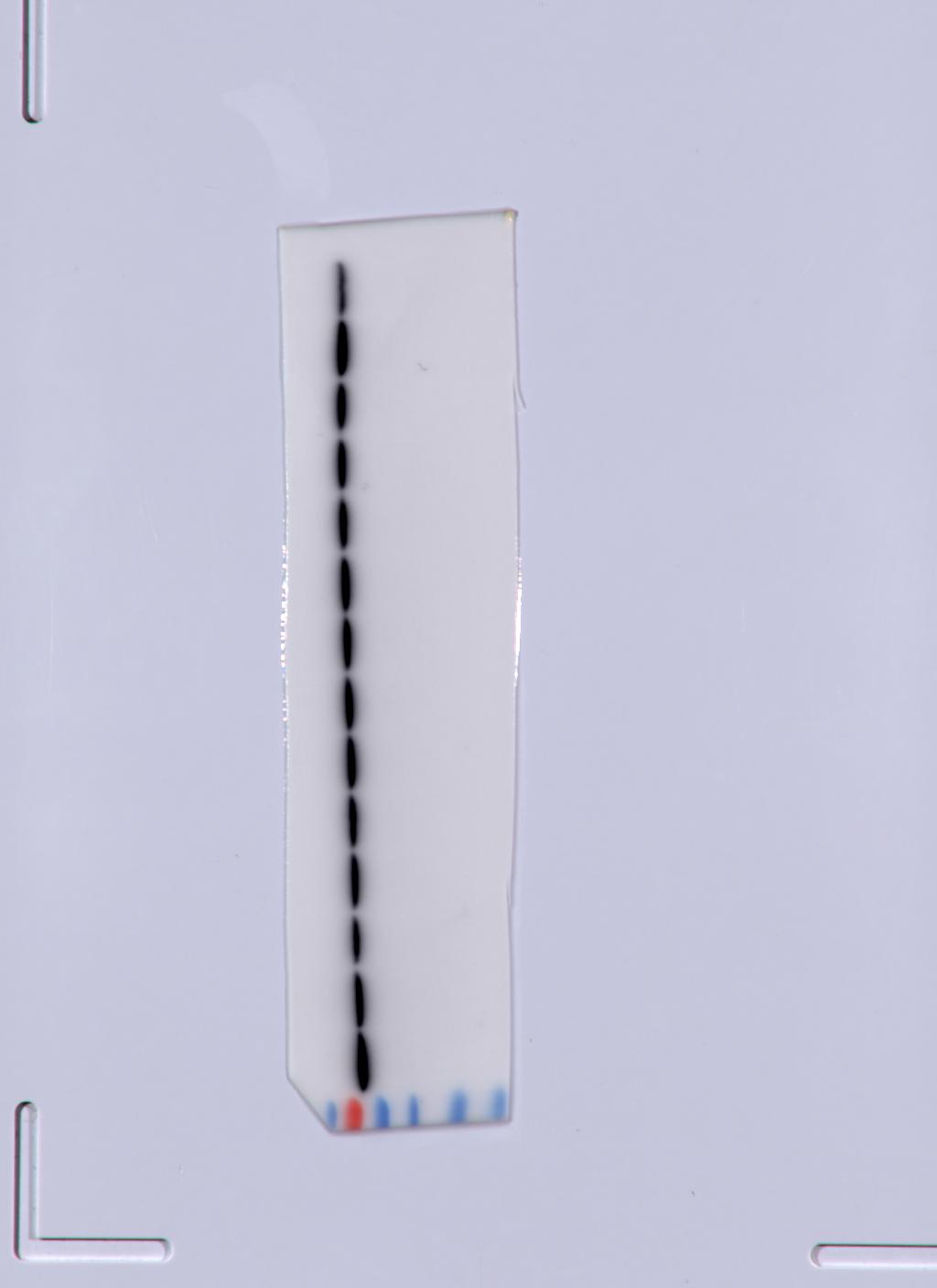

Supplement: Supplementary file 1 — Supplementary Material 1. [file 12985_2024_2385_MOESM1_ESM.zip › xuxiaoying WB/RD p-akt 2021.11.18_23.05.54_Ch/p-akt 2021.11.18_23.05.54_Ch+Marker.jpg]

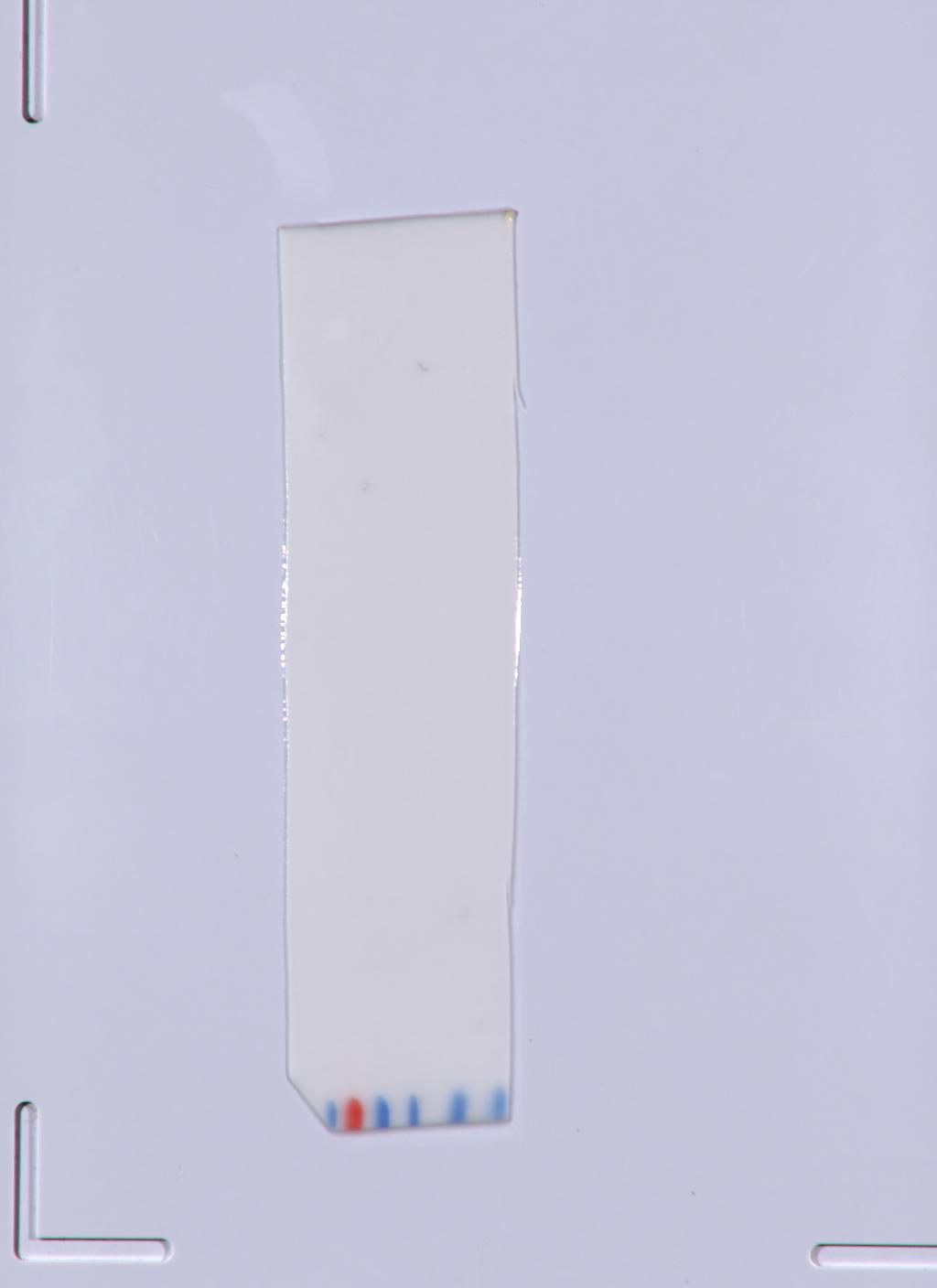

Supplement: Supplementary file 1 — Supplementary Material 1. [file 12985_2024_2385_MOESM1_ESM.zip › xuxiaoying WB/RD p-akt 2021.11.18_23.05.54_Ch/p-akt 2021.11.18_23.05.54_Ch-Marker.jpg]

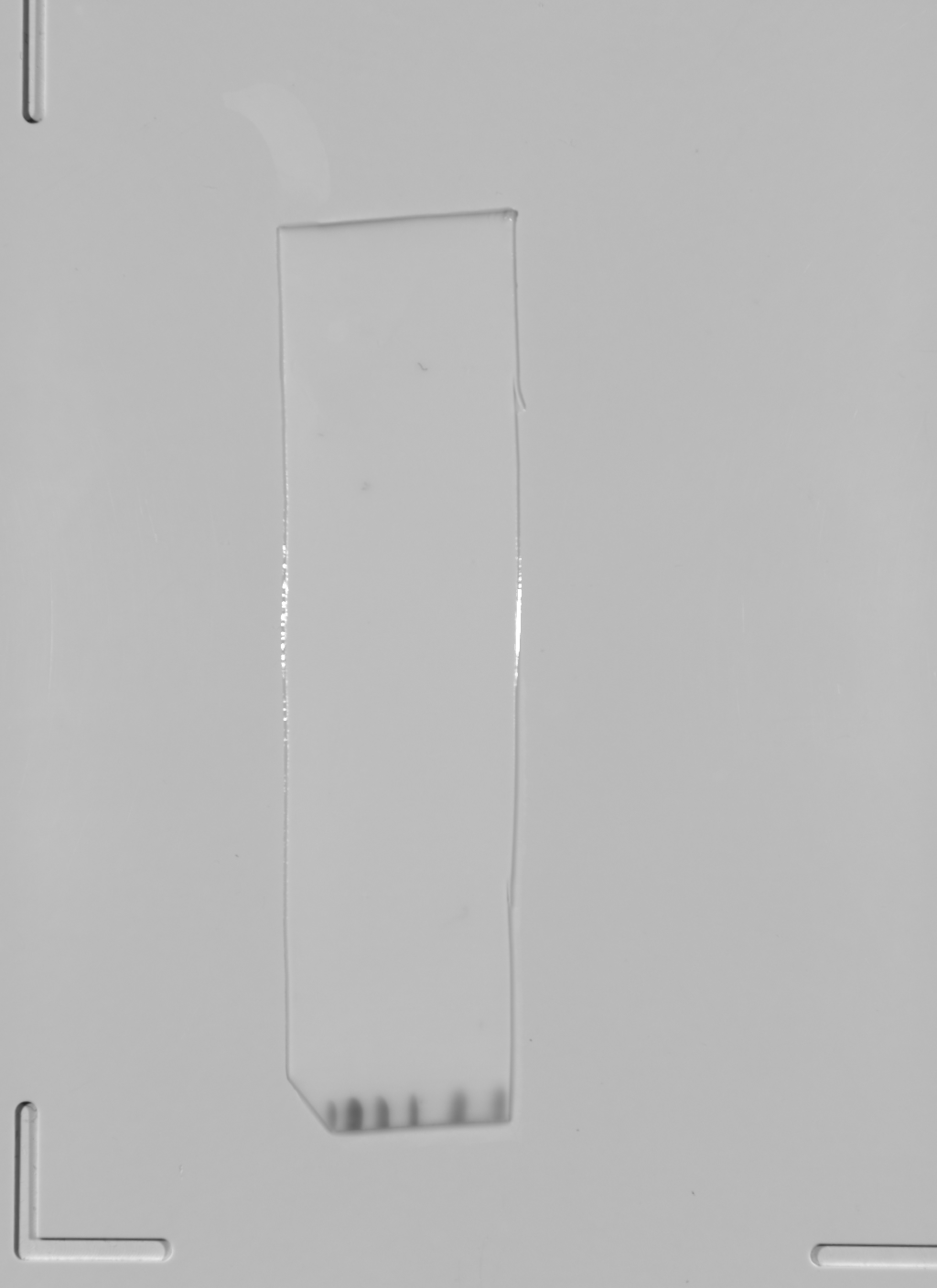

Supplement: Supplementary file 1 — Supplementary Material 1. [file 12985_2024_2385_MOESM1_ESM.zip › xuxiaoying WB/RD p-akt 2021.11.18_23.05.54_Ch/p-akt 2021.11.18_23.05.54_Ch-Marker.tif]

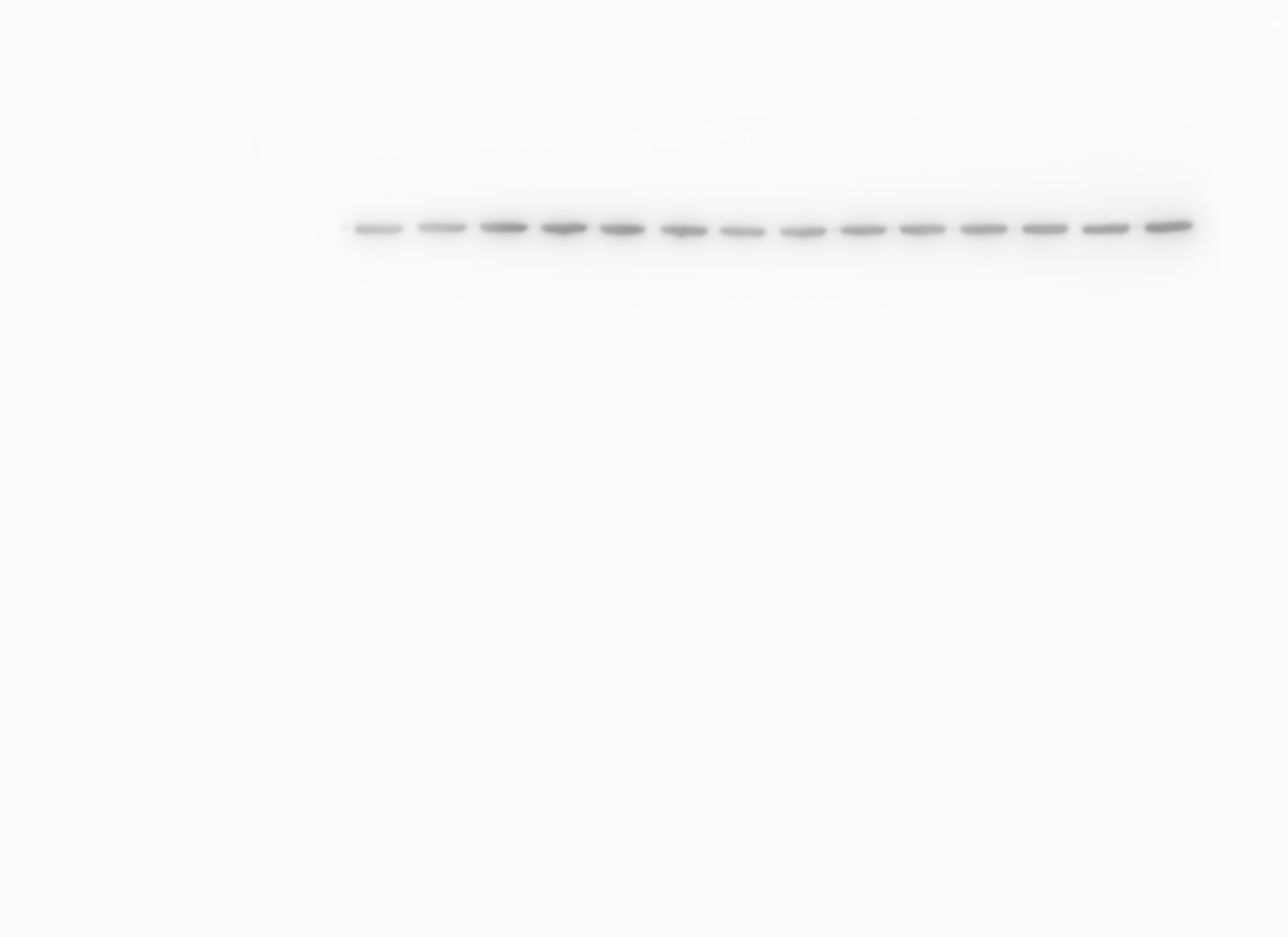

Supplement: Supplementary file 1 — Supplementary Material 1. [file 12985_2024_2385_MOESM1_ESM.zip › xuxiaoying WB/RD p-akt b-actin 7 2021.09.14_17.16.00_Ch/b-actin 7 2021.09.14_17.16.00_Ch.tif]

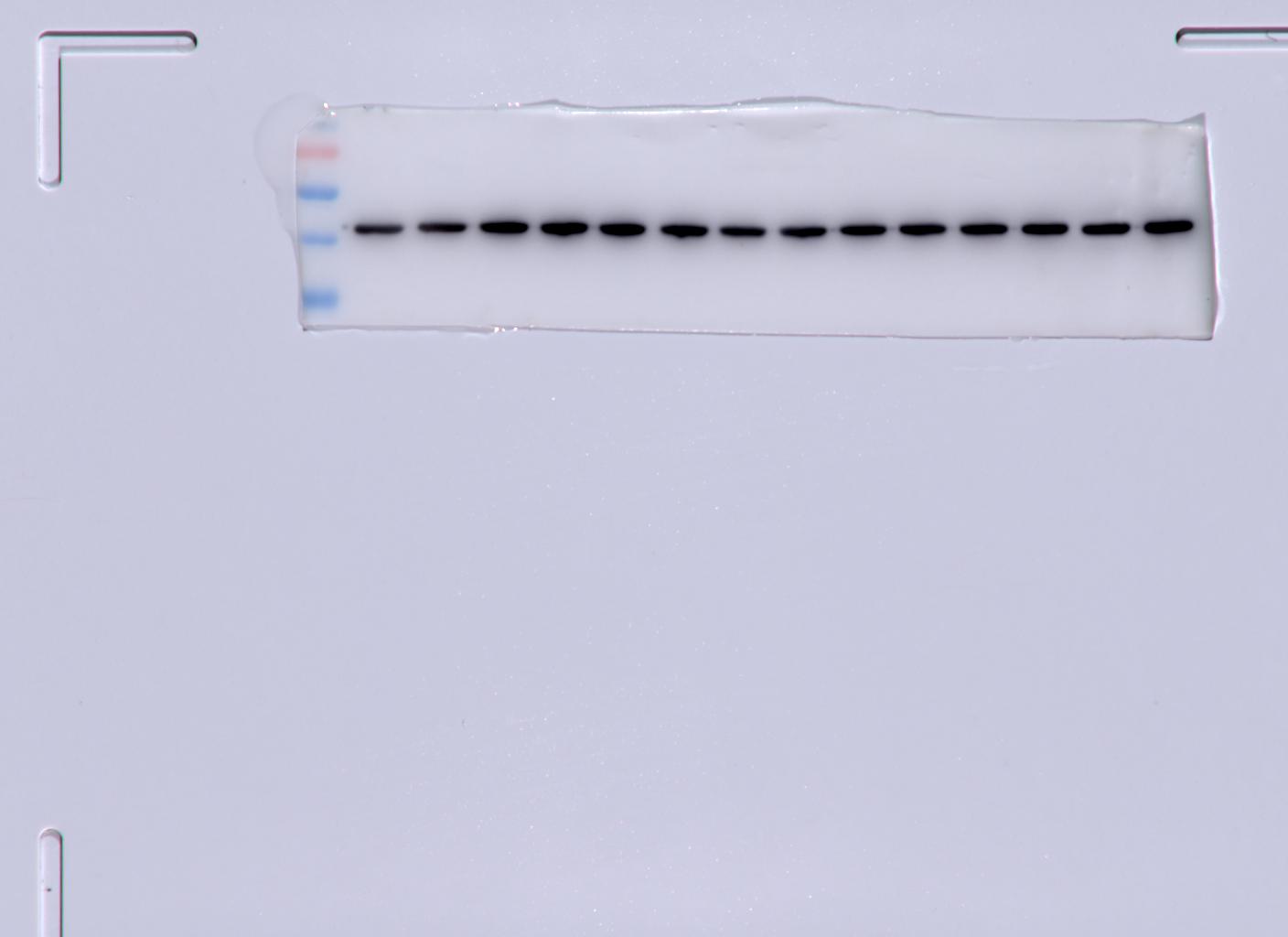

Supplement: Supplementary file 1 — Supplementary Material 1. [file 12985_2024_2385_MOESM1_ESM.zip › xuxiaoying WB/RD p-akt b-actin 7 2021.09.14_17.16.00_Ch/b-actin 7 2021.09.14_17.16.00_Ch+Marker.jpg]

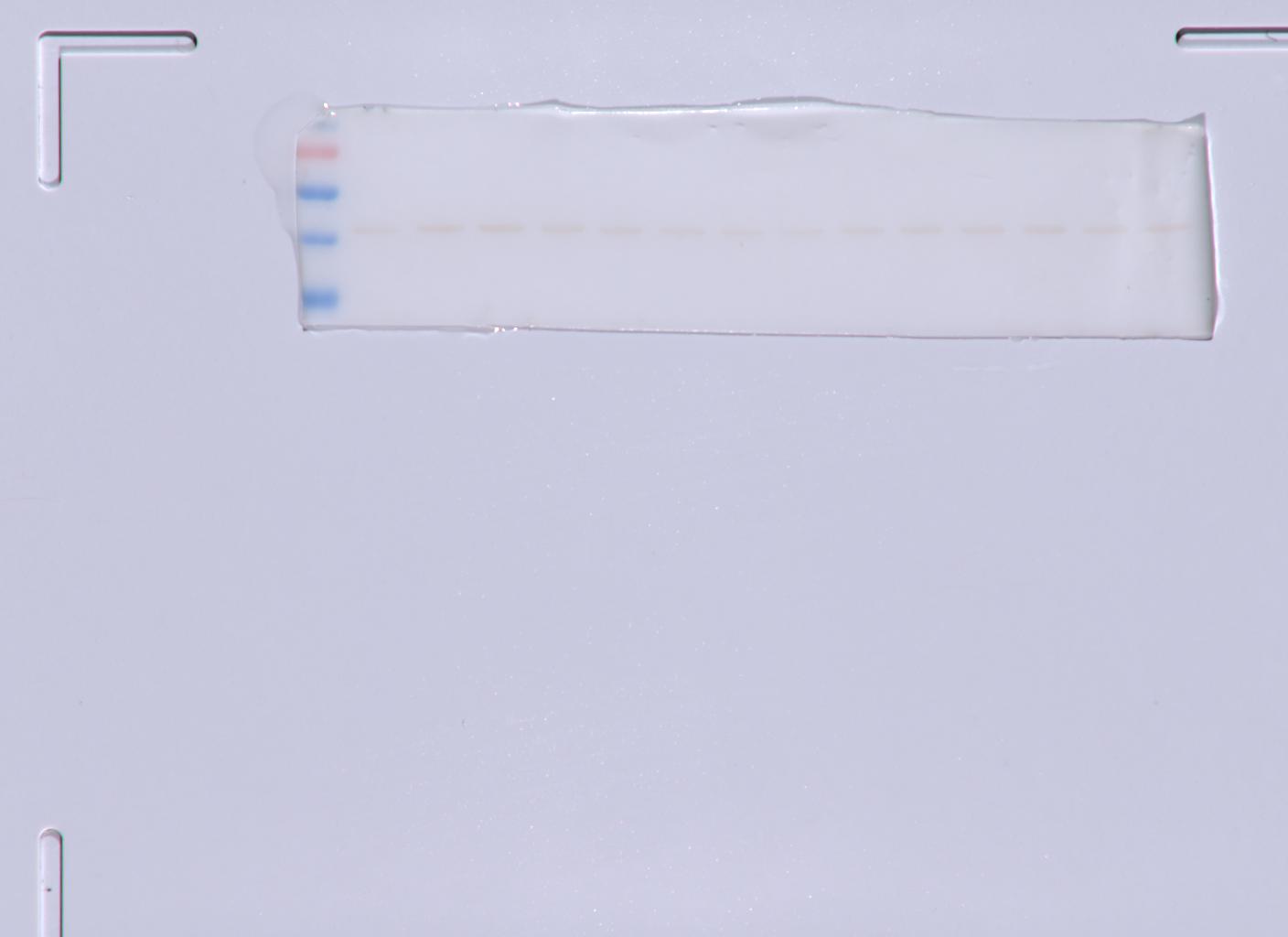

Supplement: Supplementary file 1 — Supplementary Material 1. [file 12985_2024_2385_MOESM1_ESM.zip › xuxiaoying WB/RD p-akt b-actin 7 2021.09.14_17.16.00_Ch/b-actin 7 2021.09.14_17.16.00_Ch-Marker.jpg]

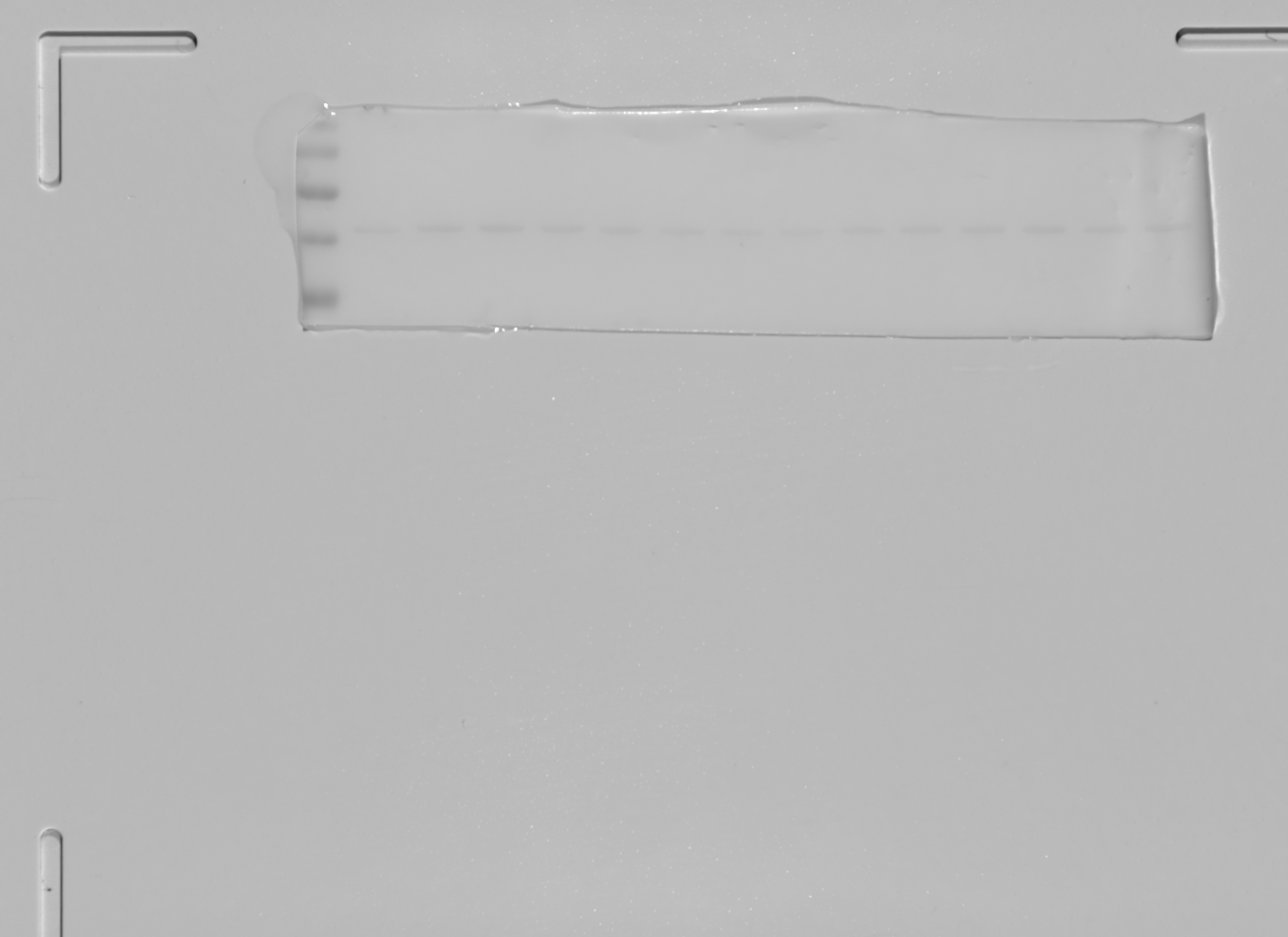

Supplement: Supplementary file 1 — Supplementary Material 1. [file 12985_2024_2385_MOESM1_ESM.zip › xuxiaoying WB/RD p-akt b-actin 7 2021.09.14_17.16.00_Ch/b-actin 7 2021.09.14_17.16.00_Ch-Marker.tif]

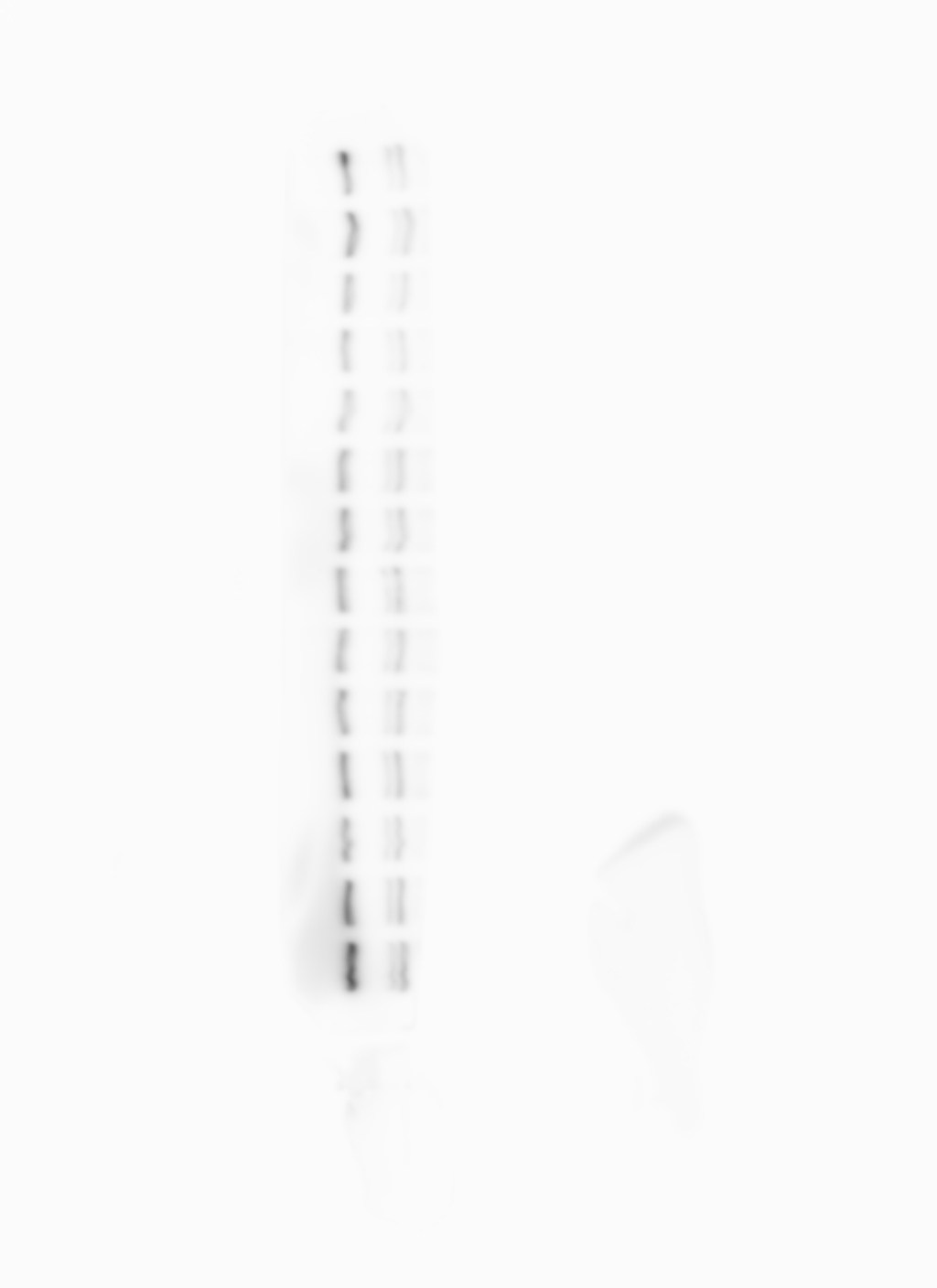

Supplement: Supplementary file 1 — Supplementary Material 1. [file 12985_2024_2385_MOESM1_ESM.zip › xuxiaoying WB/RD p-mtor 2021.11.18_22.45.20_Ch/p-mtor 2021.11.18_22.45.20_Ch.tif]

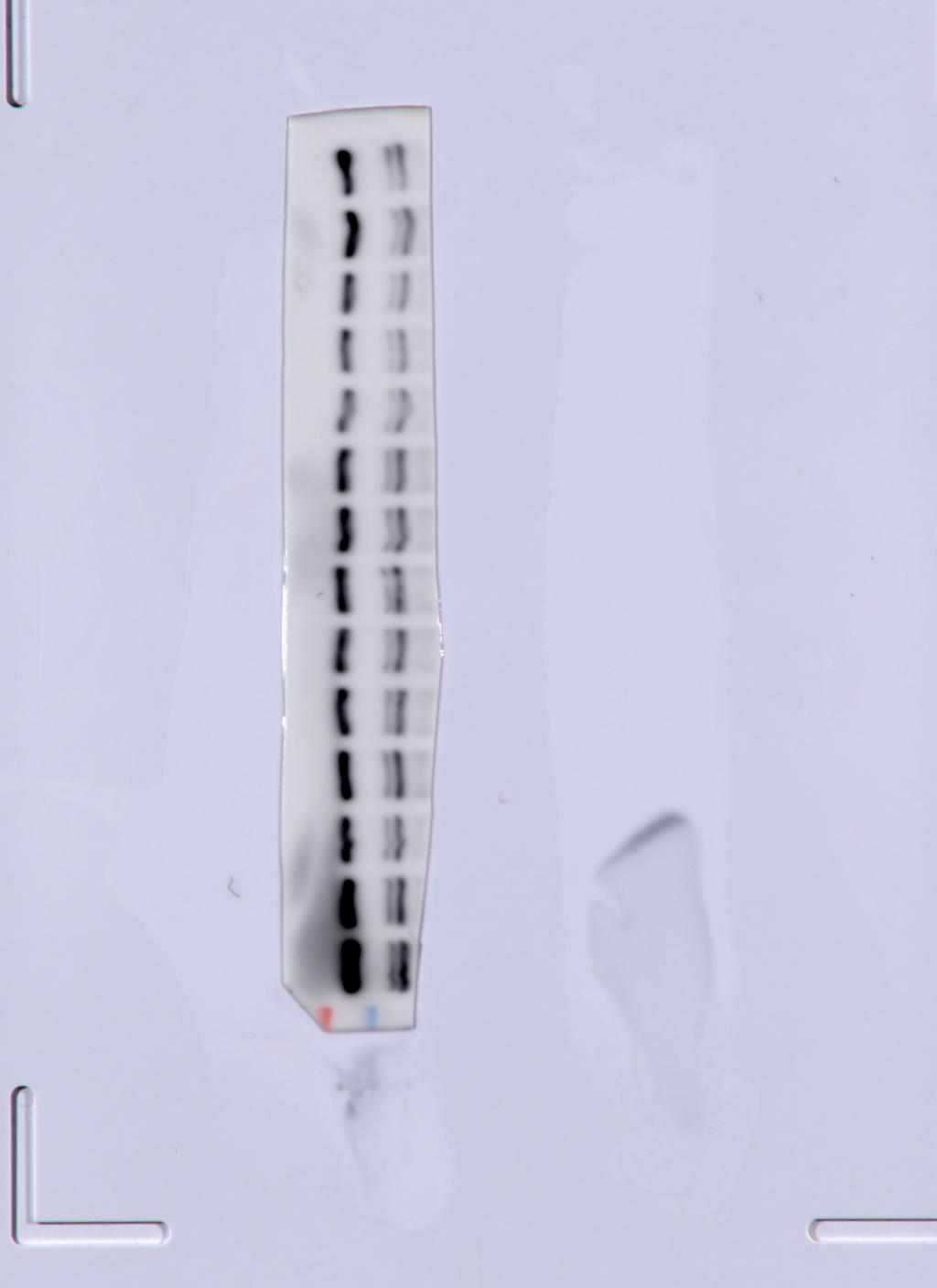

Supplement: Supplementary file 1 — Supplementary Material 1. [file 12985_2024_2385_MOESM1_ESM.zip › xuxiaoying WB/RD p-mtor 2021.11.18_22.45.20_Ch/p-mtor 2021.11.18_22.45.20_Ch+Marker.jpg]

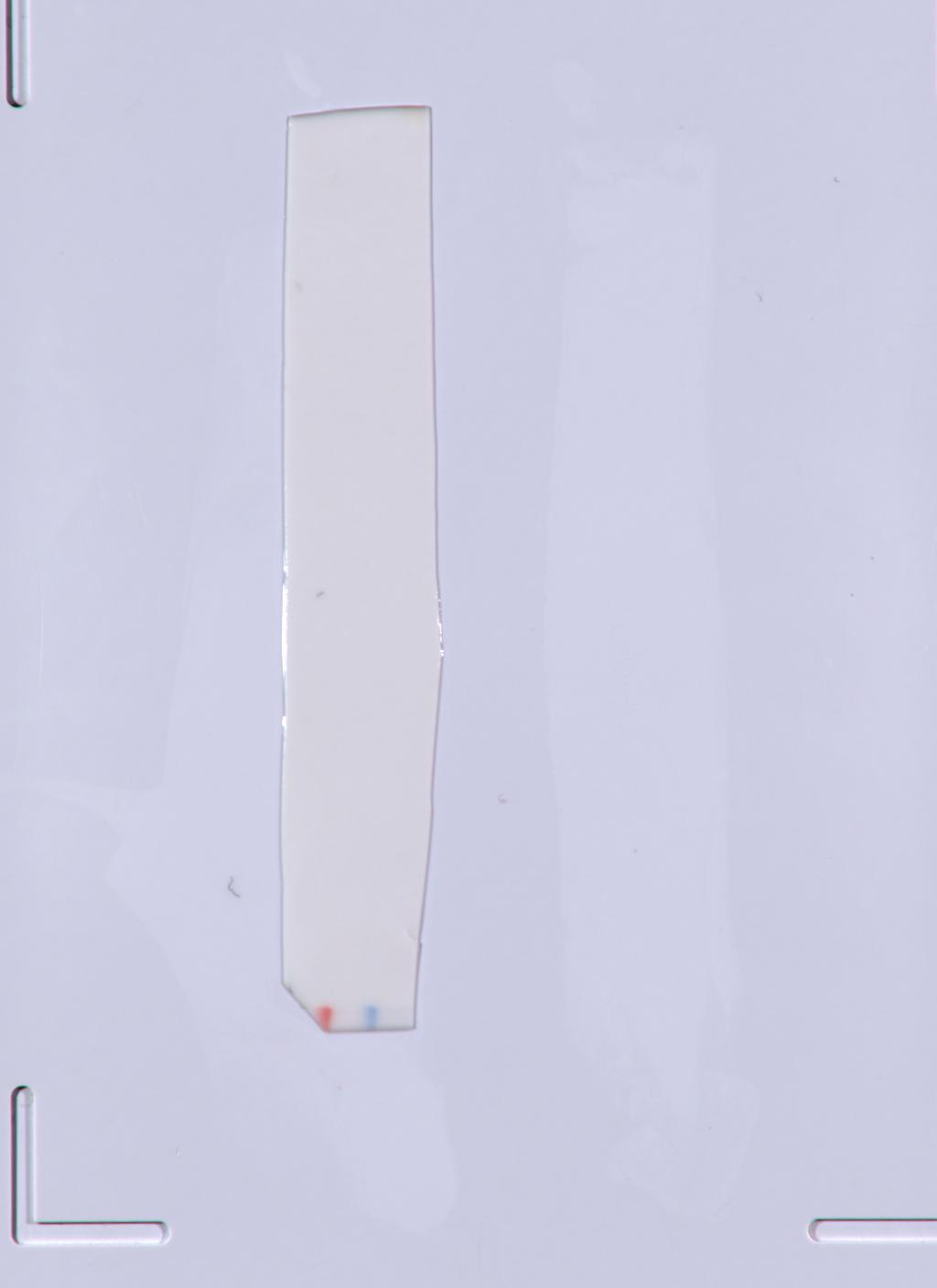

Supplement: Supplementary file 1 — Supplementary Material 1. [file 12985_2024_2385_MOESM1_ESM.zip › xuxiaoying WB/RD p-mtor 2021.11.18_22.45.20_Ch/p-mtor 2021.11.18_22.45.20_Ch-Marker.jpg]

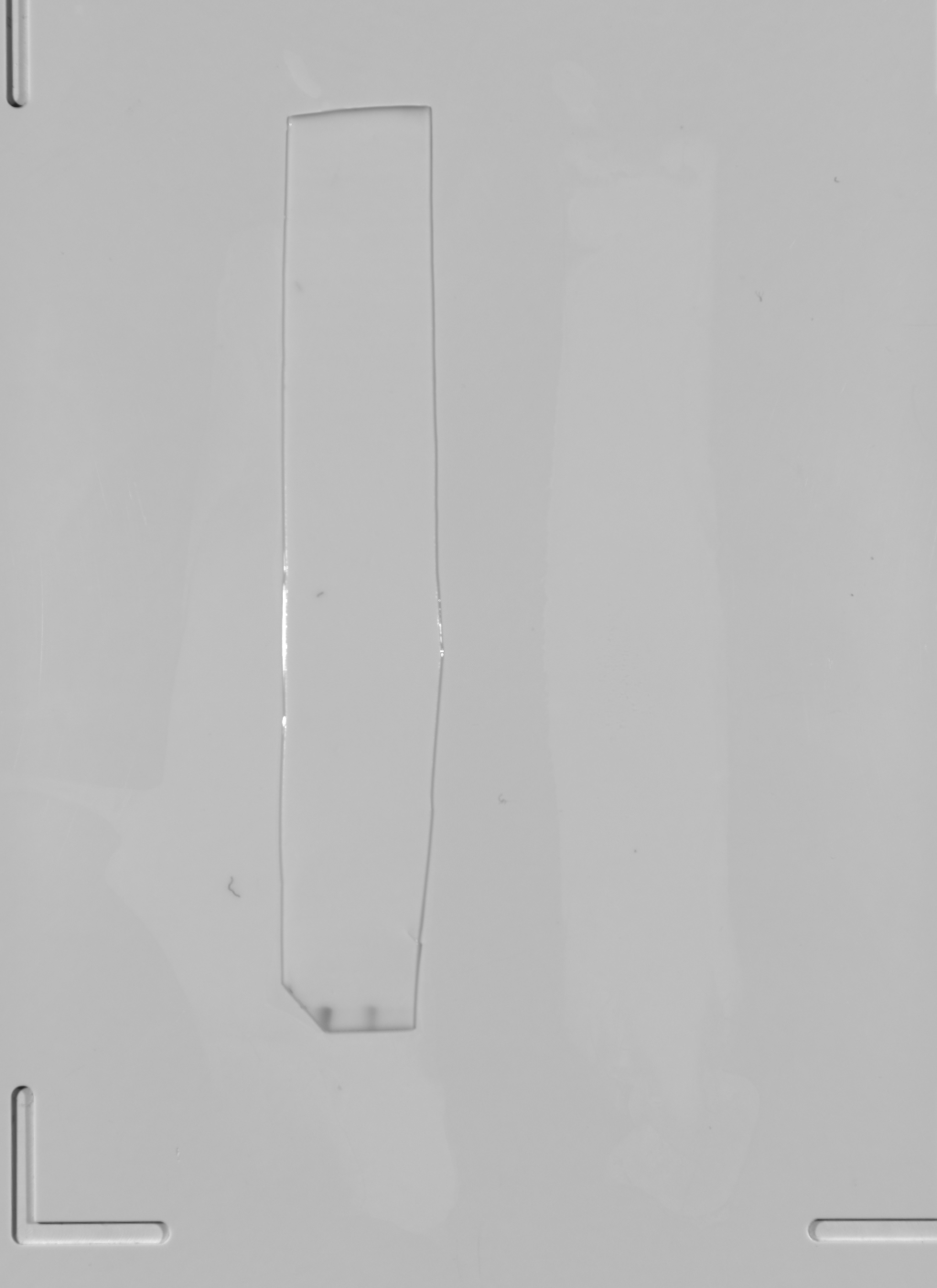

Supplement: Supplementary file 1 — Supplementary Material 1. [file 12985_2024_2385_MOESM1_ESM.zip › xuxiaoying WB/RD p-mtor 2021.11.18_22.45.20_Ch/p-mtor 2021.11.18_22.45.20_Ch-Marker.tif]

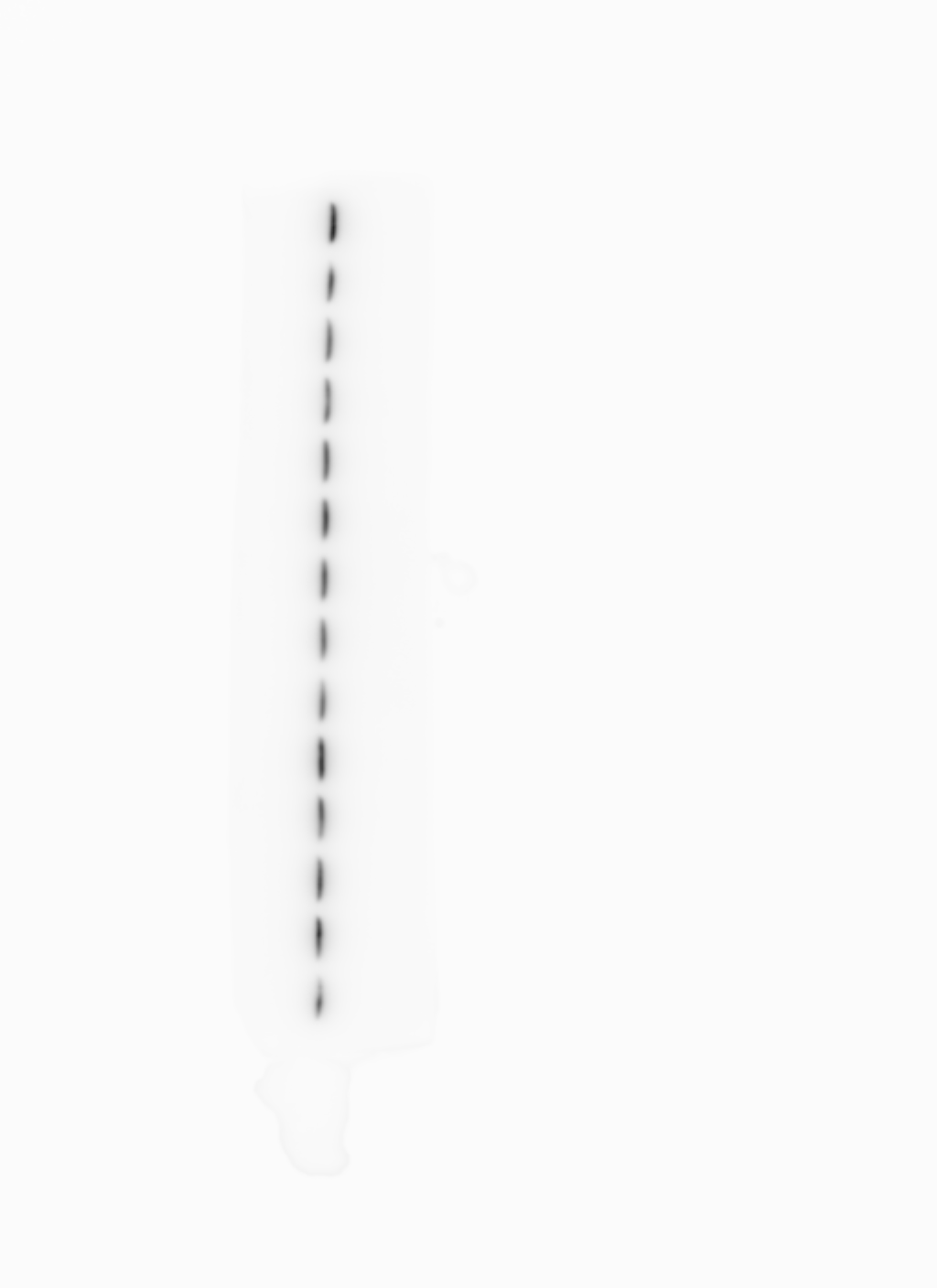

Supplement: Supplementary file 1 — Supplementary Material 1. [file 12985_2024_2385_MOESM1_ESM.zip › xuxiaoying WB/RD p-mTOR b-actin2 2021.11.15_21.37.22_Ch/nc2 2021.11.15_21.37.22_Ch.tif]

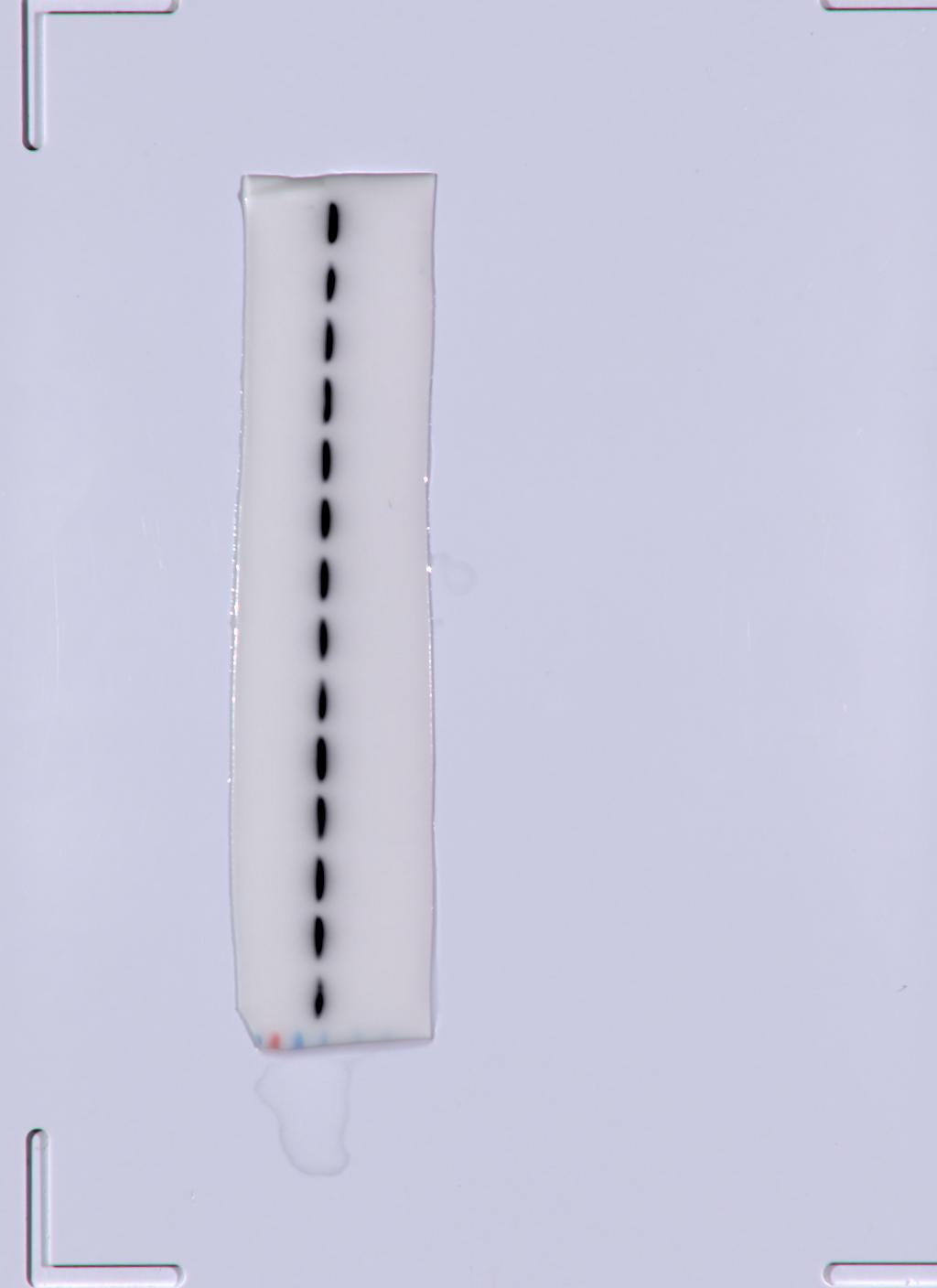

Supplement: Supplementary file 1 — Supplementary Material 1. [file 12985_2024_2385_MOESM1_ESM.zip › xuxiaoying WB/RD p-mTOR b-actin2 2021.11.15_21.37.22_Ch/nc2 2021.11.15_21.37.22_Ch+Marker.jpg]

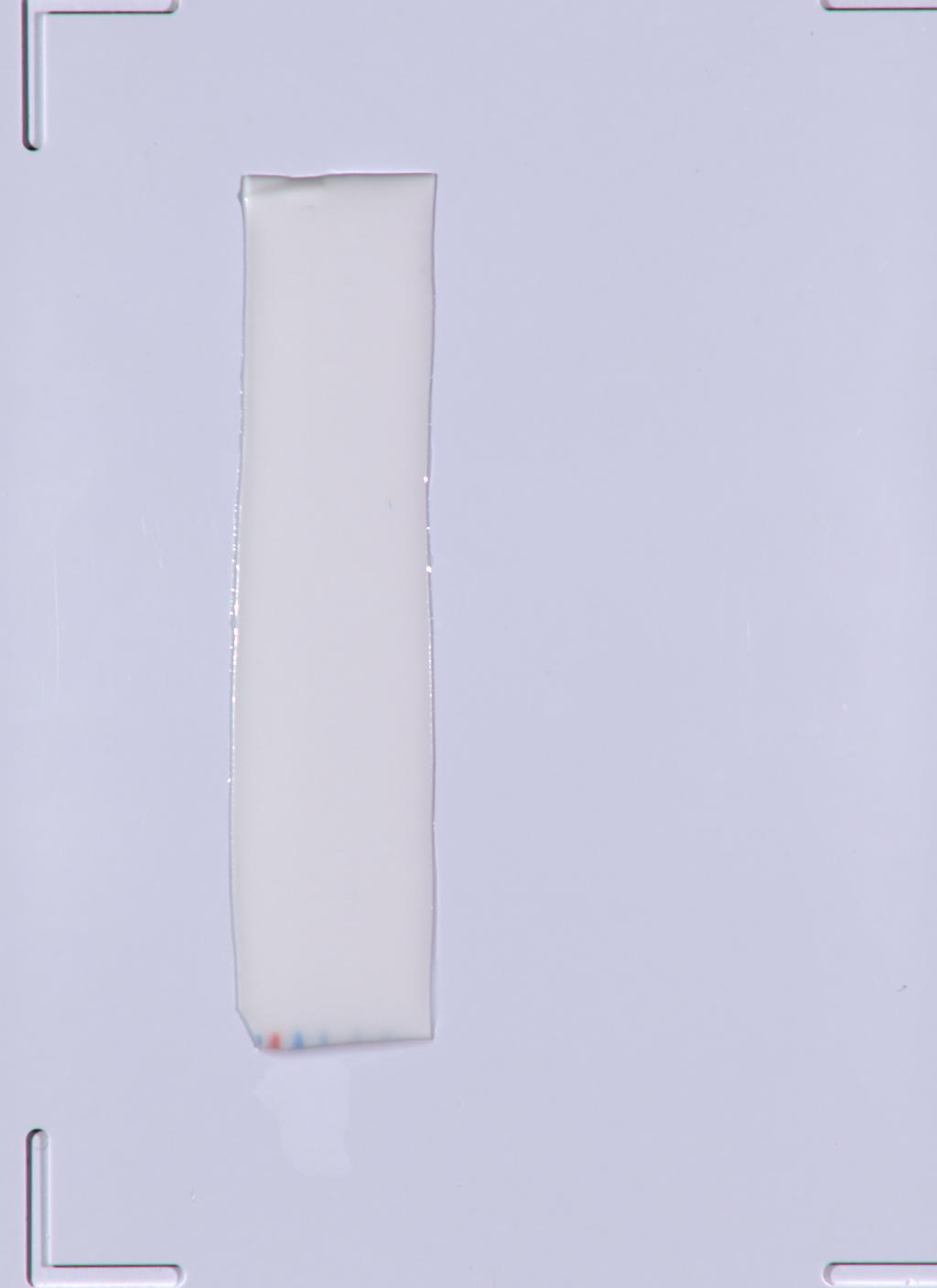

Supplement: Supplementary file 1 — Supplementary Material 1. [file 12985_2024_2385_MOESM1_ESM.zip › xuxiaoying WB/RD p-mTOR b-actin2 2021.11.15_21.37.22_Ch/nc2 2021.11.15_21.37.22_Ch-Marker.jpg]

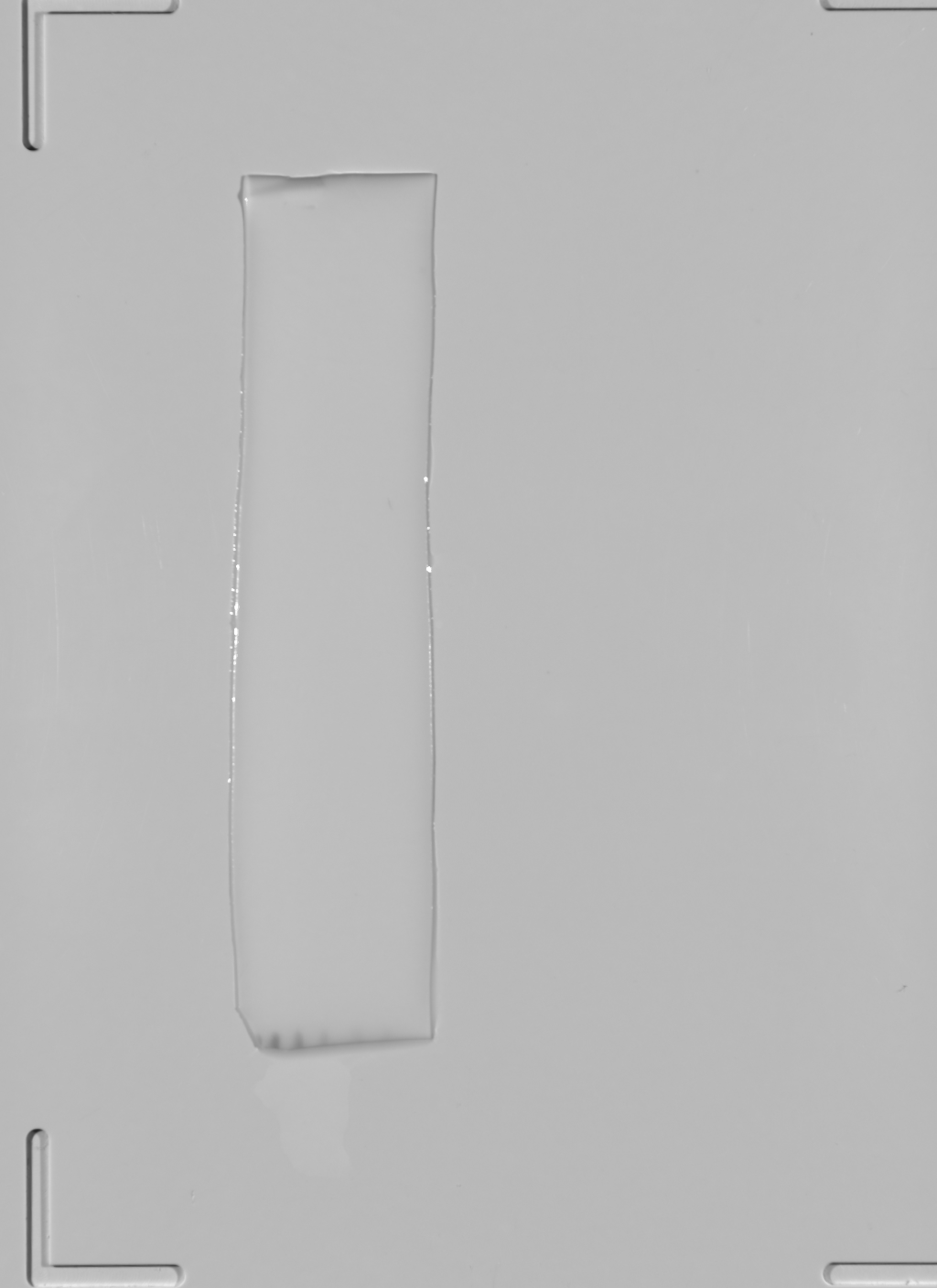

Supplement: Supplementary file 1 — Supplementary Material 1. [file 12985_2024_2385_MOESM1_ESM.zip › xuxiaoying WB/RD p-mTOR b-actin2 2021.11.15_21.37.22_Ch/nc2 2021.11.15_21.37.22_Ch-Marker.tif]

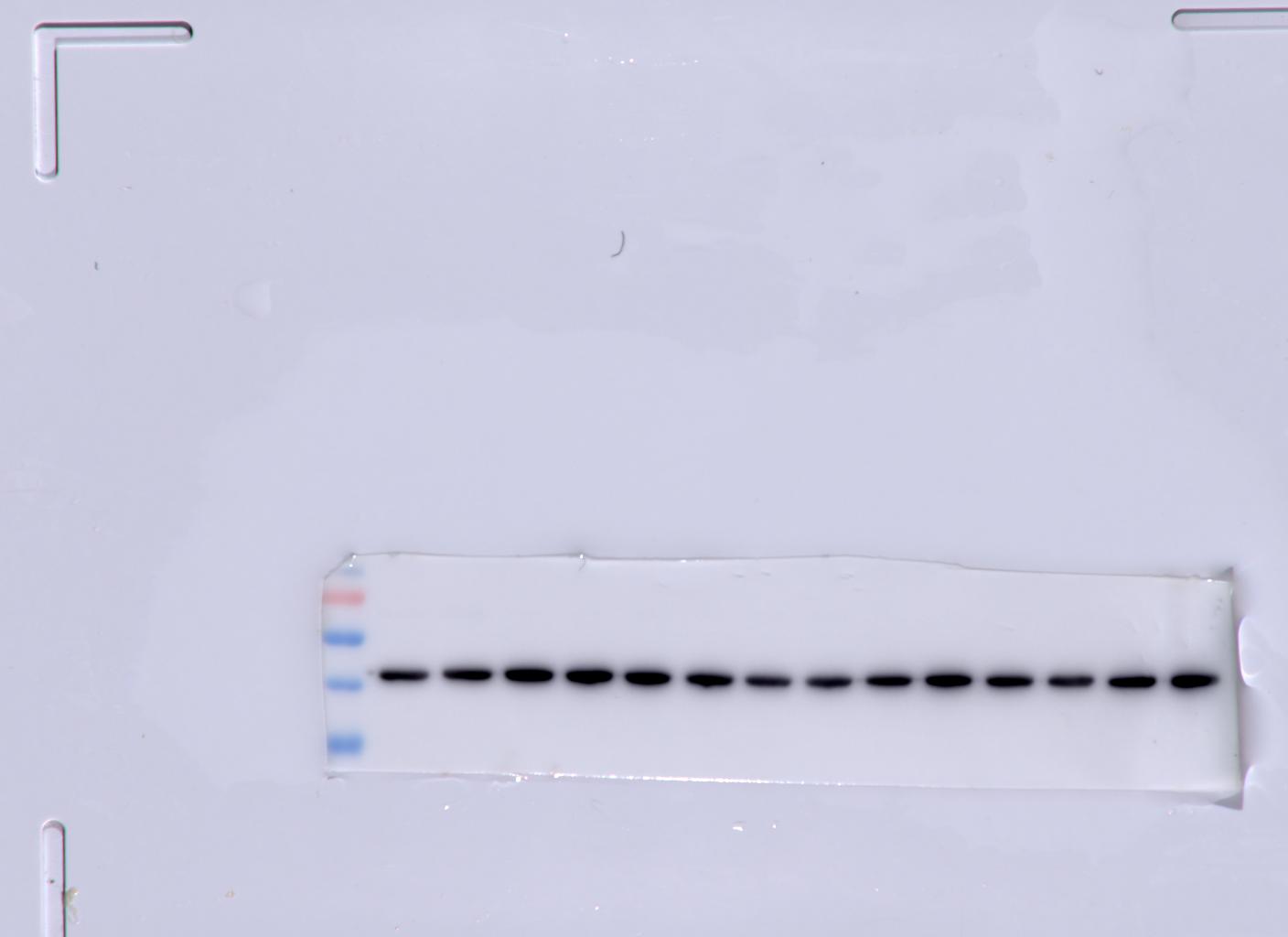

Supplement: Supplementary file 1 — Supplementary Material 1. [file 12985_2024_2385_MOESM1_ESM.zip › xuxiaoying WB/SH-SY5Y p-akt b-actin 6 2021.09.14_17.09.34_Ch/b-actin 6 2021.09.14_17.09.34_Ch+Marker.jpg]

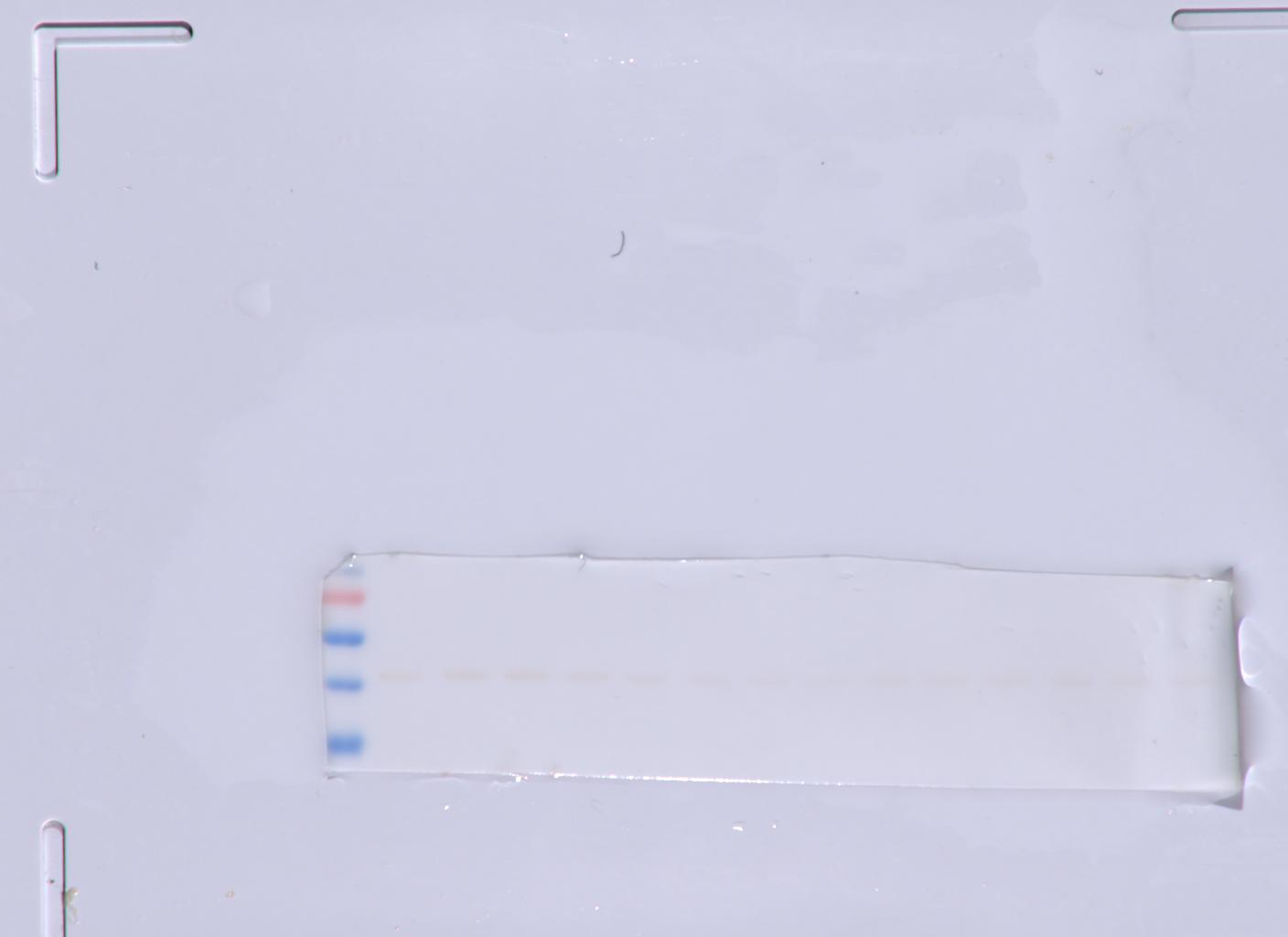

Supplement: Supplementary file 1 — Supplementary Material 1. [file 12985_2024_2385_MOESM1_ESM.zip › xuxiaoying WB/SH-SY5Y p-akt b-actin 6 2021.09.14_17.09.34_Ch/b-actin 6 2021.09.14_17.09.34_Ch-Marker.jpg]

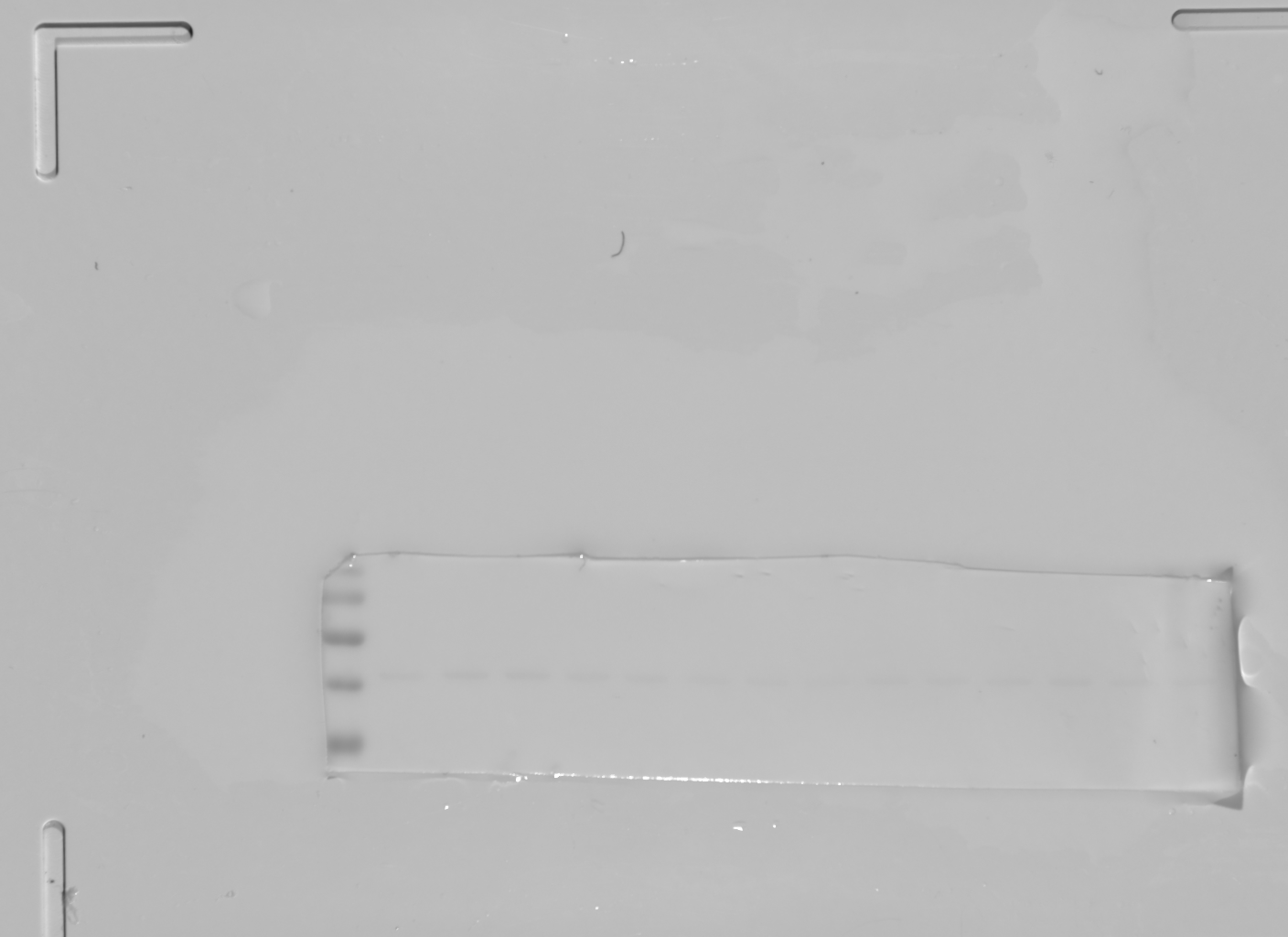

Supplement: Supplementary file 1 — Supplementary Material 1. [file 12985_2024_2385_MOESM1_ESM.zip › xuxiaoying WB/SH-SY5Y p-akt b-actin 6 2021.09.14_17.09.34_Ch/b-actin 6 2021.09.14_17.09.34_Ch-Marker.tif]

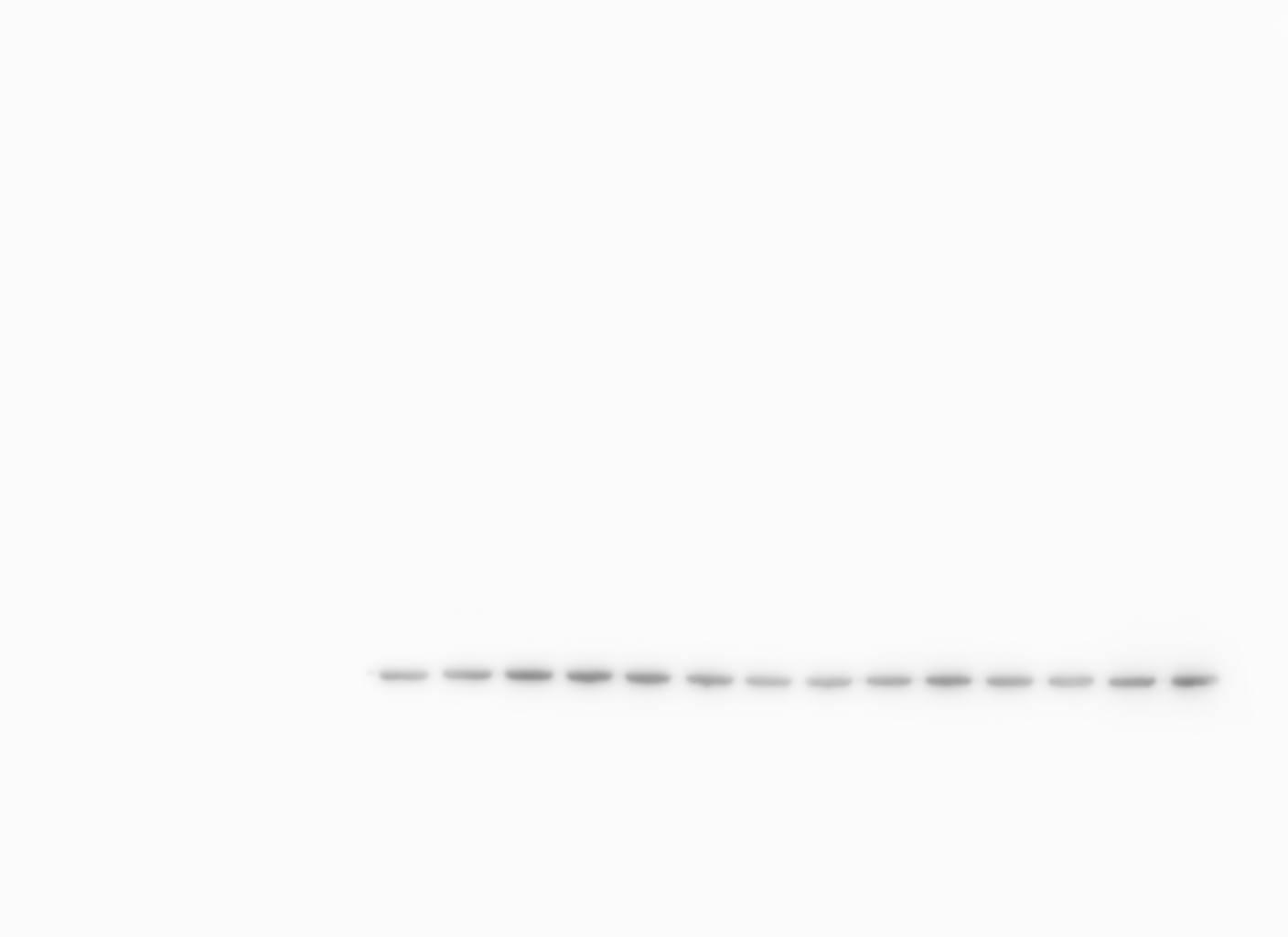

Supplement: Supplementary file 1 — Supplementary Material 1. [file 12985_2024_2385_MOESM1_ESM.zip › xuxiaoying WB/SH-SY5Y p-akt b-actin 6 2021.09.14_17.09.34_Ch/PAKTb-actin 6 2021.09.14_17.09.34_Ch.tif]

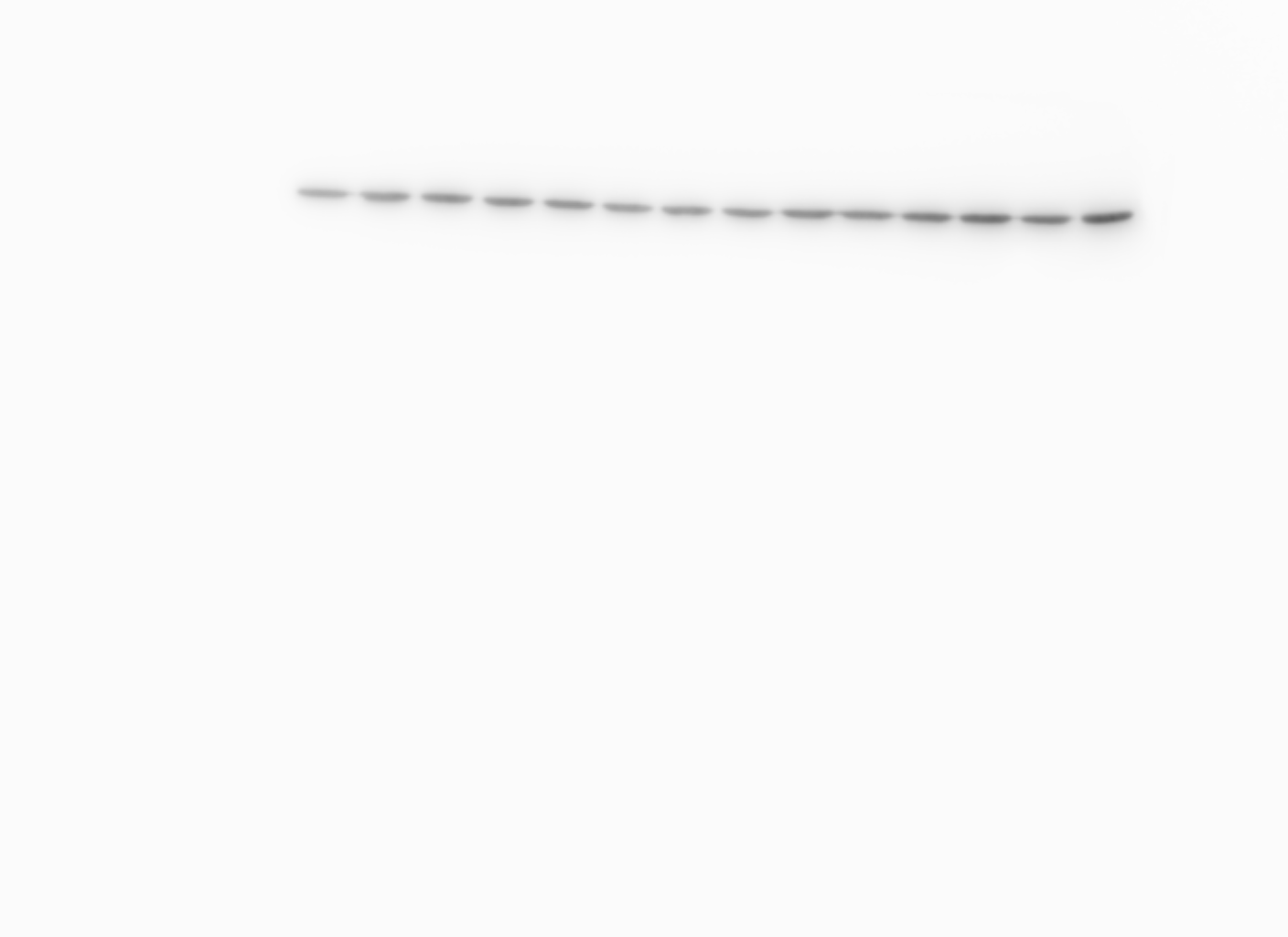

Supplement: Supplementary file 1 — Supplementary Material 1. [file 12985_2024_2385_MOESM1_ESM.zip › xuxiaoying WB/SH-SY5Y AKT b-actin 2021.09.14_18.02.05_Ch/nc 2021.09.14_18.02.05_Ch.tif]

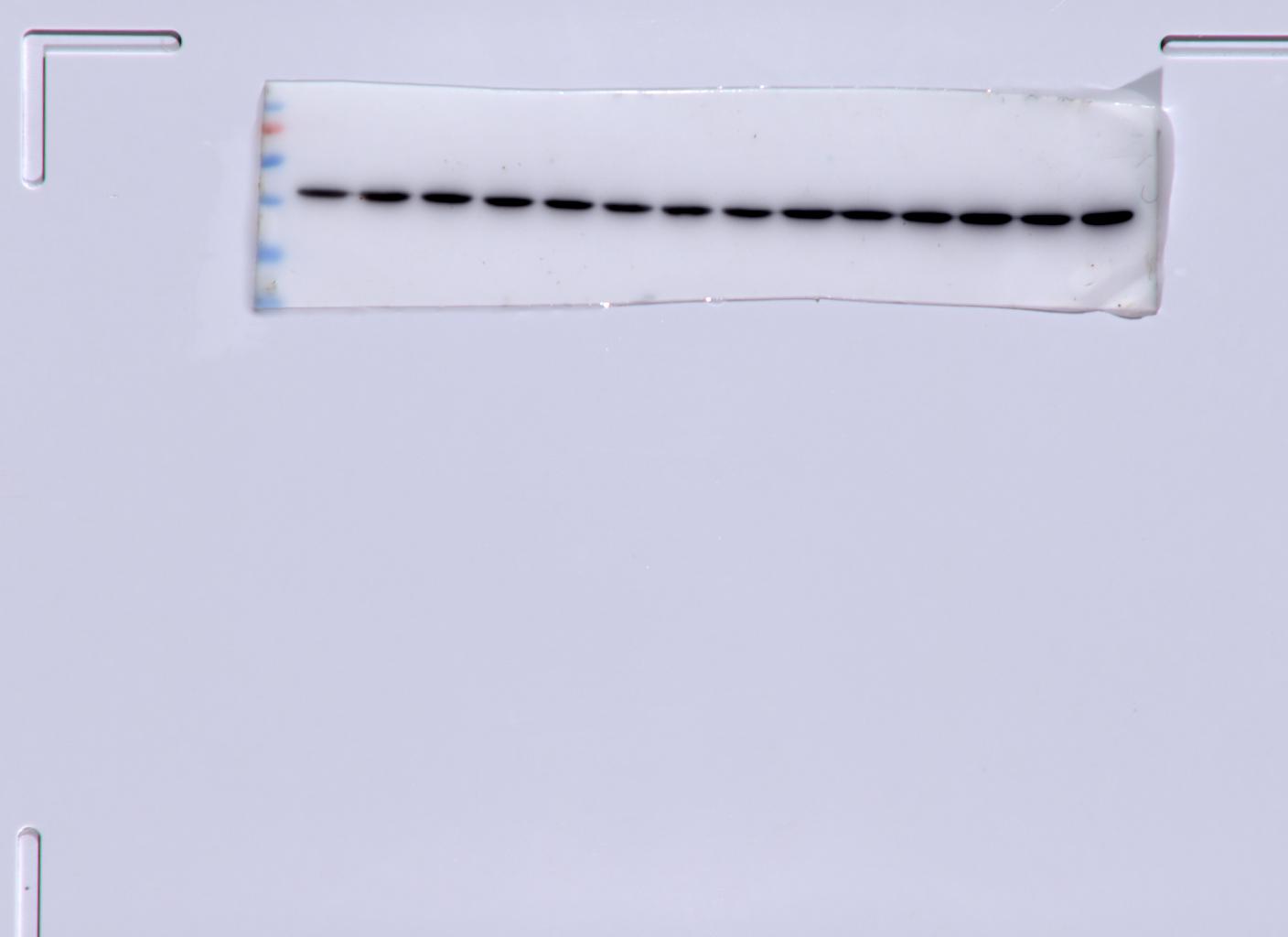

Supplement: Supplementary file 1 — Supplementary Material 1. [file 12985_2024_2385_MOESM1_ESM.zip › xuxiaoying WB/SH-SY5Y AKT b-actin 2021.09.14_18.02.05_Ch/nc 2021.09.14_18.02.05_Ch+Marker.jpg]

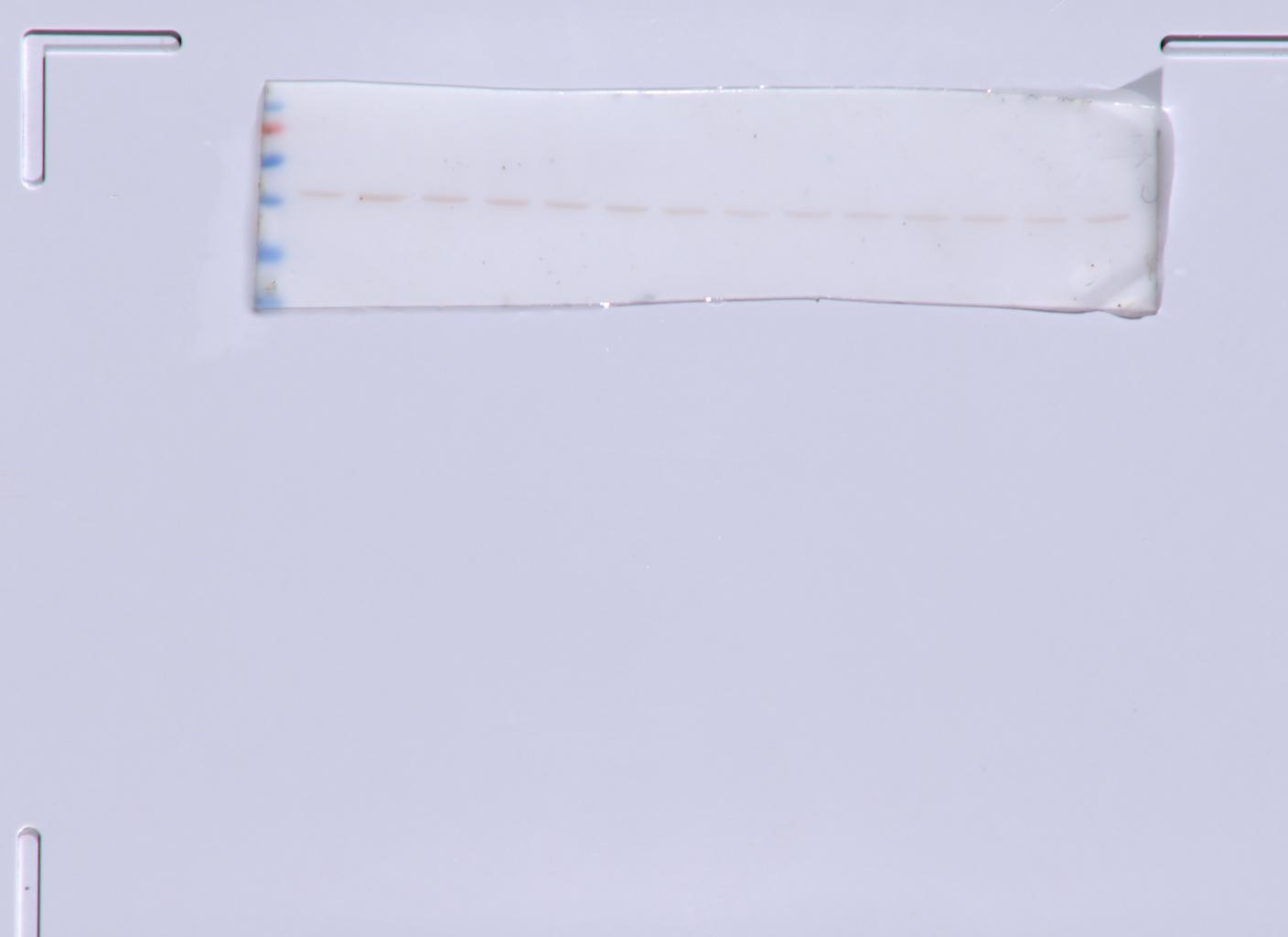

Supplement: Supplementary file 1 — Supplementary Material 1. [file 12985_2024_2385_MOESM1_ESM.zip › xuxiaoying WB/SH-SY5Y AKT b-actin 2021.09.14_18.02.05_Ch/nc 2021.09.14_18.02.05_Ch-Marker.jpg]

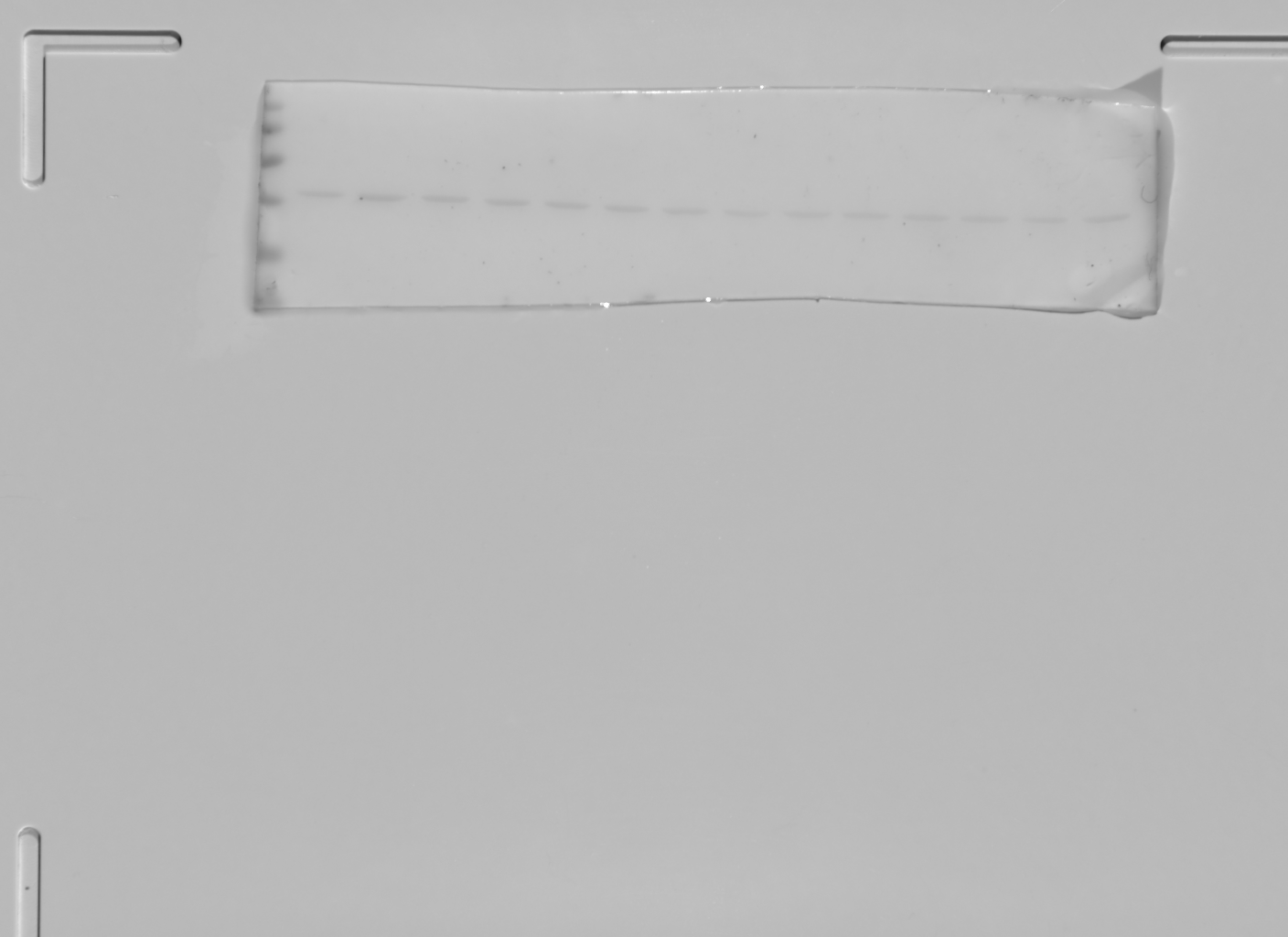

Supplement: Supplementary file 1 — Supplementary Material 1. [file 12985_2024_2385_MOESM1_ESM.zip › xuxiaoying WB/SH-SY5Y AKT b-actin 2021.09.14_18.02.05_Ch/nc 2021.09.14_18.02.05_Ch-Marker.tif]

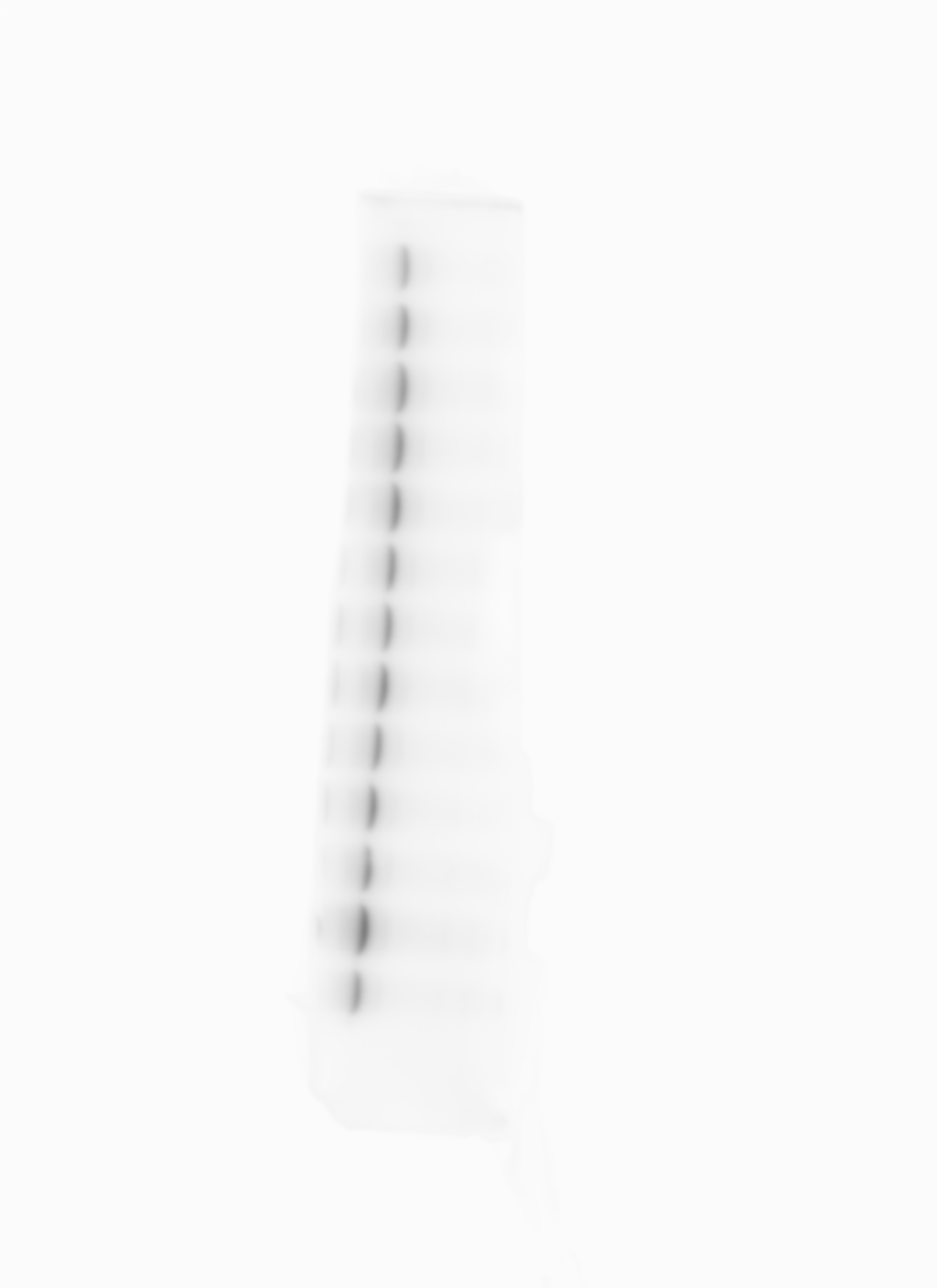

Supplement: Supplementary file 1 — Supplementary Material 1. [file 12985_2024_2385_MOESM1_ESM.zip › xuxiaoying WB/SY5Y 3d2 2021.10.09_16.32.51_Ch/3d2 2021.10.09_16.32.51_Ch.tif]

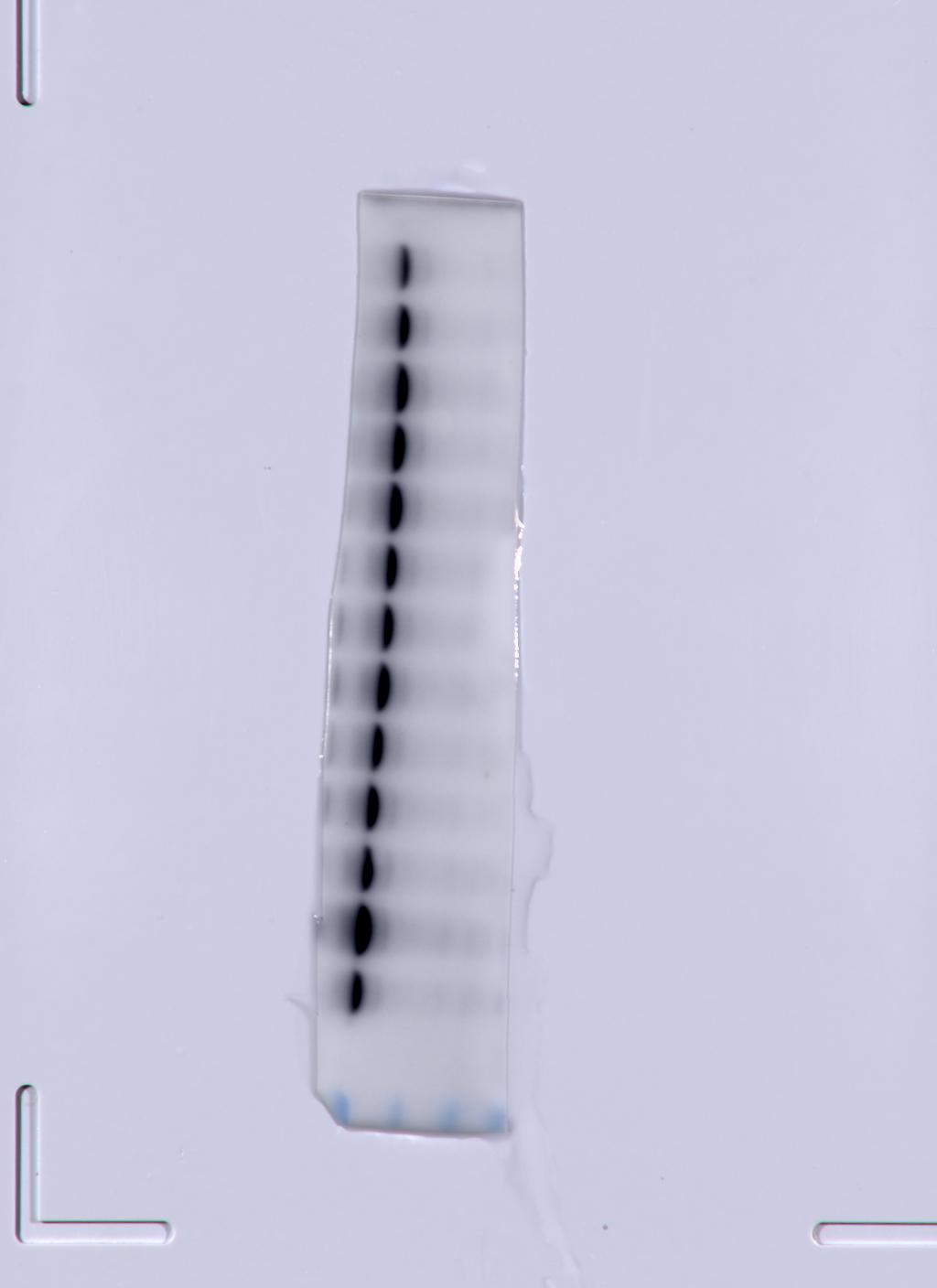

Supplement: Supplementary file 1 — Supplementary Material 1. [file 12985_2024_2385_MOESM1_ESM.zip › xuxiaoying WB/SY5Y 3d2 2021.10.09_16.32.51_Ch/3d2 2021.10.09_16.32.51_Ch+Marker.jpg]

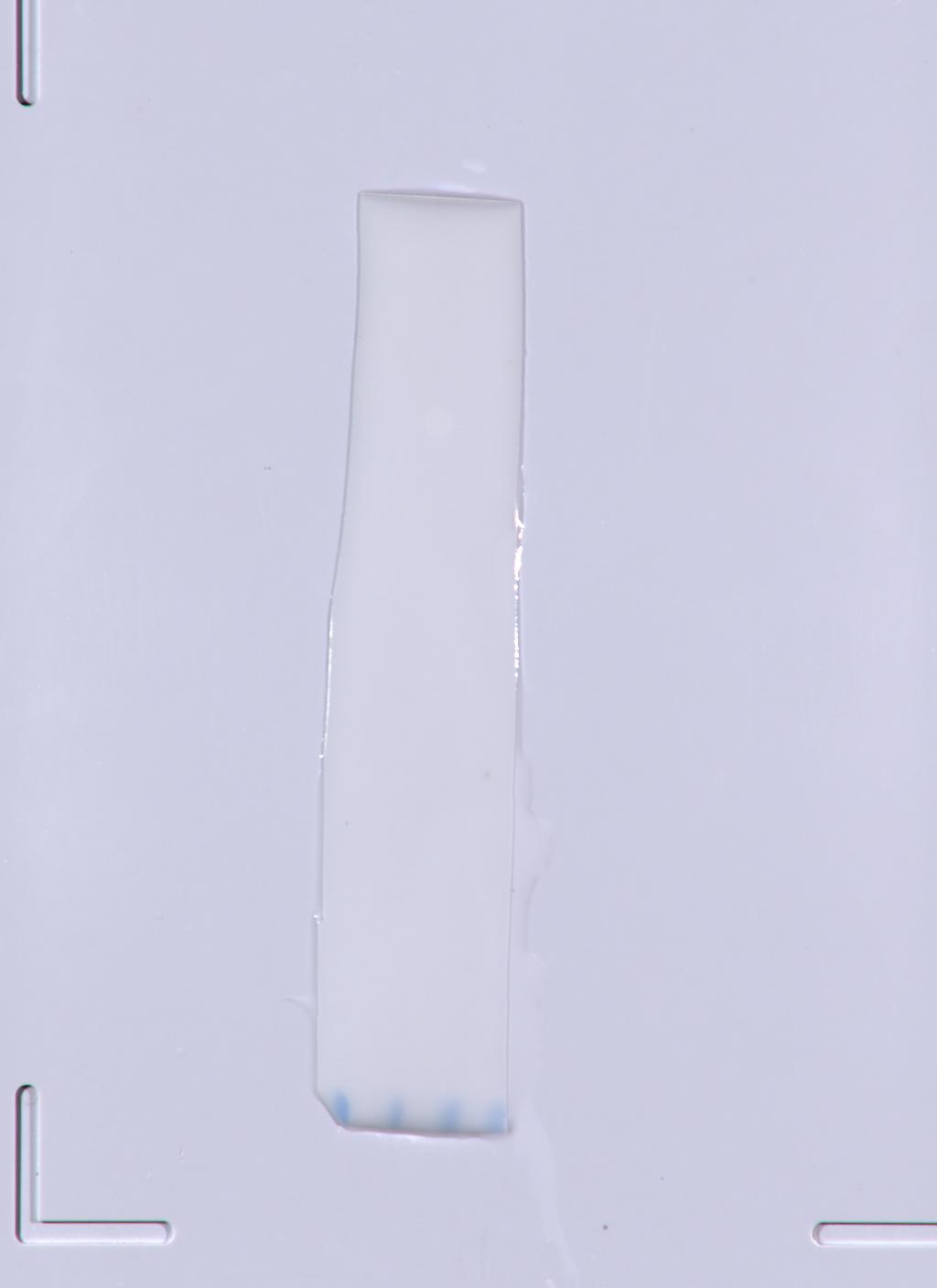

Supplement: Supplementary file 1 — Supplementary Material 1. [file 12985_2024_2385_MOESM1_ESM.zip › xuxiaoying WB/SY5Y 3d2 2021.10.09_16.32.51_Ch/3d2 2021.10.09_16.32.51_Ch-Marker.jpg]

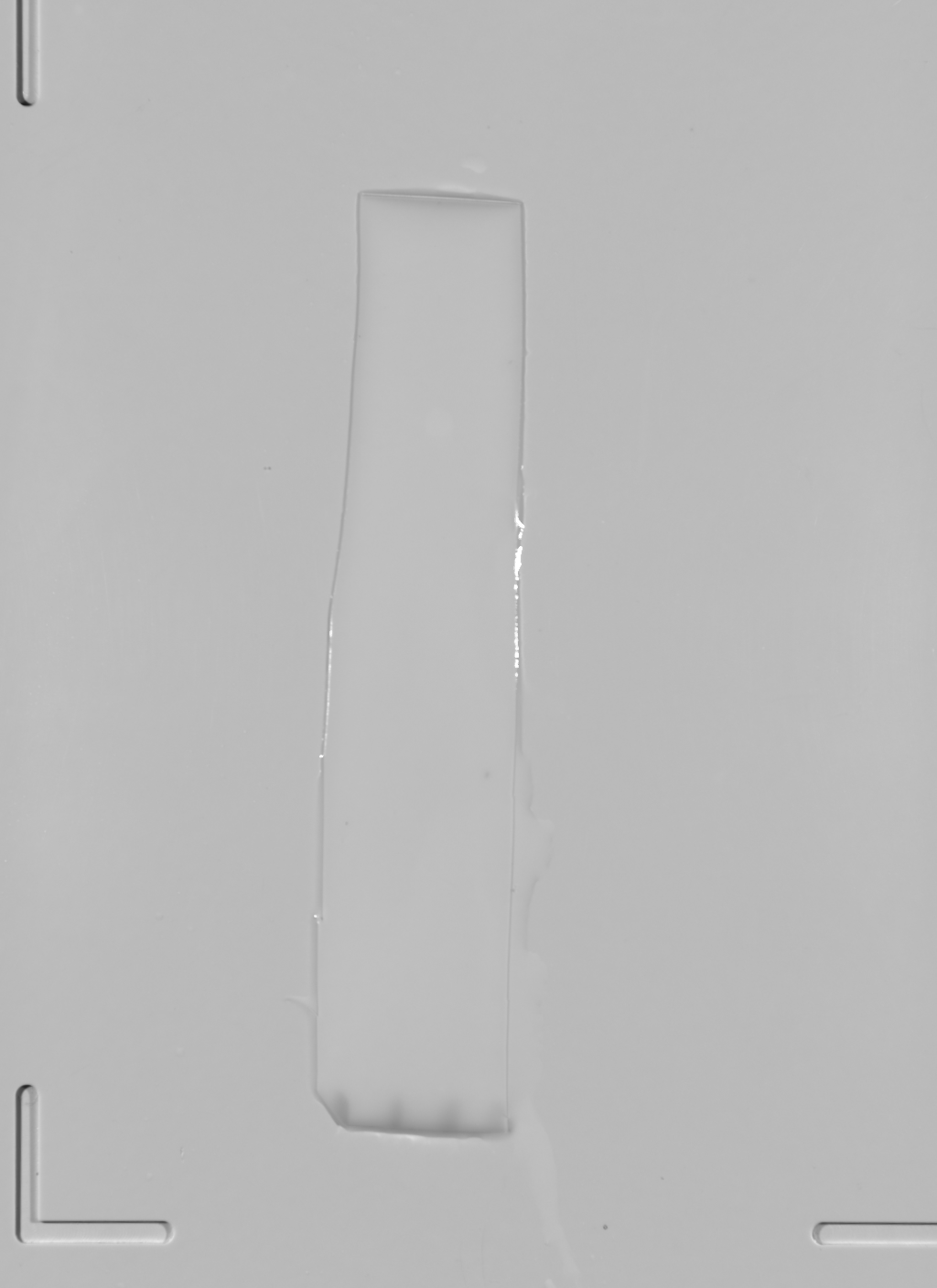

Supplement: Supplementary file 1 — Supplementary Material 1. [file 12985_2024_2385_MOESM1_ESM.zip › xuxiaoying WB/SY5Y 3d2 2021.10.09_16.32.51_Ch/3d2 2021.10.09_16.32.51_Ch-Marker.tif]

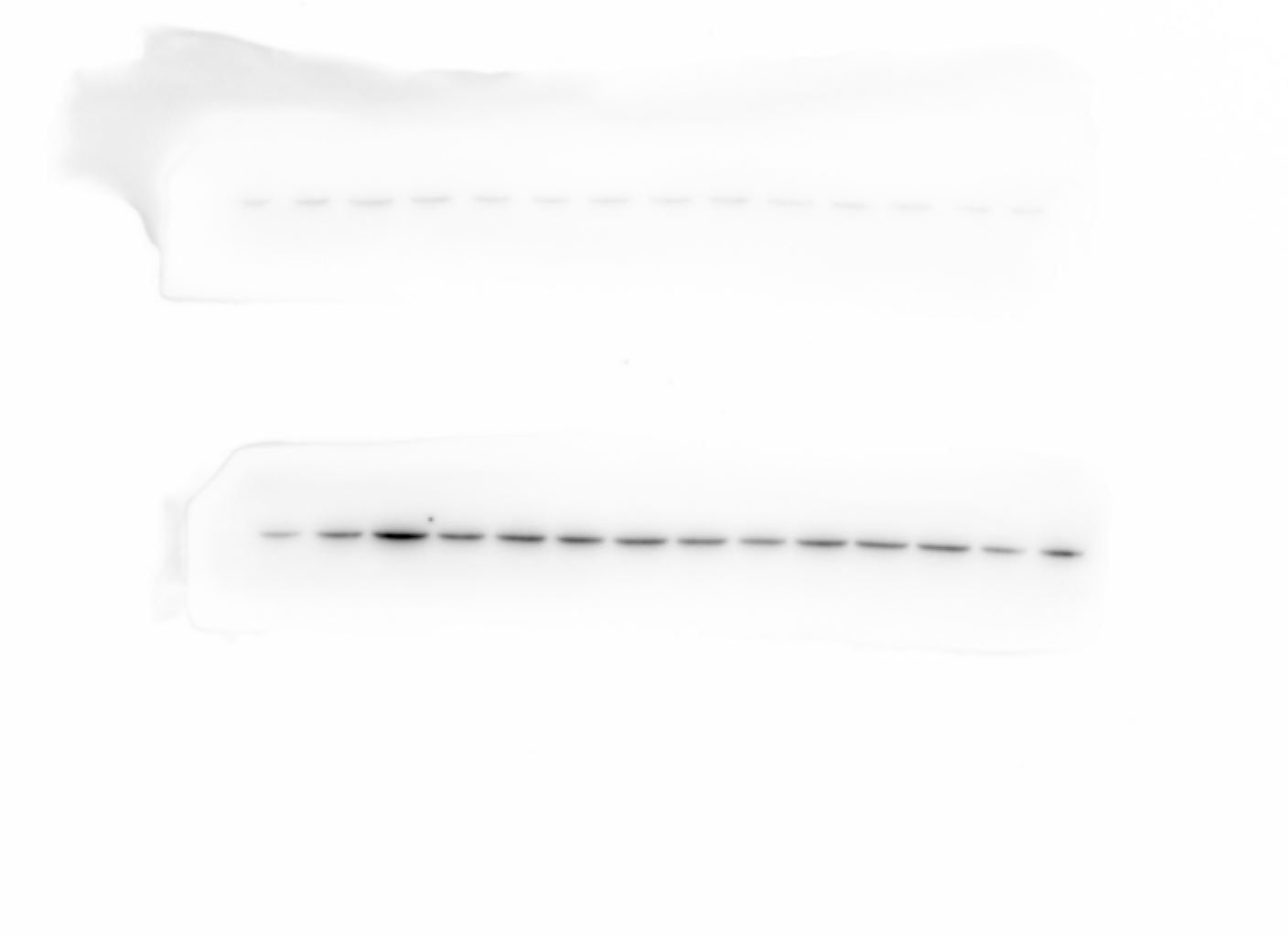

Supplement: Supplementary file 1 — Supplementary Material 1. [file 12985_2024_2385_MOESM1_ESM.zip › xuxiaoying WB/SY5Y akt 2021.09.16_18.39.59_Ch/RD+SY5Y akt 2021.09.16_18.39.59_Ch.tif]

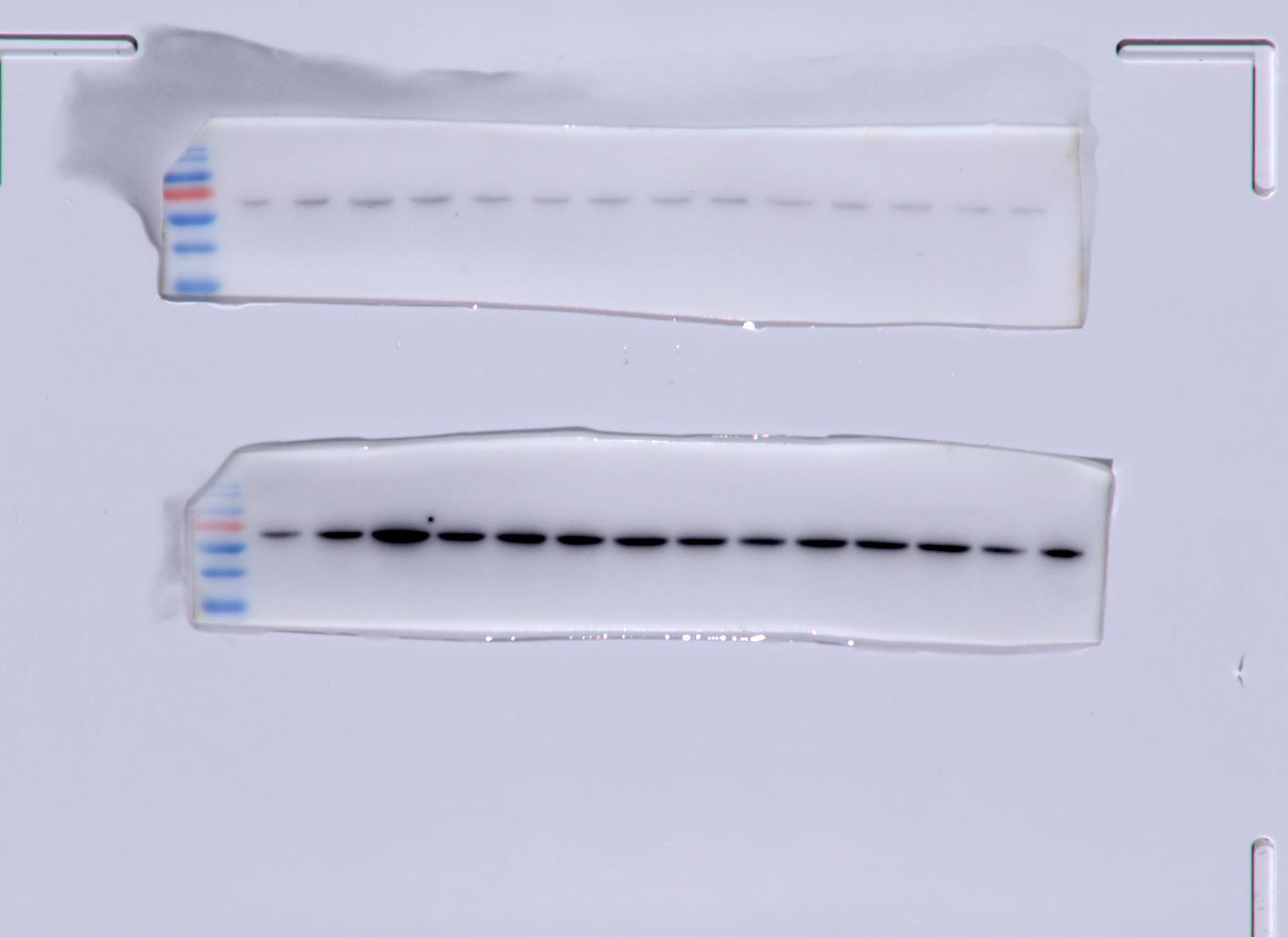

Supplement: Supplementary file 1 — Supplementary Material 1. [file 12985_2024_2385_MOESM1_ESM.zip › xuxiaoying WB/SY5Y akt 2021.09.16_18.39.59_Ch/RD+SY5Y akt 2021.09.16_18.39.59_Ch+Marker.jpg]

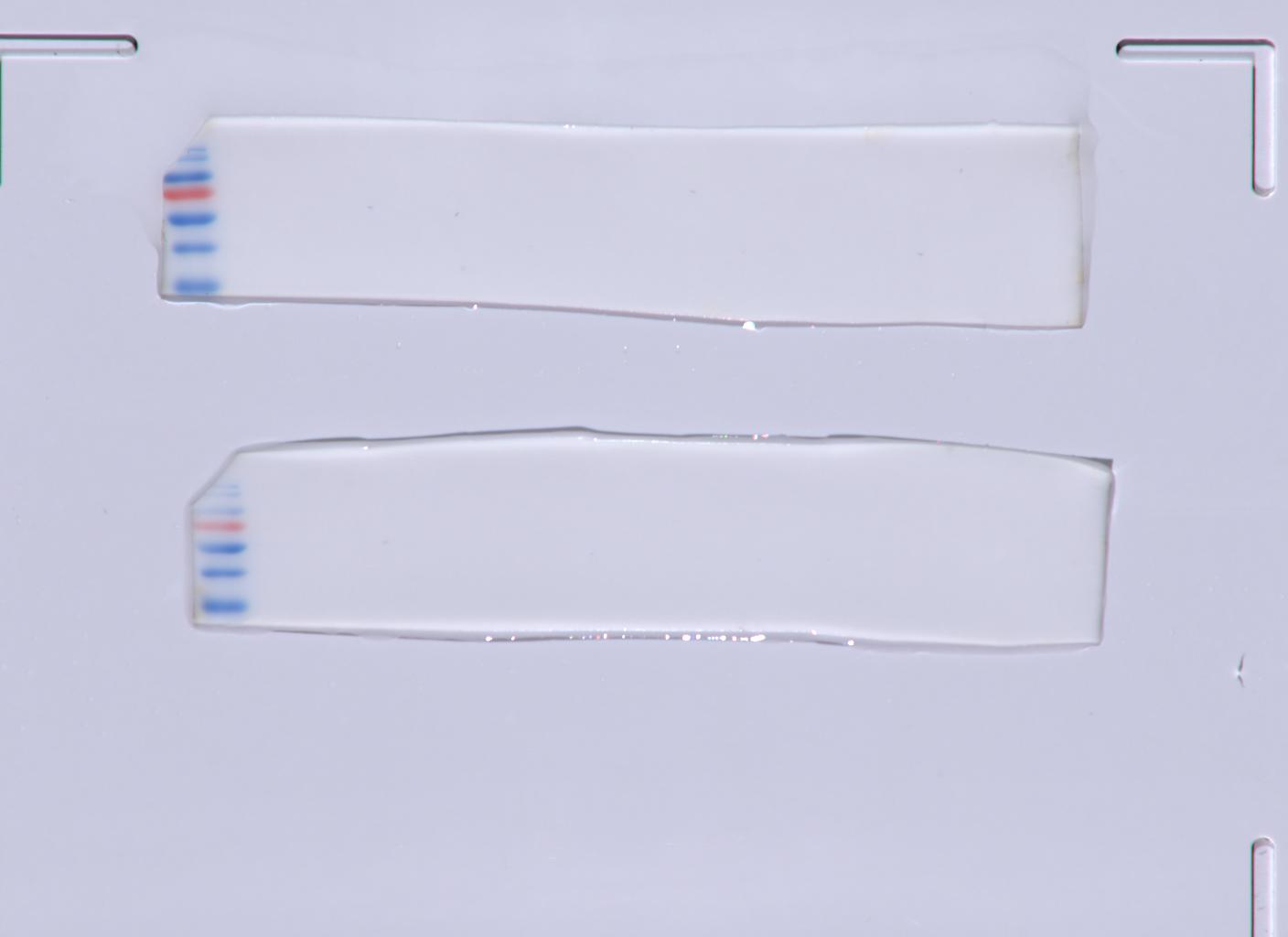

Supplement: Supplementary file 1 — Supplementary Material 1. [file 12985_2024_2385_MOESM1_ESM.zip › xuxiaoying WB/SY5Y akt 2021.09.16_18.39.59_Ch/RD+SY5Y akt 2021.09.16_18.39.59_Ch-Marker.jpg]

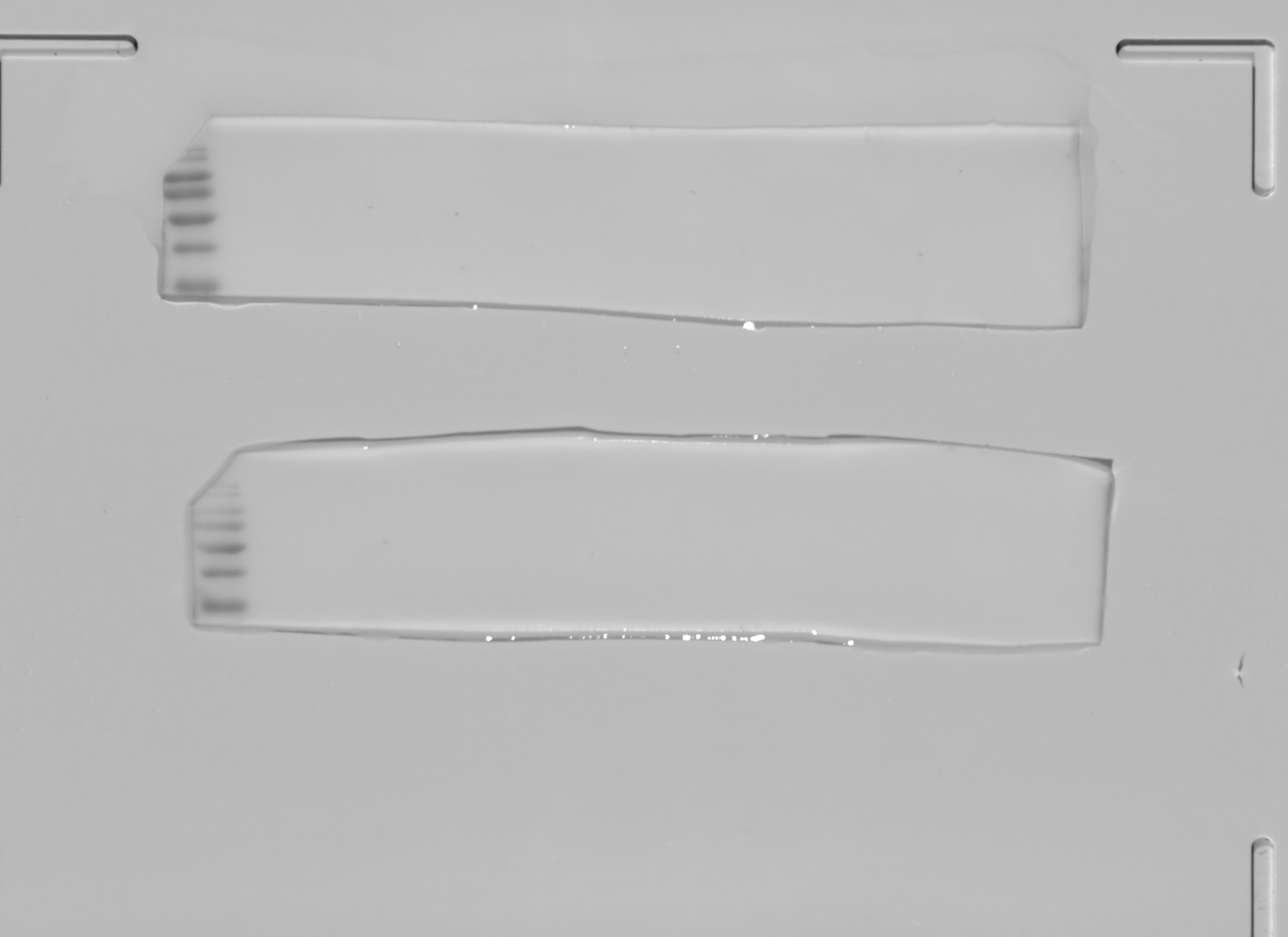

Supplement: Supplementary file 1 — Supplementary Material 1. [file 12985_2024_2385_MOESM1_ESM.zip › xuxiaoying WB/SY5Y akt 2021.09.16_18.39.59_Ch/RD+SY5Y akt 2021.09.16_18.39.59_Ch-Marker.tif]

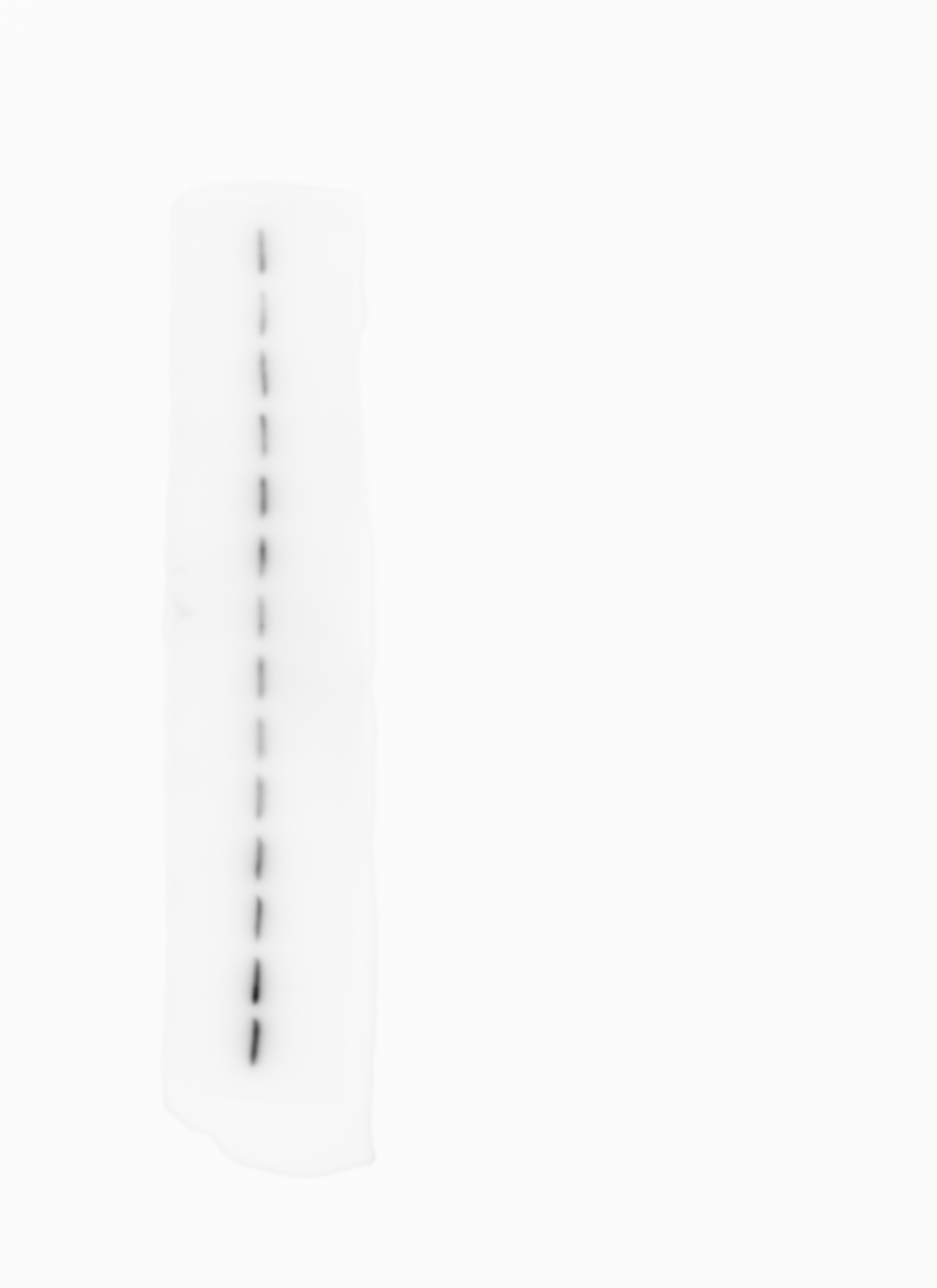

Supplement: Supplementary file 1 — Supplementary Material 1. [file 12985_2024_2385_MOESM1_ESM.zip › xuxiaoying WB/SY5Y LC3B b-actinú¿neicanú⌐2 2021.11.15_21.27.26_Ch/nc2 2021.11.15_21.27.26_Ch.tif]

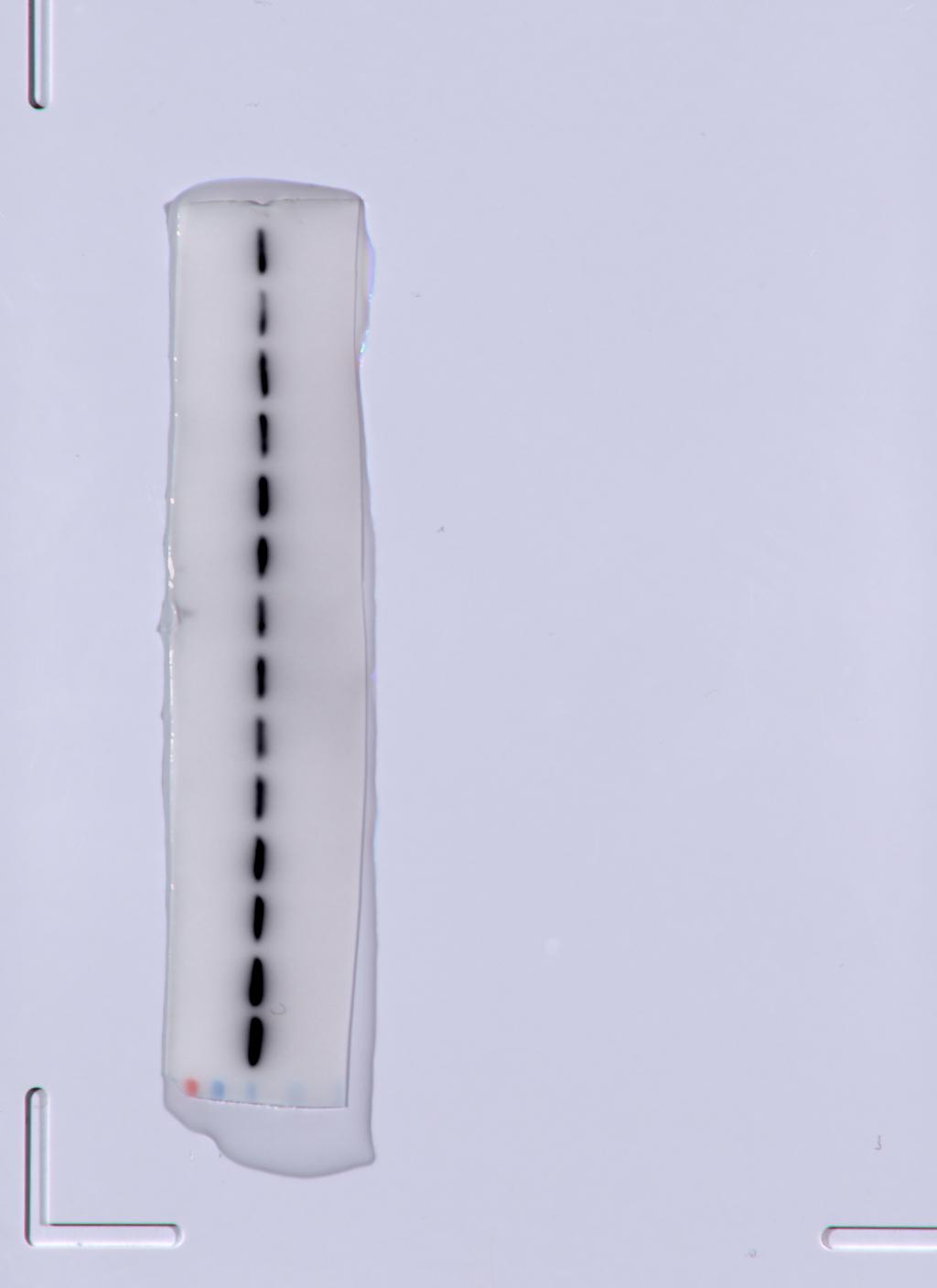

Supplement: Supplementary file 1 — Supplementary Material 1. [file 12985_2024_2385_MOESM1_ESM.zip › xuxiaoying WB/SY5Y LC3B b-actinú¿neicanú⌐2 2021.11.15_21.27.26_Ch/nc2 2021.11.15_21.27.26_Ch+Marker.jpg]

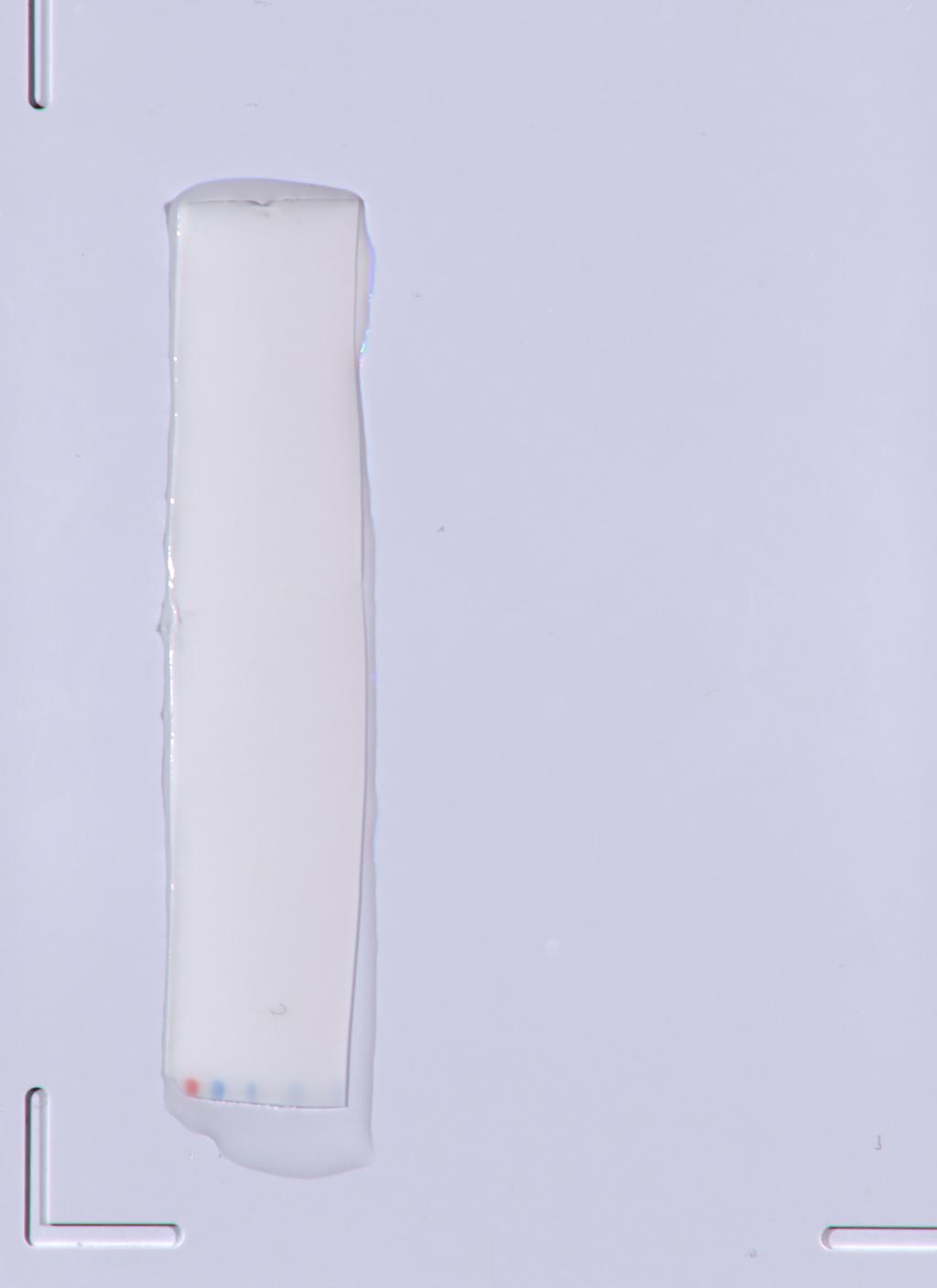

Supplement: Supplementary file 1 — Supplementary Material 1. [file 12985_2024_2385_MOESM1_ESM.zip › xuxiaoying WB/SY5Y LC3B b-actinú¿neicanú⌐2 2021.11.15_21.27.26_Ch/nc2 2021.11.15_21.27.26_Ch-Marker.jpg]

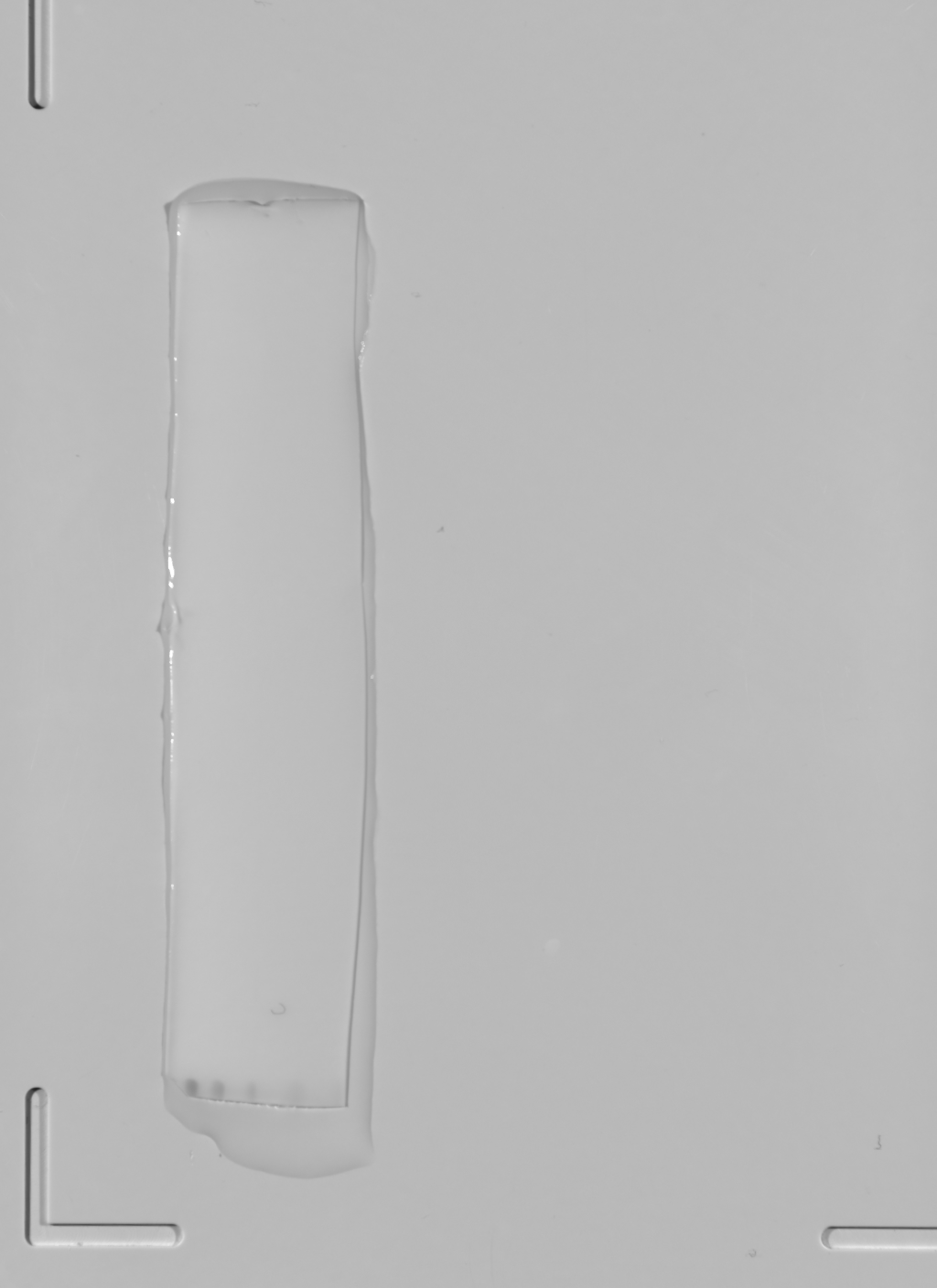

Supplement: Supplementary file 1 — Supplementary Material 1. [file 12985_2024_2385_MOESM1_ESM.zip › xuxiaoying WB/SY5Y LC3B b-actinú¿neicanú⌐2 2021.11.15_21.27.26_Ch/nc2 2021.11.15_21.27.26_Ch-Marker.tif]

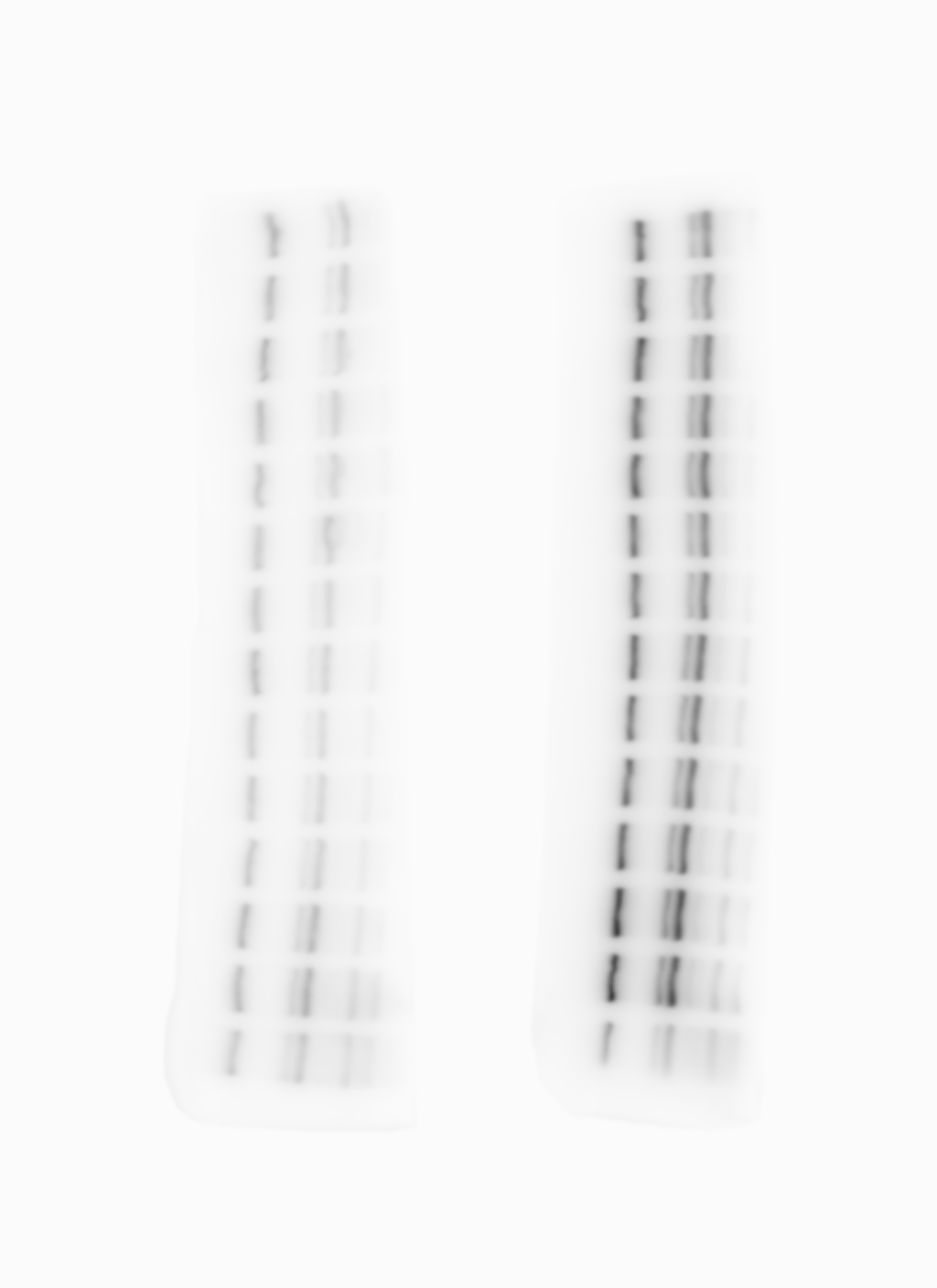

Supplement: Supplementary file 1 — Supplementary Material 1. [file 12985_2024_2385_MOESM1_ESM.zip › xuxiaoying WB/SY5Y mtor 3 2021.10.30_23.50.47_Ch/mtor3 3 2021.10.30_23.50.47_Ch.tif]

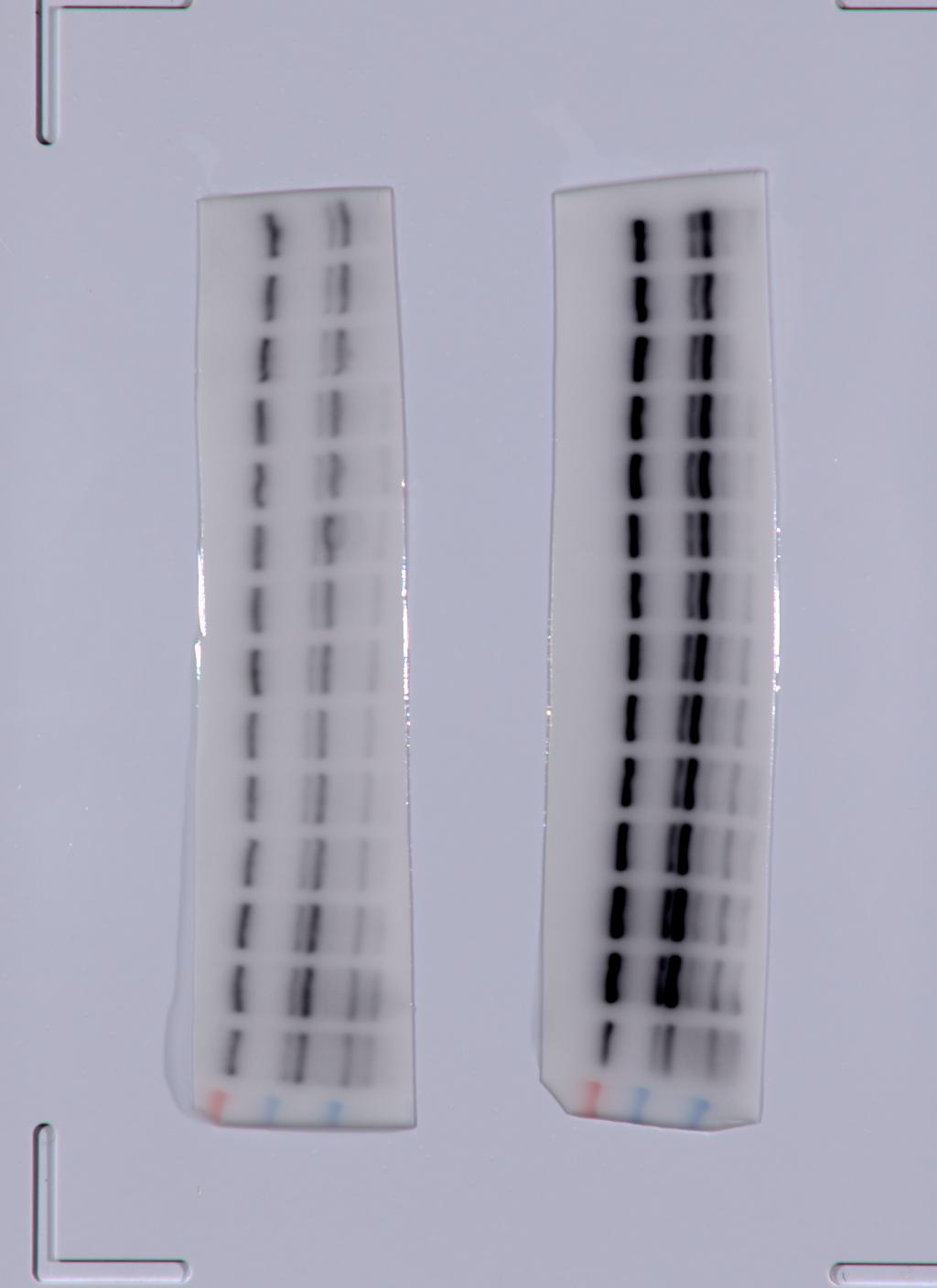

Supplement: Supplementary file 1 — Supplementary Material 1. [file 12985_2024_2385_MOESM1_ESM.zip › xuxiaoying WB/SY5Y mtor 3 2021.10.30_23.50.47_Ch/mtor3 3 2021.10.30_23.50.47_Ch+Marker.jpg]

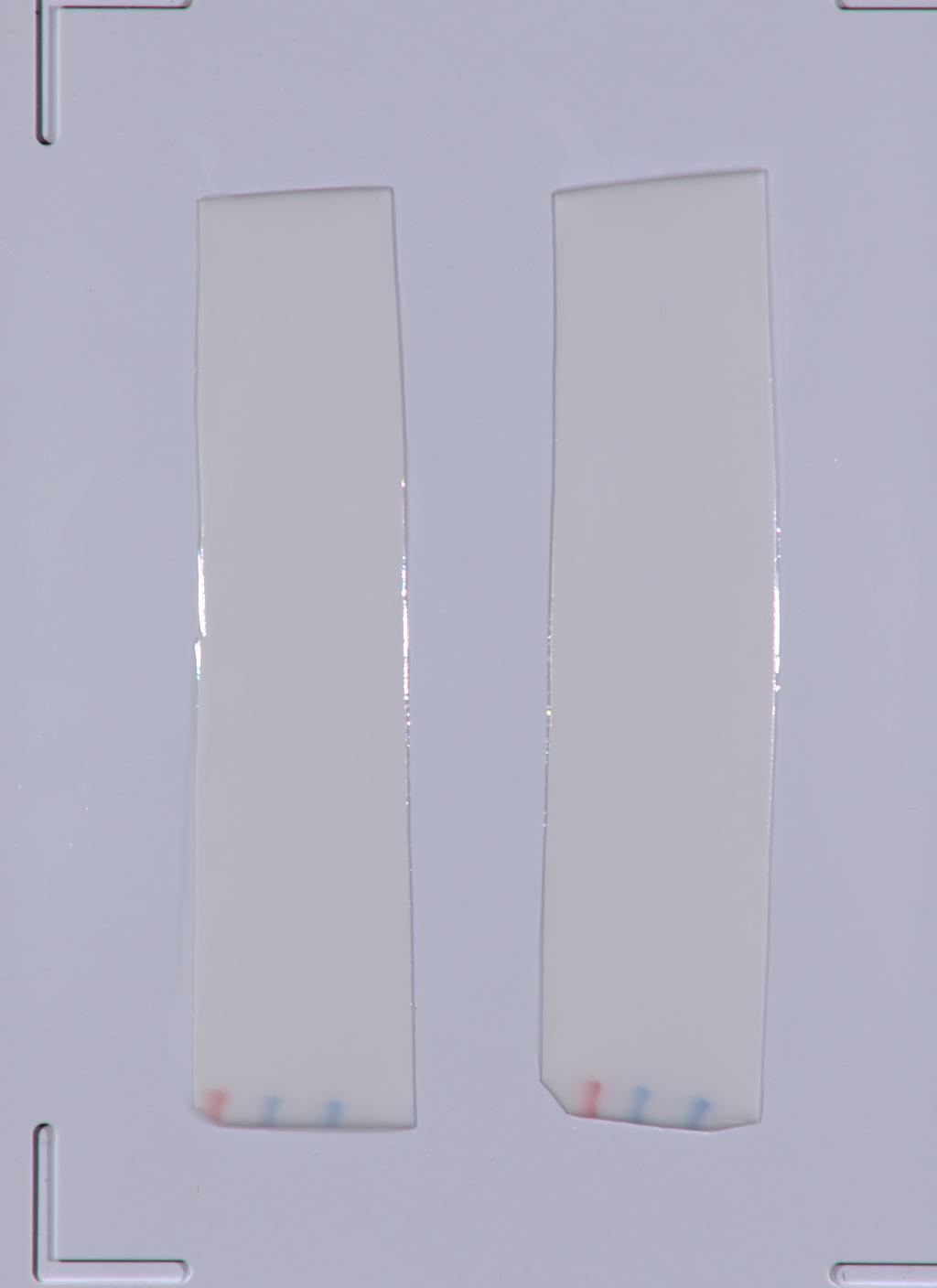

Supplement: Supplementary file 1 — Supplementary Material 1. [file 12985_2024_2385_MOESM1_ESM.zip › xuxiaoying WB/SY5Y mtor 3 2021.10.30_23.50.47_Ch/mtor3 3 2021.10.30_23.50.47_Ch-Marker.jpg]

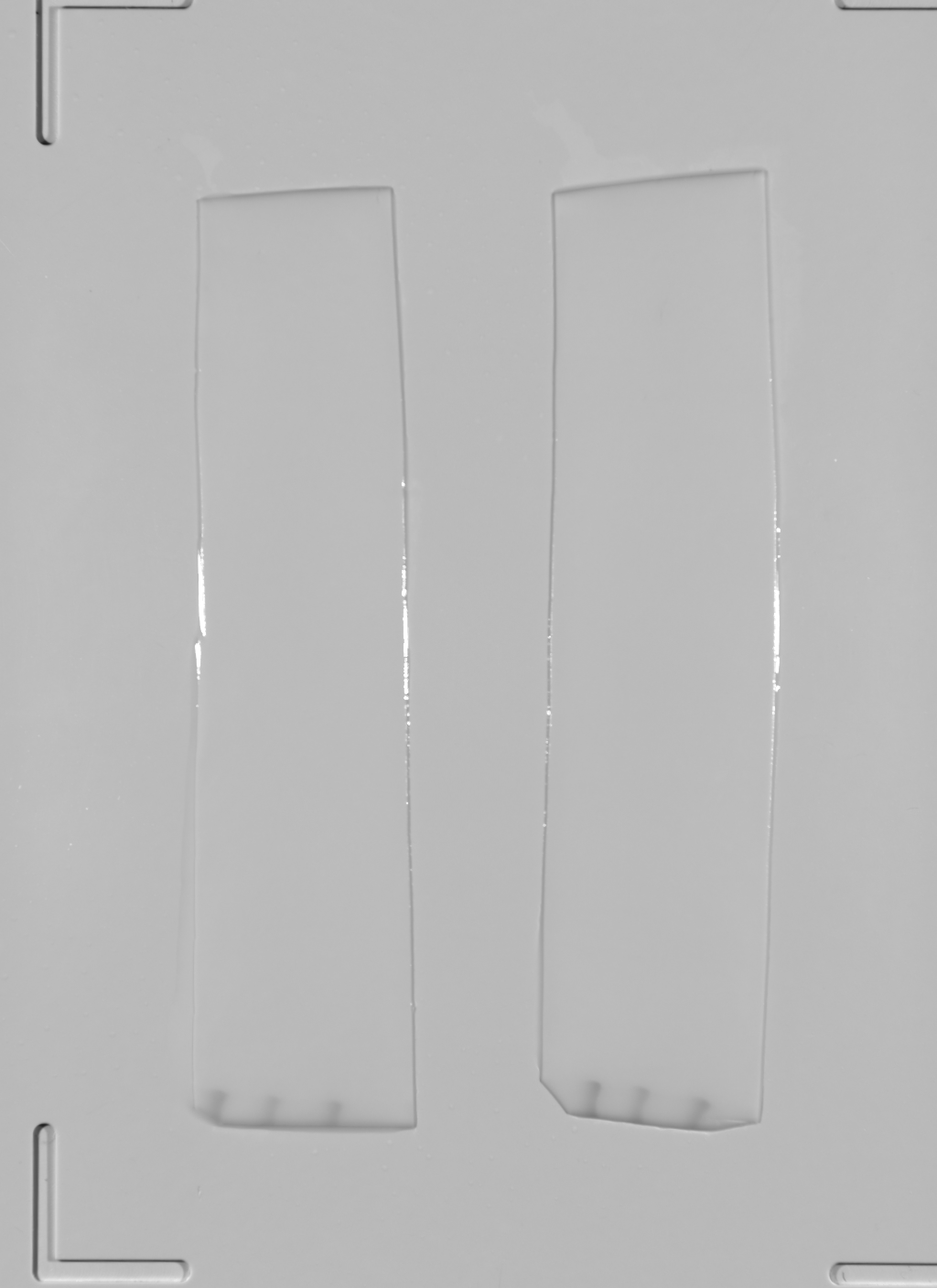

Supplement: Supplementary file 1 — Supplementary Material 1. [file 12985_2024_2385_MOESM1_ESM.zip › xuxiaoying WB/SY5Y mtor 3 2021.10.30_23.50.47_Ch/mtor3 3 2021.10.30_23.50.47_Ch-Marker.tif]

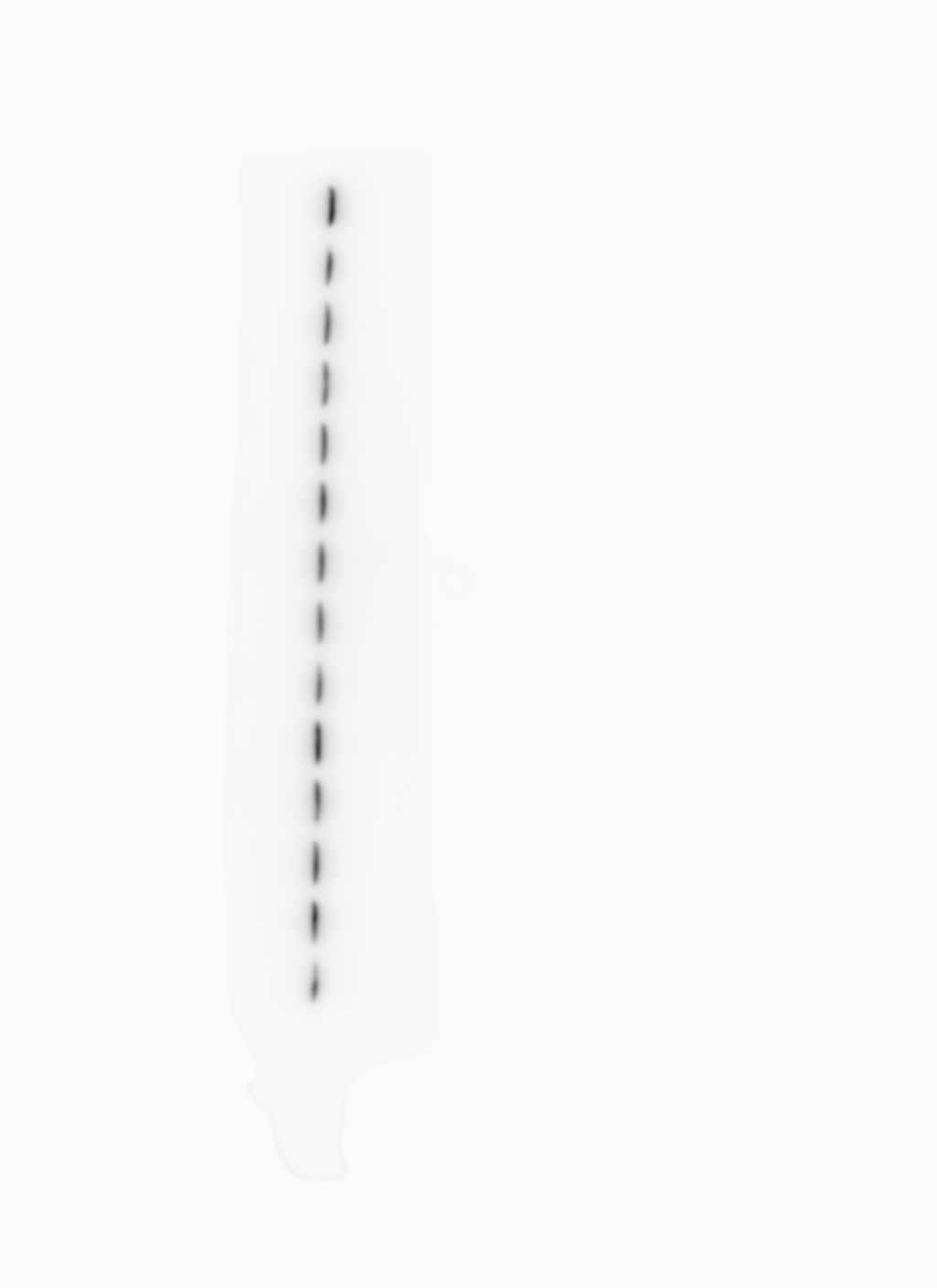

Supplement: Supplementary file 1 — Supplementary Material 1. [file 12985_2024_2385_MOESM1_ESM.zip › xuxiaoying WB/SY5Y mTOR nc2-1 2021.11.15_21.39.45_Ch/nc2-1 2021.11.15_21.39.45_Ch.tif]

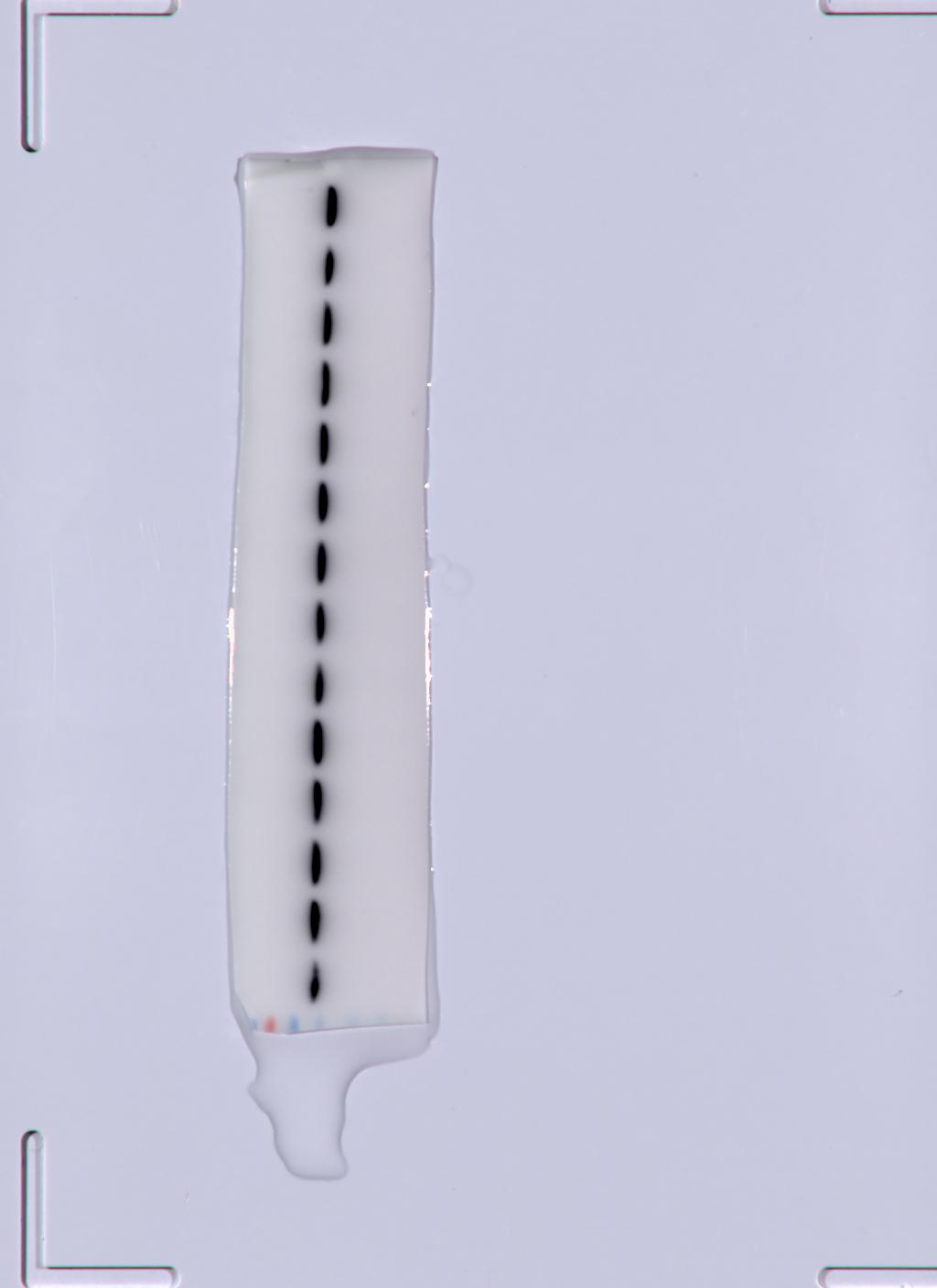

Supplement: Supplementary file 1 — Supplementary Material 1. [file 12985_2024_2385_MOESM1_ESM.zip › xuxiaoying WB/SY5Y mTOR nc2-1 2021.11.15_21.39.45_Ch/nc2-1 2021.11.15_21.39.45_Ch+Marker.jpg]

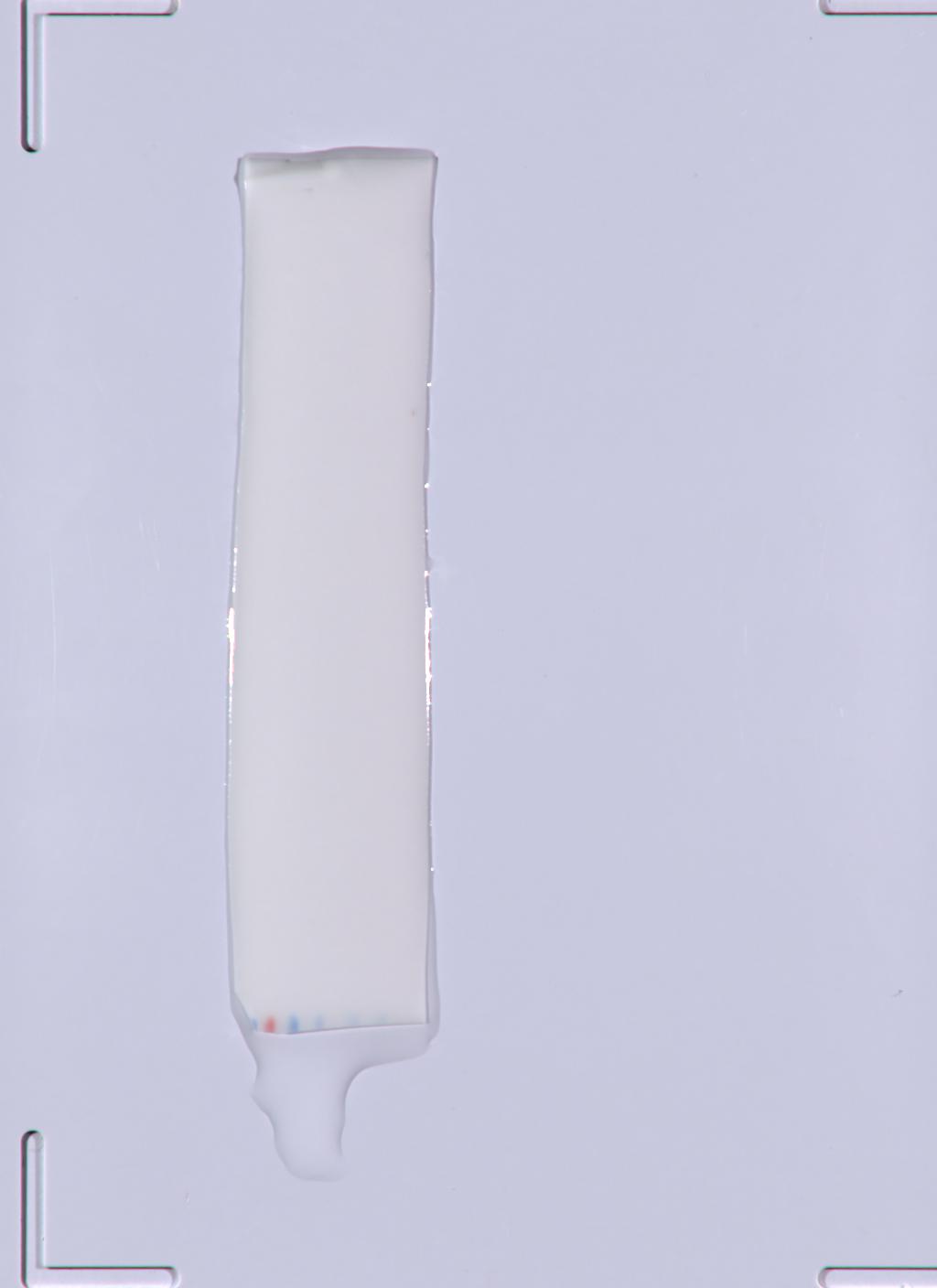

Supplement: Supplementary file 1 — Supplementary Material 1. [file 12985_2024_2385_MOESM1_ESM.zip › xuxiaoying WB/SY5Y mTOR nc2-1 2021.11.15_21.39.45_Ch/nc2-1 2021.11.15_21.39.45_Ch-Marker.jpg]

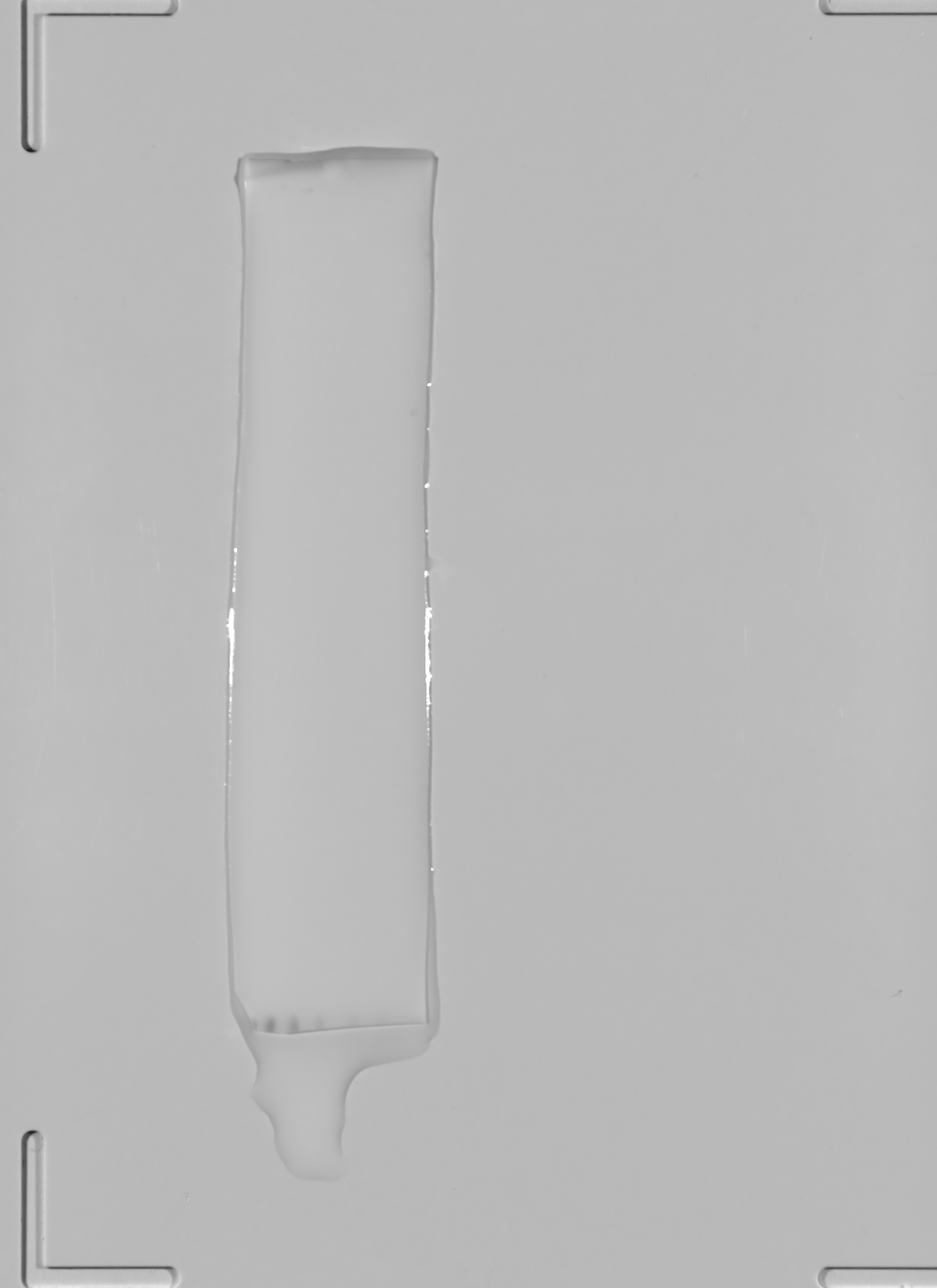

Supplement: Supplementary file 1 — Supplementary Material 1. [file 12985_2024_2385_MOESM1_ESM.zip › xuxiaoying WB/SY5Y mTOR nc2-1 2021.11.15_21.39.45_Ch/nc2-1 2021.11.15_21.39.45_Ch-Marker.tif]

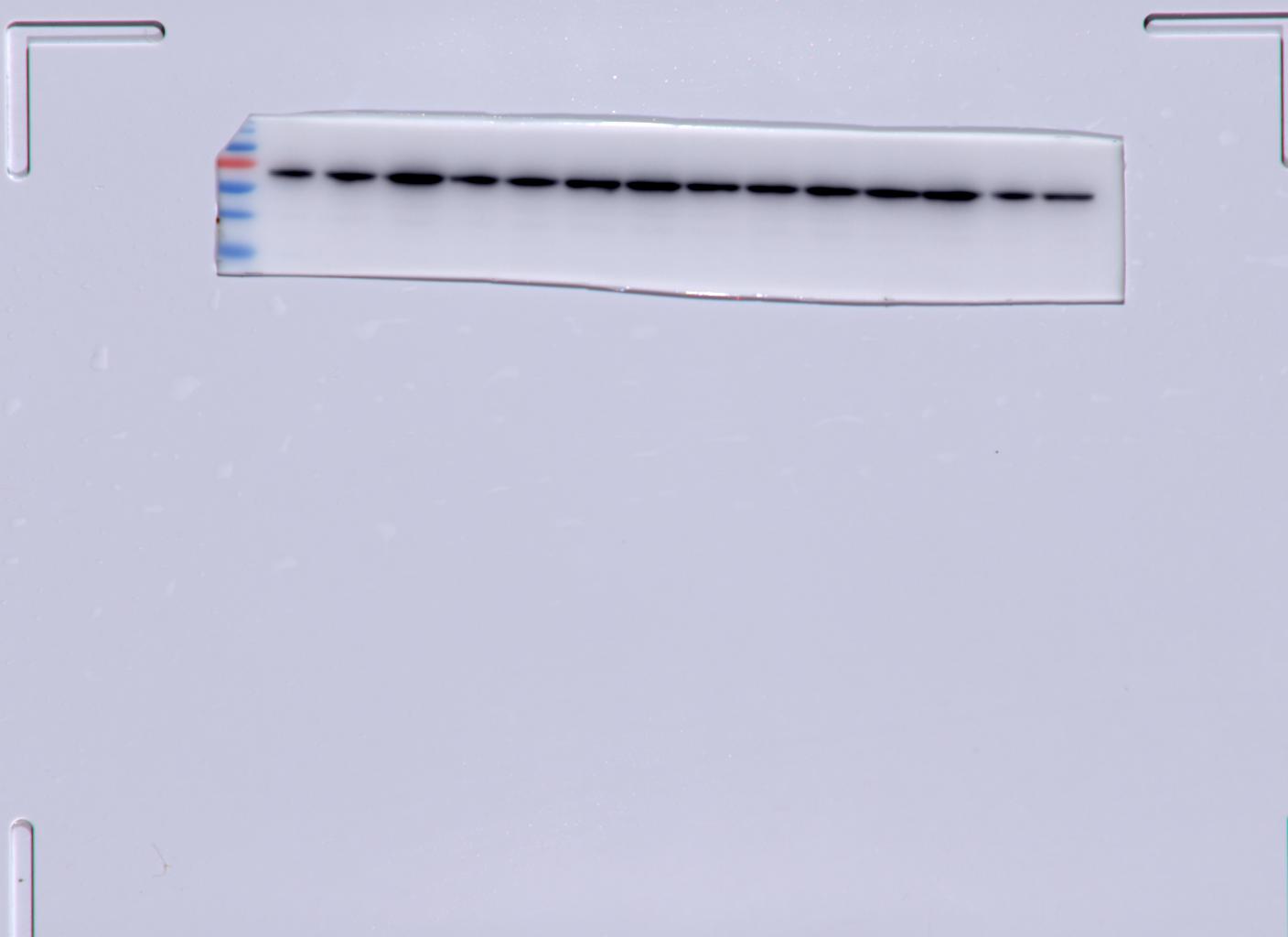

Supplement: Supplementary file 1 — Supplementary Material 1. [file 12985_2024_2385_MOESM1_ESM.zip › xuxiaoying WB/SY5Y p62 2021.09.16_17.45.07_Ch/p62 2021.09.16_17.45.07_Ch+Marker.jpg]

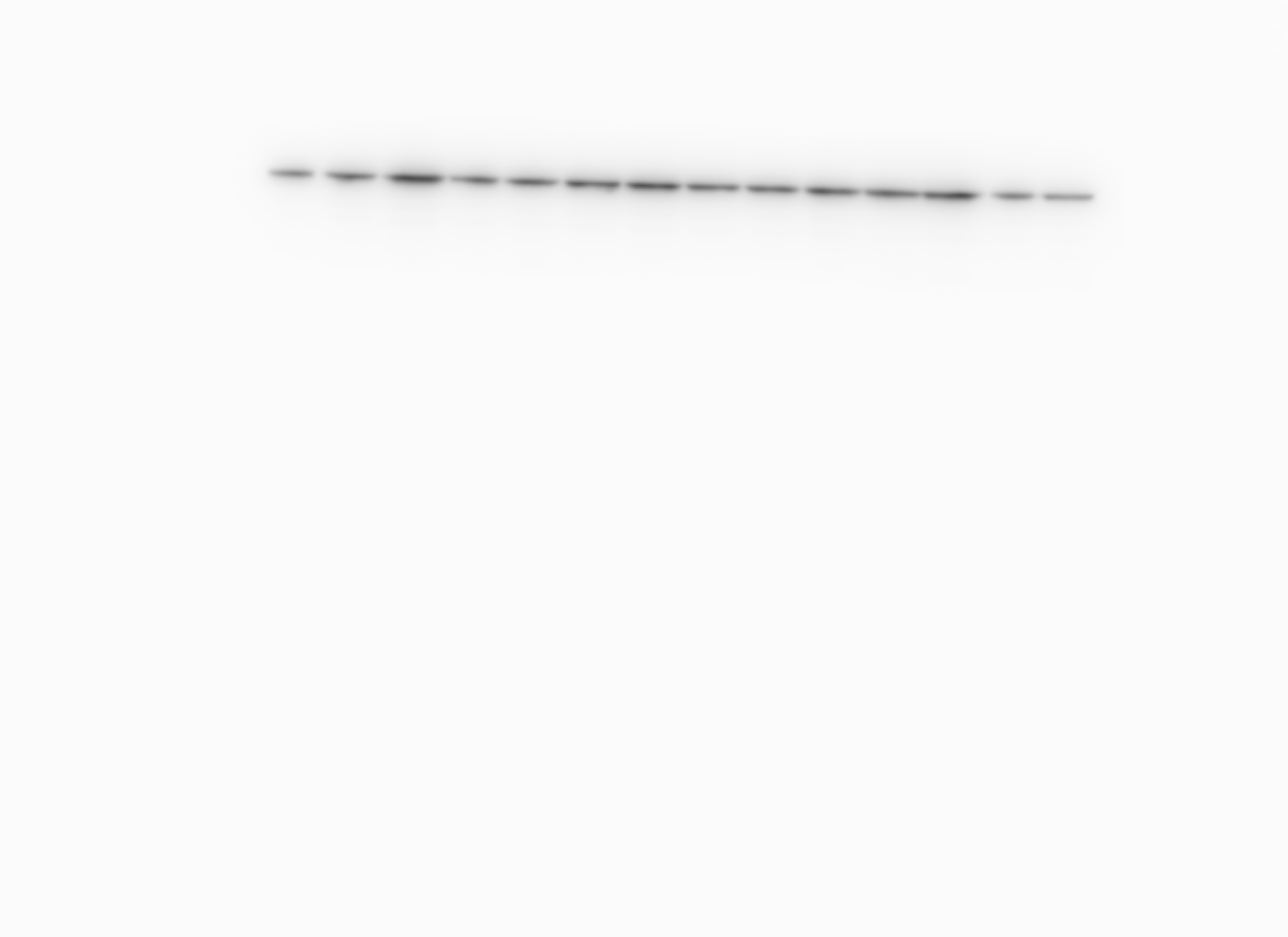

Supplement: Supplementary file 1 — Supplementary Material 1. [file 12985_2024_2385_MOESM1_ESM.zip › xuxiaoying WB/SY5Y p62 2021.09.16_17.45.07_Ch/p62 2021.09.16_17.45.07_Ch.tif]

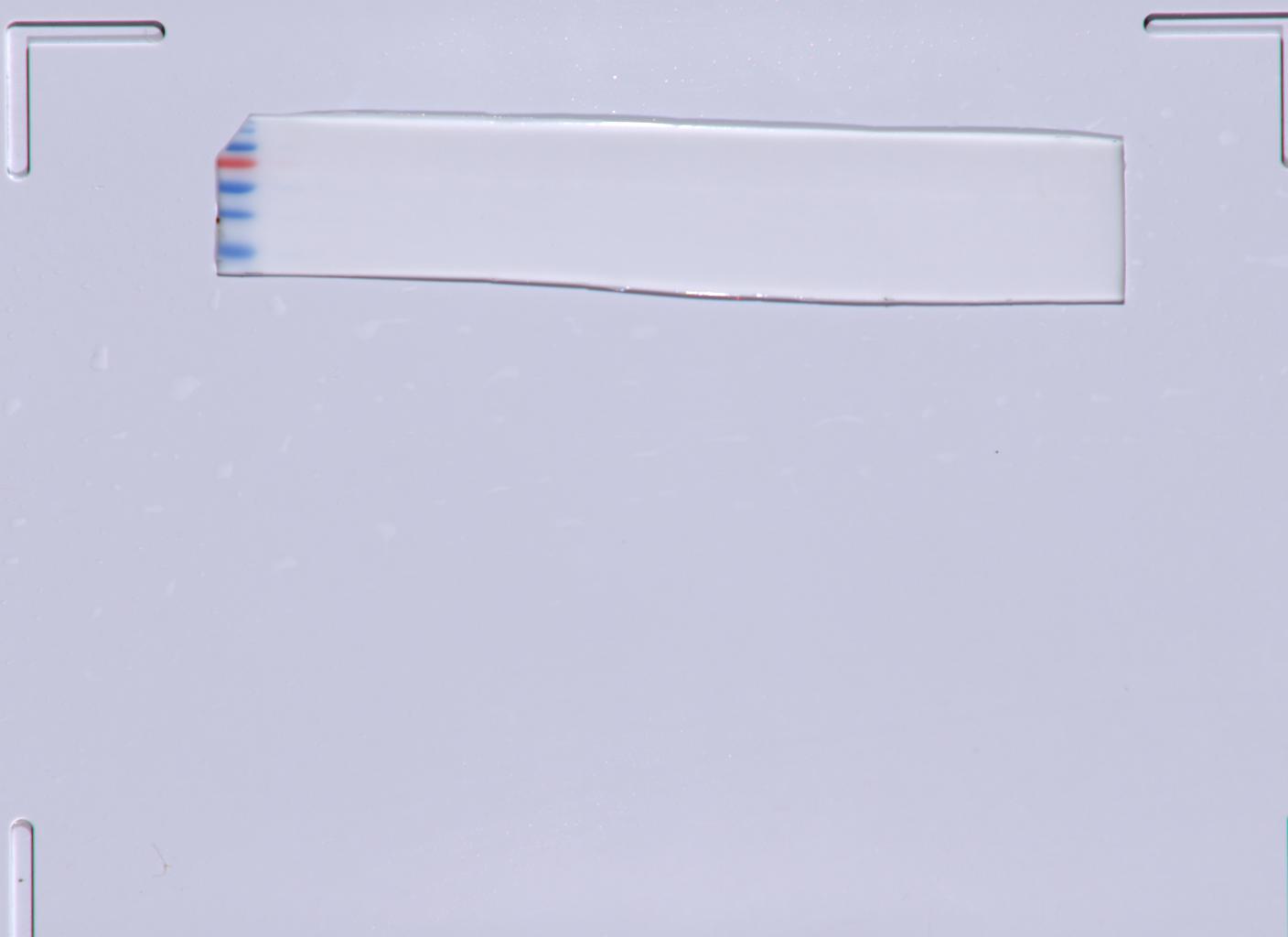

Supplement: Supplementary file 1 — Supplementary Material 1. [file 12985_2024_2385_MOESM1_ESM.zip › xuxiaoying WB/SY5Y p62 2021.09.16_17.45.07_Ch/p62 2021.09.16_17.45.07_Ch-Marker.jpg]

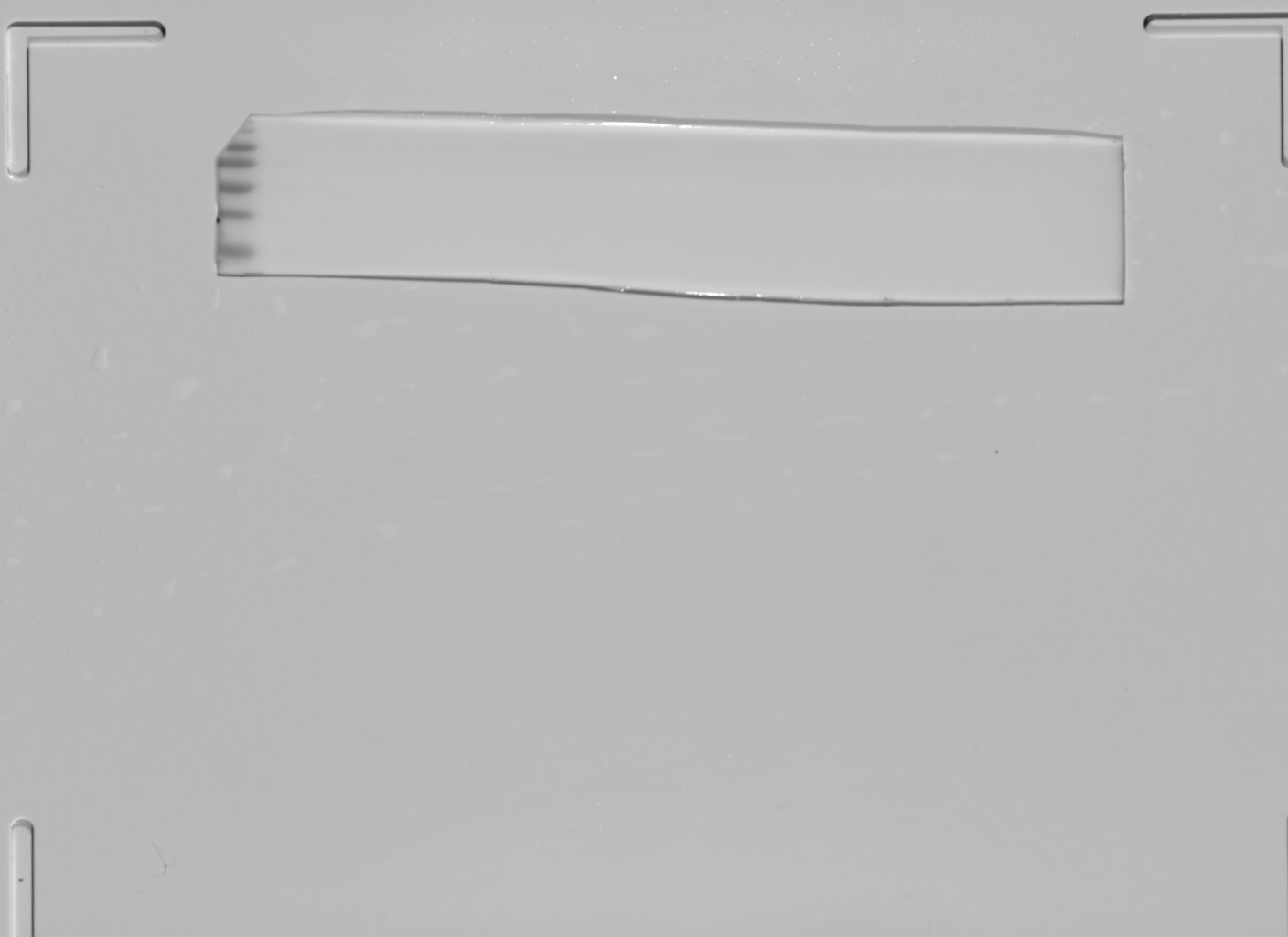

Supplement: Supplementary file 1 — Supplementary Material 1. [file 12985_2024_2385_MOESM1_ESM.zip › xuxiaoying WB/SY5Y p62 2021.09.16_17.45.07_Ch/p62 2021.09.16_17.45.07_Ch-Marker.tif]

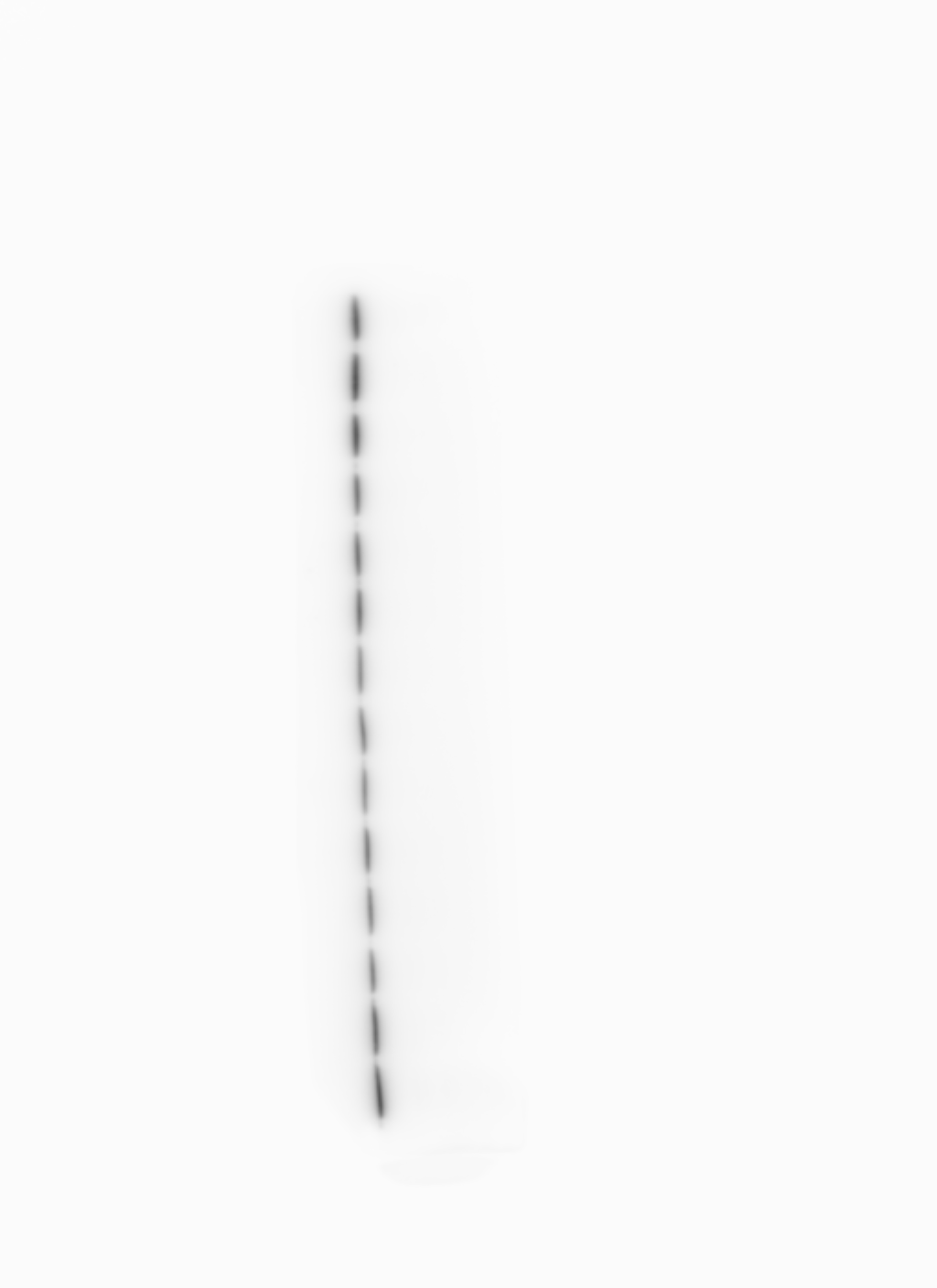

Supplement: Supplementary file 1 — Supplementary Material 1. [file 12985_2024_2385_MOESM1_ESM.zip › xuxiaoying WB/SY5Y p-akt2 2021.11.18_23.08.11_Ch/p-akt2 2021.11.18_23.08.11_Ch.tif]

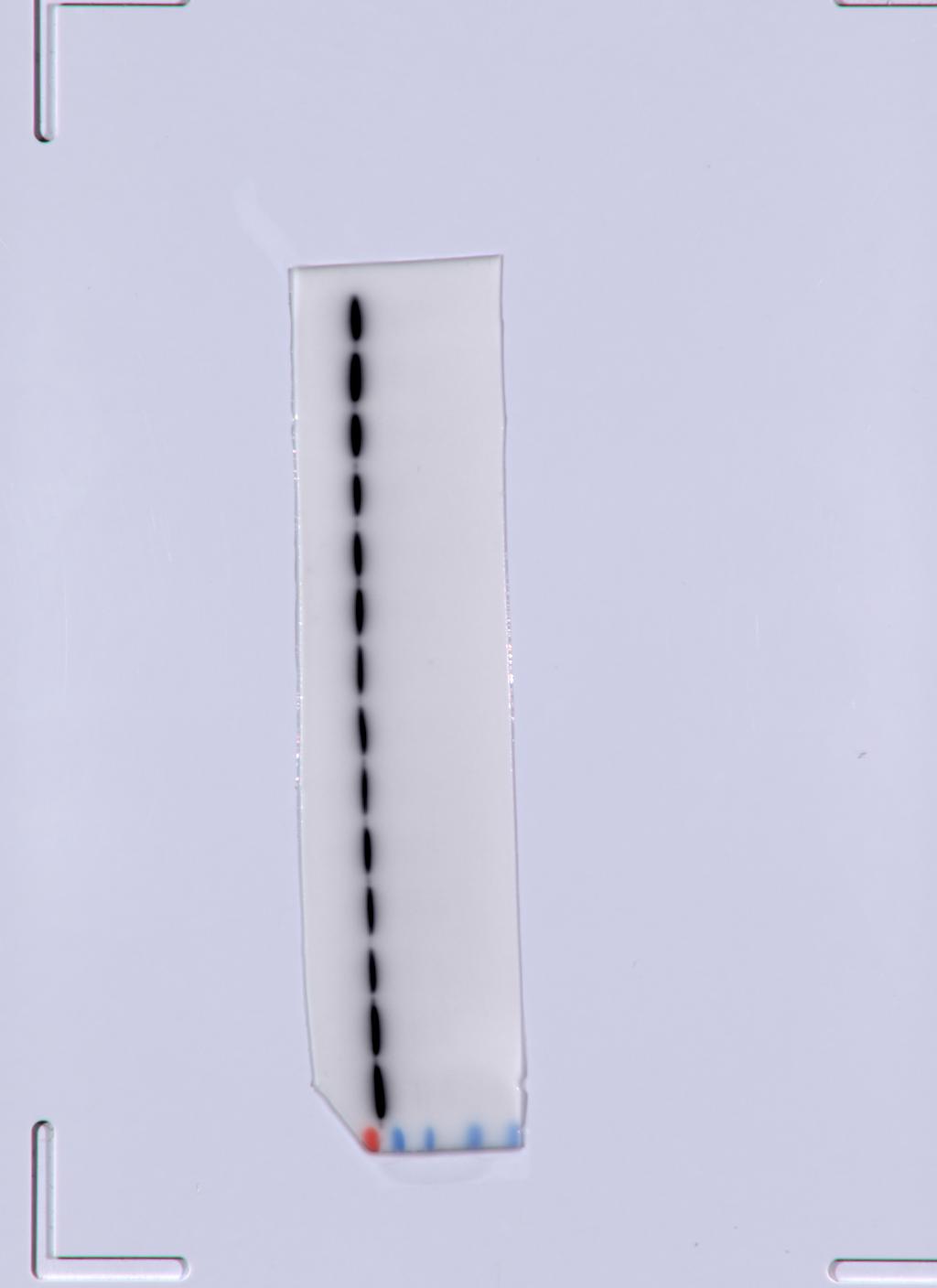

Supplement: Supplementary file 1 — Supplementary Material 1. [file 12985_2024_2385_MOESM1_ESM.zip › xuxiaoying WB/SY5Y p-akt2 2021.11.18_23.08.11_Ch/p-akt2 2021.11.18_23.08.11_Ch+Marker.jpg]

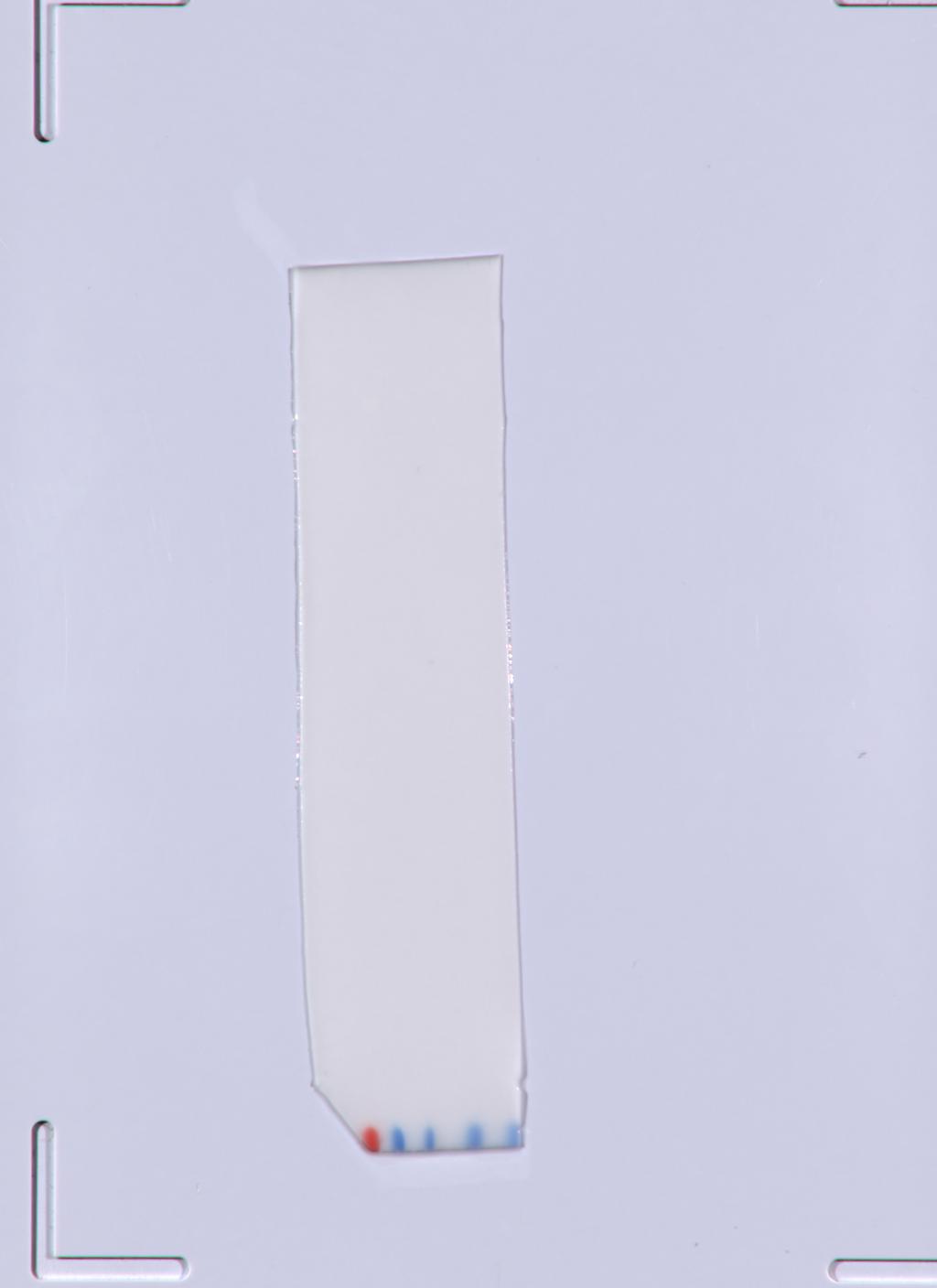

Supplement: Supplementary file 1 — Supplementary Material 1. [file 12985_2024_2385_MOESM1_ESM.zip › xuxiaoying WB/SY5Y p-akt2 2021.11.18_23.08.11_Ch/p-akt2 2021.11.18_23.08.11_Ch-Marker.jpg]

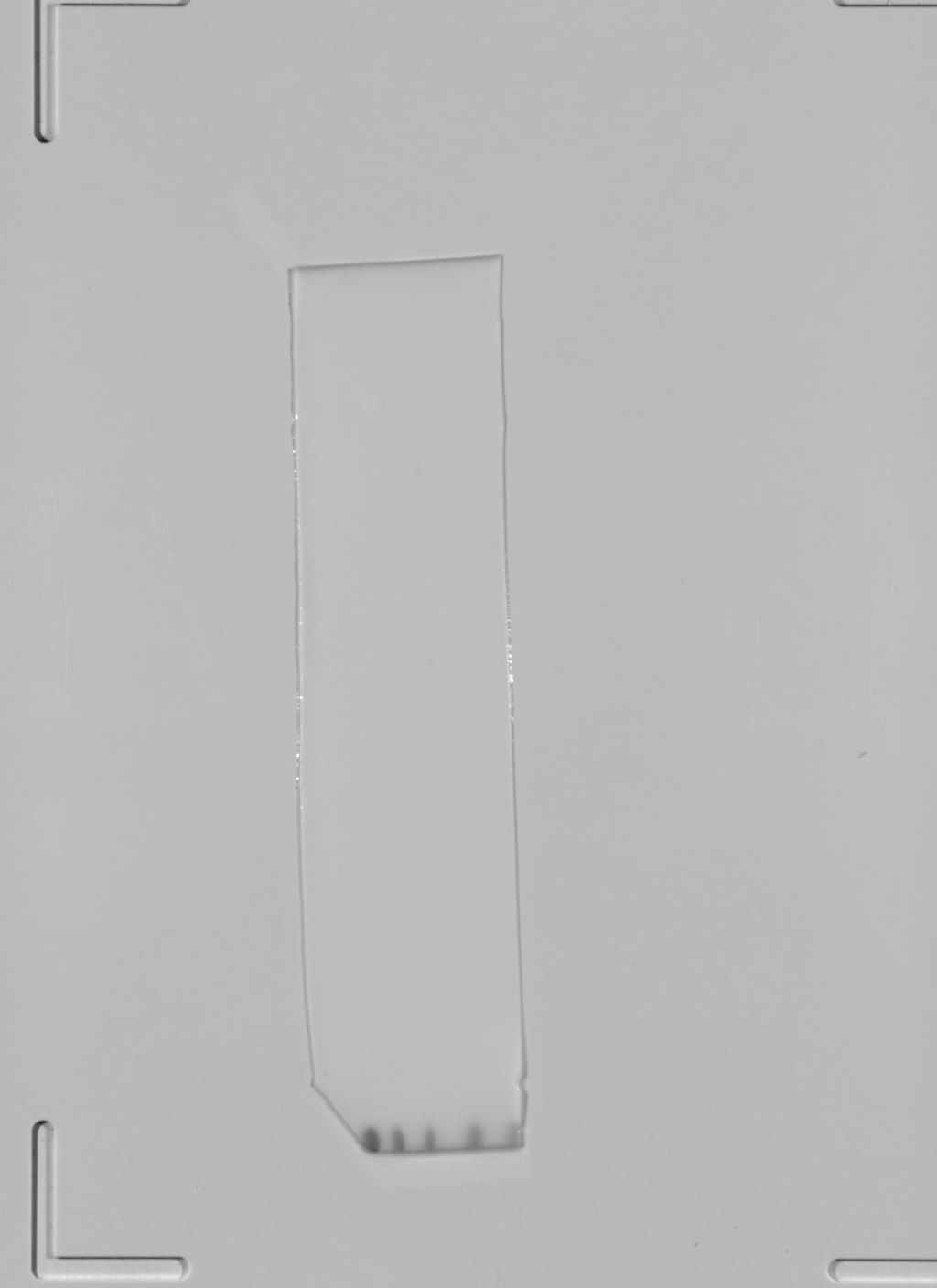

Supplement: Supplementary file 1 — Supplementary Material 1. [file 12985_2024_2385_MOESM1_ESM.zip › xuxiaoying WB/SY5Y p-akt2 2021.11.18_23.08.11_Ch/p-akt2 2021.11.18_23.08.11_Ch-Marker.tif]

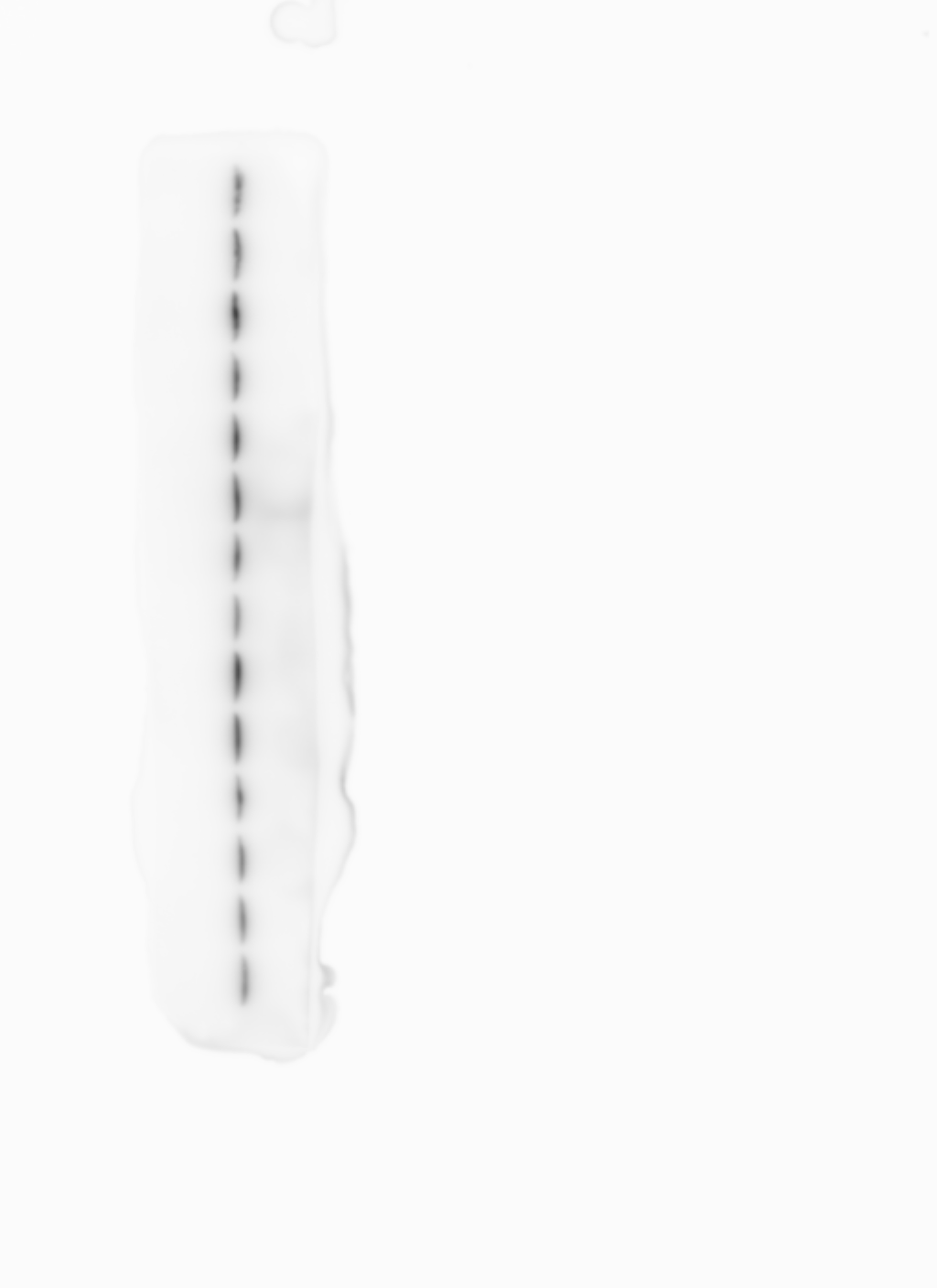

Supplement: Supplementary file 1 — Supplementary Material 1. [file 12985_2024_2385_MOESM1_ESM.zip › xuxiaoying WB/SY5Y p-mTOR NC(╔╧RD,╧┬ SY5Y)2 2021.11.14_18.30.58_Ch/ú¿ú⌐nc-1 2021.11.15_21.23.17_Ch/nc-1 2021.11.15_21.23.17_Ch.tif]

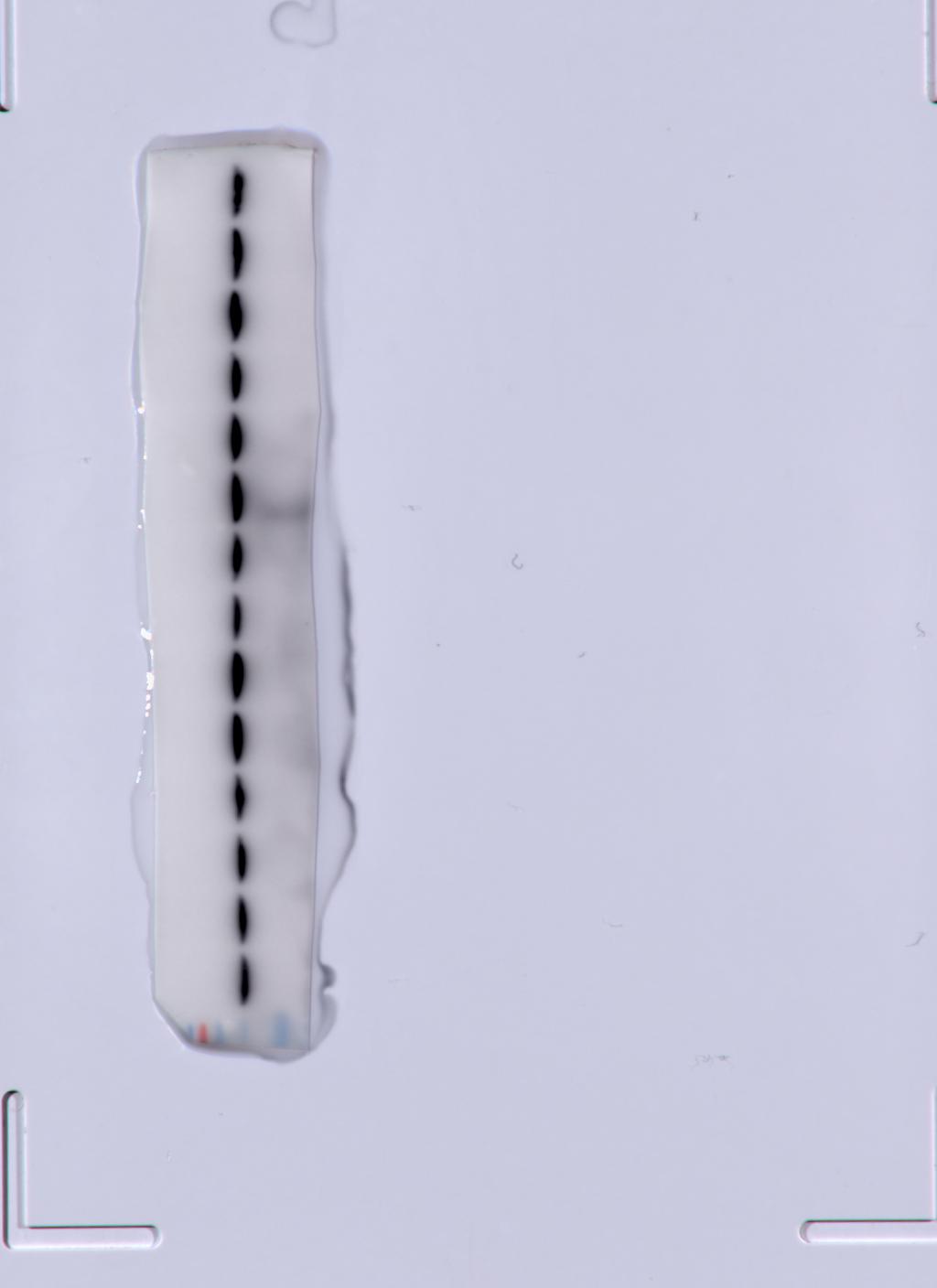

Supplement: Supplementary file 1 — Supplementary Material 1. [file 12985_2024_2385_MOESM1_ESM.zip › xuxiaoying WB/SY5Y p-mTOR NC(╔╧RD,╧┬ SY5Y)2 2021.11.14_18.30.58_Ch/ú¿ú⌐nc-1 2021.11.15_21.23.17_Ch/nc-1 2021.11.15_21.23.17_Ch+Marker.jpg]

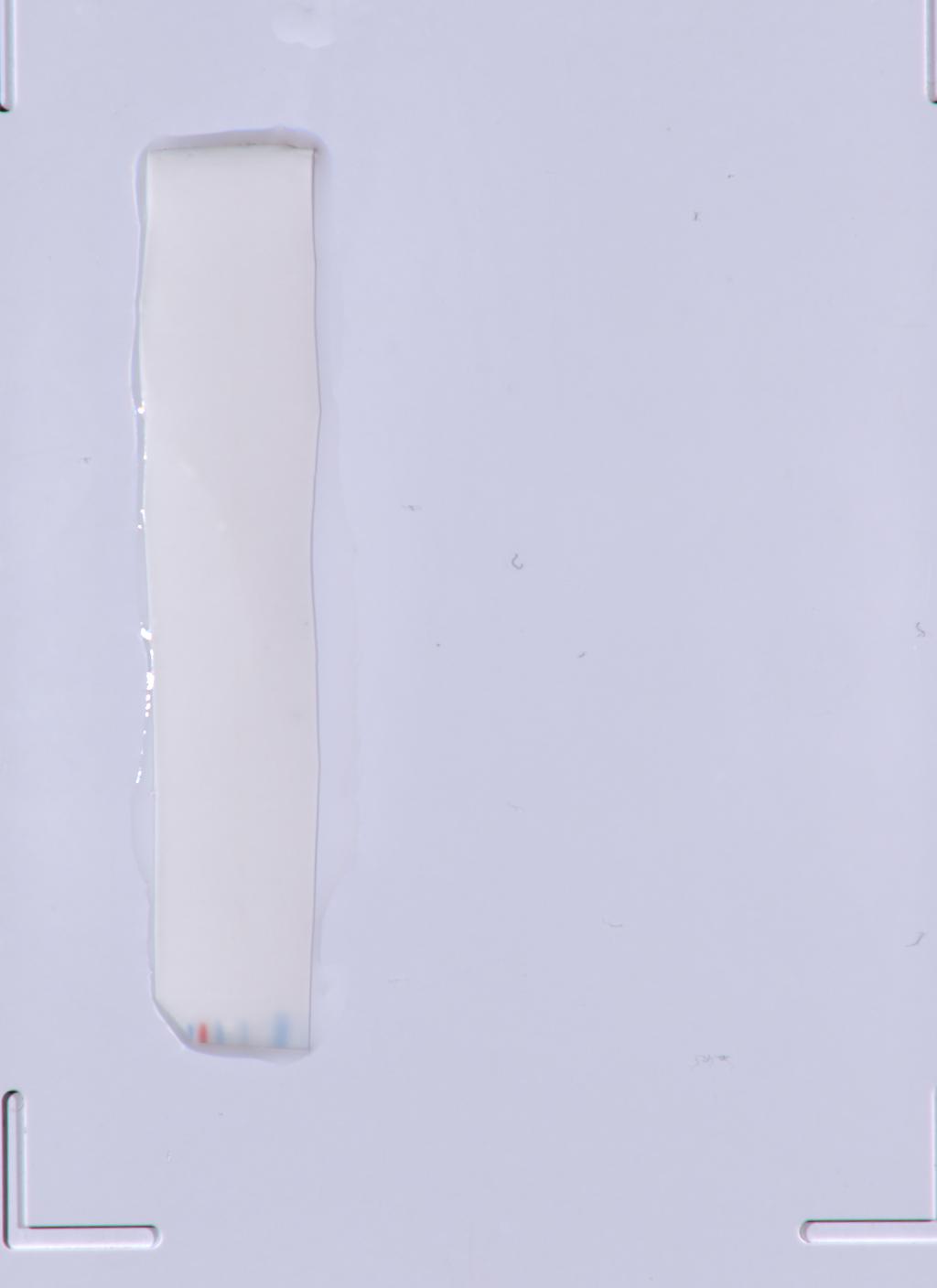

Supplement: Supplementary file 1 — Supplementary Material 1. [file 12985_2024_2385_MOESM1_ESM.zip › xuxiaoying WB/SY5Y p-mTOR NC(╔╧RD,╧┬ SY5Y)2 2021.11.14_18.30.58_Ch/ú¿ú⌐nc-1 2021.11.15_21.23.17_Ch/nc-1 2021.11.15_21.23.17_Ch-Marker.jpg]

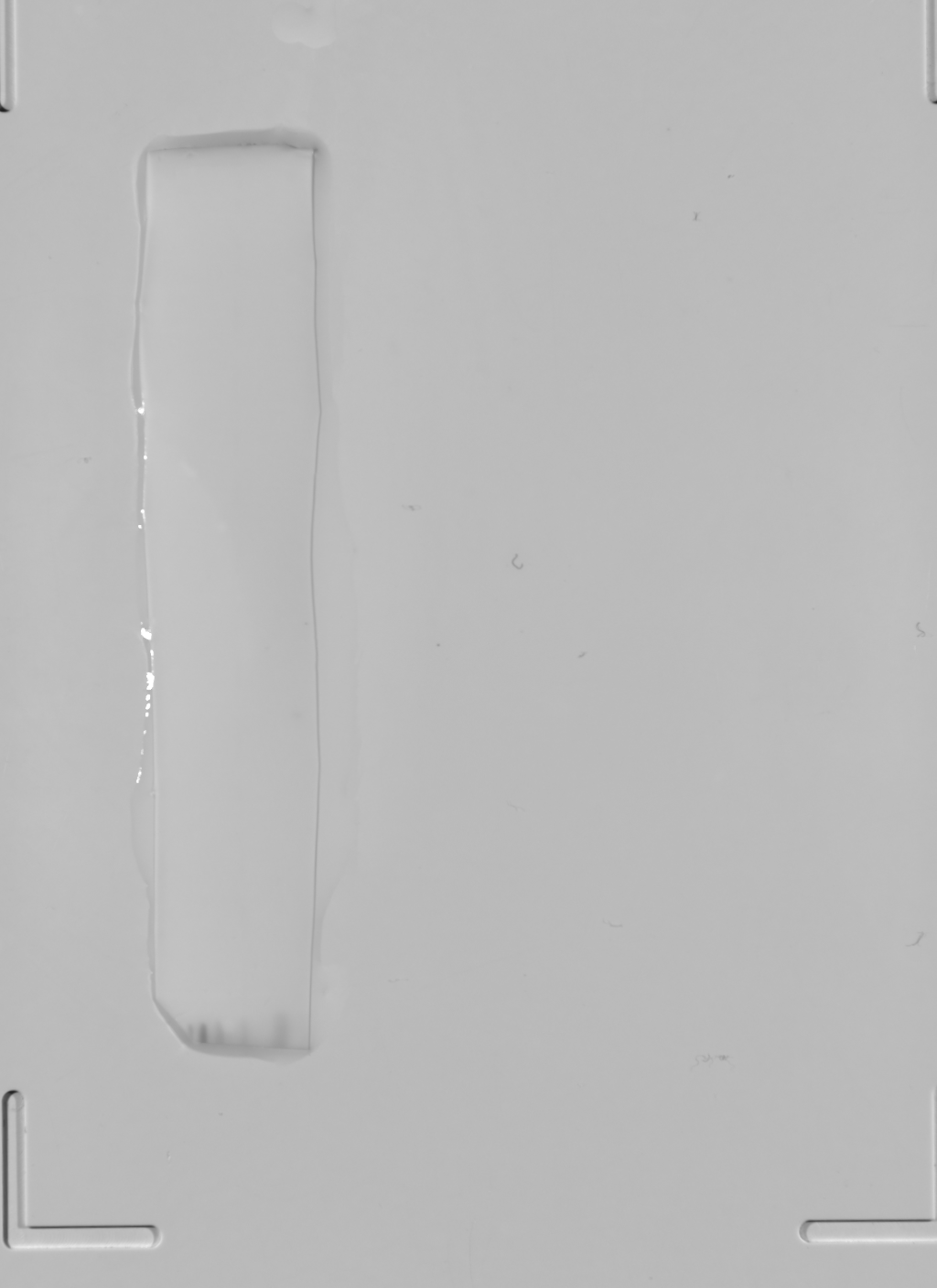

Supplement: Supplementary file 1 — Supplementary Material 1. [file 12985_2024_2385_MOESM1_ESM.zip › xuxiaoying WB/SY5Y p-mTOR NC(╔╧RD,╧┬ SY5Y)2 2021.11.14_18.30.58_Ch/ú¿ú⌐nc-1 2021.11.15_21.23.17_Ch/nc-1 2021.11.15_21.23.17_Ch-Marker.tif]

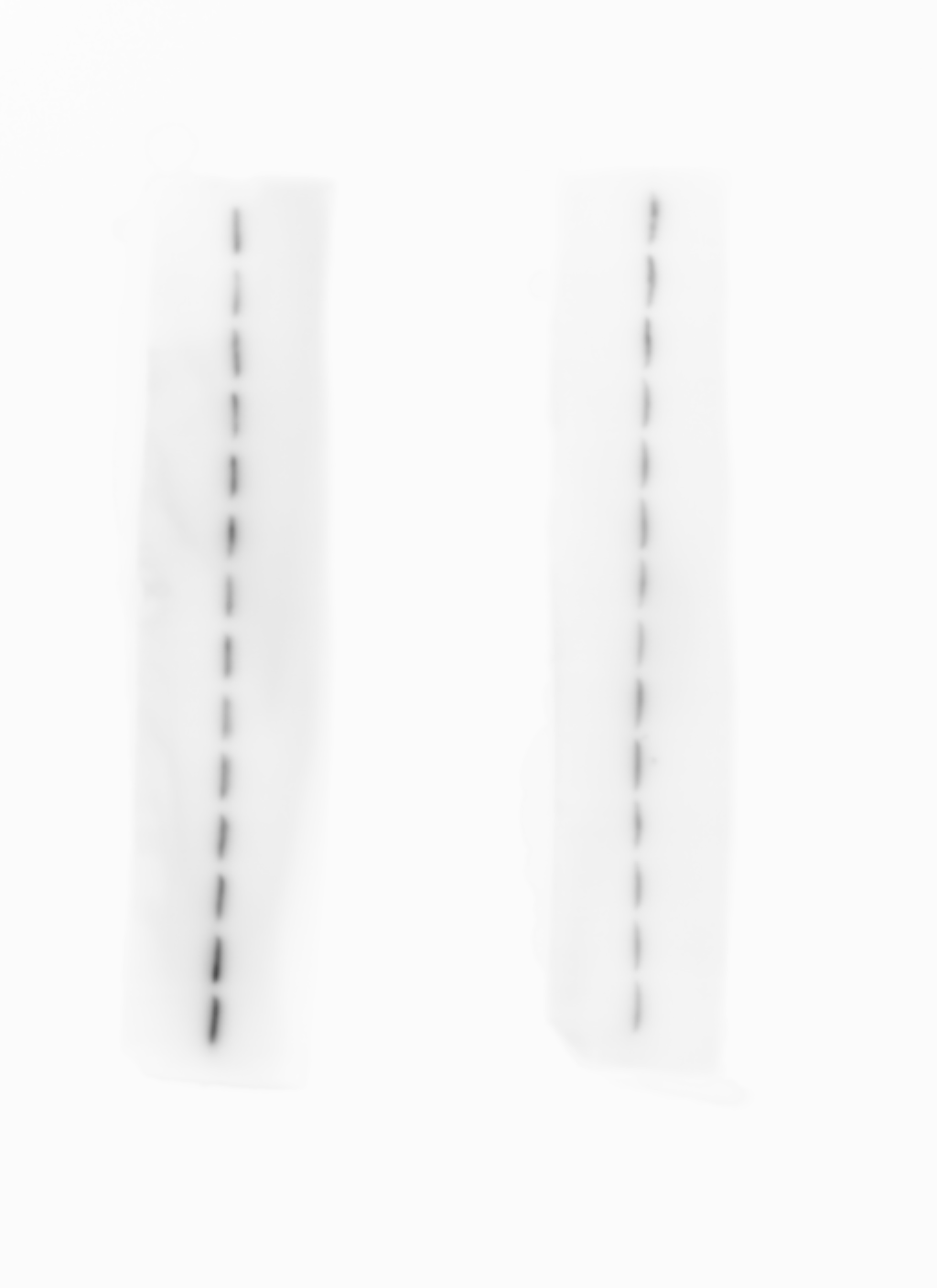

Supplement: Supplementary file 1 — Supplementary Material 1. [file 12985_2024_2385_MOESM1_ESM.zip › xuxiaoying WB/SY5Y p-mTOR NC(╔╧RD,╧┬ SY5Y)2 2021.11.14_18.30.58_Ch/nc2 2021.11.14_18.30.58_Ch.tif]

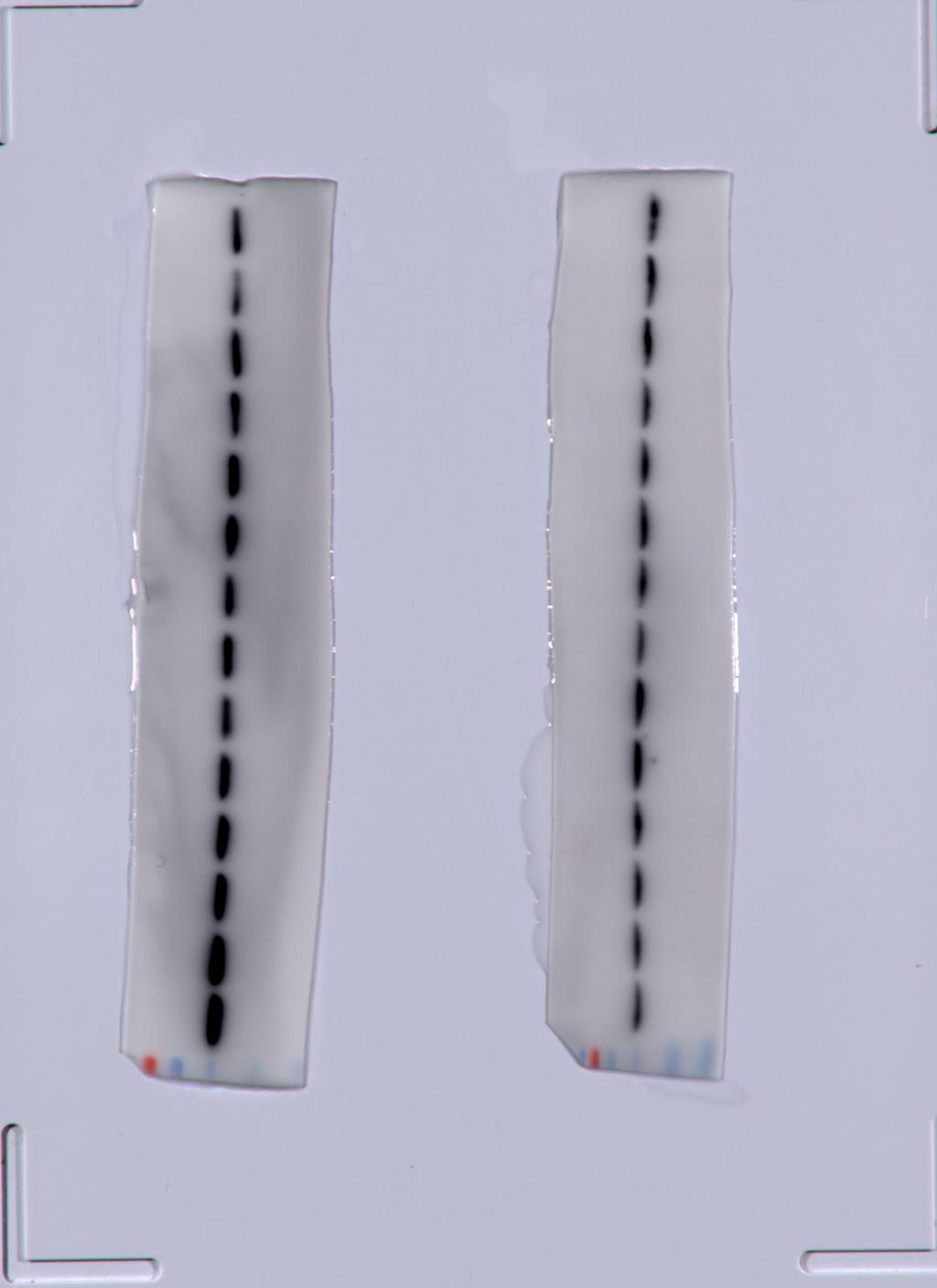

Supplement: Supplementary file 1 — Supplementary Material 1. [file 12985_2024_2385_MOESM1_ESM.zip › xuxiaoying WB/SY5Y p-mTOR NC(╔╧RD,╧┬ SY5Y)2 2021.11.14_18.30.58_Ch/nc2 2021.11.14_18.30.58_Ch+Marker.jpg]

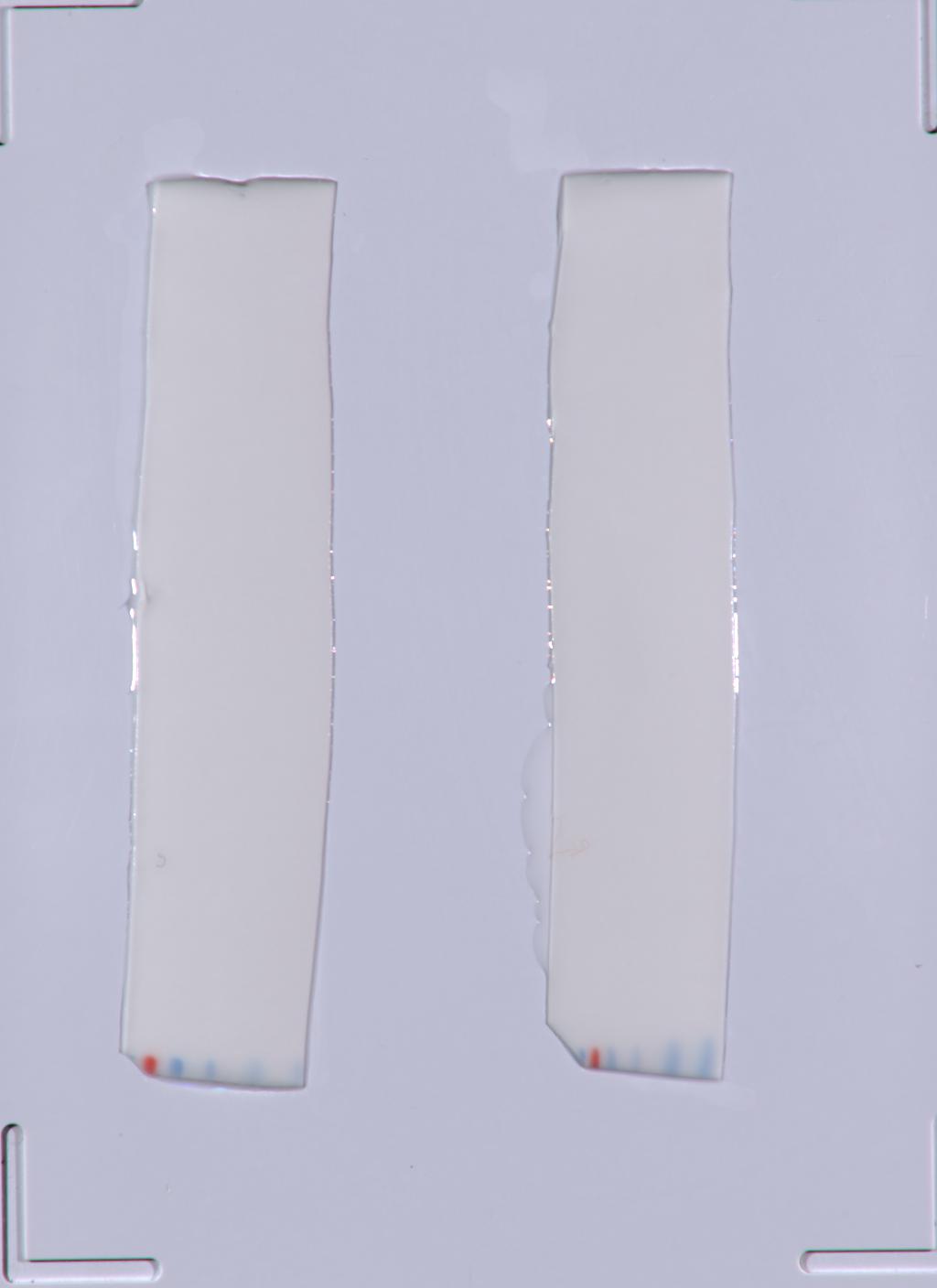

Supplement: Supplementary file 1 — Supplementary Material 1. [file 12985_2024_2385_MOESM1_ESM.zip › xuxiaoying WB/SY5Y p-mTOR NC(╔╧RD,╧┬ SY5Y)2 2021.11.14_18.30.58_Ch/nc2 2021.11.14_18.30.58_Ch-Marker.jpg]

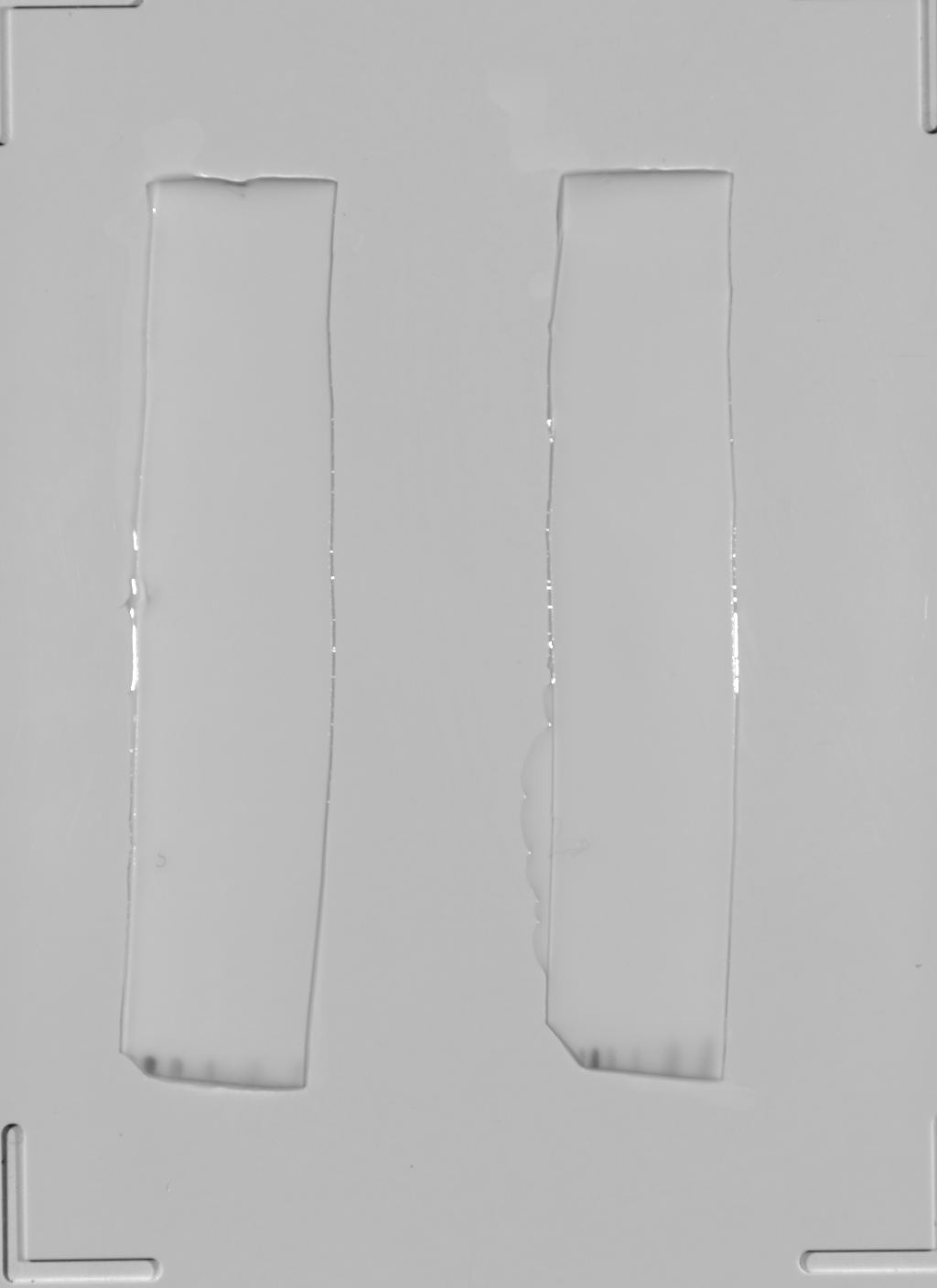

Supplement: Supplementary file 1 — Supplementary Material 1. [file 12985_2024_2385_MOESM1_ESM.zip › xuxiaoying WB/SY5Y p-mTOR NC(╔╧RD,╧┬ SY5Y)2 2021.11.14_18.30.58_Ch/nc2 2021.11.14_18.30.58_Ch-Marker.tif]

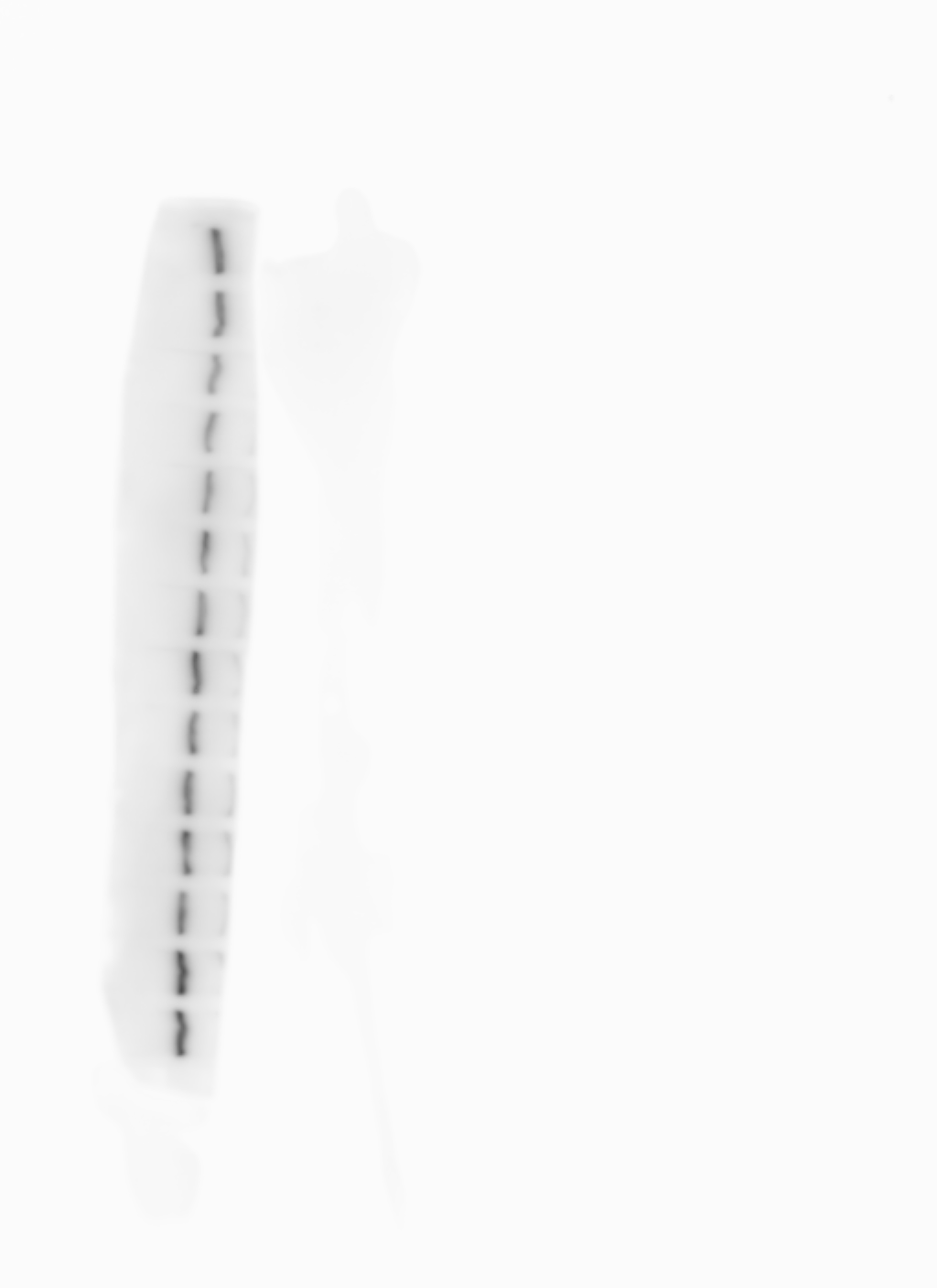

Supplement: Supplementary file 1 — Supplementary Material 1. [file 12985_2024_2385_MOESM1_ESM.zip › xuxiaoying WB/SY5Y p-mtor2-2 2021.11.18_22.57.26_Ch/p-mtor2-2 2021.11.18_22.57.26_Ch.tif]

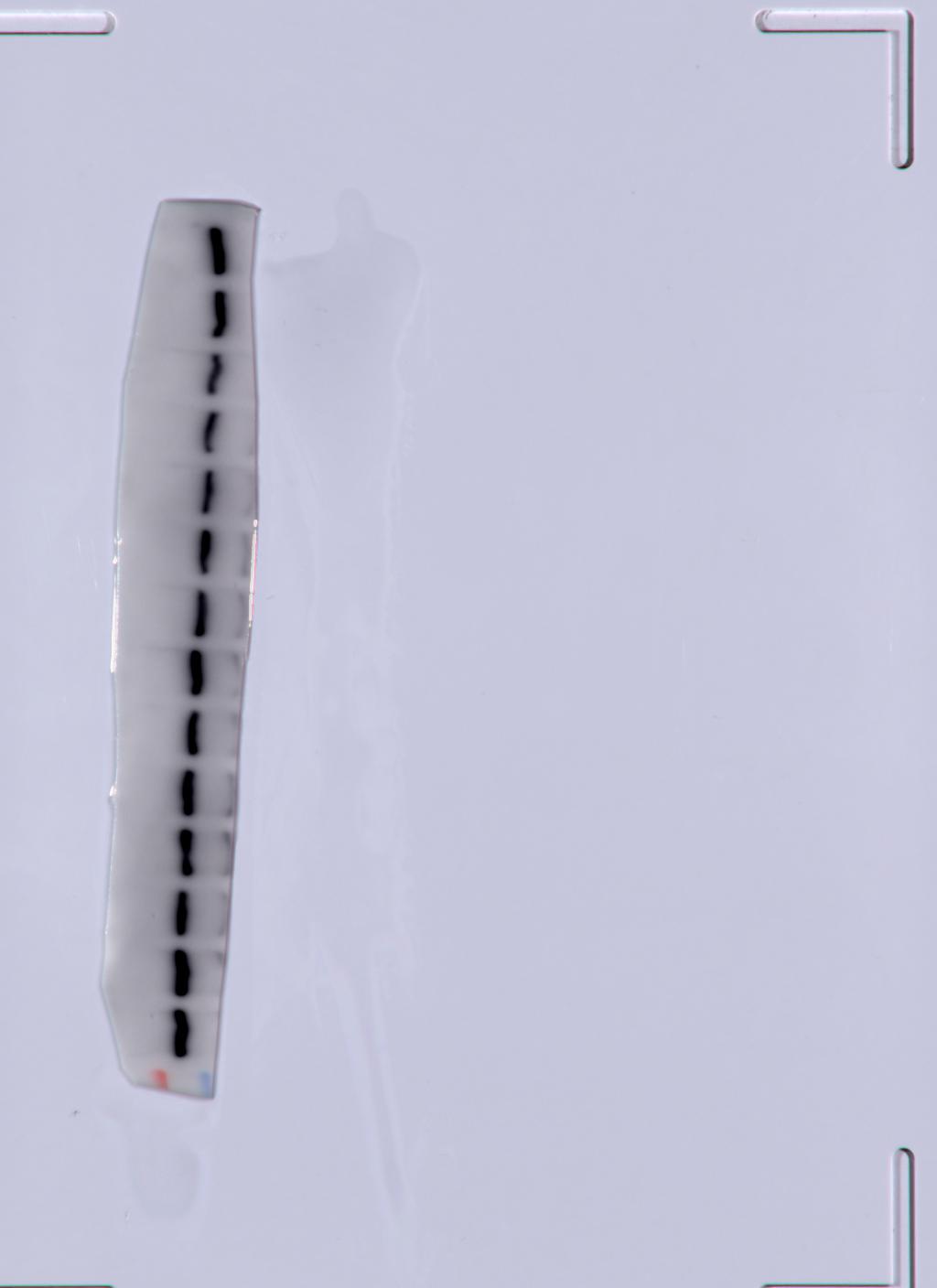

Supplement: Supplementary file 1 — Supplementary Material 1. [file 12985_2024_2385_MOESM1_ESM.zip › xuxiaoying WB/SY5Y p-mtor2-2 2021.11.18_22.57.26_Ch/p-mtor2-2 2021.11.18_22.57.26_Ch+Marker.jpg]

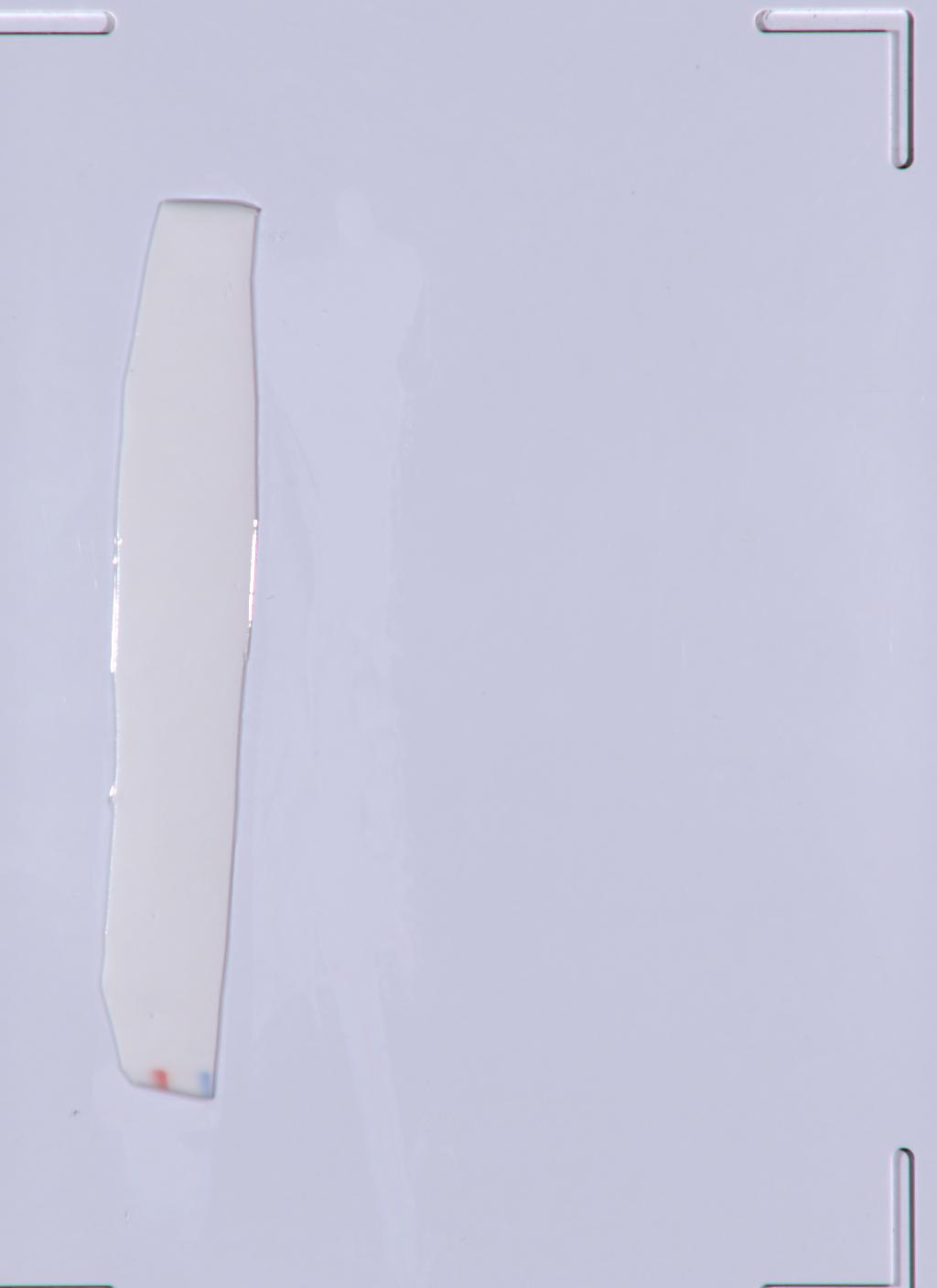

Supplement: Supplementary file 1 — Supplementary Material 1. [file 12985_2024_2385_MOESM1_ESM.zip › xuxiaoying WB/SY5Y p-mtor2-2 2021.11.18_22.57.26_Ch/p-mtor2-2 2021.11.18_22.57.26_Ch-Marker.jpg]

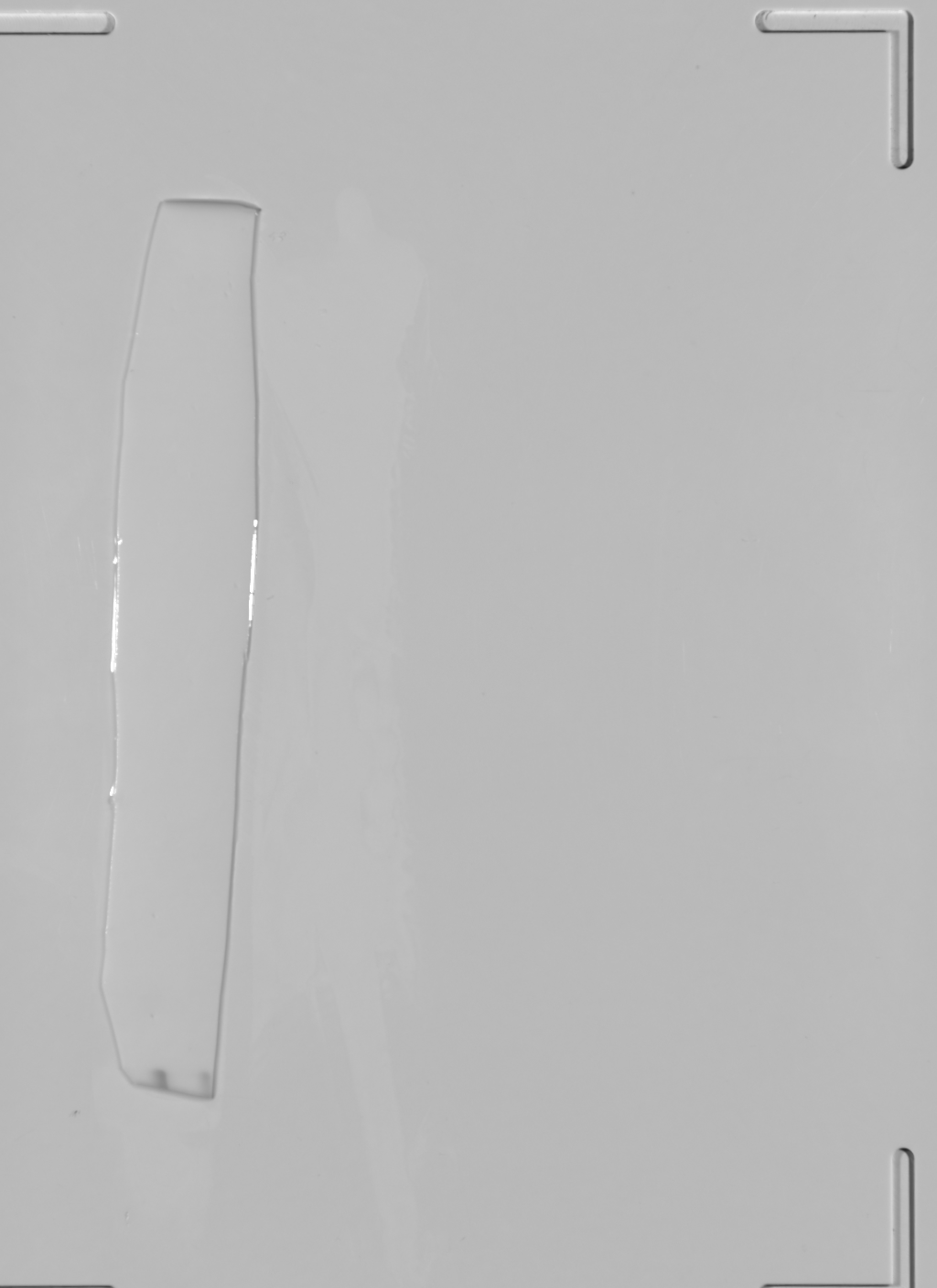

Supplement: Supplementary file 1 — Supplementary Material 1. [file 12985_2024_2385_MOESM1_ESM.zip › xuxiaoying WB/SY5Y p-mtor2-2 2021.11.18_22.57.26_Ch/p-mtor2-2 2021.11.18_22.57.26_Ch-Marker.tif]
